# Regioselective Electrochemical Borylation of Oxygenated Allylic Electrophiles: Method Development and Synthetic Applications

**DOI:** 10.1021/acscentsci.5c01074

**Published:** 2025-09-02

**Authors:** Wan-Chen Cindy Lee, Pierre-Louis Lagueux-Tremblay, Zongbin Jia, Song Lin

**Affiliations:** Department of Chemistry and Chemical Biology, 5922 Cornell University, Ithaca, New York 14853, United States

## Abstract

Allylboronic esters are highly versatile intermediates in organic synthesis. In this work, we report a general and scalable strategy for the regioselective deoxygenative borylation of allylic alcohols, enals, enones, and acrylates, upgrading these abundant functional groups in feedstock chemicals and natural products into value-added borylated synthetic handles. This method achieves efficient C–O bond activation under mild electroreductive conditions, and the effective control of regioselectivity was made possible by optimizing the borylating agent and supporting electrolyte. The utility of this approach was further demonstrated in a series of telescoped synthetic sequences, enabling alcohol and carbonyl transposition, formal cross-coupling of alcohols and aldehydes, allylic amination, and vinylogous homologation. This electrosynthetic protocol offers a broadly applicable, modular route to complex allylboron compounds from simple and readily available starting materials, including terpenoid natural products.

## Graphical abstract

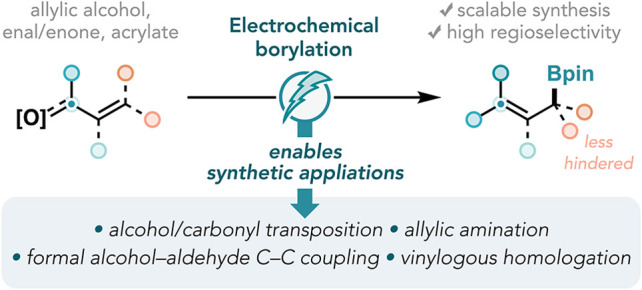

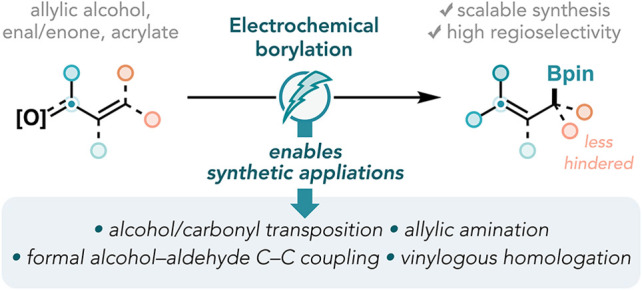

## Introduction

Allylboron compounds are highly useful reagents in organic synthesis,^1^ having been used in a diverse array of reactions including nucleophilic allylations (e.g., carbonyl allylations and Petasis-type allylations)^2–6^ and rearrangements (e.g., 1,2-boron migration and 1,3-borotropic shift),^7–9^ in addition to traditional transformations mediated by organoborons such as cross-couplings^10–12^ and Matteson-type homologations^13–15^ (Scheme 1B). Due to the lack of natural sources for these materials,^16^ various synthetic approaches have been developed for the preparation of allylborons, most often via transition metal catalysis. However, these methods often start from substrates with reactive functional groups that are not frequently encountered in commercial or naturally occurring compounds, such as allylic acetates or carbonates, allenes, and dienes.^1,13,17–25^ While methods have been advanced to convert simple alkenes into allylborons via Pd-catalyzed allylic C–H borylation, their scopes remain currently limited to cyclic and monosubstituted olefins.^26–30^ Therefore, the development of new approaches that could readily convert native functionalities in abundant starting materials to boryl groups will further streamline and expand synthetic access to allylboron synthons.

**Scheme 1:**
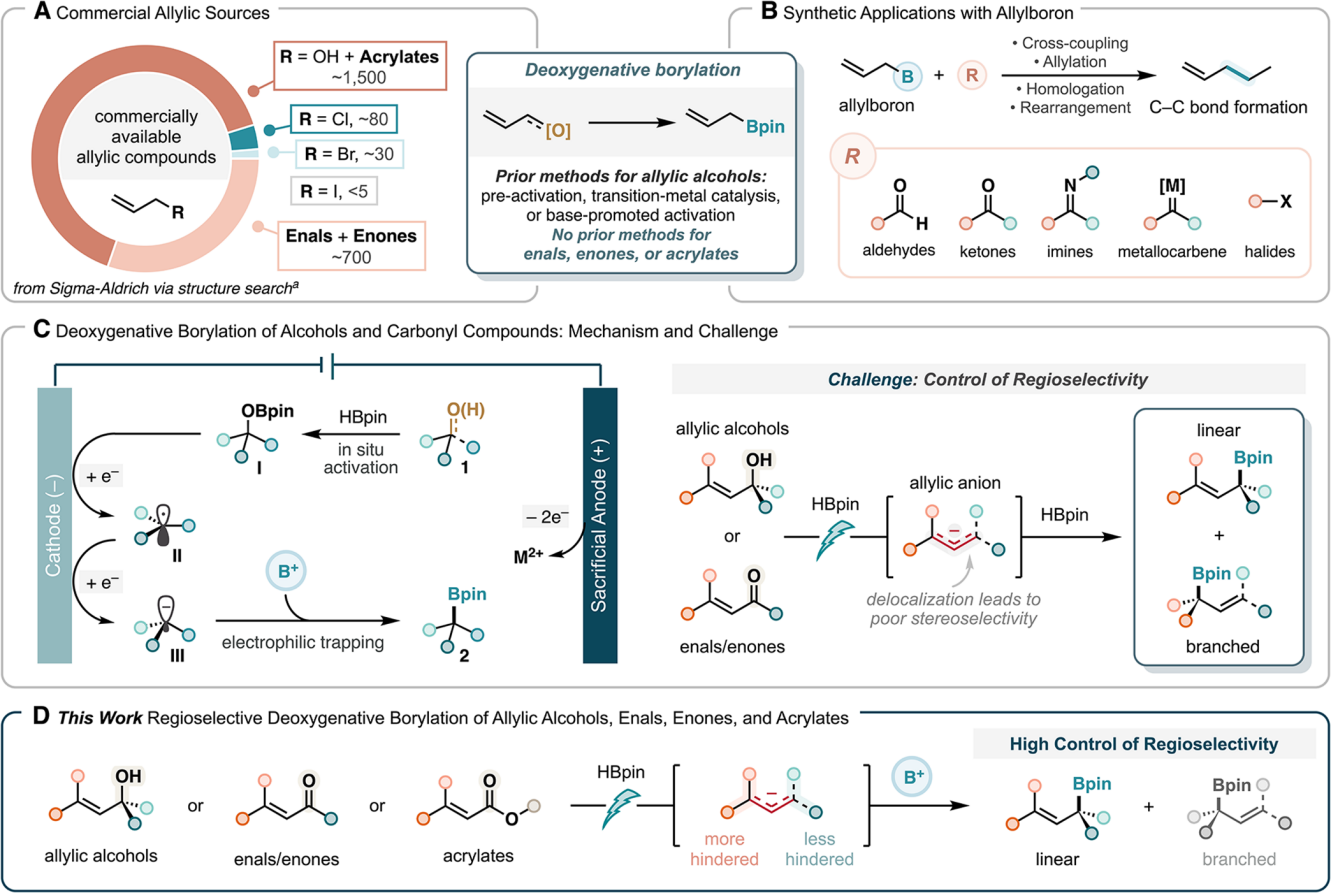
Electroreductive Approach to Regioselective Borylation of Oxygenated Allylic Electrophiles (A) Commercial Allylic Sources. (B) Synthetic Applications with Allylboron. (C) Deoxygenative Borylation of Alcohols and Carbonyl Compounds. (D) Regioselective Deoxygenative Borylation of Allylic Alcohols, Enals, Enones, and Acrylates

a It was difficult to separate allylic alcohols and acrylates through structure search function provided by Sigma-Aldrich. SciFinder search returned ∼5.4 M commercial allylic alcohols and acrylates (vs ∼200 K commercial allylic chlorides); however, we cannot individually verify the viability of all commercial sources or the accuracy of the substructure search.

An attractive strategy for the synthesis of allylboron compounds relies on the deoxygenative borylation of allylically oxygenated alkenes such as allylic alcohols, enals, enones, and acrylates—native functional groups that are prevalent in feedstock chemicals and bioactive natural products with many being commercially available (Scheme 1A).^31–33^ Among these, allylic alcohols are one of the most abundant allyl electrophiles, but they feature a poor −OH leaving group^34^ as well as an acidic H^+^ that may complicate the desired C–O functionalization.^35,36^ Traditionally, allylic alcohols are first transformed to install better leaving groups (e.g., acetate or carbonate)^37–40^ prior to further functionalization. To address the challenges associated with direct alcohol substitution, transition-metal catalysts (such as [Pd] and [Cu])^41–48^ or strong bases (such as Cs_2_CO_3_ and NaOMe)^49,50^ have been employed to activate the alcohol, the borylating agent, or both. In this regard, Szabó’s team, either independently or in collaboration with other researchers, has made seminal contributions by developing a series of borylation methods for allylic alcohols under mild conditions.^41,42,45,49^ Nevertheless, current methods are predominantly limited to the synthesis of sterically favorable primary allylic boronates, with only sparse examples giving access to more hindered secondary congeners.^51^ In particular, the efficient transformation of 1,1,3- and 1,3,3-trisubstituted allylic alcohols to the corresponding boronates has not been demonstrated, despite the prevalence of such motifs in terpenoid natural products.^52^ In recent years, radical-based strategies using preactivated hydroxyl groups (e.g., xanthates, oxalates) have been shown effective in the deoxygenative borylation of simple alcohols, though they have yet to be demonstrated in the context of allylic borylation.^53,54^

In addition to allylic alcohols, α,β-unsaturated carbonyl compounds, such as enals, enones, and acrylates, are also common functional groups found in feedstock chemicals and natural products (Scheme 1A).^33^ While methods have been developed to achieve the deoxygenative borylation of simple, isolated ketones and aldehydes,^55–62^ to date these strategies have yet to be extended to functionalize enals and enones for the synthesis of allylboron products. Similarly, acrylates to the best of our knowledge have yet to be investigated in deoxygenative borylation.^63,64^ Furthermore, the aforementioned metal- or base-promoted borylation protocols for allylic alcohols cannot be applied to carbonyl substrates as they are not amenable to the same mechanism of activation. In general, a unified approach capable of deoxygenating allylic alcohols, carbonyls, and esters remains elusive.

Electrochemistry has recently emerged as an effective approach^65–73^ for the deoxygenation of alcohol and carbonyl compounds.^74–82^ In our initial study,^83^ we found that pinacolborane (HBpin) can serve as an activating agent and boron source for the borylation of benzylic alcohols, aldehydes, and ketones. In this reaction, the substrate first reacts with HBpin—via either *O*-borylation or hydroboration—to generate the borate complex **I** (Scheme 1C). The π-acidity of the boron center induces further polarization of the C–O bond, thereby lowering the potential needed for its activation. An overall two-electron reduction then takes place at the cathode, converting complex **I** first to the corresponding alkyl radical **II** upon loss of BpinO^–^, and then to a carbanion **III**. This nucleophile subsequently reacts with another equivalent of HBpin to afford borylated product **2**. In preliminary exploration, we showed that this strategy could also effect the deoxygenative borylation of allylic alcohols, enals, and enones. However, they remained challenging substrates under the original conditions, giving moderate yields with generally low regio- and stereoselectivities. In most cases, isomeric mixtures of linear and branched allylboronic esters were formed via allylic anion intermediates (Scheme 1C). These shortcomings limit the scope and synthetic utility of the method, especially given that allylboronate isomers can be challenging to separate due to their often-similar polarities and propensity to undergo decomposition during chromatographical purification.

In this work, we present a general and scalable electroreductive approach for the deoxygenative borylation of allylic alcohols, enals, enones, and acrylates (Scheme 1D). By tuning both the borylating agent and the supporting electrolyte, we substantially improved the yield, regioselectivity, and, in some cases, stereoselectivity of the transformation. This method enables efficient differentiation of the two allylic termini via the steric effect, giving the linear product as a single regioisomer in most cases. Leveraging the versatile reactivity of allylborons, we further developed a series of one-pot or telescoped synthetic approaches for allylic transposition, formal alcohol-aldehyde cross-coupling, allylic amination, and vinylogous ketone homologation. These developments provide new synthetic strategies for accessing structurally complex and synthetically challenging targets from readily available starting materials including terpenoid natural products.

## Results and Discussion

### Reaction Strategy and Optimization

While our initial report on the electrochemical deoxygenative borylation showed that allylic alcohols, enals, and enones could be converted to allylboronic esters, the yields were modest in most cases, and the regioselectivities were generally low. Given that regioisomers often have similar polarities and are difficult to separate chromatographically, the formation of isomeric mixtures would significantly limit the synthetic utility of the method, particularly given that many reactions involving allylboron species are regiospecific. Indeed, in our model reaction using 1-vinylcyclohexanol (**1a**), we found that the original protocol failed to effectively differentiate between the tertiary and primary termini of the allylic system, resulting in a 4:1 regioisomeric mixture that could not be separated (Scheme 2). In an effort to achieve effective control over regioselectivity, we hypothesize that the moderate preference for terminal borylation arises predominantly from steric bias during the C–B bond forming step, wherein the electrogenerated allylic anion preferentially reacts at the less hindered primary position. Accordingly, tuning the steric profile of the boron electrophile is expected to improve the selectivity. In addition, we posit that the countercation of the in situ formed allylic anion, which derives from the supporting electrolyte, also influences both the reactivity and regioselectivity of the following step;^84,85^ thus, modifying the identity of the electrolyte could further improve the outcome.

**Scheme 2:**
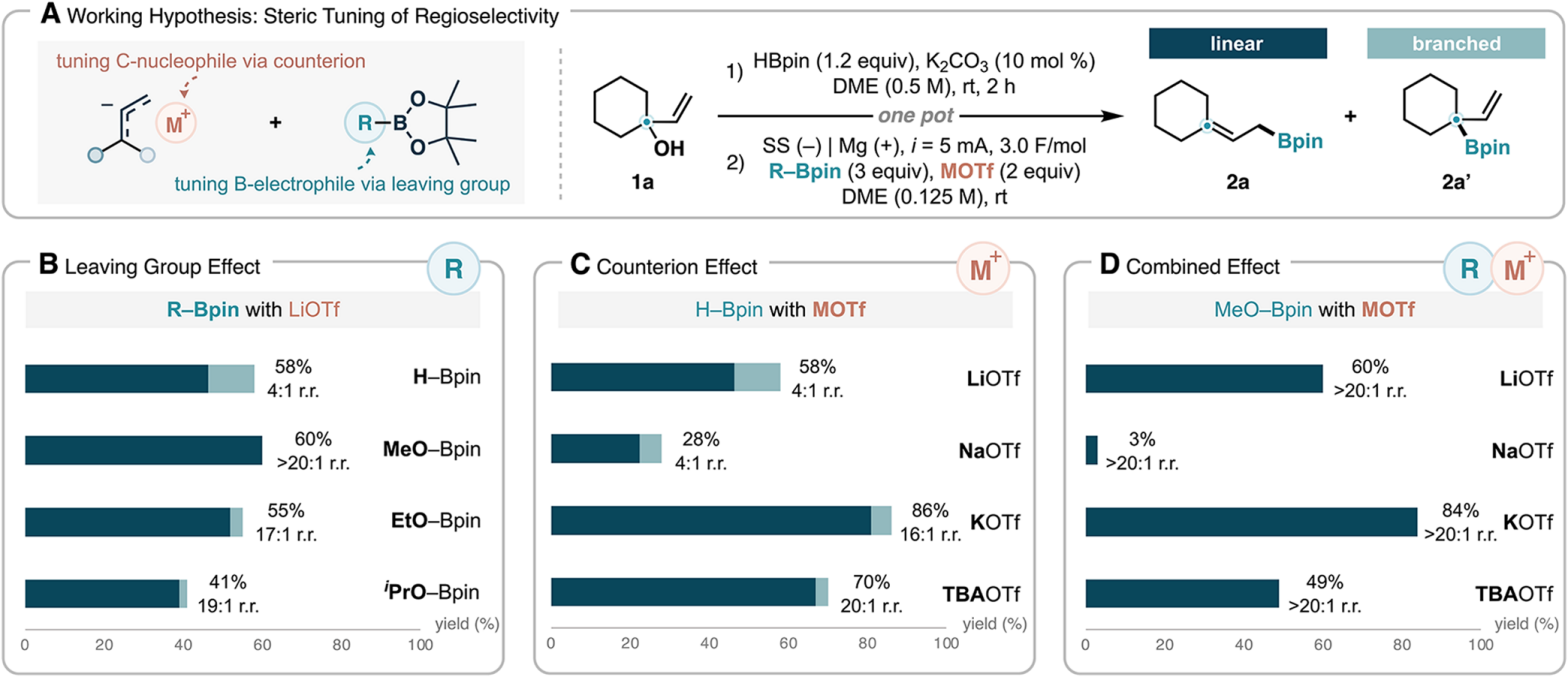
Development and Optimization of Regioselective Deoxygenative Borylation of Allylic Alcohols^a^ (A) Working Hypothesis. (B) Leaving Group Effect. (C) Counterion Effect. (D) Combined Effect

a (1) Preactivation conducted with a 0.50 mmol scale of **1** with HBpin (1.2 equiv), and K_2_CO_3_ (10 mol %) in DME (1.0 mL) at room temperature (rt) for 2 h; (2) With R–Bpin (3.0 equiv), electrolyte (2.0 equiv) in DME (3.0 mL) at rt; Constant current *i* = 5 mA, 3 F/mol; Mg anode and stainless steel (SS) cathode; Undivided cell (ElectraSyn 2.0); Isolated yields; Regioisomeric ratio (r.r.) determined by ^1^H NMR or GC-MS analysis of crude mixture. DME = dimethoxyethane. OTf = trifluoromethanesulfonate. TBA = tetrabutylammonium.

To this end, we envision that increasing the steric bulk of the leaving group on the boron electrophile could provide an ideal means (Scheme 2A), as it allows the regioselectivity to be optimized in a traceless fashion without influencing the structure of the boron group introduced. Based on this rationale, we modified our procedure in which the allylic alcohol is first treated with HBpin and a catalytic amount of K_2_CO_3_ in dimethoxyethane (DME) for 2 h to complete substrate activation, at which point a mixture of a second boron source and the electrolyte is added prior to initiating electrolysis. Following this one-pot procedure, we evaluated three alkoxy-substituted pinacolboranes and observed marked improvements in the regioisomeric ratio (r.r.) of the products compared to HBpin (Scheme 2B). Notably, when commercially available MeO–Bpin was employed, the linear product (**2a**) was obtained in 60% yield effectively as a single regioisomer (r.r. > 20:1). Further replacement of the leaving group with ethoxy (EtO) or isopropoxy (^*i*^PrO) groups resulted in lower yields and slightly decreased regioselectivities. We reason that with these bulky boron electrophiles, the rate of the desired borylation is hampered, allowing for other side reaction to take place competitively.

Following our initial hypothesis, we simultaneously explored the effect of the cation identity in the supporting electrolyte (Scheme 2C). Lithium and sodium triflates both produced identical rr values of 4:1, favoring the linear product (**2a**). Interestingly, the use of a larger potassium salt led to a significant improvement in regioselectivity (r.r. = 16:1) while also providing a higher yield of 86%. Further increasing the cation size to tetrabutyl­ammonium (TBA) resulted in even higher selectivity of r.r. = 20:1, albeit with a slightly decrease in yield. In the relatively nonpolar solvent DME, the cation is likely intimately associated with allylic anion during the borylation; thus, its identify has a substantial influence on not only the reactivity but the regioselectivity of the transformation. We note that the magnesium ion that was slowly generated from the sacrificial anode may also affect the regioselectivity; however, this effect is difficult to estimate and likely small owing to the limited solubility of Mg­(OTf)_2_ in DME.^86^ Building on these findings, we further found that the electrophile and electrolyte effects are additive, as combining the bulkier MeO–Bpin with KOTf in the deoxygenative borylation gave rise to the optimal results with the linear product **2a** in 84% yield with excellent regioselectivity (>20:1) (Scheme 2D). Detailed screening of other reaction parameters is summarized in .

Finally, we found that the standard procedure could be further simplified by mixing all reagents for both steps and assembling the electrolysis setup (including electrodes) in a single operation, followed by prestirring for 2 h before initiating electrolysis. Under these conditions, the borylated **2a** was obtained in similarly high yield (90% isolated) with excellent control of regioselectivity (>20:1 r.r.) See SI section 5.1. This simplified set of conditions was discovered after the substrate scope was completed, and thus, it was not applied to the scope study..

### Substrate Scope

Under optimal conditions, we then explored the reaction scope for the synthesis of a diverse range of allylic pinocolboronic esters (Scheme 3). A variety of tertiary cyclic allylic alcohols containing acetal (**1b**), carbamate (**1c**), siloxane (**1d**), and other substituents (**1e**–**1g**) furnished the corresponding primary allylic boronates (**2b**–**2g**) in good yields with excellent regioselectivities. In particular, cyclohexenol **1f** with a bisallylic motif was selectively transformed to terminally borylated diene **2f** without the observation of any secondary or tertiary functionalization products. Notably, this method also allows for the selective borylation of 1,1,3-trisubstituted allylic alcohols, which feature a pair of tertiary and secondary termini with reduced steric differentiation. Indeed, a panel of acyclic (**1h**) and cyclic (**1i**–**1l**) tertiary allylic alcohols reacted to give secondary allylic boronates (**2h**–**2l**) with near complete regioselectivities. As noted earlier, the transpositional borylation of alcohols from a tertiary to a secondary position has only been demonstrated once, and in low yield, in a previously reported method.^50^ Furthermore, the reaction is also suitable for enone-containing substrates, as shown in the borylation of **1m** to afford **2m** in 57% yield with an 11:1 r.r. Additional cyclic and acyclic allylic alcohols **1n**–**1r** delivered the desired primary or secondary borylation products **2n**–**2r** with generally high levels of regiochemical control. In these cases, two alkene geometrical isomers (*E*:*Z*) were formed in ratios varying between 2:1 and 11:1 favoring the less sterically congested product.

**Scheme 3:**
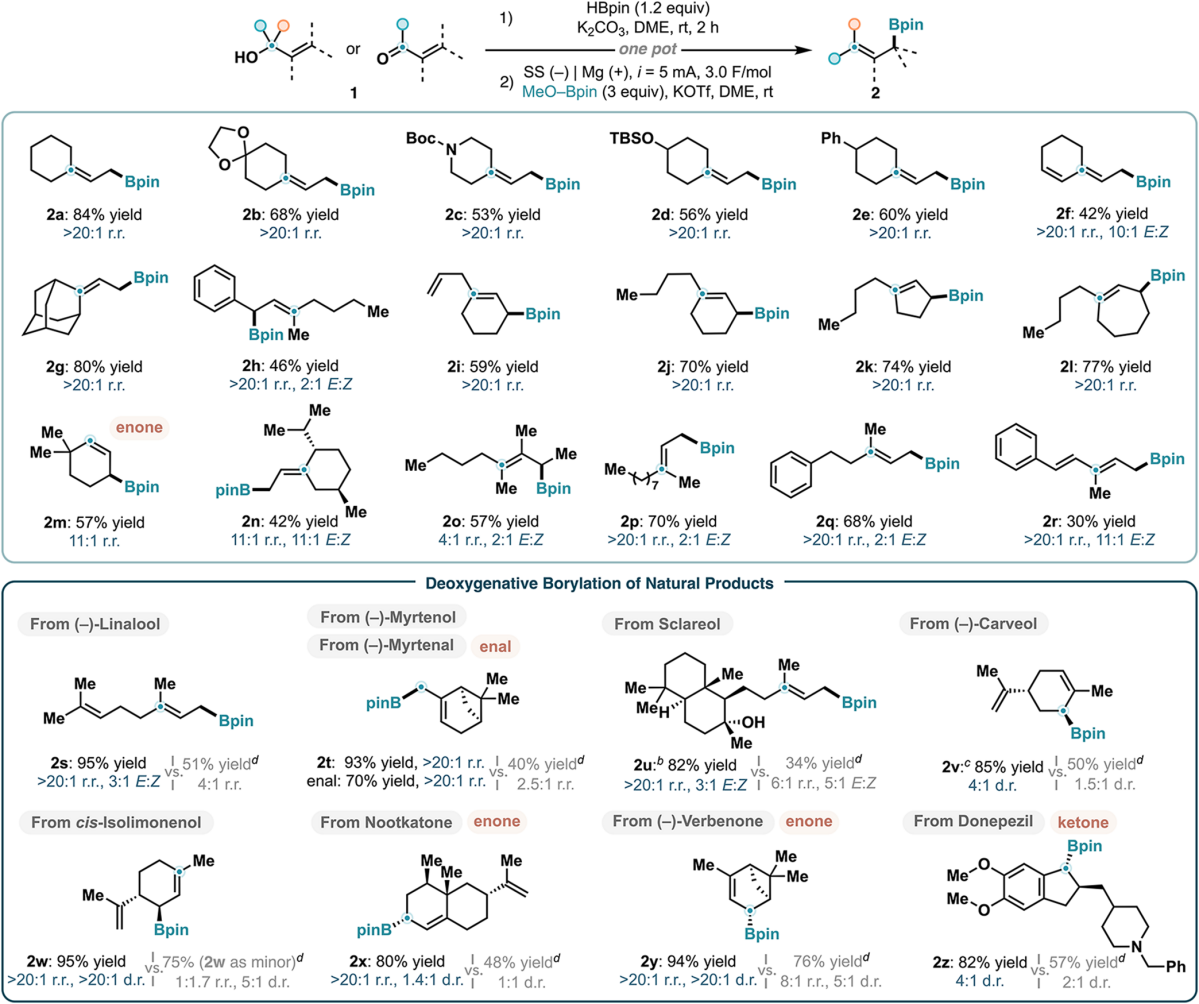
Electroreductive Deoxygenative Borylation of Allylic Alcohols, Enals, and Enones^a^

a (1) Preactivation conducted with a 0.50 mmol scale of **1** with HBpin (1.2 equiv) and K_2_CO_3_ (10 mol %) in DME (1.0 mL) at rt for 2 h; (2) With MeO–Bpin (3.0 equiv) and KOTf (2.0 equiv) in DME (3.0 mL) at rt; Constant current *i* = 5 mA, 3 F/mol; Mg anode and stainless steel (SS) cathode; Undivided cell (ElectraSyn 2.0); Isolated yields; Regioisomeric ratio (r.r.) and diastereomeric ratio (d.r. and *E*:*Z*) determined by ^1^H NMR or GC-MS analysis of crude mixture.
b (1) Preactivation with HBpin (2.5 equiv) and K_2_CO_3_ (5 mol %) in DME (1.0 mL) at 60 °C for 2 h.
c (2) With ^*i*^PrO–Bpin (3.0 equiv).
d First-generation conditions: with Fe (−), HBpin (2.5–3.0 equiv), and LiOTf (2.0 equiv); Data for products **2s**–**2v**, and **2x**–**2z** were directly adopted from our previous publication.^83^

The electrochemical system was subsequently applied to the functionalization of terpenes and related natural products containing allylic alcohols, enals, and enones (Scheme 3). Alcohols Linalool, Myrtenol, Sclareol, Carveol, and Isolimonenol were smoothly converted under the optimized conditions to the corresponding borylated products **2s**–**2w** in excellent yields with near complete regiochemical control. In the case of Sclareol (**1u**), selective borylation occurred exclusively at the allylic alcohol, leaving the unactivated tertiary hydroxy group intact.^87^ Moreover, this method is robust for the deoxygenative functionalization of enals and enones. For example, boronic ester **2t** could also be obtained with >20:1 r.r. from Myrtenal instead of Myrtenol under the standard conditions. Nootkatone, Verbenone, and Donepezil underwent efficient borylation to furnish the corresponding products **2x**–**2z**.

We note that across all examples (**2s**–**2z**), the second-generation protocol consistently outperformed the original conditions, providing the allylic borylation products in higher yields (≥80% vs 34–76%) and with substantially improved regioselectivities (>20:1 vs 1:1.7–8:1). Besides, when diastereoselectivity is concerned, the new method again showed small to significant improvements (**2v**, **2w**, **2x**, **2y**, **2z**) likely due to higher degrees of steric differentiation.

Finally, this electrochemical strategy proved to be effective for the deoxygenative borylation of acrylates (Scheme 4). Using a known protocol,^64^ cinnamate and alkyl-substituted acrylate substrates were first preactivated using HBpin in the presence of EtOMgBr, generating allylic borates that subsequently underwent electrolysis to furnish borylated products **2aa**–**2ac** in good yields with excellent control of both regioselectivity and stereoselectivity.

**Scheme 4:**
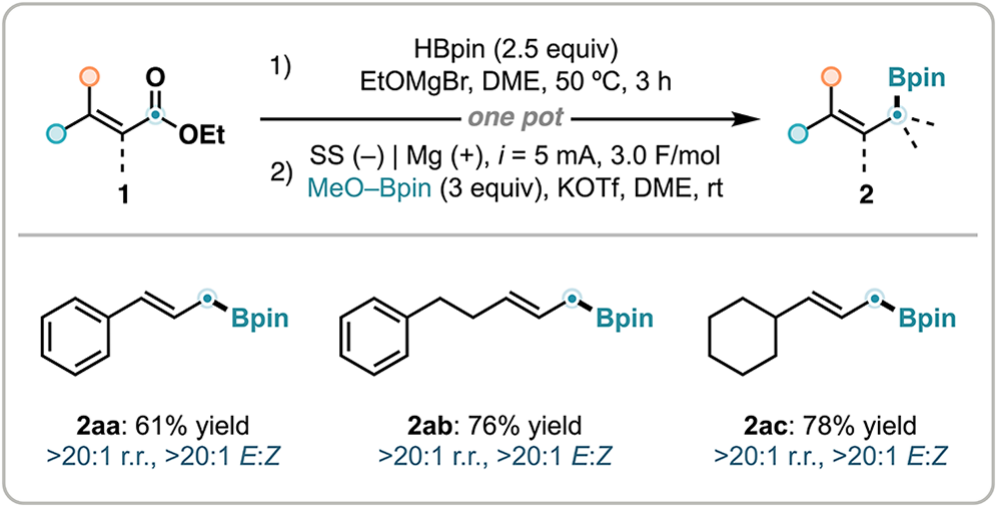
Deoxygenative Borylation of Acrylates^a^

a (1) Preactivation conducted with a 0.50 mmol scale of **1** with HBpin (2.5 equiv) and EtOMgBr (5 mol %, 0.5 M in THF) in DME (0.5 mL) at 50 °C for 3 h; (2) With MeO–Bpin (3.0 equiv) and KOTf (2.0 equiv) in DME (3.5 mL) at rt; Constant current *i* = 5 mA, 3 F/mol; Mg anode and stainless steel (SS) cathode; Undivided cell (ElectraSyn 2.0); Isolated yields; Regioisomeric ratio (r.r.) and diastereomeric ratio (d.r. and *E*:*Z*) determined by ^1^H NMR analysis of crude mixture.

### Scalable Synthesis

The newly developed electrochemical borylation was demonstrated to be scalable to gram and multigram scales with excellent efficiencies, making the method amenable to practical organic synthesis (Scheme 5). Using an ElectraSyn setup, we prepared terpene-derived allylic boronic esters **2s**, **2t**, **2w**, and **2y** on a 5 mmol scale. Owing to the high yield and regioselectivity observed in each case, product purification can be dramatically simplified, which included filtration through Celite using hexanes as the eluent to remove inorganic materials, followed by aqueous washes to eliminate any remaining pinacol borane and water-soluble impurities. Furthermore, scaling up the reaction to 50 mmol, using a custom-built reactor modified from a pressure flask, afforded >10 g of **2y** in 80% yield as a single regio- and diastereomer.

**Scheme 5:**
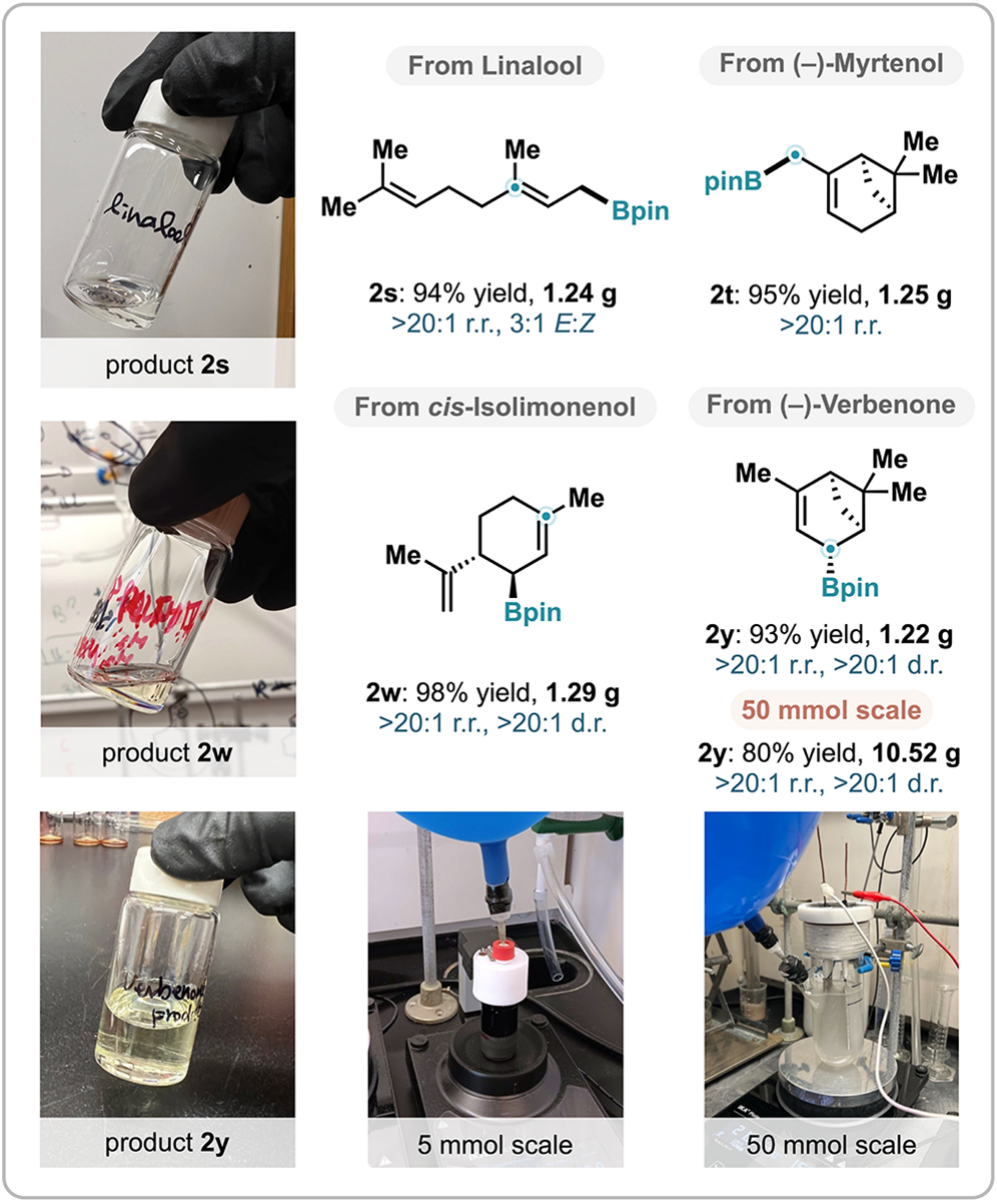
Gram-Scale Electrochemical Borylation

### Synthetic Applications

The ability to access allylic boronic esters in regioisomerically pure form unlocks opportunities to apply this method in various synthetic applications. We first leveraged this reaction in a two-step transposition of allylic alcohols,^88,89^ enabling hydroxyl migration across the allylic system via regioselective deoxygenative borylation followed by oxidative deborylation with aqueous hydrogen peroxide, without the need for purification of the borylated intermediate (Scheme 6A). Sclareol, Isolimonenol, and Geranyllinalool all underwent the planned synthetic sequence to afford rearranged allylic alcohols **3a**–**3c** in high yields. This approach thus transformed readily available and inexpensive allylic alcohols into their isomers that are significantly more expensive or not currently commercially available.

**Scheme 6:**
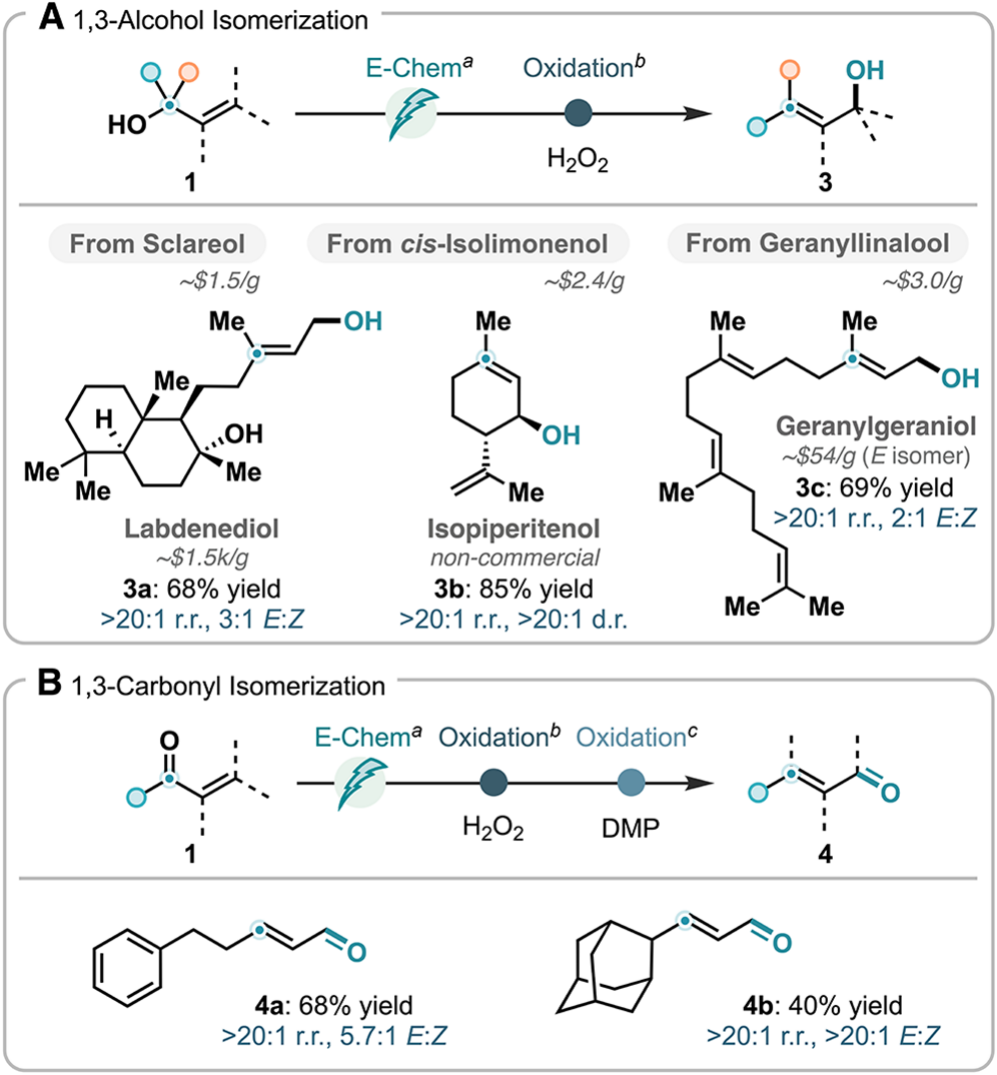
Alcohol and Carbonyl Transpositions (A) 1,3-Alcohol Isomerization. (B) 1,3-Carbonyl Isomerization

a (1) Preactivation conducted with a 0.50 mmol scale of **1** with HBpin (1.2 equiv) and K_2_CO_3_ (10 mol %) in DME (1.0 mL) at rt for 2 h; (2) With MeO–Bpin (3.0 equiv) and KOTf (2.0 equiv) in DME (3.0 mL) at rt; Constant current *i* = 5 mA, 3 F/mol; Mg anode and stainless steel (SS) cathode; Crude mixture was used directly for the next step without workup or purification.
b With H_2_O_2_ (1 mL) and 4N NaOH (1 mL) in THF at rt for 4 h; Followed by aqueous workup.
c With DMP (1.5 equiv) and NaHCO_3_ (20 mg) in DCM at rt for 2 h.

By modifying the oxidation steps, we also achieved 1,3-carbonyl isomerization, transforming an α,β-unsaturated ketone to an α,β-unsaturated aldehyde (Scheme 6B). Upon oxidation of the allylic boronic esters with H_2_O_2_ to generate the corresponding allylic alcohols, subsequent oxidation by Dess-Martin periodinane (DMP) afforded products **4a** and **4b** as single constitutional isomers in synthetically useful yields.

Allylborons are excellent synthons for allylation chemistry in target-oriented synthesis,^1,43,45^ with allyl-containing motifs commonly found in complex natural products and related biologically active agents. We envision that a synthetic sequence consisting of tandem deoxygenative borylation and deborylative allylation would provide efficient access to C­(sp^3^)–C­(sp^3^) coupling products from readily available allylic alcohols and aldehydes, without needing a transition metal catalyst (Scheme 7A). Because the borylation occurs selectively at the less hindered terminus of the allylic system and the subsequent aldehyde allylation proceeds regiospecifically via a six-membered-ring transition state, this process will forge a C–C bond at the more substituted allylic position, furnishing a highly congested alcohol moiety.

**Scheme 7:**
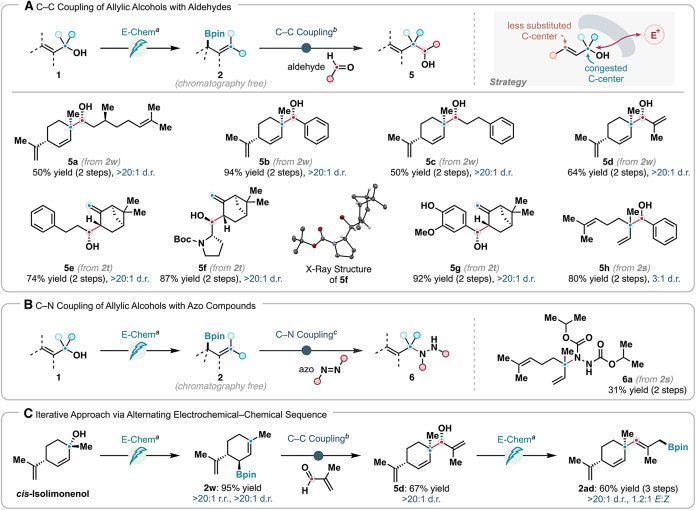
Synthetic Applications of Regioselective Deoxygenative Borylation (A) C–C Coupling of Allylic Alcohols with Aldehydes. (B) C–N Coupling of Allylic Alcohols with Azo Compounds. (C) Iterative Approach via Alternating Electrochemical–Chemical Sequence

a (1) Preactivation conducted with a 0.50 mmol scale of **1** with HBpin (1.2 equiv) and K_2_CO_3_ (10 mol %) in DME (1.0 mL) at rt for 2 h; (2) With MeO–Bpin (3.0 equiv) and KOTf (2.0 equiv) in DME (3.0 mL) at rt; Constant current *i* = 5 mA, 3 F/mol; Mg anode and stainless steel (SS) cathode; Followed by aqueous workup.
b With aldehyde (1.2 equiv) in toluene at 50 °C for 16 h; Followed by aqueous workup and column purification.
c With azo (1.1 equiv) and hydrobenzoin (20 mol %) in AcOH/^*i*^PrOH (1:1) at rt for 16 h.

This strategy was successfully demonstrated in the synthesis of eight homoallylic alcohols (**5a**–**5h**). For example, coupling of *cis*-Isolimonenol with Citronellal, both of which are abundant monoterpene natural products, provided artificial diterpene **5a** in 50% yield as a single stereoisomer. Similarly, the coupling of *cis*-Isolimonenol with benzaldehyde, hydrocinnamaldehyde, and Methacrolein gave rise to products **5b**, **5c**, and **5d**, respectively, forming contiguous tertiary and quaternary stereogenic centers in the process. When Myrtenol, a primary allylic alcohol, was used, an overall transpositional C–C coupling at the more congested secondary allylic position was achieved, generating chiral alcohols **5e**–**5g** upon coupling with hydrocinnamaldehyde, Boc-l-prolinal, or Vanillin. The stereochemistry of **5f** was determined by X-ray crystallography. Linalool, an acyclic tertiary allylic alcohol, also proved suitable for this tandem protocol, giving product **5h** in 80% yield with 3:1 d.r. Moreover, Linalool could engage in a similar sequence with diisopropyl azodicarboxylate as an electrophilic nitrogen source to furnish the corresponding allylic amination product **6a** in 31% yield (Scheme 7B).^3,90^ These transformations highlight the versatility of the deoxygenative borylation platform in enabling diverse C–C and C–N bond formations from abundant terpene-derived scaffolds.

We further adopted this tandem alcohol–aldehyde coupling strategy in the iterative synthesis of skipped polyenes (Scheme 7C). For instance, upon allylation of Methacrolein with electrosynthesized **2w** derived from Isolimonenol, the resultant new allylic alcohol (**5d**) was subjected to a second electrochemical borylation, yielding trienyl boronic ester **2ad** in a combined 60% yield over three steps. These alternating electrochemical-chemical steps could in theory be repeated to further elongate the carbon skeleton in a rapid and selective fashion.

Lastly, we developed a synthetic sequence to enable carbonylvinyl homologation (Scheme 8). In this approach, ketones were first allylated using allylmagnesium bromide, and the resulting tertiary allylic alcohols were then subjected to the 1,3-isomerization protocol described above. This four-step telescoped sequence was applied to a range of naturally occurring ketones, including Menthone, Camphor, Sulcatone, and Carvone, affording the corresponding α,β-unsaturated aldehydes **4c–4f**.

**Scheme 8:**
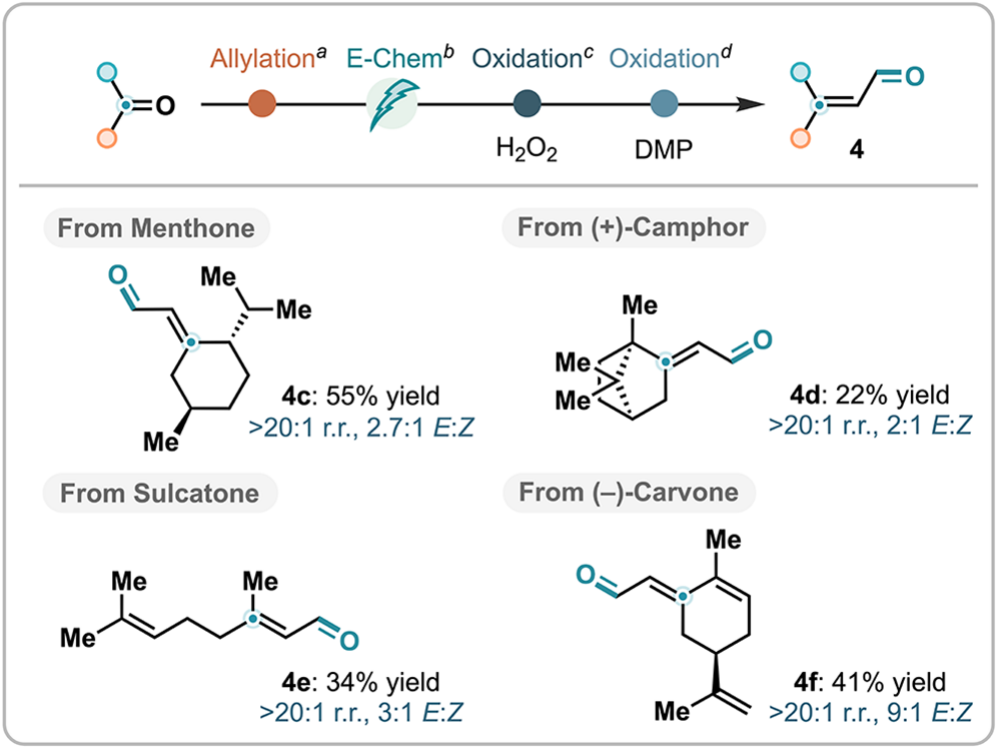
Carbonyl Vinylogous Homologation

a Conducted with a 0.50 mmol scale of ketone with allylmagnesium bromide (1.2 equiv) in THF at rt for 2 h; Followed by aqueous workup.
b (1) Preactivation with HBpin (1.2 equiv) and K_2_CO_3_ (10 mol %) in DME (1.0 mL) at rt for 2 h; (2) With MeO–Bpin (3.0 equiv) and KOTf (2.0 equiv) in DME (3.0 mL) at rt; Constant current *i* = 5 mA, 3 F/mol; Mg anode and stainless steel (SS) cathode; Crude mixture was used directly for the next step without workup or purification.
c With H_2_O_2_ (1 mL) and 4N NaOH (1 mL) in THF at rt for 4 h; Followed by aqueous workup.
d With DMP (1.5 equiv) and NaHCO_3_ (20 mg) in DCM at rt for 2 h.

## Conclusion

In summary, we report an electrochemical protocol for the deoxygenative borylation of allylic alcohols, enals, enones, and acrylates with high efficiency and excellent regioselectivity. Using this approach, a suite of structurally diverse allylboronic esters were synthesized from readily available starting materials, including terpenoid natural products and their derivatives. The synthetic significance of this method was further demonstrated in a series of tandem synthetic procedures to enable alcohol and carbonyl transposition, formal cross-coupling of alcohols and aldehydes, allylic amination, and vinylogous homologation. Given the prevalence of allylic alcohols and carbonyl-containing compounds in natural products and synthetic intermediates, along with the broad versatility of allylboronic esters, we anticipate that this electrosynthetic strategy will provide rapid, stereoselective, and scalable routes from feedstock chemicals to complex organic molecules.

## Supplementary Material





## Notes

Experimental procedures; characterization data including NMR spectra for all new compounds; X-ray crystallographic data for the selected compound (PDF)
Transparent Peer Review report available (PDF)
