# Supplementary material for: Regioselective Electrochemical Borylation of Oxygenated Allylic Electrophiles: Method Development and Synthetic Applications
Source: ACS Cent Sci. 2025 Sep 2;11(10):1959–68. doi: 10.1021/acscentsci.5c01074 (PMC12550627; doi:10.1021/acscentsci.5c01074)
Supplement: Supplementary file 1 [file oc5c01074_si_001.pdf]

Supporting Information

Regioselective Electrochemical Borylation of Oxygenated Allylic Electrophiles: Method Development and Synthetic Applications

Wan-Chen Cindy Lee,[†] Pierre-Louis Lagueux-Tremblay,[†] Zongbin Jia, and Song Lin*

[1]Department of Chemistry and Chemical Biology, Cornell University, Ithaca, New York 14850, US

[†]Authors contributed equally

Table of Contents

1. General Information **S3**

2. Optimization of Reaction Conditions **S4**

2.1. Optimization of Electrochemical Deoxygenative Borylation S4

3. Reaction Setup **S6**

3.1. Experimental Setup for Electrochemical Deoxygenative Borylation S6

4. Synthesis of Allylic Alcohols **S7**

4.1. General Procedure for Preparation of Allylic Alcohols S7

5. Synthesis of Allylboronic Esters **S9**

5.1. General Procedure for E-Borylation of Allylic Alcohols, Enals, and Enones S9

5.2. General Procedure for E-Borylation of Acrylates S18

5.3. General Procedures for Gram-Scale E-Borylation S20

6. Synthetic Applications of Electrochemical Deoxygenative Borylation **S24**

6.1. General Procedures for Alcohol Transposition S24

6.2. General Procedures for Carbonyl Transposition S26

6.3. General Procedures for C–C Coupling S27

6.4. General Procedures for C–N Coupling S30

6.5. General Procedures for Vinylogous Homologation S31

7. X-ray Crystallography **S33**

8. References **S35**

9. NMR Spectra **S36**

1. General Information

All reactions were performed in anhydrous solvents. Flash chromatography was performed using silica gel 60 (230-400 mesh) from SiliCycle. Thin layer chromatography (TLC) experiments were performed on aluminum sheets coated with silica gel 60 F_{254}. Commercial reagents were purchased from Sigma Aldrich, Alfa Aesar, Acros, TCI, AK Scientific, AmBeed, Combi-Blocks and Oakwood and used as received without further purification. NMR spectra were acquired using Varian Inova 400 MHz, and Inova 500 MHz spectrometer. Spectra were processed using MNova software. Chemical shifts are reported in parts per million (ppm), coupling constants (*J*) in Hz and are calibrated to residual protonated solvent (^{1}H NMR: $CDCl_3$ = δ 7.26 and ^{13}C NMR: $CDCl_3$ = δ 77.16). Data are represented as follows: chemical shift, multiplicity (br = broad, s = singlet, d = doublet, t = triplet, q = quartet, d = doublet of doublets, td = triplet of doublets, qd = quartet of doublets, m = multiplet), coupling constants in Hertz (Hz), integration. Regioisomeric ratio (r.r.) and diastereomeric ratio (d.r.) determined by ^{1}H NMR or GC-MS analysis of crude reaction mixture and all spectral data correspond to the major isomer. The high-resolution mass spectrometry (HRMS) data were obtained on a Thermo Fisher Scientific Exactive series DART Mass Spectrometer and Agilent 8860 GC System with an Agilent 5977B Mass Selective Detector. The X-ray diffraction data were collected using Rigaku XtaLAB Synergy diffractometer. All electrolysis reactions were performed using ElectraSyn 2.0.

2. Optimization of Reaction Conditions

2.1. Optimization of Electrochemical Deoxygenative Borylation

Table S1. Optimization of Electrochemical Deoxygenative Borylation

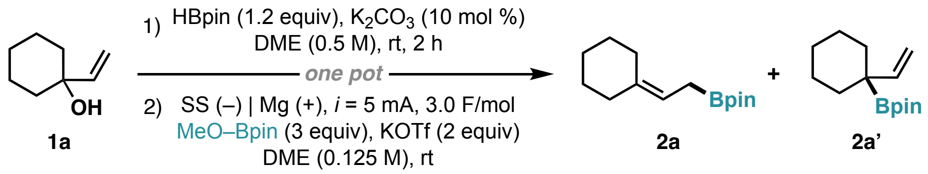

Entry	Deviation from Standard Conditions	Yield of **2a**
1	none	84% yield, >20:1 r.r.
2	no electricity	0% yield
3	Cu (–) I Mg (+)	82% yield, >20:1 r.r.
4	Zn (–) I Mg (+)	75% yield, >20:1 r.r.
5	Fe (–) I Mg (+)	71% yield, >20:1 r.r.
6	MeO–Bpin (1 equiv)	42% yield, >20:1 r.r.
7	MeO–Bpin (2 equiv)	82% yield, >20:1 r.r.
8	MeO–Bpin (4 equiv)	86% yield, >20:1 r.r.
9	KOTf (1 equiv)	63% yield, >20:1 r.r.
10	KOTf (3 equiv)	84% yield, >20:1 r.r.
11	9-BBN (2 equiv) instead of MeO–Bpin	0% yield
12	LiOTf instead of KOTf; *i* = 2 mA, 3.0 F/mol	60% yield, >20:1 r.r.
13	LiOTf instead of KOTf; *i* = 20 mA, 3.0 F/mol	52% yield, >20:1 r.r.
14	LiOTf instead of KOTf; *i* = 5 mA, 2.0 F/mol	50% yield, >20:1 r.r.
15	LiOTf instead of KOTf; *i* = 5 mA, 4.0 F/mol	33% yield, >20:1 r.r.

Table S2. Optimization of Electrochemical Deoxygenative Borylation

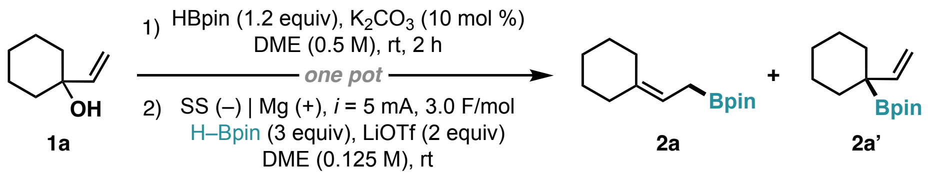

Entry	Deviation from Standard Conditions	Yield of **2a**
1	none	58% yield, 4:1 r.r.
2	SS (–) I Zn (+)	30% yield, 4:1 r.r.
3	SS (–) I Fe (+)	6% yield, >20:1 r.r.
4	SS (–) I Al (+)	24% yield, 1.5:1 r.r.
5	$LiPF_6$ (2 equiv) instead of LiOTf	25% yield, 2.5:1 r.r.
6	$LiClO_4$ (2 equiv) instead of LiOTf	53% yield, 4:1 r.r.
7	LiCl (2 equiv) instead of LiOTf	52% yield, 3:1 r.r.
8	LiTFSI (2 equiv) instead of LiOTf	74% yield, 3:1 r.r.
9	SS (–) I C (+) with DIPEA (2 equiv)	29% yield, 2.5:1 r.r.
10	divided cell	37% yield, 4:1 r.r.
11	THF instead of DME	70% yield, 3:1 r.r.
12	DME/TPPA (3:1) instead of DME	37% yield, 5:1 r.r.

3. Reaction Setup

3.1. Experimental Setup for Electrochemical Deoxygenative Borylation

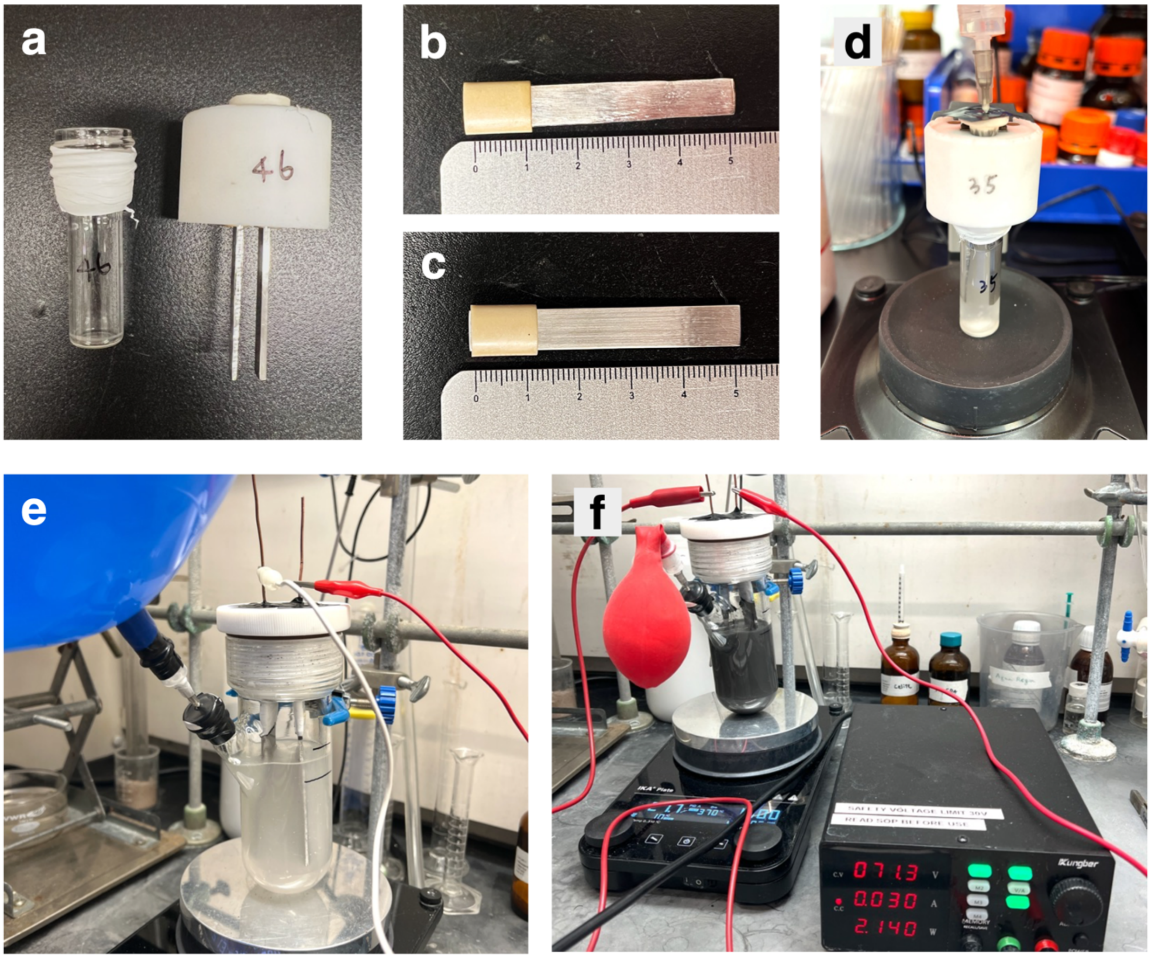

Figure S1. Experimental Setup. (a) A ElectraSyn 5 mL vial wrapped with PTFE tape and the cap containing a Mg anode and a SS cathode. (b) A magnesium electrode. (c) A stainless steel electrode from IKA. (d) Assembled ElectraSyn vial and cap setup during electrolysis. (e) Setup for 50 mmol scale reaction. (f) Reaction mixture after electrolysis on 50 mmol scale.

4. Synthesis of Allylic Alcohols

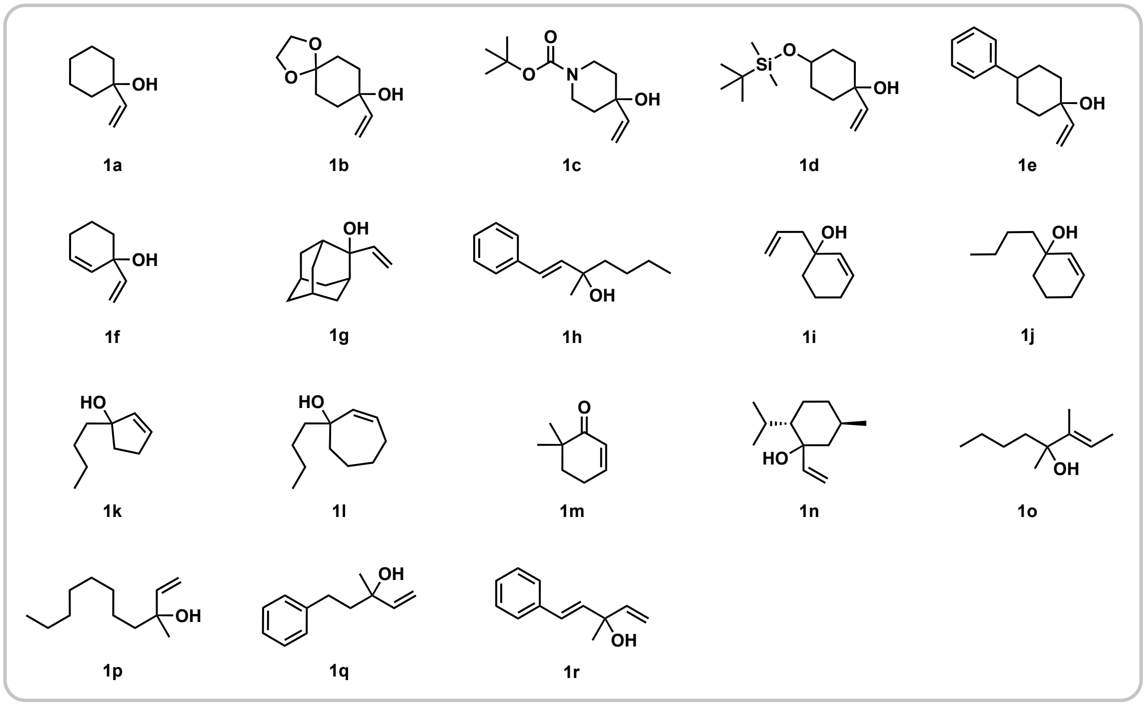

Known compounds: **1a**,[1] **1b**,[2] **1c**,[3] **1e**,[4] **1f**,[5] **1g**,[6] **1h**,[7] **1i**,[5] **1j**,[8] **1k**,[9] **1l**,[9] **1m**,[10] **1n**,[11] **1p**,[12] **1q**,[13] **1r**.[14]

4.1. General Procedure for Preparation of Allylic Alcohols

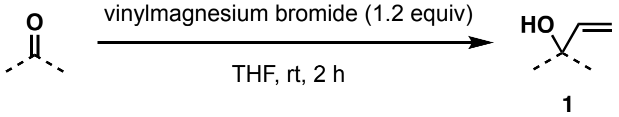

General Procedure: A 25 mL round-bottom flask was charged with 5 mmol of carbonyl compound in anhydrous THF (0.2 M). The mixture was cooled to 0 ºC in an ice bath and then vinylmagnesium bromide (1.0 M in THF, 1.2 equiv) was added dropwise to the mixture. The solution was warmed to rt and stirred for 2 h, and then quenched by the saturated NH_4Cl. The crude mixture was extracted with diethyl ether (3 x 50 mL) and the combined organic layers were washed with brine, dried over anhydrous Na_2SO_4, filtered, and the mixture was concentrated under reduced pressure. The crude residue was purified by flash chromatography (EtOAc:hexanes = 1:10) to afford product **1**.

4-((*tert*-Butyldimethylsilyl)oxy)-1-vinylcyclohexan-1-ol (1d)

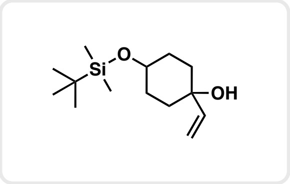

Yield: 53%. Colorless liquid. ^{1}H NMR (400 MHz, $CDCl_3$) isomer mixture: δ 6.07 – 5.86 (m, 1H), 5.26 (dt, *J* = 17.3, 1.6 Hz, 1H), 5.04 (ddd, *J* = 10.7, 3.7, 1.3 Hz, 1H), 3.97 – 3.55 (m, 1H), 1.98 – 1.72 (m, 2H), 1.70 – 1.65 (m, 3H), 1.56 – 1.48 (m, 2H), 1.43 – 1.19 (m, 2H), 0.89 (d, *J* = 2.1 Hz, 9H), 0.05 (d, *J* = 6.7 Hz, 6H). ^{13}C NMR (101 MHz, $CDCl_3$) major isomer: δ 145.5, 111.9, 71.8, 70.9, 35.3, 31.2, 26.0, 18.3. HRMS-(DART+) calculated for C14H29O2Si $[M+H]^+$: 257.1931, found: 257.1924.

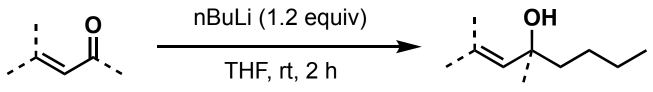

General Procedure: A 25 mL round-bottom flask was charged with 5 mmol of allylic carbonyl compound in anhydrous THF (0.2 M). The mixture was cooled to 0 ºC in an ice bath and then n-buthyllithium (2.5 M in hexanes, 1.2 equiv) was added dropwise to the mixture. The solution was warmed to rt and stirred for 2 h, and then quenched by the saturated NH_4Cl. The crude mixture was extracted with diethyl ether (3 x 50 mL) and the combined organic layers were washed with brine, dried over anhydrous Na_2SO_4, filtered, and the mixture was concentrated under reduced pressure. The crude residue was purified by flash chromatography (EtOAc:hexanes = 1:10) to afford product **1**.

(*E*)-3,4-Dimethyloct-2-en-4-ol (1o)

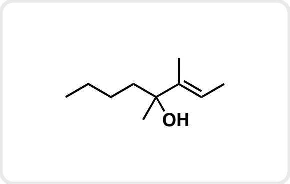

Yield: 71%. Colorless liquid. ^{1}H NMR (400 MHz, $CDCl_3$) isomer mixture: δ 5.54 (m, 1H), 1.65 – 1.58 (m, 6H), 1.54 (ddd, *J* = 9.3, 6.4, 2.1 Hz, 2H), 1.37 (s, 1H), 1.32 – 1.25 (m, 5H), 1.20 – 1.06 (m, 2H), 0.88 (t, *J* = 7.3 Hz, 3H). ^{13}C NMR (126 MHz, $CDCl_3$) major isomer: δ 140.7, 117.1, 75.9, 40.3, 27.7, 26.3, 24.9, 23.2, 14.2. HRMS-(DART+) calculated for C10H21O $[M+H]^+$: 157.1587, found: 157.1582.

5. Synthesis of Allylboronic Esters

5.1. General Procedure for E-Borylation of Allylic Alcohols, Enals, and Enones

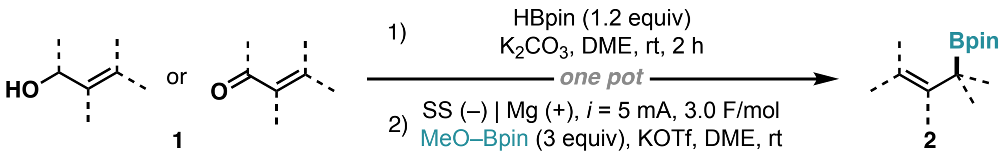

Step 1 –Pre-activation of Alcohol/Enal/Enone: In a nitrogen-filled glovebox, an 8 mL vial equipped with a magnetic stir bar was charged with compound **1** (0.5 mmol, 1.0 equiv), K_2CO_3 (0.05 mmol, 0.1 equiv), and 1.0 mL of DME. To this suspension, HBpin (0.6 mmol, 1.2 equiv) was added dropwise via syringe. *(**Caution**: hydrogen gas evolution was observed upon addition.)* The reaction mixture was stirred at room temperature for 2 h in the glovebox.

Step 2 – Electrochemical Borylation: To a 5 mL ElectraSyn vial, KOTf (1.0 mmol, 2.0 equiv) and MeO–Bpin (1.5 mmol, 3.0 equiv) were added. The reaction mixture from *Step 1* was transferred into the ElectraSyn vial using 3 mL of DME. The vial was sealed with a preassembled cap equipped with a magnesium anode and a stainless steel cathode, removed from the glovebox, and maintained under a nitrogen atmosphere (balloon). Electrolysis was carried out at room temperature under a constant current of 5 mA until a total charge of 3 F/mol was passed (~8 h).

***Step 1 and Step 2 could be combined:** In a nitrogen-filled glovebox, a 5 mL ElectraSyn vial equipped with a magnetic stir bar was charged with compound **1** (0.5 mmol, 1.0 equiv), K_2CO_3 (0.05 mmol, 0.1 equiv), KOTf (1.0 mmol, 2.0 equiv), MeO–Bpin (1.5 mmol, 3.0 equiv), and DME (4.0 mL). To this suspension, HBpin (0.6 mmol, 1.2 equiv) was added dropwise via syringe. *(**Caution**: hydrogen gas evolution was observed upon addition.)* The vial was sealed with a preassembled cap equipped with a magnesium anode and a stainless steel cathode, removed from the glovebox, and maintained under a nitrogen atmosphere (balloon). The reaction mixture was stirred at room temperature for 2 h, followed by electrolysis at room temperature under a constant current of 5 mA until a total charge of 3 F/mol was passed (~8 h).

Workup: Upon completion, the reaction was quenched by the addition of 30 μL of water (~3 drops) and stirred for 5 min. *(**Caution**: failure to quench the reaction may cause fire upon exposure of the magnesium electrode to air.)* The mixture was then diluted with 20 mL of hexanes and passed through a short pad of Celite to remove the electrolyte. The filtrate was concentrated under reduced pressure, and the crude residue was further purified by flash chromatography (EtOAc:hexanes = 1:20) to afford the borylated product **2**. *(**Note:** purification should be performed promptly to avoid product decomposition on silica.)*

2-(2-Cyclohexylideneethyl)-4,4,5,5-tetramethyl-1,3,2-dioxaborolane (2a)

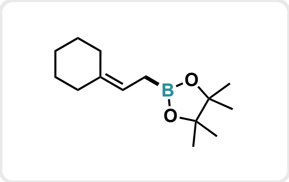

Yield: 84%. Colorless oil. >20:1 r.r.. ^{1}H NMR (500 MHz, $CDCl_3$) δ 5.16 (t, *J* = 7.7 Hz, 1H), 2.07 (dt, *J* = 12.8, 5.8 Hz, 4H), 1.59 (d, *J* = 7.8 Hz, 2H), 1.53 – 1.45 (m, 6H), 1.23 (s, 12H). ^{13}C NMR (126 MHz, $CDCl_3$) δ 139.8, 115.1, 83.2, 37.1, 28.8, 28.7, 27.7, 27.1, 24.9. ^{11}B NMR (160 MHz, $CDCl_3$) δ 33.37. HRMS-(DART+) calculated for C14H26BO2 $[M+H]^+$: 237.2021, found: 237.2022.

2-(2-(1,4-Dioxaspiro[4.5]decan-8-ylidene)ethyl)-4,4,5,5-tetramethyl-1,3,2-dioxaborolane (2b)

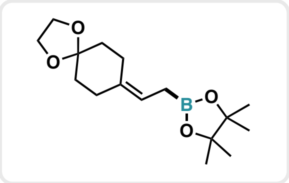

Yield: 68%. Colorless oil. >20:1 r.r.. ^{1}H NMR (500 MHz, $CDCl_3$) δ 5.23 (tt, *J* = 7.8, 1.3 Hz, 1H), 3.93 (s, 4H), 2.21 (dt, *J* = 12.3, 6.2 Hz, 4H), 1.65 – 1.57 (m, 6H), 1.21 (s, 12H). ^{13}C NMR (126 MHz, $CDCl_3$) δ 136.8, 116.8, 109.3, 83.2, 64.4, 36.3, 35.4, 33.5, 25.0, 24.8. ^{11}B NMR (160 MHz, $CDCl_3$) δ 33.00. HRMS-(DART+) calculated for C16H28BO4 $[M+H]^+$: 295.2075, found: 295.2071.

***tert*-Butyl 4-(2-(4,4,5,5-tetramethyl-1,3,2-dioxaborolan-2-yl)ethylidene)piperidine-1-carboxylate (2c)**

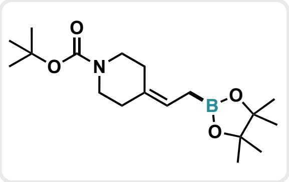

Yield: 53%. Colorless oil. >20:1 r.r.. ^{1}H NMR (400 MHz, $CDCl_3$) δ 5.29 (t, *J* = 7.8 Hz, 1H), 3.34 (t, *J* = 5.7 Hz, 4H), 2.12 (dt, *J* = 17.7, 5.8 Hz, 4H), 1.59 (d, *J* = 7.7 Hz, 2H), 1.43 (s, 9H), 1.20 (s, 12H). ^{13}C NMR (101 MHz, $CDCl_3$) δ 154.9, 135.0, 118.0, 83.3, 79.4, 46.1, 44.6, 35.8, 28.6, 28.2, 24.8. ^{11}B NMR (160 MHz, $CDCl_3$) δ 33.32. HRMS-(DART+) calculated for C18H33BNO4 $[M+H]^+$: 338.2497, found: 338.2493.

***tert*-Butyldimethyl((4-(2-(4,4,5,5-tetramethyl-1,3,2-dioxaborolan-2-yl)ethylidene)cyclohexyl)oxy) silane (2d)**

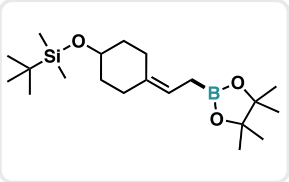

Yield: 56%. Colorless oil. >20:1 r.r.. ^{1}H NMR (500 MHz, $CDCl_3$) δ 5.18 (tt, *J* = 7.7, 1.3 Hz, 1H), 3.78 (tt, *J* = 7.9, 3.5 Hz, 1H), 2.39 (ddt, *J* = 13.8, 6.7, 4.1 Hz, 1H), 2.33 – 2.22 (m, 1H), 1.95 (dddd, *J* = 13.4, 9.8, 3.9, 1.5 Hz, 1H), 1.90 – 1.83 (m, 1H), 1.73 – 1.66 (m, 2H), 1.58 (d, *J* = 7.6 Hz, 2H), 1.41 (dddd, *J* = 16.2, 8.1, 6.8, 4.0 Hz, 2H), 1.22 (s, 12H), 0.87 (s, 9H), 0.03 (s, 6H). ^{13}C NMR (126 MHz, $CDCl_3$) δ 138.1, 115.8, 83.2, 70.3, 36.9, 35.8, 33.2, 26.0, 24.87, 24.86, 24.7, 18.3, -4.5. ^{11}B NMR (160 MHz, $CDCl_3$) δ 33.03. HRMS-(DART+) calculated for C20H40BO3Si $[M+H]^+$: 367.2834, found: 367.2830.

4,4,5,5-Tetramethyl-2-(2-(4-phenylcyclohexylidene)ethyl)-1,3,2-dioxaborolane (2e)

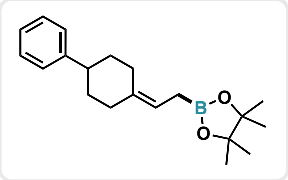

Yield: 60%. Colorless oil. >20:1 r.r.. ^{1}H NMR (500 MHz, $CDCl_3$) δ 7.34 – 7.27 (m, 2H), 7.24 – 7.17 (m, 3H), 5.31 (tt, J = 7.8, 2.0 Hz, 1H), 2.78 – 2.66 (m, 2H), 2.35 (ddt, J = 13.4, 4.3, 2.3 Hz, 1H), 2.21 (dddd, J = 15.6, 11.2, 3.5, 1.9 Hz, 1H), 1.97 (dddd, J = 14.9, 12.7, 4.1, 2.2 Hz, 2H), 1.88 (dddd, J = 16.5, 11.9, 3.0, 1.6 Hz, 1H), 1.68 (d, J = 7.8 Hz, 2H), 1.58 – 1.45 (m, 2H), 1.28 (s, 12H). ^{13}C NMR (126 MHz, $CDCl_3$) δ 147.4, 138.4, 128.4, 126.9, 125.9, 116.0, 83.2, 44.9, 36.8, 36.0, 34.9, 28.4, 24.9. ^{11}B NMR (160 MHz, $CDCl_3$) δ 33.57. HRMS-(DART+) calculated for C20H30BO2 $[M+H]^+$: 313.2333, found: 313.2332.

(*E*)-2-(2-(Cyclohex-2-en-1-ylidene)ethyl)-4,4,5,5-tetramethyl-1,3,2-dioxaborolane (2f)

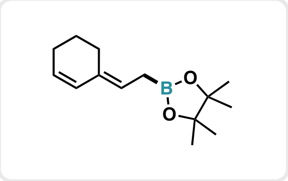

Yield: 42%. Colorless oil. >20:1 r.r.. 10:1 *E*:*Z*. ^{1}H NMR (400 MHz, $CDCl_3$) δ 6.05 (dt, J = 9.8, 2.1 Hz, 1H), 5.62 (dt, J = 9.2, 4.2 Hz, 1H), 5.36 (t, J = 8.1 Hz, 1H), 2.33 – 2.24 (m, 2H), 2.08 (q, J = 5.7 Hz, 2H), 1.67 (dt, J = 12.4, 6.7 Hz, 4H), 1.24 (s, 12H). ^{13}C NMR (101 MHz, $CDCl_3$) δ 135.2, 131.4, 126.3, 121.7, 83.3, 25.7, 25.3, 24.9, 22.6. ^{11}B NMR (160 MHz, $CDCl_3$) δ 33.05. HRMS-(DART+) calculated for C14H24BO2 [M+H]+: 235.1864, found: 235.1862.

2-(2-((1*r*,3*r*,5*R*,7*S*)-Adamantan-2-ylidene)ethyl)-4,4,5,5-tetramethyl-1,3,2-dioxaborolane (2g)

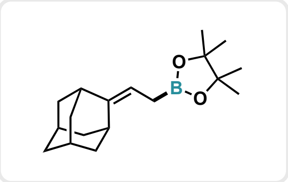

Yield: 80%. Colorless oil. >20:1 r.r.. ^{1}H NMR (400 MHz, $CDCl_3$) δ 5.13 (t, J = 7.8 Hz, 1H), 2.80 – 2.74 (m, 1H), 2.34 (t, J = 3.3 Hz, 1H), 1.94 – 1.91 (m, 2H), 1.85 – 1.80 (m, 6H), 1.72 (td, J = 11.8, 5.5 Hz, 4H), 1.57 (d, J = 7.5 Hz, 2H), 1.24 (s, 12H). ^{13}C NMR (101 MHz, $CDCl_3$) δ 147.7, 109.8, 83.1, 40.5, 40.0, 38.8, 37.5, 32.0, 28.8, 24.9. ^{11}B NMR (160 MHz, $CDCl_3$) δ 32.72. HRMS-(DART+) calculated for C18H30BO2 $[M+H]^+$: 289.2333, found: 289.2328.

(*E*)-4,4,5,5-Tetramethyl-2-(3-methyl-1-phenylhept-2-en-1-yl)-1,3,2-dioxaborolane (2h)

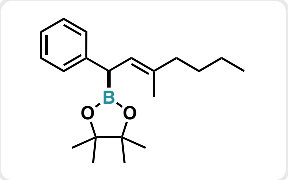

Yield: 46%. Colorless oil. >20:1 r.r.. 2:1 *E*:*Z*. ^{1}H NMR (500 MHz, $CDCl_3$) isomer mixture: δ 7.30 – 7.24 (m, 4H), 7.17 – 7.12 (m, 1H), 5.49 (m, 1H), 3.43 (t, J = 9.2 Hz, 1H), 2.22 – 2.00 (m, 2H), 1.75 (d, J = 1.1 Hz, 1H), 1.66 (d, J = 1.4 Hz, 2H), 1.43 – 1.36 (m, 2H), 1.34 – 1.28 (m, 2H), 1.22 (s, 12H), 0.91 (m, 3H). ^{13}C NMR (126 MHz, $CDCl_3$) major isomer: δ 142.9, 135.7, 128.4, 128.2, 125.2, 124.1, 83.4, 39.6, 30.4, 24.7, 24.6, 22.4, 16.4, 14.1. ^{11}B NMR (160 MHz, $CDCl_3$) δ 32.52. HRMS-(DART+) calculated for C20H32BO2 $[M+H]^+$: 315.2490, found: 315.2487.

2-(3-Allylcyclohex-2-en-1-yl)-4,4,5,5-tetramethyl-1,3,2-dioxaborolane (2i)

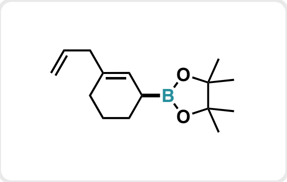

Yield: 59%. Colorless oil. >20:1 r.r.. ^{1}H NMR (500 MHz, $CDCl_3$) δ 5.78 (ddt, J = 16.9, 10.1, 6.8 Hz, 1H), 5.46 (dt, J = 3.3, 1.6 Hz, 1H), 5.04 – 4.93 (m, 2H), 2.73 – 2.60 (m, 2H), 1.89 (dq, J = 5.1, 3.0 Hz, 2H), 1.77 (s, 1H), 1.68 (ddd, J = 9.3, 5.3, 2.7 Hz, 2H), 1.61 – 1.51 (m, 2H), 1.23 (s, 12H). ^{13}C NMR (126 MHz, $CDCl_3$) δ 137.5, 134.9, 122.3, 115.3, 83.1, 42.9, 28.2, 24.9, 24.8, 24.1, 23.1. ^{11}B NMR (160 MHz, $CDCl_3$) δ 33.47. HRMS-(DART+) calculated for C15H26BO2 $[M+H]^+$: 249.2020, found: 249.2016.

2-(3-Butylcyclohex-2-en-1-yl)-4,4,5,5-tetramethyl-1,3,2-dioxaborolane (2j)

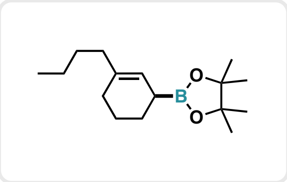

Yield: 70%. Colorless oil. >20:1 r.r.. ^{1}H NMR (500 MHz, $CDCl_3$) δ 5.39 (dt, J = 3.4, 1.5 Hz, 1H), 1.98 – 1.83 (m, 4H), 1.75 (s, 1H), 1.71 – 1.64 (m, 2H), 1.61 – 1.50 (m, 2H), 1.38 – 1.31 (m, 2H), 1.30 – 1.24 (m, 2H), 1.22 (s, 12H), 0.87 (t, J = 7.2 Hz, 3H). ^{13}C NMR (126 MHz, $CDCl_3$) δ 136.8, 120.8, 83.1, 38.0, 30.1, 28.2, 24.9, 24.8, 24.2, 23.2, 22.5, 14.2. ^{11}B NMR (160 MHz, $CDCl_3$) δ 33.36. HRMS-(DART+) calculated for C16H30BO2 $[M+H]^+$: 265.2333, found: 265.2328.

2-(3-Butylcyclopent-2-en-1-yl)-4,4,5,5-tetramethyl-1,3,2-dioxaborolane (2k)

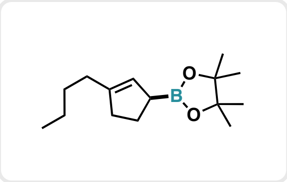

Yield: 74%. Colorless oil. >20:1 r.r.. ^{1}H NMR (500 MHz, $CDCl_3$) δ 5.27 (m, 1H), 2.23 (m, 2H), 2.13 (m, 1H), 2.08 – 1.96 (m, 3H), 1.86 (m, 1H), 1.39 (m, 2H), 1.31 – 1.25 (m, 2H), 1.22 (s, 12H), 0.87 (t, J = 7.3 Hz, 3H). ^{13}C NMR (126 MHz, $CDCl_3$) δ 144.3, 123.5, 83.0, 35.7, 31.0, 30.1, 26.2, 24.9, 24.8, 22.6, 14.1. ^{11}B NMR (160 MHz, $CDCl_3$) δ 33.77. HRMS-(DART+) calculated for C15H28BO2 $[M+H]^+$: 251.2177, found: 251.2170.

2-(3-Butylcyclohept-2-en-1-yl)-4,4,5,5-tetramethyl-1,3,2-dioxaborolane (2l)

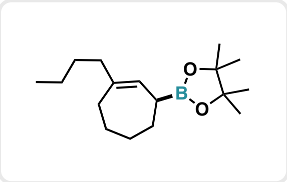

Yield: 77%. Colorless oil. >20:1 r.r.. ^{1}H NMR (500 MHz, $CDCl_3$) δ 5.54 (dq, J = 6.6, 1.2 Hz, 1H), 2.16 – 2.07 (m, 1H), 2.01 (ddd, J = 14.8, 8.5, 2.0 Hz, 1H), 1.98 – 1.92 (m, 2H), 1.87 (dtt, J = 12.6, 6.4, 2.8 Hz, 2H), 1.68 – 1.58 (m, 2H), 1.51 (ddddd, J = 13.9, 8.8, 6.9, 3.5, 1.9 Hz, 1H), 1.46 – 1.39 (m, 1H), 1.38 – 1.24 (m, 5H), 1.23 (s, 12H), 0.87 (t, J = 7.1 Hz, 3H). ^{13}C NMR (126 MHz, $CDCl_3$) δ 144.9, 126.2, 83.1, 40.5, 32.7, 32.6, 30.5, 29.0, 26.9, 24.88, 24.87, 22.6, 14.2. ^{11}B NMR (160 MHz, $CDCl_3$) δ 33.94. HRMS-(DART+) calculated for C17H32BO2 $[M+H]^+$: 279.2490, found: 279.2484.

2-(4,4-Dimethylcyclohex-2-en-1-yl)-4,4,5,5-tetramethyl-1,3,2-dioxaborolane (2m)

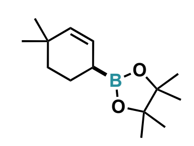

Yield: 57%. Colorless oil. 11:1 r.r.. ^{1}H NMR (500 MHz, $CDCl_3$) δ 5.59 – 5.55 (m, 1H), 5.38 (dd, J = 10.0, 2.0 Hz, 1H), 1.76 – 1.62 (m, 3H), 1.47 – 1.35 (m, 2H), 1.22 (s, 12H), 0.94 (d, J = 2.2 Hz, 6H). ^{13}C NMR (126 MHz, $CDCl_3$) δ 136.6, 125.2, 83.2, 37.5, 31.4, 30.2, 24.9, 24.8, 21.3. ^{11}B NMR (160 MHz, $CDCl_3$) δ 33.44. HRMS-(DART+) calculated for C14H26BO2 $[M+H]^+$: 237.2021, found: 237.2012.

2-((*E*)-2-((2*S*,5*R*)-2-Isopropyl-5-methylcyclohexylidene)ethyl)-4,4,5,5-tetramethyl-1,3,2-dioxa borolane (2n)

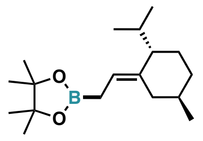

Yield: 42%. Colorless oil. 11:1 r.r.. 11:1 *E*:*Z*. ^{1}H NMR (500 MHz, $CDCl_3$) isomer mixture: δ 5.86 – 5.06 (m, 1H), 2.38 – 2.10 (m, 1H), 1.94 – 1.53 (m, 8H), 1.32 – 1.24 (m, 1H), 1.21 (d, J = 4.4 Hz, 12H), 1.15 – 1.06 (m, 1H), 0.91 – 0.80 (m, 9H). ^{13}C NMR (126 MHz, $CDCl_3$) major isomer: δ 139.5, 115.9, 83.1, 51.4, 34.6, 32.1, 31.7, 26.7, 26.6, 24.9, 24.8, 22.1, 20.4, 20.0. ^{11}B NMR (160 MHz, $CDCl_3$) δ 33.16. HRMS-(DART+) calculated for C18H34BO2 $[M+H]^+$: 293.2646, found: 293.2640.

(*E*)-2-(3,4-Dimethyloct-3-en-2-yl)-4,4,5,5-tetramethyl-1,3,2-dioxaborolane (2o)

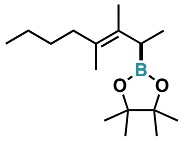

Yield: 57%. Colorless oil. 4:1 r.r.. 2:1 *E*:*Z*. ^{1}H NMR (500 MHz, $CDCl_3$) isomer mixture: δ 2.38 – 2.11 (m, 1H), 2.08 – 1.93 (m, 2H), 1.74 – 1.47 (m, 6H), 1.29 (qdd, J = 11.2, 7.7, 4.9 Hz, 4H), 1.23 – 1.18 (m, 12H), 1.02 (dd, J = 7.5, 2.9 Hz, 3H), 0.88 (t, J = 7.0 Hz, 3H). ^{13}C NMR (126 MHz, $CDCl_3$) major isomer: δ 129.8, 127.7, 82.9, 34.7, 30.6, 24.9, 24.7, 22.7, 18.5, 16.2, 14.32, 14.30. ^{11}B NMR (160 MHz, $CDCl_3$) δ 33.69. HRMS-(DART+) calculated for C16H32BO2 $[M+H]^+$: 267.2490, found: 267.2487.

(*E*)-4,4,5,5-Tetramethyl-2-(3-methylundec-2-en-1-yl)-1,3,2-dioxaborolane (2p)

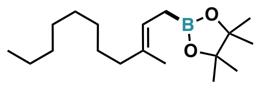

Yield: 70%. Colorless oil. >20:1 r.r.. 2:1 *E*:*Z*. ^{1}H NMR (500 MHz, $CDCl_3$) isomer mixture: δ 5.21 (ddt, J = 7.6, 6.3, 1.4 Hz, 1H), 1.96 (q, J = 7.9 Hz, 2H), 1.68 – 1.65 (m, 1H), 1.59 (t, J = 6.5 Hz, 2H), 1.55 (s, 2H), 1.41 – 1.23 (m, 12H), 1.22 (s, 12H), 0.86 (t, J = 6.8 Hz, 3H). ^{13}C NMR (126 MHz, $CDCl_3$) major isomer: δ 135.6, 118.3, 83.1, 39.9, 32.1, 29.7, 29.5, 29.4, 28.2, 24.9, 22.8, 15.9, 14.2. ^{11}B NMR (160 MHz, $CDCl_3$) δ 33.13. HRMS-(DART+) calculated for C18H36BO2 $[M+H]^+$: 295.2803, found: 295.2797.

(*E*)-4,4,5,5-Tetramethyl-2-(3-methyl-5-phenylpent-2-en-1-yl)-1,3,2-dioxaborolane (2q)

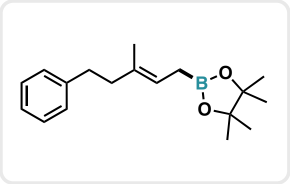

Yield: 68%. Colorless oil. >20:1 r.r.. 2:1 *E*:*Z*. ^{1}H NMR (500 MHz, $CDCl_3$) isomer mixture: δ 7.29 – 7.25 (m, 2H), 7.23 – 7.14 (m, 3H), 5.29 (m, 1H), 2.75 – 2.63 (m, 2H), 2.30 (dt, *J* = 8.7, 6.2 Hz, 2H), 1.73 (d, *J* = 1.4 Hz, 1H), 1.65 (s, 2H), 1.63 – 1.55 (m, 2H), 1.25 (s, 12H). ^{13}C NMR (126 MHz, $CDCl_3$) major isomer: δ 142.8, 134.8, 128.5, 128.3, 125.7, 119.2, 83.2, 41.8, 35.0, 24.9, 16.2. ^{11}B NMR (160 MHz, $CDCl_3$) δ 33.07. HRMS-(DART+) calculated for C18H28BO2 $[M+H]^+$: 287.2177, found: 287.2179.

4,4,5,5-Tetramethyl-2-((2*E*,4*E*)-3-methyl-5-phenylpenta-2,4-dien-1-yl)-1,3,2-dioxaborolane (2r)

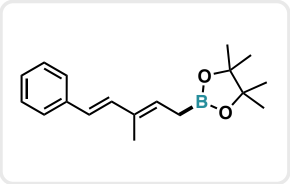

Yield: 30%. Colorless oil. >20:1 r.r.. 11:1 *E*:*Z*. ^{1}H NMR (500 MHz, $CDCl_3$) δ 7.38 – 7.34 (m, 2H), 7.28 – 7.24 (m, 2H), 7.16 – 7.12 (m, 1H), 6.82 (d, *J* = 16.1 Hz, 1H), 6.38 (d, *J* = 16.1 Hz, 1H), 5.76 (t, *J* = 7.7 Hz, 1H), 1.85 – 1.79 (m, 5H), 1.23 (s, 12H). ^{13}C NMR (126 MHz, $CDCl_3$) δ 138.3, 134.4, 134.1, 129.1, 128.6, 126.8, 126.2, 124.8, 83.5, 24.9, 12.4. ^{11}B NMR (160 MHz, $CDCl_3$) δ 32.88. HRMS-(DART+) calculated for C18H26BO2 $[M+H]^+$: 285.2020, found: 285.2016.

(*E*)-2-(3,7-Dimethylocta-2,6-dien-1-yl)-4,4,5,5-tetramethyl-1,3,2-dioxaborolane (2s)

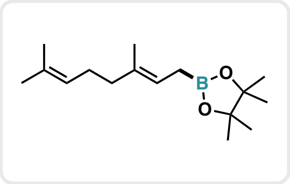

Yield: 95%. Colorless oil. >20:1 r.r.. 3:1 *E*:*Z*. ^{1}H NMR (500 MHz, $CDCl_3$) isomer mixture: δ 5.23 (td, *J* = 6.1, 3.1 Hz, 1H), 5.14 – 5.05 (m, 1H), 2.08 – 1.94 (m, 4H), 1.66 (d, *J* = 9.1 Hz, 4H), 1.58 (t, *J* = 5.2 Hz, 7H), 1.22 (d, *J* = 4.0 Hz, 12H). ^{13}C NMR (101 MHz, $CDCl_3$) major isomer: δ 135.2, 131.2, 124.6, 118.7, 83.2, 39.9, 31.9, 26.9, 25.8, 24.9, 17.8, 16.0. ^{11}B NMR (160 MHz, $CDCl_3$) δ 33.17. HRMS-(DART+) calculated for C16H30BO2 $[M+H]^+$: 265.2334, found: 265.2330.

2-(((1*R*,5*S*)-6,6-Dimethylbicyclo[3.1.1]hept-2-en-2-yl)methyl)-4,4,5,5-tetramethyl-1,3,2-dioxaborolane (2t)

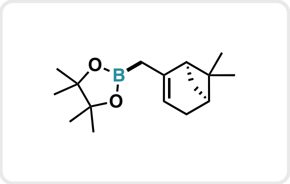

Yield: 93%. Colorless oil. >20:1 r.r.. ^{1}H NMR (500 MHz, $CDCl_3$) δ 5.18 (tq, *J* = 3.0, 1.5 Hz, 1H), 2.32 (dt, *J* = 8.4, 5.5 Hz, 1H), 2.26 – 2.13 (m, 2H), 2.05 (ttd, *J* = 5.8, 2.9, 1.3 Hz, 1H), 1.99 (td, *J* = 5.6, 1.6 Hz, 1H), 1.65 (dtt, *J* = 16.5, 14.7, 1.8 Hz, 2H), 1.26 (d, *J* = 3.6 Hz, 4H), 1.23 (s, 12H), 0.86 (s, 3H). ^{13}C NMR (126 MHz, $CDCl_3$) δ 144.7, 115.8, 83.3, 47.5, 40.8, 38.1, 31.8, 31.5, 26.6, 25.0, 24.9, 21.3. ^{11}B NMR (160 MHz, $CDCl_3$) δ 32.88. HRMS-(DART+) calculated for C18H28BO2 $[M+H]^+$: 287.2177, found: 287.2175.

(1*R*,2*R*,4a*S*,8a*S*)-2,5,5,8a-Tetramethyl-1-((*E*)-3-methyl-5-(4,4,5,5-tetramethyl-1,3,2-dioxaborolan-2-yl)pent-3-en-1-yl)decahydronaphthalen-2-ol (2u)

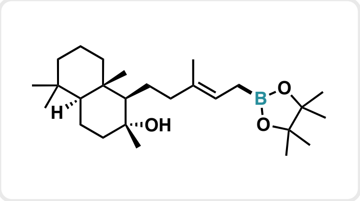

General Procedure *step 1* using HBpin (2.5 equiv) at 60 ºC for 2 h. Yield: 82%. Colorless oil. >20:1 r.r.. 3:1 *E*:*Z*. ^{1}H NMR (400 MHz, $CDCl_3$) isomer mixture: δ 5.23 – 5.08 (m, 1H), 2.05 (dt, *J* = 12.8, 6.6 Hz, 1H), 1.93 (tt, *J* = 11.9, 5.5 Hz, 1H), 1.80 (dt, *J* = 11.6, 3.0 Hz, 1H), 1.67 – 1.55 (m, 5H), 1.53 (s, 3H), 1.51 – 1.47 (m, 1H), 1.43 – 1.25 (m, 5H), 1.20 (s, 12H), 1.18 – 1.13 (m, 1H), 1.10 (d, *J* = 6.9 Hz, 1H), 1.02 (s, 3H), 0.96 – 0.86 (m, 2H), 0.82 (s, 3H), 0.75 (d, *J* = 6.0 Hz, 6H). ^{13}C NMR (101 MHz, $CDCl_3$) major isomer: δ 135.7, 119.6, 83.3, 77.4, 73.3, 60.1, 56.3, 44.9, 42.4, 42.1, 40.0, 39.5, 33.5, 33.3, 24.8, 24.7, 23.4, 22.9, 21.6, 20.8, 18.5, 15.9, 15.6. ^{11}B NMR (160 MHz, $CDCl_3$) δ 34.02. HRMS-(DART+) calculated for C26H48BO3 $[M+H]^+$: 419.3691, found: 419.3688.

4,4,5,5-Tetramethyl-2-((1*S*,5*S*)-2-methyl-5-(prop-1-en-2-yl)cyclohex-2-en-1-yl)-1,3,2-dioxaborolane (2v)

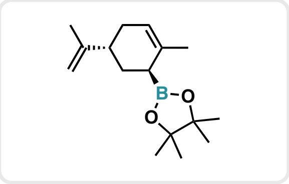

General Procedure *step 2* using ^{i}PrO–Bpin (3.0 equiv). Yield: 85%. Colorless oil. 4:1 d.r.. ^{1}H NMR (400 MHz, $CDCl_3$) isomer mixture: δ 5.54 – 5.35 (m, 1H), 4.70 (t, *J* = 1.3 Hz, 2H), 2.10 – 1.82 (m, 5H), 1.75 – 1.68 (m, 6H), 1.26 (d, *J* = 2.1 Hz, 12H), 1.22 (d, *J* = 2.7 Hz, 1H). ^{13}C NMR (101 MHz, $CDCl_3$) major isomer: δ 150.5, 133.6, 121.2, 108.5, 83.4, 41.5, 30.8, 30.4, 24.9, 24.8, 24.1, 23.5, 21.2. ^{11}B NMR (160 MHz, $CDCl_3$) δ 33.82. HRMS-(DART+) calculated for C16H28BO2 $[M+H]^+$: 263.2177, found: 263.2175.

4,4,5,5-Tetramethyl-2-((1*R*,6*R*)-3-methyl-6-(prop-1-en-2-yl)cyclohex-2-en-1-yl)-1,3,2-dioxaborolane (2w)

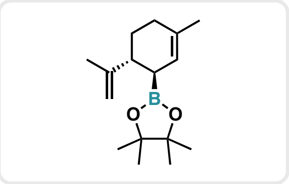

Yield: 95%. Colorless oil. >20:1 r.r.. >20:1 d.r.. ^{1}H NMR (500 MHz, $CDCl_3$) δ 5.28 (tt, *J* = 2.6, 1.2 Hz, 1H), 4.73 – 4.67 (m, 2H), 2.27 (td, *J* = 10.8, 2.8 Hz, 1H), 2.10 – 2.01 (m, 1H), 1.93 – 1.82 (m, 2H), 1.76 – 1.72 (m, 1H), 1.71 (s, 3H), 1.63 (dd, *J* = 2.4, 1.2 Hz, 3H), 1.47 – 1.40 (m, 1H), 1.21 (d, *J* = 3.9 Hz, 12H). ^{13}C NMR (126 MHz, $CDCl_3$) δ 150.1, 133.1, 120.2, 109.6, 83.2, 42.9, 30.4, 28.7, 24.9, 24.7, 24.1, 20.4. ^{11}B NMR (160 MHz, $CDCl_3$) δ 33.38. HRMS-(DART+) calculated for C16H28BO2 [M+H]+: 263.2177, found: 263.2173..

2-((2*R*,4*R*,4a*S*,6*R*)-4,4a-Dimethyl-6-(prop-1-en-2-yl)-2,3,4,4a,5,6,7,8-octahydronaphthalen-2-yl)-4,4,5,5-tetramethyl-1,3,2-dioxaborolane (2x)

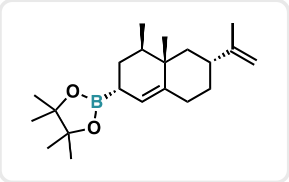

Yield: 80%. Colorless oil. >20:1 r.r.. 1.4:1 d.r.. ^{1}H NMR (400 MHz, $CDCl_3$) isomer mixture: δ 5.40 – 5.33 (m, 1H), 4.66 (s, 2H), 2.33 – 2.17 (m, 2H), 2.10 (dtd, *J* = 13.9, 4.5, 2.6 Hz, 1H), 1.89 – 1.72 (m, 3H), 1.70 (d, *J* = 3.0 Hz, 3H), 1.55 – 1.38 (m, 3H), 1.24 – 1.20 (m, 12H), 1.17 (dd, *J* = 7.3, 3.9 Hz, 2H), 0.93 (s, 3H), 0.86 (d, *J* = 6.9 Hz, 3H). ^{13}C NMR (101 MHz, $CDCl_3$) major isomer: δ 150.9, 142.1, 120.3, 108.4, 83.1, 45.1, 41.9, 41.1, 40.4, 37.7, 33.4, 33.1, 28.9, 24.9, 24.8, 20.9, 18.8, 15.6. ^{11}B NMR (160 MHz, $CDCl_3$) δ 34.13. HRMS-(DART+) calculated for C21H36BO2 [M+H]+: 331.2803, found: 331.2803.

4,4,5,5-Tetramethyl-2-((1*R*,2*R*,5*S*)-4,6,6-trimethylbicyclo[3.1.1]hept-3-en-2-yl)-1,3,2-dioxaborolane (2y)

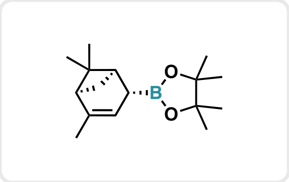

Yield: 94%. Colorless oil. >20:1 r.r.. >20:1 d.r.. ^{1}H NMR (500 MHz, $CDCl_3$) δ 5.25 (dq, *J* = 3.1, 1.6 Hz, 1H), 2.32 (dt, *J* = 8.6, 5.6 Hz, 1H), 2.16 (tt, *J* = 6.0, 1.9 Hz, 1H), 1.98 (q, *J* = 2.6 Hz, 1H), 1.92 (td, *J* = 5.6, 1.5 Hz, 1H), 1.68 – 1.64 (m, 3H), 1.25 (s, 3H), 1.23 (d, *J* = 4.2 Hz, 12H), 1.10 (d, *J* = 8.6 Hz, 1H), 0.82 (s, 3H). ^{13}C NMR (101 MHz, $CDCl_3$) δ 143.5, 116.6, 83.2, 47.1, 42.5, 37.9, 30.4, 26.3, 24.9, 24.8, 23.5, 20.9. ^{11}B NMR (160 MHz, $CDCl_3$) δ 33.55. HRMS-(DART+) calculated for C16H28BO2 $[M+H]^+$: 263.2177, found: 263.2182.

1-Benzyl-4-(((1*R*,2*S*)-5,6-dimethoxy-1-(4,4,5,5-tetramethyl-1,3,2-dioxaborolan-2-yl)-2,3-dihydro-1*H*-inden-2-yl)methyl)piperidine (2z)

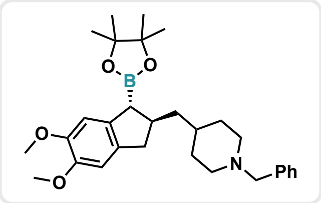

Yield: 82%. Colorless oil. 4:1 d.r.. ^{1}H NMR (400 MHz, $CDCl_3$) isomer mixture: δ 7.33 – 7.28 (m, 4H), 7.26 – 7.20 (m, 1H), 6.73 (d, *J* = 16.9 Hz, 2H), 3.82 (s, 6H), 3.49 (s, 2H), 3.04 – 2.81 (m, 3H), 2.76 – 2.46 (m, 2H), 2.37 – 2.07 (m, 1H), 1.95 (tt, *J* = 8.8, 3.7 Hz, 2H), 1.78 – 1.58 (m, 2H), 1.46 (tt, *J* = 13.8, 8.6 Hz, 2H), 1.36 – 1.28 (m, 2H), 1.26 (s, 3H), 1.25 (s, 3H), 1.23 (s, 4H), 1.18 (dd, *J* = 7.5, 4.3 Hz, 3H). ^{13}C NMR (101 MHz, $CDCl_3$) major isomer: δ 147.8, 147.6, 138.7, 136.3, 135.5, 129.3, 128.2, 126.9, 108.0, 107.9, 83.4, 75.1, 63.7, 56.13, 56.10, 54.1, 43.8, 40.4, 40.2, 34.5, 32.9, 32.5, 25.1, 24.9, 24.7. ^{11}B NMR (160 MHz, $CDCl_3$) δ 22.20. HRMS-(DART+) calculated for C30H43BNO4 $[M+H]^+$: 492.3279, found: 492.3277.

4,4,5,5-Tetramethyl-2-((*E*)-2-methyl-3-((1*R*,4*R*)-1-methyl-4-(prop-1-en-2-yl)cyclohex-2-en-1-yl)allyl)-1,3,2-dioxaborolane (2ad)

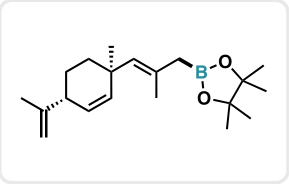

Yield: 60%. Colorless oil. 1.2:1 *E*:*Z*. ^{1}H NMR (400 MHz, $CDCl_3$) isomer mixture: δ 5.67 (ddd, *J* = 9.2, 6.4, 2.4 Hz, 1H), 5.45 (dd, *J* = 10.1, 2.5 Hz, 1H), 5.15 (d, *J* = 8.1 Hz, 1H), 4.73 (d, *J* = 6.0 Hz, 2H), 2.71 (ddt, *J* = 8.6, 5.7, 2.7 Hz, 1H), 1.92 – 1.74 (m, 2H), 1.73 (d, *J* = 7.9 Hz, 6H), 1.69 – 1.64 (m, 1H), 1.61 – 1.46 (m, 3H), 1.24 (s, 12H), 1.09 (s, 3H). ^{13}C NMR (101 MHz, $CDCl_3$) major isomer: δ 149.5, 137.5, 133.1, 132.9, 127.6, 110.1, 83.3, 43.4, 36.4, 35.4, 29.8, 27.6, 25.4, 24.85, 24.83, 20.8, 19.1. ^{11}B NMR (160 MHz, $CDCl_3$) δ 33.64. HRMS-(DART+) calculated for C20H34BO2 $[M+H]^+$: 317.2647, found: 317.2647.

5.2. General Procedure for E-Borylation of Acrylates

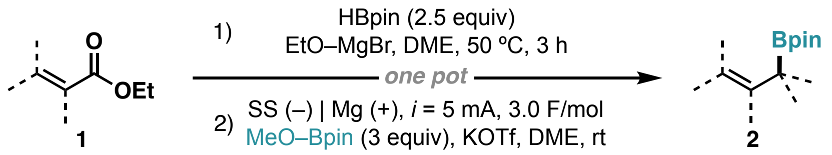

Step 1 –Pre-activation of Acrylate: In a nitrogen-filled glovebox, an 8 mL vial equipped with a magnetic stir bar was charged with compound **1** (0.5 mmol, 1.0 equiv), 0.05 mL of EtO–MgBr (0.5 M in THF, 0.05 equiv), and 0.5 mL of DME. To this suspension, HBpin (1.25 mmol, 2.5 equiv) was added dropwise via syringe. The reaction mixture was stirred at 50 ºC for 3 h.

Step 2 – Electrochemical Borylation: To a 5 mL ElectraSyn vial, KOTf (1.0 mmol, 2.0 equiv) and MeO–Bpin (1.5 mmol, 3.0 equiv) were added. The reaction mixture from *Step 1* was transferred into the ElectraSyn vial using 3.5 mL of DME. The vial was sealed with a preassembled cap equipped with a magnesium anode and a stainless steel cathode, removed from the glovebox, and maintained under a nitrogen atmosphere (balloon). Electrolysis was carried out at room temperature under a constant current of 5 mA until a total charge of 3 F/mol was passed (~8 h).

Workup: Upon completion, the reaction was quenched by the addition of 30 μL of water (~3 drops) and stirred for 5 min. ***(Caution**: failure to quench the reaction may cause fire upon exposure of the magnesium electrode to air.)* The mixture was then diluted with 20 mL of hexanes and passed through a short pad of Celite to remove the electrolyte. The filtrate was concentrated under reduced pressure, and the crude residue was further purified by flash chromatography (EtOAc:hexanes = 1:20) to afford the borylated product **2**. ***(Note:** purification should be performed promptly to avoid product decomposition on silica.)*

2-Cinnamyl-4,4,5,5-tetramethyl-1,3,2-dioxaborolane (2aa)

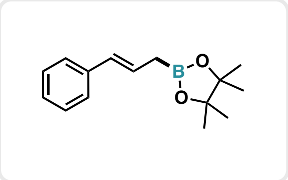

Yield: 61%. Colorless oil. >20:1 r.r.. >20:1 *E*:*Z*. ^{1}H NMR (400 MHz, $CDCl_3$) δ 7.25 (d, *J* = 8.7 Hz, 2H), 7.19 (t, *J* = 7.0 Hz, 2H), 7.08 (t, *J* = 7.3 Hz, 1H), 6.37 – 6.16 (m, 2H), 1.80 (d, *J* = 7.1 Hz, 2H), 1.18 (s, 12H). ^{13}C NMR (101 MHz, $CDCl_3$) δ 138.3, 130.4, 128.5, 126.6, 126.4, 125.9, 83.5, 24.9. ^{11}B NMR (160 MHz, $CDCl_3$) δ 33.21. HRMS-(DART+) calculated for C15H22BO2 $[M+H]^+$: 245.1707, found: 245.1707.

(*E*)-4,4,5,5-Tetramethyl-2-(5-phenylpent-2-en-1-yl)-1,3,2-dioxaborolane (2ab)

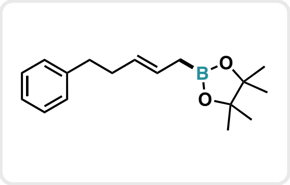

Yield: 76%. Colorless oil. >20:1 r.r.. >20:1 *E*:*Z*. ^{1}H NMR (400 MHz, $CDCl_3$) δ 7.23 (d, *J* = 6.9 Hz, 2H), 7.19 – 7.11 (m, 3H), 5.55 – 5.37 (m, 2H), 2.68 – 2.60 (m, 2H), 2.34 – 2.24 (m, 2H), 1.63 (d, *J* = 6.6 Hz, 2H), 1.22 (s, 12H). ^{13}C NMR (101 MHz, $CDCl_3$) δ 142.4, 130.1, 128.6, 128.3, 125.7, 125.6, 83.3, 36.3, 34.6, 24.9. ^{11}B NMR (160 MHz, $CDCl_3$) δ 33.42. HRMS-(DART+) calculated for C17H26BO2 $[M+H]^+$: 273.2021, found: 273.2019.

(*E*)-2-(3-Cyclohexylallyl)-4,4,5,5-tetramethyl-1,3,2-dioxaborolane (2ac)

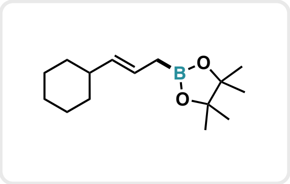

Yield: 78%. Colorless oil. >20:1 r.r.. >20:1 *E*:*Z*. ^{1}H NMR (400 MHz, $CDCl_3$) δ 5.45 – 5.26 (m, 2H), 1.87 (tdd, *J* = 10.8, 6.3, 3.1 Hz, 1H), 1.71 – 1.54 (m, 7H), 1.28 – 1.23 (m, 1H), 1.22 (s, 12H), 1.18 (d, *J* = 2.4 Hz, 1H), 1.17 – 1.08 (m, 1H), 1.01 (qd, *J* = 12.1, 2.9 Hz, 2H). ^{13}C NMR (101 MHz, $CDCl_3$) δ 137.2, 122.3, 83.2, 40.9, 33.4, 26.4, 26.3, 24.9. ^{11}B NMR (160 MHz, $CDCl_3$) δ 33.08. HRMS-(DART+) calculated for C15H28BO2 $[M+H]^+$: 251.2177, found: 251.2175.

5.3. General Procedures for Gram-Scale E-Borylation

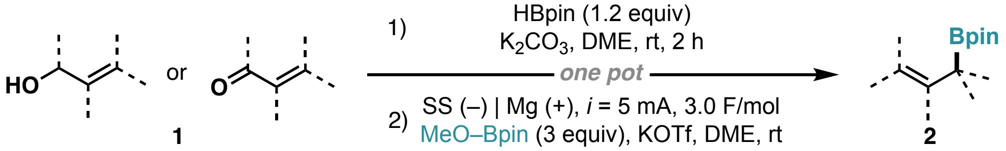

• 5 mmol Scale •

Step 1 –Pre-activation of Alcohol/Enal/Enone: In a nitrogen-filled glovebox, a 20 mL vial equipped with a magnetic stir bar was charged with compound **1** (5.0 mmol, 1.0 equiv), K_2CO_3 (0.25 mmol, 0.05 equiv), and 5.0 mL of DME. To this suspension, HBpin (6.0 mmol, 1.2 equiv) was added dropwise via syringe. *(**Caution**: hydrogen gas evolution was observed upon addition.)* The reaction mixture was stirred at room temperature for 4 h in the glovebox.

Step 2 – Electrochemical Borylation: To a 20 mL ElectraSyn vial, KOTf (10.0 mmol, 2.0 equiv) and MeO–Bpin (15.0 mmol, 3.0 equiv) were added. The reaction mixture from *Step 1* was transferred into the ElectraSyn vial using 10 mL of DME. The vial was sealed with a preassembled cap equipped with a magnesium anode and a stainless steel cathode, removed from the glovebox, and maintained under a nitrogen atmosphere (balloon). Electrolysis was carried out at room temperature under a constant current of 5 mA until a total charge of 3 F/mol was passed.

Workup: Upon completion, the reaction was quenched by the addition of 300 μL of water and stirred for 5 min. *(**Caution**: failure to quench the reaction may cause fire upon exposure of the magnesium electrode to air.)* The mixture was then diluted with 200 mL of hexanes and passed through a short pad of Celite to remove the electrolyte. The filtrate was then washed sequentially with saturated NH_4Cl (2 x 25 mL), saturated $NaHCO_3$ (2 x 25 mL), water (2 x 25 mL), and brine (1 x 25 mL). The organic phase was dried over Na_2SO_4, filtered, and concentrated under reduced pressure to obtain the product **2**.

• 50 mmol Scale •

Step 1 –Pre-activation of Alcohol/Enal/Enone: In a nitrogen-filled glovebox, a 100 mL flask equipped with a magnetic stir bar was charged with compound **1** (50.0 mmol, 1.0 equiv), K_2CO_3 (2.5 mmol, 0.05 equiv), and 50.0 mL of DME. To this suspension, HBpin (60.0 mmol, 1.2 equiv) was added dropwise via syringe. *(**Caution**: hydrogen gas evolution was observed upon addition.)* The reaction mixture was stirred at room temperature for 4 h in the glovebox.

Step 2 – Electrochemical Borylation: To a 250 mL flask, KOTf (100.0 mmol, 2.0 equiv) and MeO–Bpin (150.0 mmol, 3.0 equiv) were added. The reaction mixture from *Step 1* was transferred into the flask using 100 mL of DME. The vial was sealed with a preassembled cap equipped with

a magnesium anode and a stainless steel cathode, removed from the glovebox, and maintained under a nitrogen atmosphere (balloon). Electrolysis was carried out at room temperature under a constant current of 30 mA until a total charge of 3 F/mol was passed.

Workup: Upon completion, the reaction was quenched by the addition of 5 mL of water and stirred for 5 min. *(**Caution**: failure to quench the reaction may cause fire upon exposure of the magnesium electrode to air.)* The mixture was then diluted with 600 mL of hexanes and passed through a short pad of Celite to remove the electrolyte. The filtrate was then washed sequentially with saturated NH_4Cl (2 x 100 mL), saturated $NaHCO_3$ (2 x 100 mL), water (2 x 100 mL), and brine (1 x 100 mL). The organic phase was dried over Na_2SO_4, filtered, and concentrated under reduced pressure to obtain the product **2**.

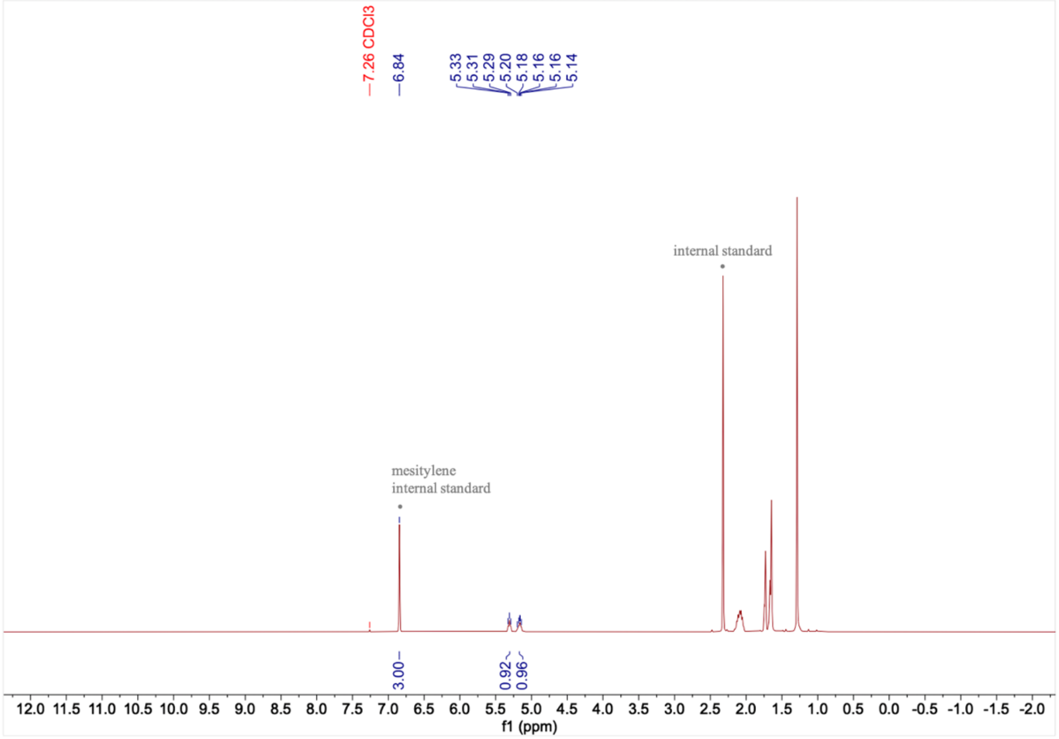

Figure S2. ^{1}H NMR spectrum of the crude mixture of **2s** with mesitylene as an internal standard.

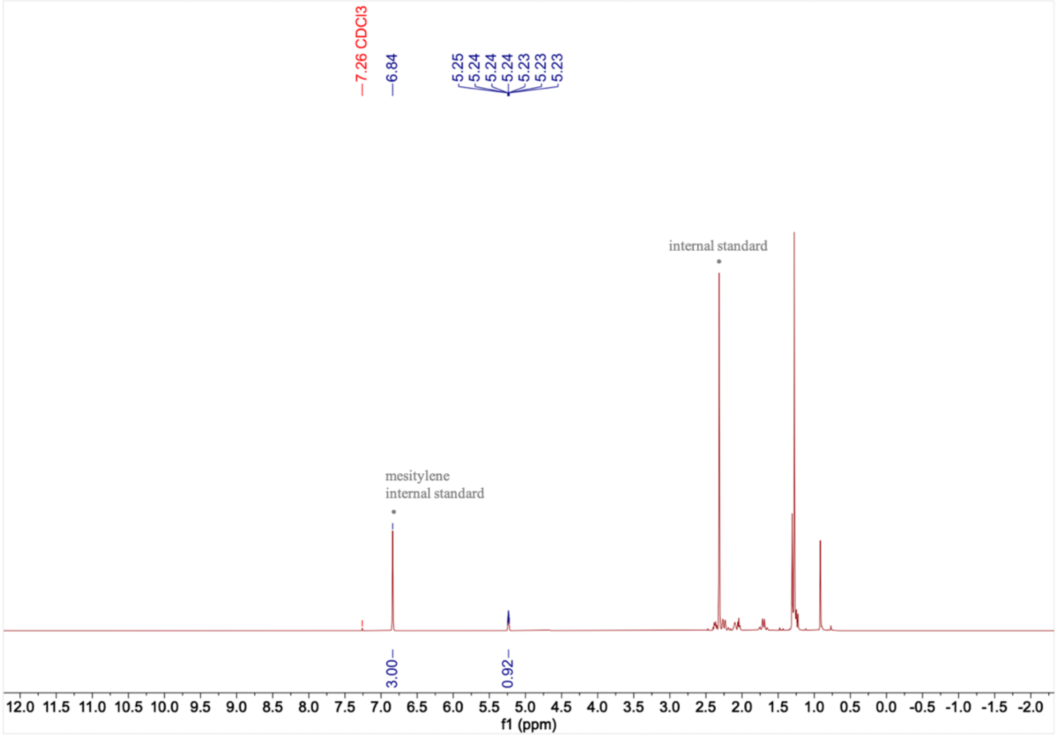

Figure S3. ^{1}H NMR spectrum of the crude mixture of **2t** with mesitylene as an internal standard.

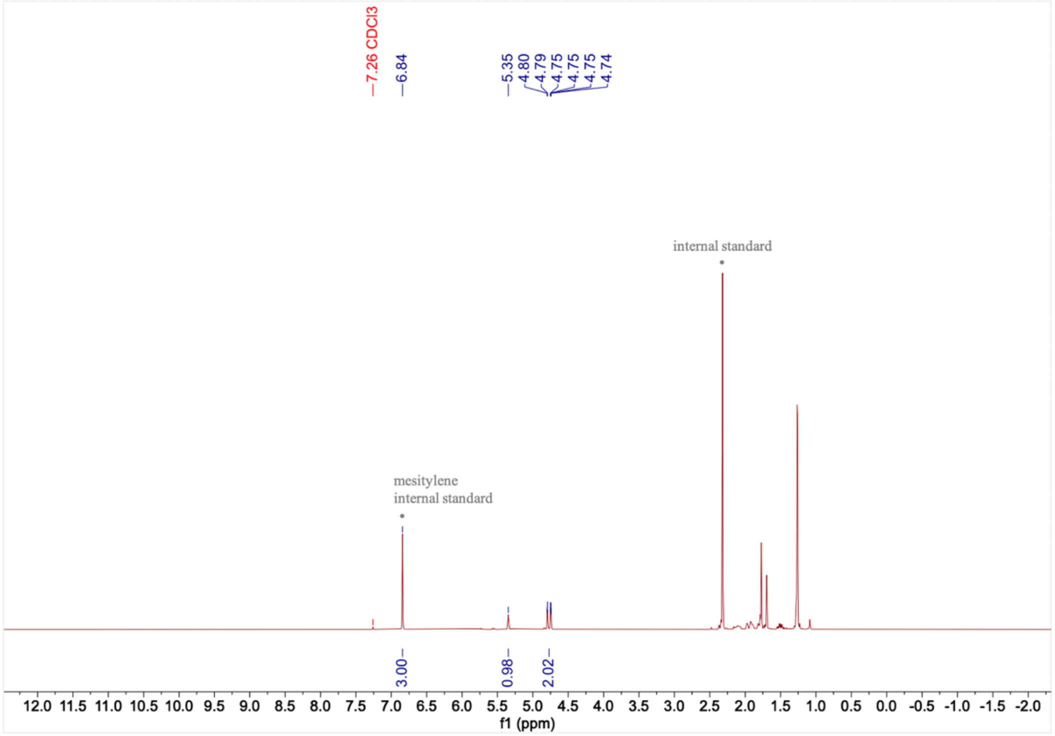

Figure S4. ^{1}H NMR spectrum of the crude mixture of **2w** with mesitylene as an internal standard.

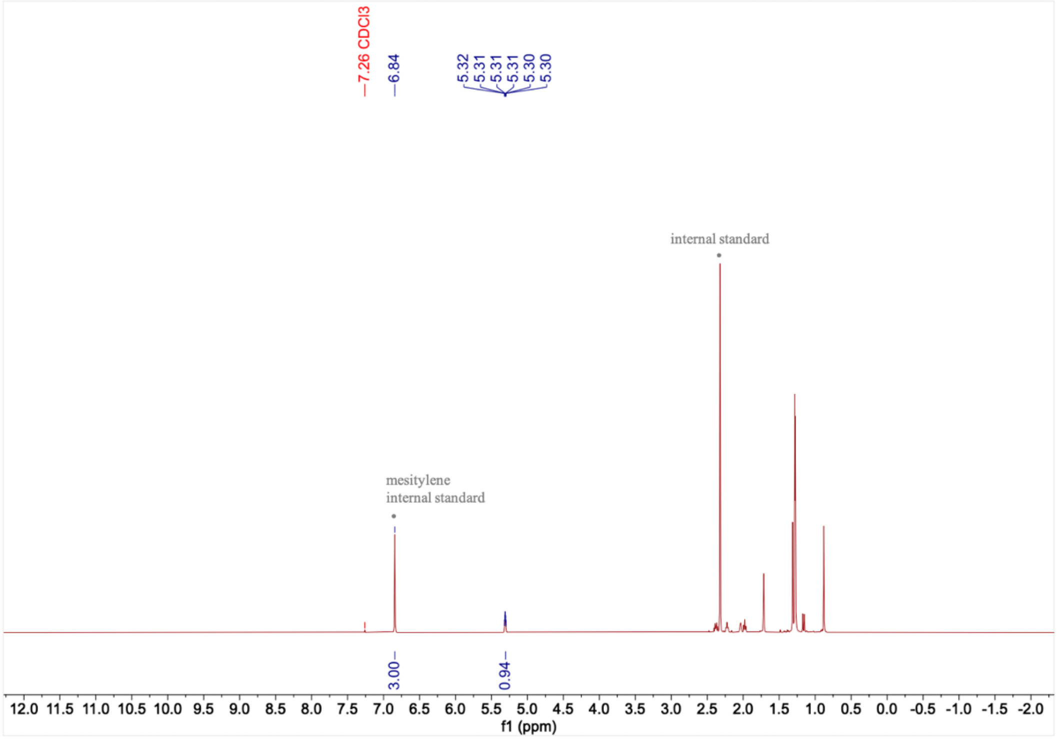

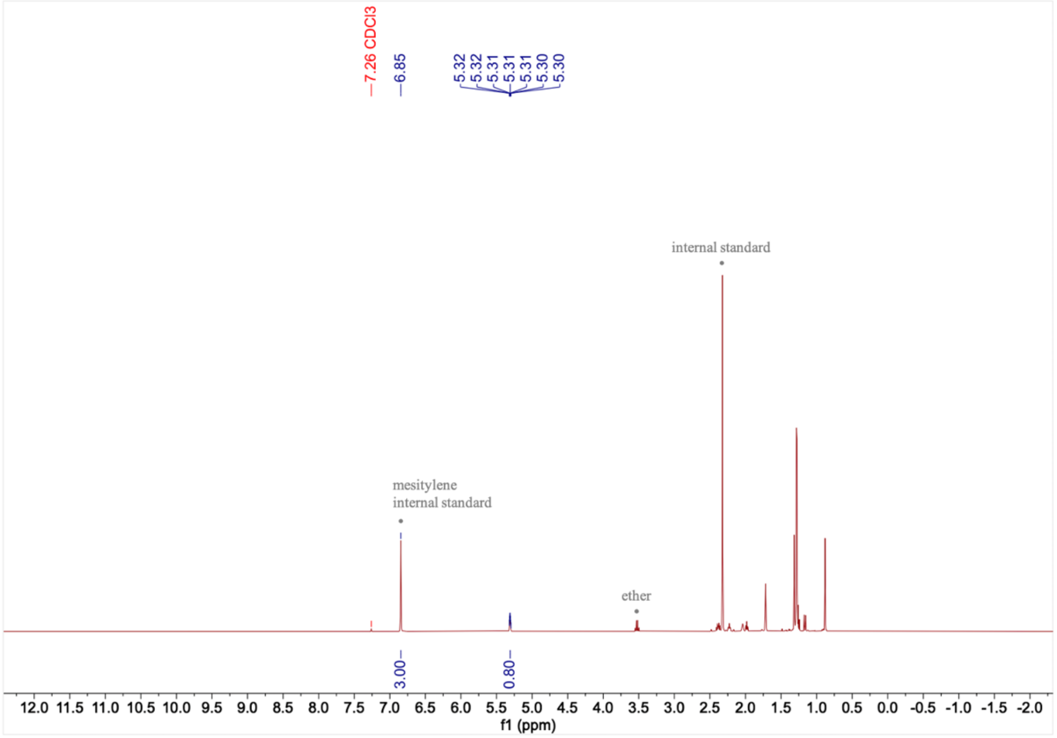

Figure S5. ^{1}H NMR spectrum of the crude mixture of **2y** with mesitylene as an internal standard (5 mmol and 50 mmol scale, respectively).

6. Synthetic Applications of Electrochemical Deoxygenative Borylation

6.1. General Procedures for Alcohol Transposition

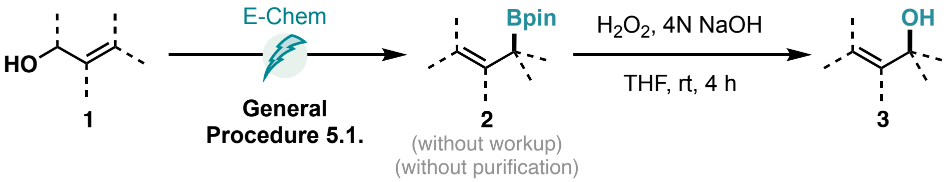

General Procedure: The borylated intermediate **2** was obtained from 0.5 mmol of **1** following *General Procedure 5.1.*, Steps 1 and 2, **without the workup step**. After electrolysis, the crude mixture containing **2** was transferred to a 20 mL vial using 4 mL of THF. The reaction mixture was cooled to 0 ºC in an ice-water bath, followed by the sequential addition of 1 mL of 4 N aqueous NaOH and 1 mL of 30% aqueous H_2O_2. The reaction was stirred at room temperature for 4 h. It was then cooled again to 0 ºC and quenched by slow addition of 3 mL of saturated aqueous Na_2SO_3. The mixture was extracted with diethyl ether (3 x 20 mL), and the combined organic layers were dried over Na_2SO_4. The filtrate was concentrated under reduced pressure, and the crude residue was purified by flash chromatography (EtOAc:hexanes = 1:10) to afford product **3**.

(1*R*,2*R*,4a*S*,8a*S*)-1-((*E*)-5-Hydroxy-3-methylpent-3-en-1-yl)-2,5,5,8a-tetramethyldecahydronaphthalen-2-ol (3a)

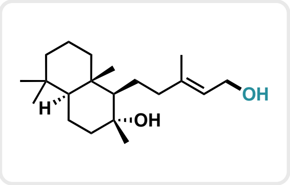

Yield: 68%. White solid. >20:1 r.r.. 3:1 *E*:*Z*. ^{1}H NMR (400 MHz, $CDCl_3$) isomer mixture: δ 5.47 – 5.37 (m, 1H), 4.18 – 3.96 (m, 2H), 2.30 – 2.01 (m, 2H), 1.83 (dt, *J* = 12.2, 3.1 Hz, 1H), 1.76 – 1.47 (m, 7H), 1.38 (dddd, *J* = 16.5, 11.7, 5.1, 2.6 Hz, 4H), 1.29 – 1.20 (m, 2H), 1.19 – 1.12 (m, 1H), 1.11 (s, 3H), 1.04 (t, *J* = 4.0 Hz, 1H), 0.98 – 0.88 (m, 2H), 0.85 (s, 3H), 0.77 (d, *J* = 3.3 Hz, 6H). ^{13}C NMR (101 MHz, $CDCl_3$) major isomer: δ 140.7, 123.4, 74.2, 61.2, 59.3, 56.2, 44.6, 42.9, 42.1, 39.8, 39.3, 33.5, 33.3, 24.0, 23.6, 21.6, 20.7, 18.5, 16.5, 15.6. HRMS-(DART+) calculated for C20H37O2 $[M+H]^+$: 309.2788, found: 309.2791.

(1*R*,6*S*)-3-Methyl-6-(prop-1-en-2-yl)cyclohex-2-en-1-ol (3b)

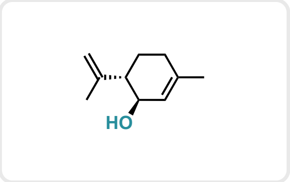

Yield: 85%. Colorless oil. >20:1 r.r.. >20:1 d.r.. ^{1}H NMR (400 MHz, $CDCl_3$) δ 5.44 (s, 1H), 4.86 (d, *J* = 15.0 Hz, 2H), 4.11 (d, *J* = 8.9 Hz, 1H), 2.07 (ddd, *J* = 12.3, 8.9, 3.2 Hz, 2H), 1.92 (dd, *J* = 17.7, 5.5 Hz, 1H), 1.83 – 1.76 (m, 1H), 1.73 (s, 3H), 1.69 (s, 3H), 1.60 (tt, *J* = 12.7, 6.4 Hz, 1H). ^{13}C NMR (101 MHz, $CDCl_3$) δ 146.6, 136.8, 124.5, 112.5, 68.8, 51.1, 30.4, 26.3, 23.2, 19.5. HRMS-(DART+) calculated for C10H17O $[M+H]^+$: 153.1274, found: 153.1273.

(2*E*,6*E*,10*E*)-3,7,11,15-Tetramethylhexadeca-2,6,10,14-tetraen-1-ol (3c)

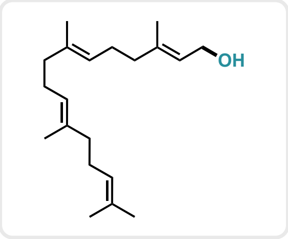

Yield: 69%. Colorless oil. >20:1 r.r.. 2:1 *E*:*Z*. ^{1}H NMR (400 MHz, $CDCl_3$) isomer mixture: δ 5.42 (q, *J* = 7.5 Hz, 1H), 5.10 (q, *J* = 6.7 Hz, 3H), 4.17 – 4.05 (m, 2H), 2.16 – 2.02 (m, 8H), 1.98 (q, *J* = 7.0 Hz, 4H), 1.75 (s, 1H), 1.68 (s, 5H), 1.60 (s, 9H). ^{13}C NMR (101 MHz, $CDCl_3$) major isomer: δ 139.9, 135.5, 135.1, 131.4, 124.5, 124.3, 123.9, 123.5, 59.5, 39.84, 39.81, 39.7, 26.9, 26.8, 26.5, 25.8, 17.8, 16.4, 16.1. HRMS-(DART+) calculated for C20H35O $[M+H]^+$: 291.2682, found: 291.2682.

6.2. General Procedures for Carbonyl Transposition

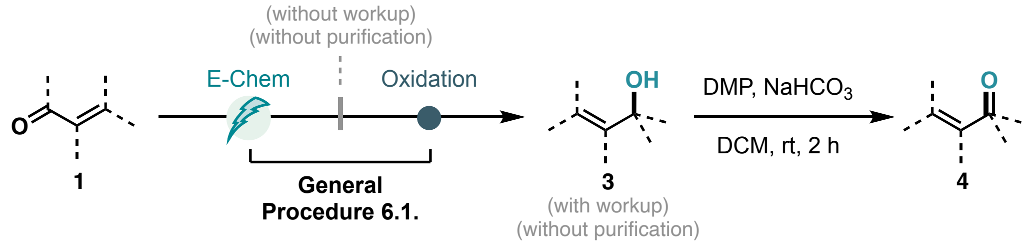

General Procedure: The alcohol intermediate **3** was obtained from 0.5 mmol of **1** by following *General Procedure 6.1.*, **including the aqueous workup step but without chromatographic purification of compound 3**. After the workup, the concentrated crude mixture containing **3** was transferred to a 20 mL vial using 4 mL of DCM. The reaction mixture was cooled to 0 ºC in an ice-water bath, followed by the sequential addition of $NaHCO_3$ (20 mg) and Dess–Martin periodinane (DMP, 1.5 equiv). The reaction was stirred at room temperature for 2 h. Upon completion, the reaction was quenched by the addition of saturated aqueous $NaHCO_3$. The mixture was extracted with DCM (3 x 20 mL) and brine (1 x 20 mL). The combined organic layers were dried over Na_2SO_4, filtered, and concentrated under reduced pressure. The crude residue was purified by flash chromatography (EtOAc:hexanes = 1:20) to afford product **4**.

(*E*)-5-Phenylpent-2-enal (4a)

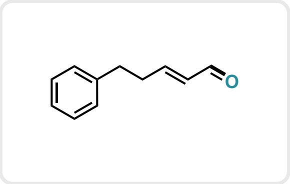

Yield: 68%. Yellow oil. >20:1 r.r.. 5.7:1 *E*:*Z*. ^{1}H NMR (400 MHz, $CDCl_3$) isomer mixture: δ 9.90 – 9.36 (m, 1H), 7.25 – 7.08 (m, 5H), 6.84 – 6.50 (m, 1H), 6.11 – 5.84 (m, 1H), 2.88 – 2.58 (m, 4H). ^{13}C NMR (101 MHz, $CDCl_3$) major isomer: δ 194.0, 157.4, 140.4, 133.5, 128.7, 128.4, 126.5, 34.3, 34.2. HRMS-(DART+) calculated for C11H13O $[M+H]^+$: 161.0961, found: 161.0961.

(*E*)-3-((1*r*,3*r*,5*r*,7*r*)-Adamantan-2-yl)acrylaldehyde (4b)

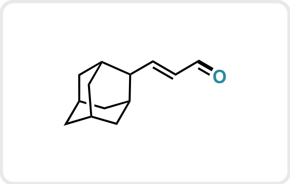

Yield: 40%. White solid. >20:1 r.r.. >20:1 *E*:*Z*. ^{1}H NMR (400 MHz, $CDCl_3$) δ 9.50 (d, *J* = 7.8 Hz, 1H), 6.63 (d, *J* = 15.8 Hz, 1H), 5.98 (dd, *J* = 15.8, 7.8 Hz, 1H), 2.04 (d, *J* = 5.3 Hz, 3H), 1.80 – 1.69 (m, 5H), 1.67 (s, 7H). ^{13}C NMR (101 MHz, $CDCl_3$) δ 195.1, 168.5, 128.3, 41.1, 36.63, 36.60, 28.1. HRMS-(DART+) calculated for C13H19O $[M+H]^+$: 191.1430, found: 191.1429.

6.3. General Procedures for C–C Coupling

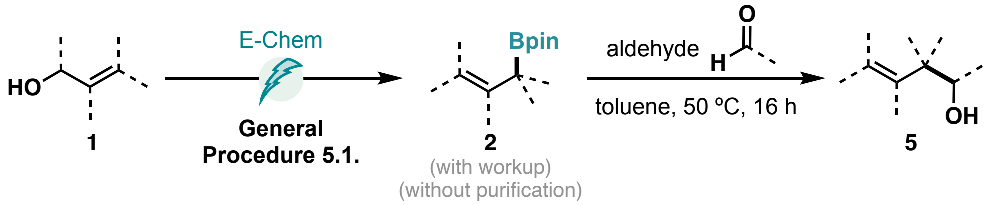

General Procedure: The borylated intermediate **2** was obtained from 0.5 mmol of **1** following *General Procedure 5.1.*, Steps 1 and 2, **including the workup step but without chromatographic purification**. After electrolysis, the crude mixture containing **2** was quenched by the addition of 300 μL of water and stirred for 5 min. ***(Caution**: failure to quench the reaction may cause fire upon exposure of the magnesium electrode to air.)* The mixture was then diluted with 20 mL of hexanes and passed through a short pad of Celite to remove the electrolyte. The filtrate was then washed sequentially with saturated NH_4Cl (2 x 10 mL), saturated $NaHCO_3$ (2 x 10 mL), water (2 x 10 mL), and brine (1 x 10 mL). The organic phase was dried over Na_2SO_4, filtered, and concentrated under reduced pressure to obtain the borylated intermediate **2** for the next step without further purification.

To an 8 mL vial, the crude mixture containing **2**, aldehyde (1.2 equiv), and anhydrous toluene (2 mL) were added. The reaction mixture was stirred at 50 °C for 16 h. After completion, the mixture was diluted with 50 mL of diethyl ether and washed with saturated aqueous NH_4Cl (2 x 10 mL). The combined organic layers were dried over Na_2SO_4, filtered, and concentrated under reduced pressure. The crude residue was purified by flash chromatography (EtOAc:hexanes = 1:10) to afford product **5**.

(1*R*,3*S*)-3,7-Dimethyl-1-((1*R*,4*R*)-1-methyl-4-(prop-1-en-2-yl)cyclohex-2-en-1-yl)oct-6-en-1-ol (5a)

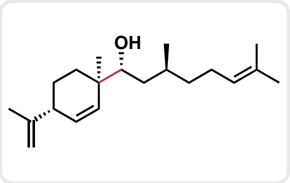

Yield: 50%. Colorless oil. >20:1 d.r.. ^{1}H NMR (400 MHz, $CDCl_3$) δ 5.69 (dd, *J* = 10.1, 2.2 Hz, 1H), 5.46 (dd, *J* = 10.2, 2.7 Hz, 1H), 5.11 (t, *J* = 7.3 Hz, 1H), 4.74 (d, *J* = 7.4 Hz, 2H), 3.41 (d, *J* = 10.4 Hz, 1H), 2.69 (ddt, *J* = 7.6, 5.1, 2.5 Hz, 1H), 1.99 (h, *J* = 7.4 Hz, 2H), 1.80 (dt, *J* = 12.5, 4.5 Hz, 1H), 1.72 (s, 3H), 1.68 (s, 3H), 1.61 (s, 3H), 1.55 – 1.14 (m, 8H), 0.95 (s, 3H), 0.90 (d, *J* = 6.5 Hz, 3H). ^{13}C NMR (101 MHz, $CDCl_3$) δ 149.2, 134.6, 132.4, 131.2, 125.0, 110.2, 75.9, 43.8, 40.1, 38.6, 37.8, 29.5, 28.6, 25.9, 25.8, 25.2, 23.5, 20.7, 18.9, 17.8. HRMS-(DART+) calculated for C20H35O $[M+H]^+$: 291.2683, found: 291.2683.

(*S*)-((1*R*,4*R*)-1-Methyl-4-(prop-1-en-2-yl)cyclohex-2-en-1-yl)(phenyl)methanol (5b)

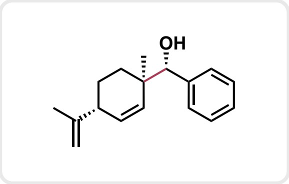

Yield: 94%. Colorless oil. >20:1 d.r.. ^{1}H NMR (400 MHz, $CDCl_3$) δ 7.35 – 7.27 (m, 5H), 5.74 (dt, *J* = 10.3, 1.6 Hz, 1H), 5.55 (ddd, *J* = 10.2, 2.7, 1.4 Hz, 1H), 4.79 – 4.68 (m, 2H), 4.48 (s, 1H), 2.72 (ddt, *J* = 10.3, 5.4, 2.5 Hz, 1H), 1.91 – 1.84 (m, 1H), 1.78 (dt, *J* = 13.5, 4.7 Hz, 1H), 1.72 (s, 3H), 1.51 – 1.43 (m, 1H), 1.32 – 1.19 (m, 2H), 0.95 (s, 3H). ^{13}C NMR (101 MHz, $CDCl_3$) δ 149.0, 140.7, 134.4, 132.6, 128.1, 127.7, 127.6, 110.3, 81.3, 43.6, 40.9, 28.7, 24.9, 23.9, 20.7. HRMS-(DART+) calculated for C17H23O $[M+H]^+$: 243.1743, found: 243.1740.

(*R*)-1-((1*R*,4*R*)-1-Methyl-4-(prop-1-en-2-yl)cyclohex-2-en-1-yl)-3-phenylpropan-1-ol (5c)

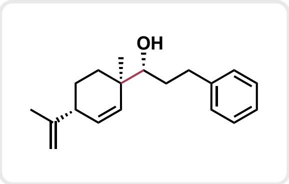

Yield: 50%. Colorless oil. >20:1 d.r.. ^{1}H NMR (400 MHz, $CDCl_3$) δ 7.31 (t, *J* = 7.4 Hz, 2H), 7.22 (dd, *J* = 13.5, 6.5 Hz, 3H), 5.72 (ddd, *J* = 10.2, 2.1, 1.1 Hz, 1H), 5.47 (ddd, *J* = 10.3, 2.7, 1.3 Hz, 1H), 4.76 (dd, *J* = 9.6, 1.9 Hz, 2H), 3.37 (dd, *J* = 10.5, 1.8 Hz, 1H), 2.97 (ddd, *J* = 14.6, 10.3, 5.0 Hz, 1H), 2.74 – 2.61 (m, 2H), 1.91 – 1.75 (m, 3H), 1.74 (s, 3H), 1.71 – 1.59 (m, 2H), 1.56 – 1.37 (m, 2H), 0.97 (s, 3H). ^{13}C NMR (101 MHz, $CDCl_3$) δ 149.0, 142.6, 134.4, 132.7, 128.6, 128.5, 125.9, 110.2, 77.9, 43.7, 40.2, 33.4, 32.5, 28.6, 25.2, 23.6, 20.7. HRMS-(DART+) calculated for C19H27O $[M+H]^+$: 271.2056, found: 271.2054.

(*R*)-2-Methyl-1-((1*R*,4*R*)-1-methyl-4-(prop-1-en-2-yl)cyclohex-2-en-1-yl)prop-2-en-1-ol (5d)

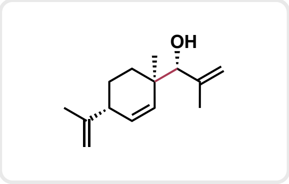

Yield: 64%. Colorless oil. >20:1 d.r.. ^{1}H NMR (500 MHz, $CDCl_3$) δ 5.71 – 5.53 (m, 2H), 5.03 – 4.88 (m, 2H), 4.80 – 4.65 (m, 2H), 3.88 (s, 1H), 2.71 (ddt, *J* = 10.2, 5.3, 2.5 Hz, 1H), 1.86 – 1.75 (m, 4H), 1.72 (m, 3H), 1.61 (s, 1H), 1.56 – 1.36 (m, 3H), 1.02 (s, 2H). ^{13}C NMR (126 MHz, $CDCl_3$) δ 149.2, 145.3, 134.6, 131.8, 114.9, 110.2, 82.8, 43.7, 40.4, 29.7, 25.2, 24.6, 20.7, 20.4. HRMS-(DART+) calculated for C14H23O $[M+H]^+$: 207.1744, found: 207.1743.

(*R*)-1-((1*R*,3*S*,5*R*)-6,6-Dimethyl-2-methylenebicyclo[3.1.1]heptan-3-yl)-3-phenylpropan-1-ol (5e)

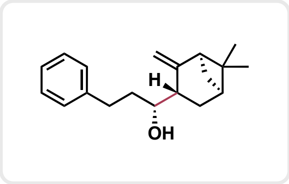

Yield: 74%. Colorless oil. >20:1 d.r.. ^{1}H NMR (400 MHz, $CDCl_3$) δ 7.32 – 7.18 (m, 5H), 4.88 (s, 1H), 4.81 (s, 1H), 3.40 (t, *J* = 8.8 Hz, 1H), 2.98 (ddd, *J* = 14.8, 10.6, 4.9 Hz, 1H), 2.72 (ddd, *J* = 13.7, 10.2, 6.5 Hz, 1H), 2.59 (s, 1H), 2.49 (t, *J* = 5.4 Hz, 1H), 2.39 (td, *J* = 9.4, 2.1 Hz, 1H), 2.28 (dtd, *J* = 11.8, 5.9, 2.9 Hz, 1H), 2.13 – 1.94 (m, 3H), 1.66 (dp, *J* = 14.1, 4.8 Hz, 1H), 1.56 (dt, *J* = 13.6, 3.1 Hz, 1H), 1.27 (s, 3H), 1.24 (s, 1H), 0.75 (s, 3H). ^{13}C NMR (101 MHz, $CDCl_3$) δ 152.2, 142.7, 128.6, 128.5, 125.8, 111.3, 74.8, 52.4, 42.1, 41.3, 40.8, 37.1, 32.9, 27.8, 26.6, 25.9, 21.7. HRMS-(DART+) calculated for C19H27O $[M+H]^+$: 271.2056, found: 271.2055.

tert-Butyl (_S_)-2-((_S_)-((1_R_,3_S_,5_R_)-6,6-dimethyl-2-methylenebicyclo[3.1.1]heptan-3-yl)(hydroxy)methyl) pyrrolidine-1-carboxylate (5f)

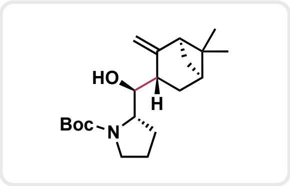

Yield: 87%. White solid. >20:1 d.r.. ^{1}H NMR (400 MHz, $CDCl_3$) δ 4.82 (s, 1H), 4.70 (s, 1H), 4.25 (s, 1H), 3.84 – 3.39 (m, 2H), 3.33 (dt, J = 11.3, 5.9 Hz, 1H), 2.71 (s, 1H), 2.41 (q, J = 6.2 Hz, 1H), 2.23 (dq, J = 11.4, 5.4 Hz, 1H), 2.17 – 2.08 (m, 1H), 2.01 – 1.87 (m, 4H), 1.80 (ddt, J = 12.4, 9.1, 4.6 Hz, 2H), 1.45 (s, 9H), 1.22 (d, J = 7.2 Hz, 4H), 1.19 – 1.12 (m, 1H), 0.72 (s, 3H). ^{13}C NMR (101 MHz, $CDCl_3$) δ 152.2, 110.4, 82.9, 82.5, 79.8, 59.6, 52.6, 47.4, 41.3, 40.7, 38.6, 29.1, 28.6, 26.5, 25.9, 24.9, 24.4, 21.7. HRMS-(DART+) calculated for C20H34NO3 [M+H]$^{+}$: 336.2533, found: 336.2529. The structure was characterized by X-ray crystallography and single crystals were obtained by vapor diffusion of pentane into a dichloromethane solution of the compound.

4-((_S_)-((1_R_,3_S_,5_R_)-6,6-Dimethyl-2-methylenebicyclo[3.1.1]heptan-3-yl)(hydroxy)methyl)-2-methoxy phenol (5g)

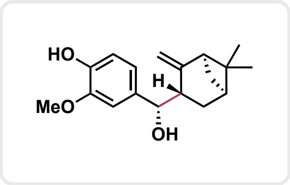

Yield: 92%. Colorless oil. >20:1 d.r.. ^{1}H NMR (500 MHz, $CDCl_3$) δ 6.85 (d, J = 1.1 Hz, 1H), 6.81 (d, J = 1.1 Hz, 2H), 5.56 (t, J = 1.8 Hz, 1H), 4.93 (t, J = 1.6 Hz, 1H), 4.87 (t, J = 1.6 Hz, 1H), 4.28 (d, J = 9.6 Hz, 1H), 3.84 (s, 3H), 2.91 (s, 1H), 2.65 (dddd, J = 11.2, 9.7, 2.3, 1.2 Hz, 1H), 2.46 (t, J = 5.5 Hz, 1H), 2.22 (dtd, J = 10.2, 5.8, 1.8 Hz, 1H), 1.84 (tdd, J = 5.9, 4.0, 2.1 Hz, 1H), 1.66 (ddt, J = 13.6, 9.5, 1.8 Hz, 1H), 1.43 (ddd, J = 14.3, 4.0, 2.3 Hz, 1H), 1.23 (d, J = 10.3 Hz, 1H), 1.18 (s, 3H), 0.65 (s, 3H). ^{13}C NMR (126 MHz, $CDCl_3$) δ 152.8, 146.8, 145.4, 134.4, 121.4, 114.0, 111.7, 109.9, 78.7, 56.1, 52.5, 43.2, 41.5, 40.6, 27.1, 26.6, 25.8, 21.6. HRMS-(DART+) calculated for C18H25O3 [M+H]$^{+}$: 289.1798, found: 289.1797.

2,6-Dimethyl-1-phenyl-2-vinylhept-5-en-1-ol (5h)

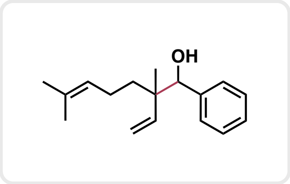

Yield: 80%. Colorless oil. 3:1 d.r.. ^{1}H NMR (400 MHz, $CDCl_3$) isomer mixture: δ 7.29 – 7.20 (m, 5H), 5.87 – 5.70 (m, 1H), 5.28 – 5.13 (m, 1H), 5.10 – 4.92 (m, 2H), 4.39 (d, J = 6.3 Hz, 1H), 2.03 (s, 1H), 1.82 (h, J = 7.8 Hz, 2H), 1.62 (s, 3H), 1.52 (s, 3H), 1.41 – 1.32 (m, 1H), 1.29 – 1.19 (m, 1H), 1.04 (s, 1H), 0.89 (s, 2H). ^{13}C NMR (101 MHz, $CDCl_3$) major isomer: δ 144.1, 140.6, 131.4, 128.2, 127.6, 127.5, 124.9, 115.9, 80.2, 46.1, 37.7, 25.8, 22.9, 17.8. HRMS-(DART+) calculated for C17H25O [M+H]$^{+}$: 245.1899, found: 245.1898.

6.4. General Procedures for C–N Coupling

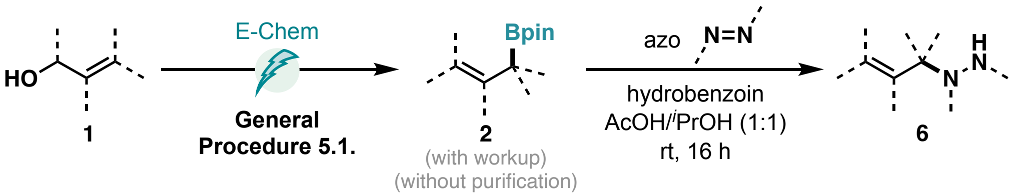

General Procedure: The borylated intermediate **2** was obtained from 0.5 mmol of **1** following *General Procedure 5.1.*, Steps 1 and 2, **including the workup step but without chromatographic purification**. After electrolysis, the crude mixture containing **2** was quenched by the addition of 300 µL of water and stirred for 5 min. ***(Caution**: failure to quench the reaction may cause fire upon exposure of the magnesium electrode to air.)* The mixture was then diluted with 20 mL of hexanes and passed through a short pad of Celite to remove the electrolyte. The filtrate was then washed sequentially with saturated NH_4Cl (2 x 10 mL), saturated $NaHCO_3$ (2 x 10 mL), water (2 x 10 mL), and brine (1 x 10 mL). The organic phase was dried over Na_2SO_4, filtered, and concentrated under reduced pressure to obtain the borylated intermediate **2** for the next step without further purification.

To an 8 mL vial, the crude mixture containing **2**, isopropanol (0.5 mL), acetic acid (0.5 mL), and hydrobenzoin (0.2 equiv) were added. The mixture was stirred at rt for 10 min, followed by the addition of azo compound (1.1 equiv). The reaction was then stirred at rt for 16 h. Upon completion, the mixture was concentrated under reduced pressure, and the crude residue was purified by flash chromatography (EtOAc:hexanes = 1:4) to afford product **6**.

Diisopropyl 1-(3,7-dimethylocta-1,6-dien-3-yl)hydrazine-1,2-dicarboxylate (6a)

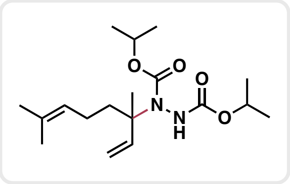

Yield: 31%. Colorless oil. ^{1}H NMR (500 MHz, $CDCl_3$) δ 6.32 – 5.99 (m, 2H), 5.14 – 5.00 (m, 3H), 4.99 – 4.84 (m, 2H), 2.13 – 1.88 (m, 3H), 1.71 – 1.58 (m, 7H), 1.47 – 1.32 (m, 3H), 1.23 (dddd, J = 17.0, 10.5, 6.3, 4.1 Hz, 12H). ^{13}C NMR (126 MHz, $CDCl_3$) δ 156.8, 144.4, 142.3, 131.7, 124.3, 111.6, 69.7, 64.9, 38.8, 38.1, 25.8, 23.6, 23.13, 23.10, 22.2, 22.1, 22.0, 17.8. HRMS-(DART+) calculated for C18H33N2O4 $[M+H]^+$: 341.2435, found: 341.2435.

6.5. General Procedures for Vinylogous Homologation

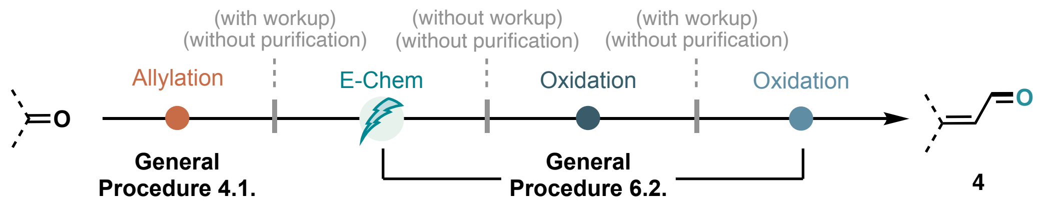

General Procedure: Ketone (0.5 mmol) was subjected to allylation following *General Procedure 4.1.*, **including the aqueous workup but without chromatographic purification**. After the workup, the concentrated crude mixture followed by *General Procedures 6.2.*, then the final crude product was purified by flash chromatography (EtOAc:hexanes = 1:20) to afford product **4**.

(*E*)-2-((2*S*,5*R*)-2-Isopropyl-5-methylcyclohexylidene)acetaldehyde (4c)

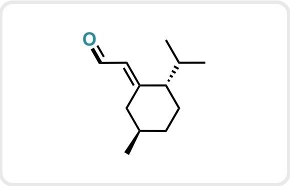

Yield: 55%. Yellow oil. >20:1 r.r.. 2.7:1 *E*:*Z*. ^{1}H NMR (400 MHz, $CDCl_3$) isomer mixture: δ 10.02 (dd, *J* = 13.9, 8.3 Hz, 1H), 5.88 (dd, *J* = 8.1, 1.0 Hz, 1H), 2.94 (td, *J* = 15.0, 7.3 Hz, 1H), 2.63 – 2.16 (m, 1H), 2.03 – 1.79 (m, 5H), 1.76 – 1.40 (m, 1H), 1.22 (ddt, *J* = 12.9, 11.2, 5.2 Hz, 1H), 1.00 (dd, *J* = 9.2, 6.6 Hz, 3H), 0.92 (dd, *J* = 6.9, 3.7 Hz, 3H), 0.87 (d, *J* = 6.6 Hz, 2H), 0.83 – 0.72 (m, 1H). ^{13}C NMR (101 MHz, $CDCl_3$) major isomer: δ 190.9, 169.9, 125.8, 52.8, 36.3, 34.2, 31.6, 27.6, 26.9, 21.9, 20.8, 19.6. HRMS-(DART+) calculated for C12H21O $[M+H]^+$: 181.1587, found: 181.1587.

(*E*)-2-((1*R*,4*R*)-1,7,7-Trimethylbicyclo[2.2.1]heptan-2-ylidene)acetaldehyde (4d)

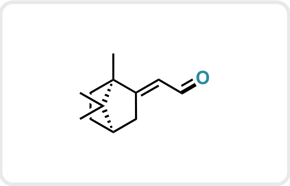

Yield: 22%. Yellow oil. >20:1 r.r.. 2:1 *E*:*Z*. ^{1}H NMR (400 MHz, $CDCl_3$) isomer mixture: δ 10.22 – 9.80 (m, 1H), 5.93 – 5.76 (m, 1H), 2.95 – 2.57 (m, 1H), 2.52 – 2.04 (m, 1H), 1.96 – 1.71 (m, 3H), 1.57 – 1.38 (m, 1H), 1.37 (s, 2H), 1.26 (d, *J* = 8.9 Hz, 1H), 1.00 – 0.92 (m, 4H), 0.85 (s, 2H), 0.78 (s, 1H). ^{13}C NMR (101 MHz, $CDCl_3$) major isomer: δ 191.0, 177.3, 125.2, 55.9, 50.3, 44.3, 40.7, 35.1, 27.8, 20.0, 18.9, 17.1. HRMS-(DART+) calculated for C12H19O $[M+H]^+$: 179.1430, found: 179.1430.

(*E*)-3,7-dimethylocta-2,6-dienal (4e)

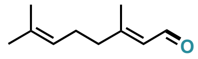

Yield: 34%. Yellow oil. >20:1 r.r.. 3:1 *E*:*Z*. ^{1}H NMR (400 MHz, $CDCl_3$) isomer mixture: δ 10.04 – 9.82 (m, 1H), 5.93 – 5.84 (m, 1H), 5.15 – 5.02 (m, 1H), 2.61 – 2.24 (m, 1H), 2.21 (d, *J* = 6.8 Hz, 2H), 2.17 (d, *J* = 1.3 Hz, 2H), 1.98 (d, *J* = 1.3 Hz, 1H), 1.71 – 1.67 (m, 3H), 1.65 – 1.54 (m, 4H). ^{13}C NMR (126 MHz, $CDCl_3$) major isomer: δ 191.5, 164.0, 133.1, 127.6, 122.7, 40.8, 25.9, 25.8, 17.9, 17.7. HRMS-(DART+) calculated for C10H17O $[M+H]^+$: 153.1274, found: 153.1275.

(*R*,*E*)-2-(2-Methyl-5-(prop-1-en-2-yl)cyclohex-2-en-1-ylidene)acetaldehyde (4f)

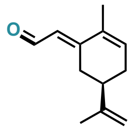

Yield: 41%. Yellow oil. >20:1 r.r.. 9:1 *E*:*Z*. ^{1}H NMR (400 MHz, $CDCl_3$) δ 10.16 (d, *J* = 8.1 Hz, 1H), 6.17 (dd, *J* = 6.1, 3.4 Hz, 1H), 5.94 (d, *J* = 8.1 Hz, 1H), 4.83 – 4.78 (m, 2H), 3.38 (dd, *J* = 9.2, 4.7 Hz, 1H), 2.48 – 2.33 (m, 3H), 2.25 – 2.13 (m, 1H), 1.86 (s, 3H), 1.77 (s, 3H). ^{13}C NMR (101 MHz, $CDCl_3$) δ 191.6, 157.3, 147.8, 137.4, 133.0, 123.2, 110.5, 41.4, 31.9, 30.9, 20.7, 19.5. HRMS-(DART+) calculated for C12H17O $[M+H]^+$: 177.1274, found: 177.1275.

7. X-ray Crystallography

Low-temperature X-ray diffraction data were collected on a Rigaku XtaLAB Synergy diffractometer coupled to a Rigaku Hypix detector with either Mo Kα radiation (λ = 0.71073 Å) or Cu Kα radiation (λ = 1.54184 Å), from a PhotonJet micro-focus X-ray source at 100 K. The diffraction images were processed and scaled using the CrysAlisPro software.[15] The structures were solved through intrinsic phasing using SHELXT[16] and refined against F^2 on all data by full-matrix least squares with SHELXL[17] following established refinement strategies.[18] All non-hydrogen atoms were refined anisotropically. All hydrogen atoms bound to carbon were included in the model at geometrically calculated positions and refined using a riding model. The isotropic displacement parameters of all hydrogen atoms were fixed to 1.2 times the U_{eq} value of the atoms they are linked to (1.5 times for methyl groups). Crystallographic data are available from the Cambridge Crystallographic Database Centre under accession codes CCDC 2434840.

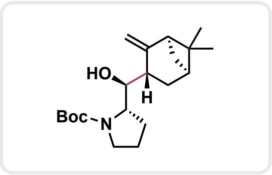

CCDC 2434840

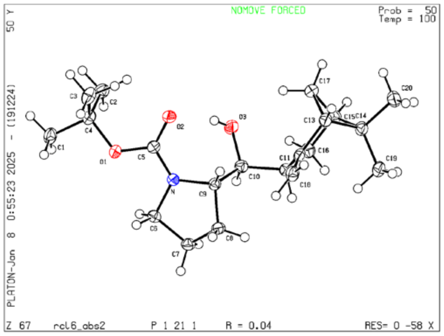

Table S3. Crystal data and structure refinement for C20H33NO3.

Identification code	rcl6_abs2	
Empirical formula	C20 H33 N O3	
Formula weight	335.47	
Temperature	100.00(10) K	
Wavelength	1.54184 Å	
Crystal system	Monoclinic	
Space group	P 1 21 1	
Unit cell dimensions	a = 10.3990(2) Å	α= 90°.
	b = 6.47340(10) Å	β= 97.5210(10)°.
	c = 13.9305(2) Å	γ = 90°.
Volume	929.69(3) Å^3	
Z	2	
Density (calculated)	1.198 Mg/m^3	
Absorption coefficient	0.625 mm^{-1}	
F(000)	368	
Crystal size	0.372 x 0.059 x 0.029 mm^3	
Theta range for data collection	3.200 to 79.872°.	
Index ranges	-13<=h<=13, -8<=k<=8, -17<=l<=13	
Reflections collected	20405	
Independent reflections	4020 [R(int) = 0.0637]	
Completeness to theta = 67.684°	100.0 %	
Absorption correction	Gaussian	
Max. and min. transmission	1.000 and 0.540	
Refinement method	Full-matrix least-squares on F^2	
Data / restraints / parameters	4020 / 2 / 226	
Goodness-of-fit on F^2	1.123	
Final R indices [I>2sigma(I)]	R1 = 0.0410, wR2 = 0.1090	
R indices (all data)	R1 = 0.0436, wR2 = 0.1126	
Absolute structure parameter	0.11(11)	
Extinction coefficient	n/a	
Largest diff. peak and hole	0.190 and -0.243 e.Å^{-3}	

8. References

(1) Deng, Z. M.; Wei, J. L.; Liao, L. H.; Huang, H. Y.; Zhao, X. D. Organoselenium-Catalyzed, Hydroxy-Controlled Regio- and Stereoselective Amination of Terminal Alkenes: Efficient Synthesis of 3-Amino Allylic Alcohols. *Org. Lett.* **2015**, *17*, 1834–1837.

(2) Williams, R. B.; Eldridge, G.; Starks, C. M.; Guzzo, P. R.; Huang, Z., Phenethyldihydrobenzodioxolones and Methods of Use. U.S. Patent No 9,562,031: 2017.

(3) Zheng, H. C.; Lejkowski, M.; Hall, D. G. Mild and Selective Boronic Acid Catalyzed 1,3-Transposition of Allylic Alcohols and Meyer-Schuster Rearrangement of Propargylic Alcohols. *Chem. Sci.* **2011**, *2*, 1305–1310.

(4) Miralles, N.; Alam, R.; Szabó, K. J.; Fernández, E. Transition-Metal-Free Borylation of Allylic and Propargylic Alcohols. *Angew. Chem., Int. Ed.* **2016**, *55*, 4303–4307.

(5) Özdemirhan, D. Optically Active Tertiary Alcohols by Biocatalysis. *Synth. Commun.* **2017**, *47*, 629–645.

(6) Zhang, J. T.; Zhang, L. L.; Xie, W.; Chen, M.; Zhang, C.; Qin, Y. L.; Zhao, J. Y.; Wang, F.; Liu, Z. Q. Electrochemical Allylation of Aldehydes and Ketones with Allylic Alcohols. *Green Chem.* **2024**, *26*, 7002–7006.

(7) Pace, V.; Castoldi, L.; Hoyos, P.; Sinisterra, J. V.; Pregnolato, M.; Sánchez-Montero, J. M. Highly Regioselective Control of 1,2-Addition of Organolithiums to α,β-Unsaturated Compounds Promoted by Lithium Bromide in 2-Methyltetrahydrofuran: A Facile and Eco-Friendly Access to Allylic Alcohols and Amines. *Tetrahedron* **2011**, *67*, 2670–2675.

(8) Rohlfs, T.; Gerken, L.; Nova-Fernández, J. L.; Malagón, S.; Uygur, M.; Cabrera, S.; Alemán, J.; Mancheño, O. G. Visible-Light-Mediated Selective Allylic C–H Oxygenation of Cycloalkenes. *Synlett* **2024**, *35*, 1047–1051.

(9) Li, J. J.; Tan, C. H.; Gong, J. X.; Yang, Z. Palladium-Catalyzed Oxidative Rearrangement of Tertiary Allylic Alcohols to Enones with Oxygen in Aqueous Solvent. *Org. Lett.* **2014**, *16*, 5370–5373.

(10) Rigotti, T.; Schwinger, D. P.; Grassl, R.; Jandl, C.; Bach, T. Enantioselective Crossed Intramolecular [2+2] Photocycloaddition Reactions Mediated by a Chiral Chelating Lewis Acid. *Chem. Sci.* **2022**, *13*, 2378–2384.

(11) Su, W.; Wang, T. T.; Tian, X.; Han, J. R.; Zhen, X. L.; Fan, S. M.; You, Y. X.; Zhang, Y. K.; Qiao, R. X.; Cheng, Q. S.; Liu, S. X. Stereoselective Dehydroxyboration of Allylic Alcohols to Access (*E*)-Allylboronates by a Combination of C–OH Cleavage and Boron Transfer under Iron Catalysis. *Org. Lett.* **2021**, *23*, 9094–9099.

(12) Serebryakov, E.; Gamalevich, G. Synthesis of 2-Acetoxy-3,7-dimethylpentadecane(diprionylacetate) Involving Two Sigmatropic Rearrangements. *Bulletin of the Academy of Sciences of the USSR, Division of chemical science* **1987**, *36*, 99–103.

(13) Park, K.; Oka, N.; Sawama, Y.; Ikawa, T.; Yamada, T.; Sajiki, H. Platinum on Carbon-Catalysed Site-Selective H–D Exchange Reaction of Allylic Alcohols Using Alkyl amines as a Hydrogen Source. *Org. Chem. Front.* **2022**, *9*, 1986–1991.

(14) Ndungu, J. M.; Larson, K. K.; Sarpong, R. Development of an Anomalous Heck Reaction: Skeletal Rearrangement of Divinyl and Enyne Carbinols. *Org. Lett.* **2005**, *7*, 5845–5848.

(15) CrysAlisPro, R. O. D. version 1.171. 39.15 e. *Rigaku Corporation, The Woodlands, TX* **2015**.

(16) Sheldrick, G. M. SHELXT–Integrated Space-Group and Crystal-Structure Determination. *Acta Crystallogr. A* **2015**, *71*, 3–8.

(17) Sheldrick, G. M. A Short History of SHELXT. *Acta Crystallogr. A* **2008**, *64*, 112–122.

(18) Müller, P. Practical Suggestions for Better Crystal Structures. *Crystallogr. Rev.* **2009**, *15*, 57–83.

9. NMR Spectra

^{1}H NMR of **1d** in Chloroform-*d*

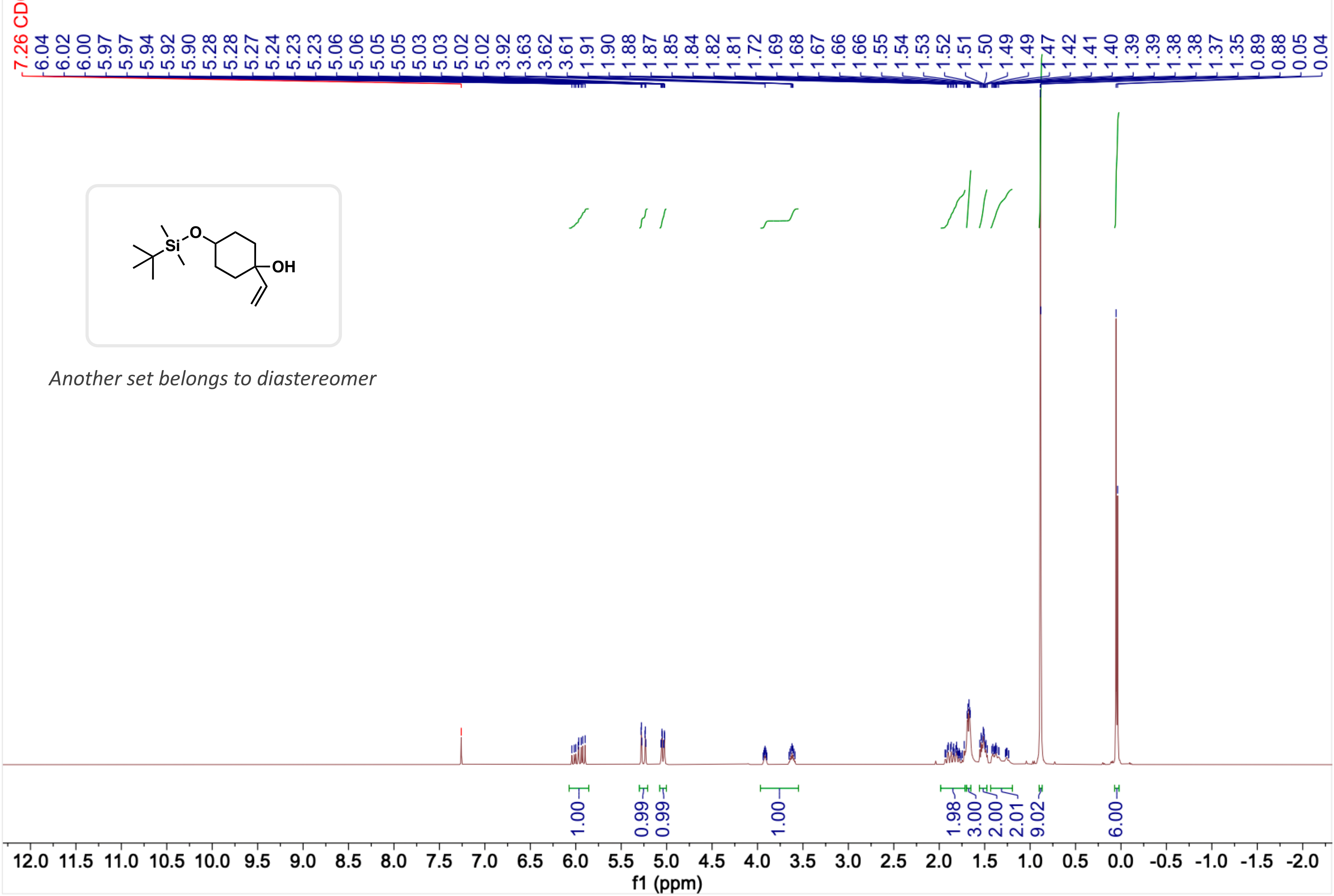

^{13}C NMR of **1d** in Chloroform-*d*

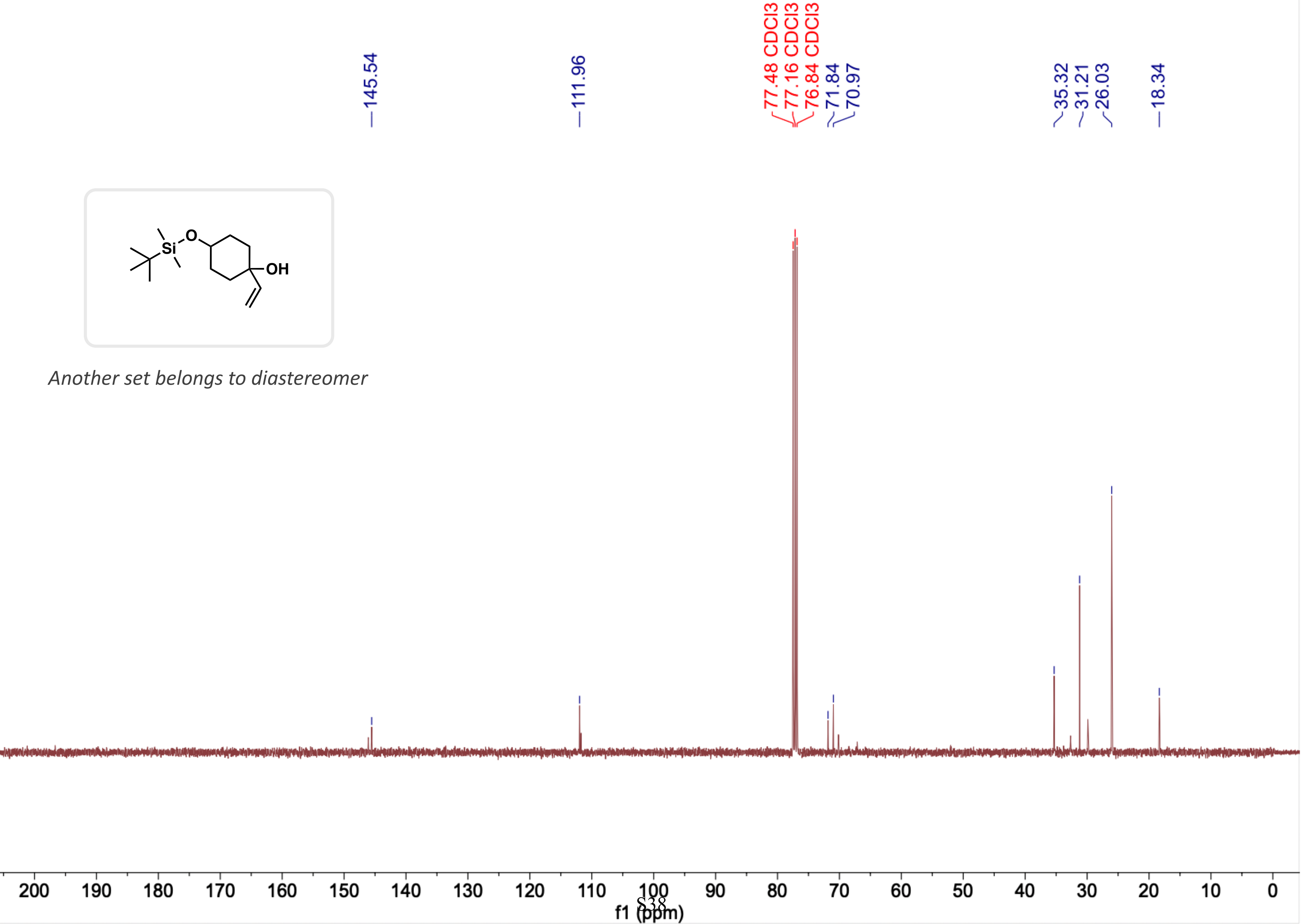

Another set belongs to diastereomer

^{1}H NMR of **1o** in Chloroform-*d*

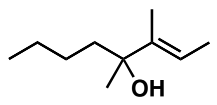

Another set belongs to diastereomer

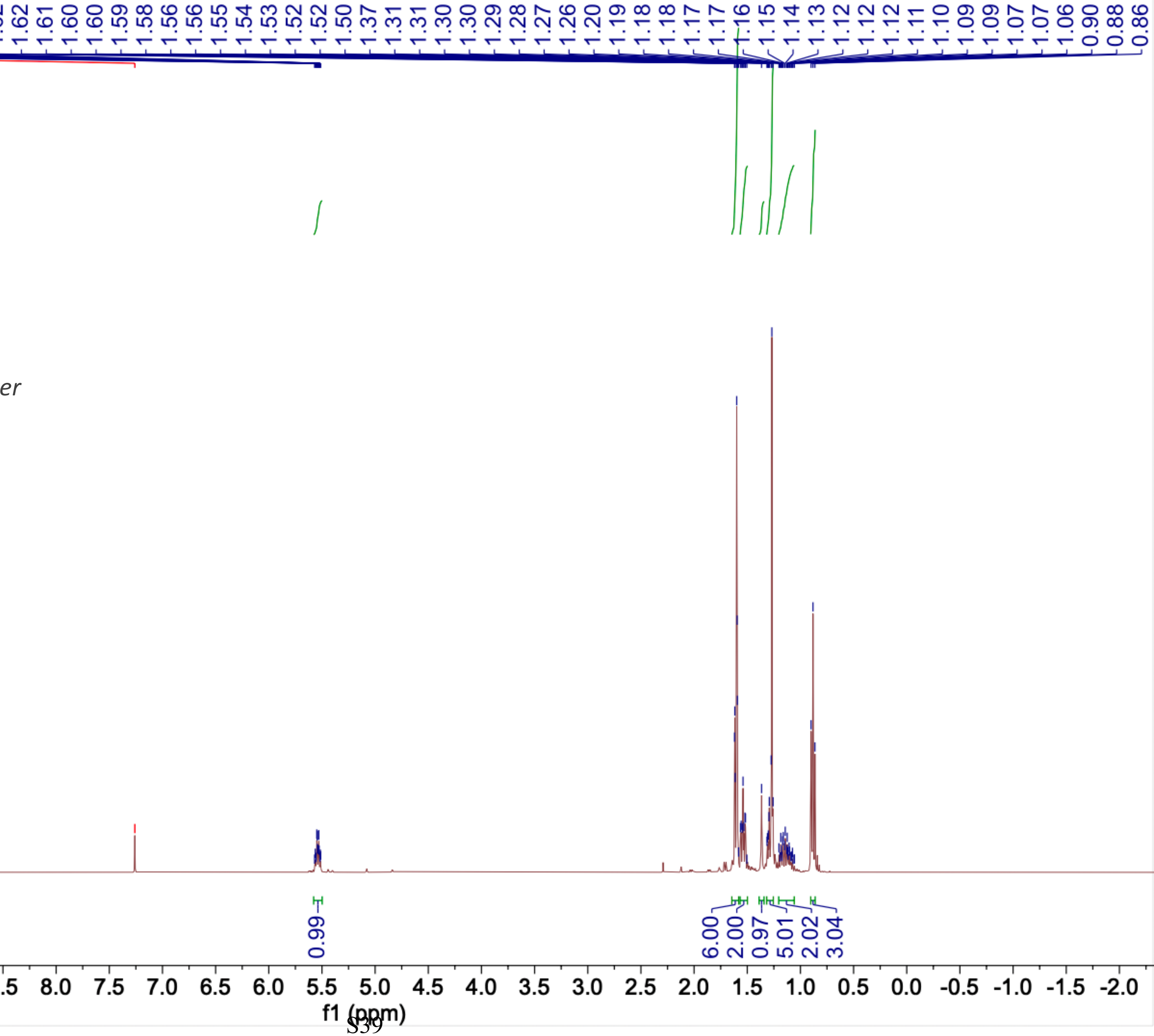

^{13}C NMR of **1o** in Chloroform-*d*

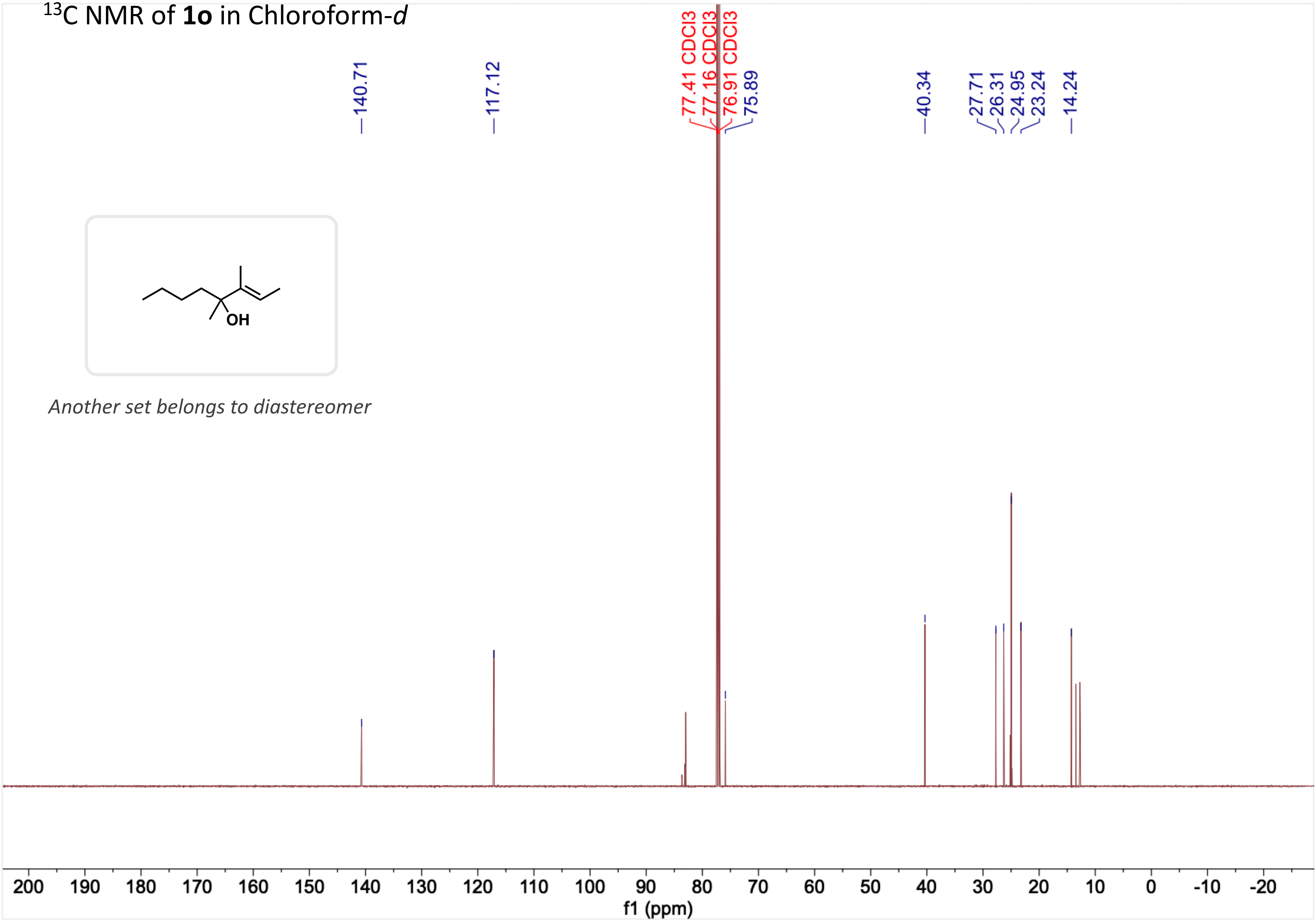

^{1}H NMR of **2a** in Chloroform-*d*

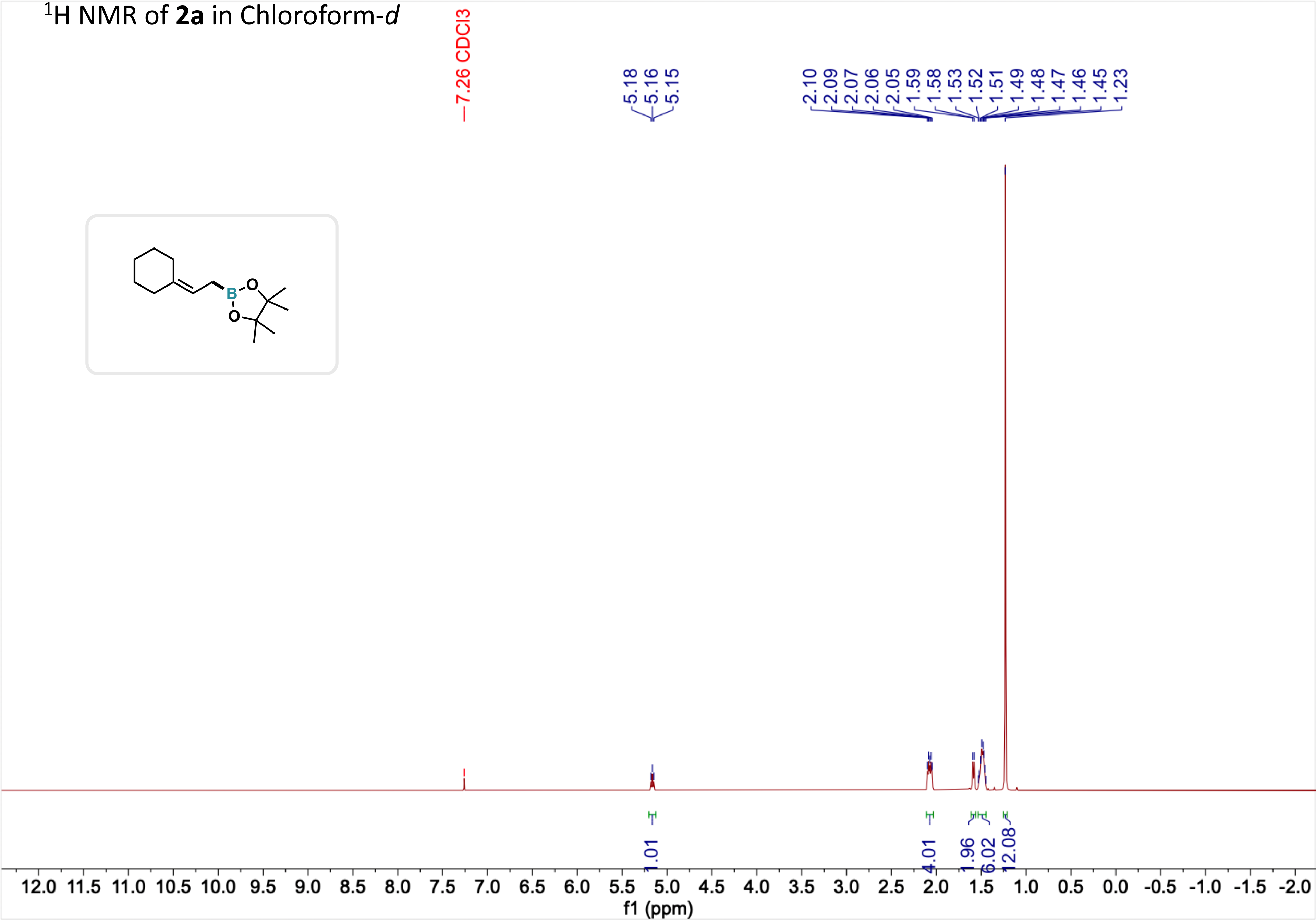

^{13}C NMR of **2a** in Chloroform-*d*

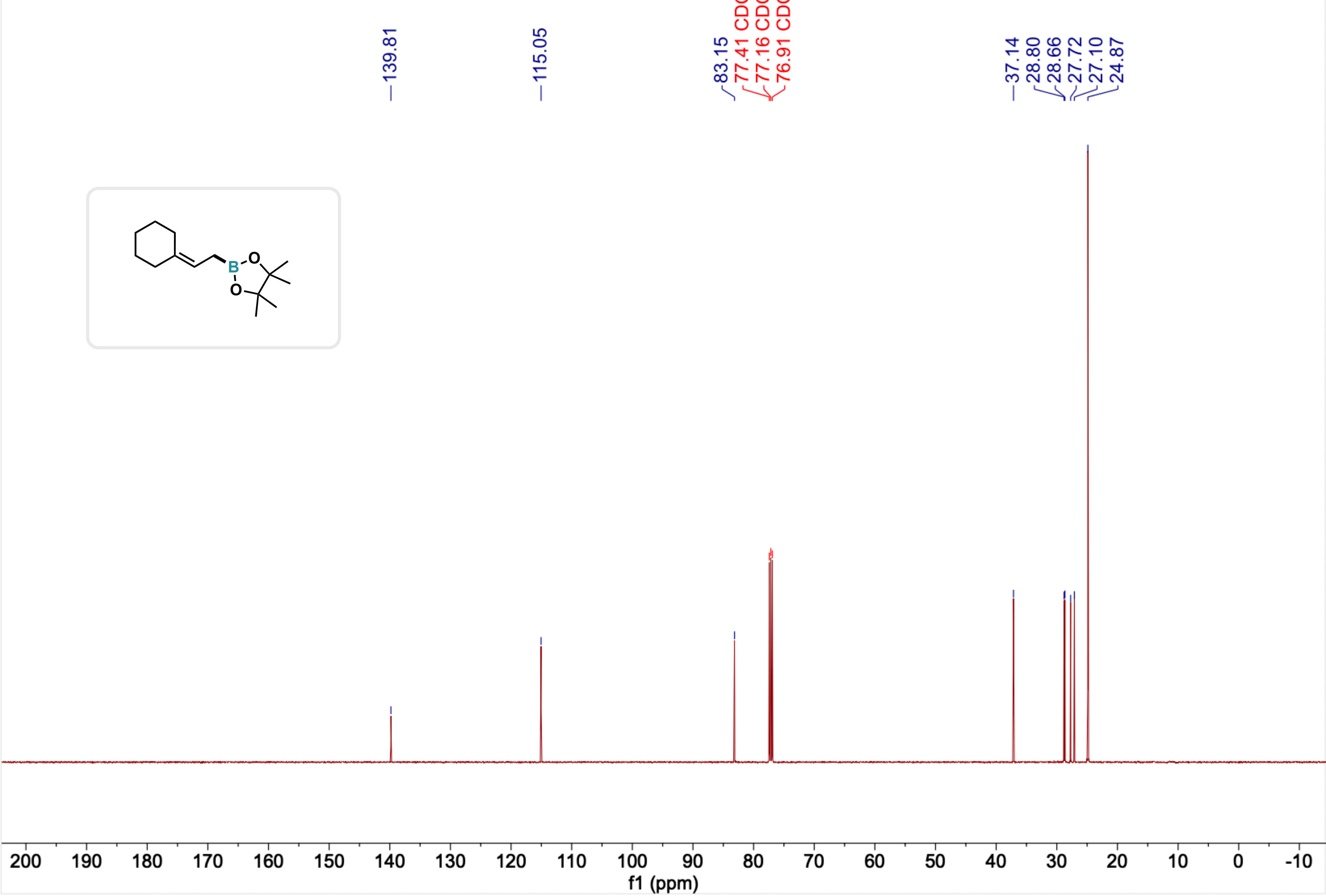

^{11}B NMR of **2a** in Chloroform-*d*

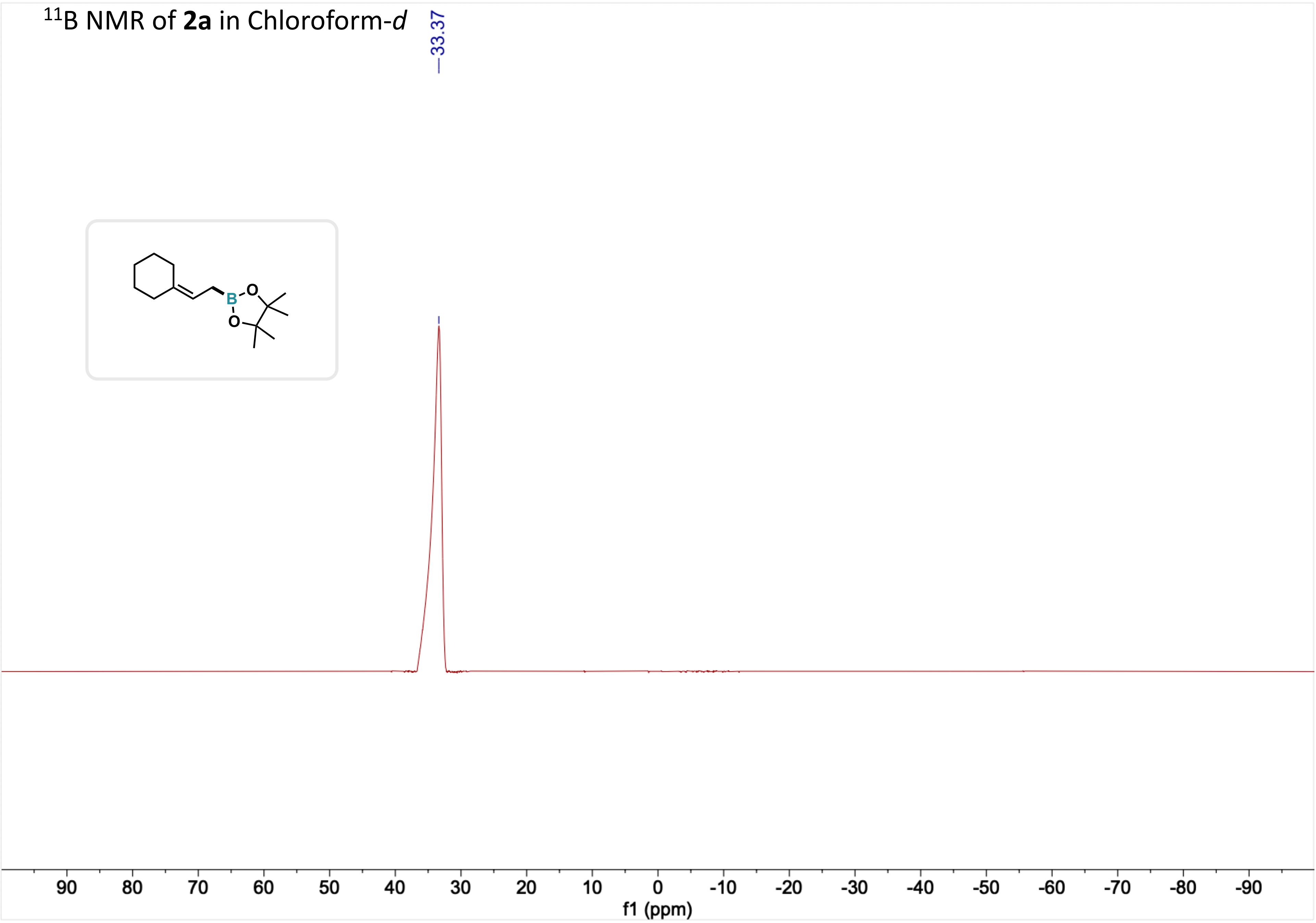

^{1}H NMR of **2b** in Chloroform-*d*

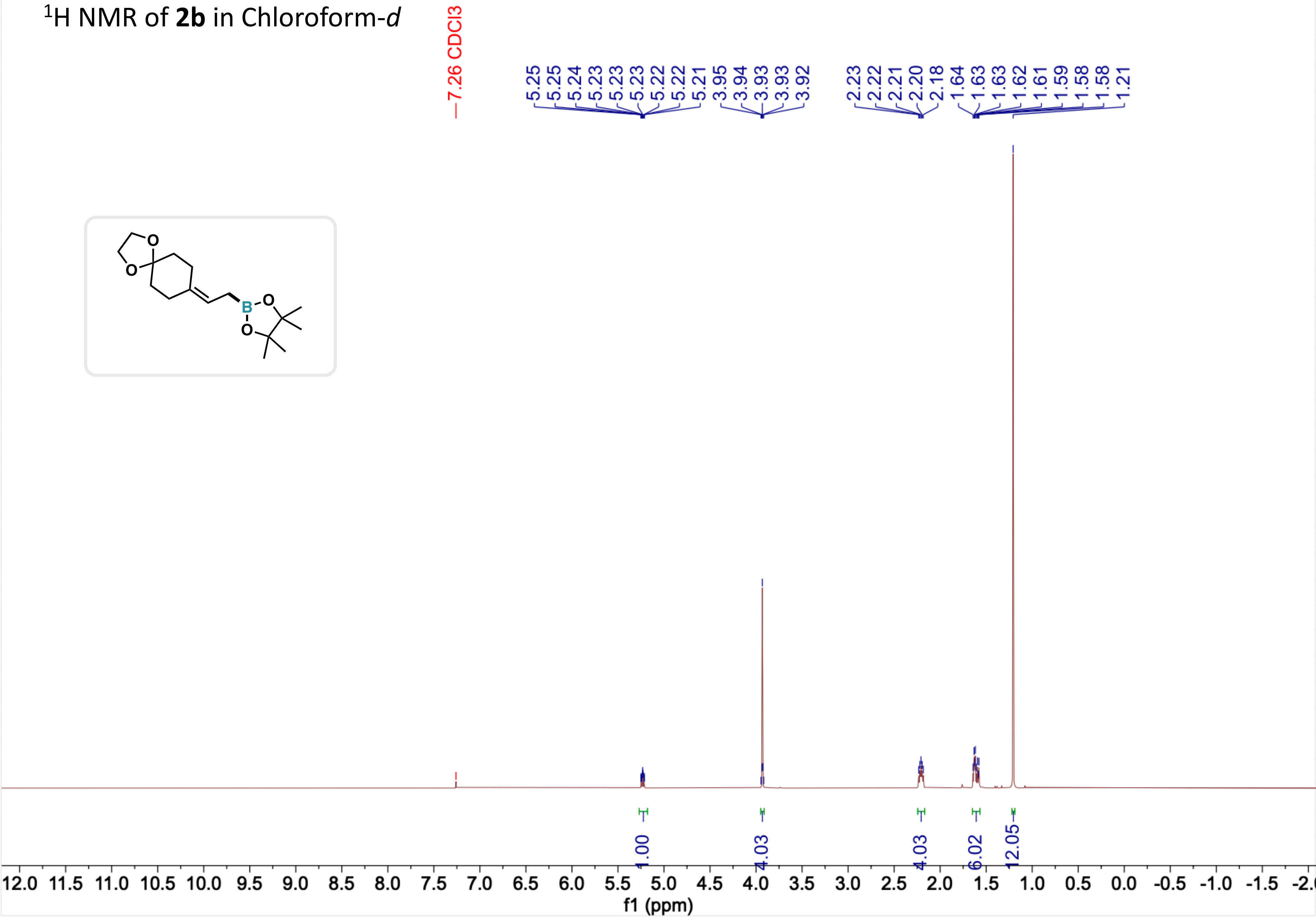

^{13}C NMR of **2b** in Chloroform-*d*

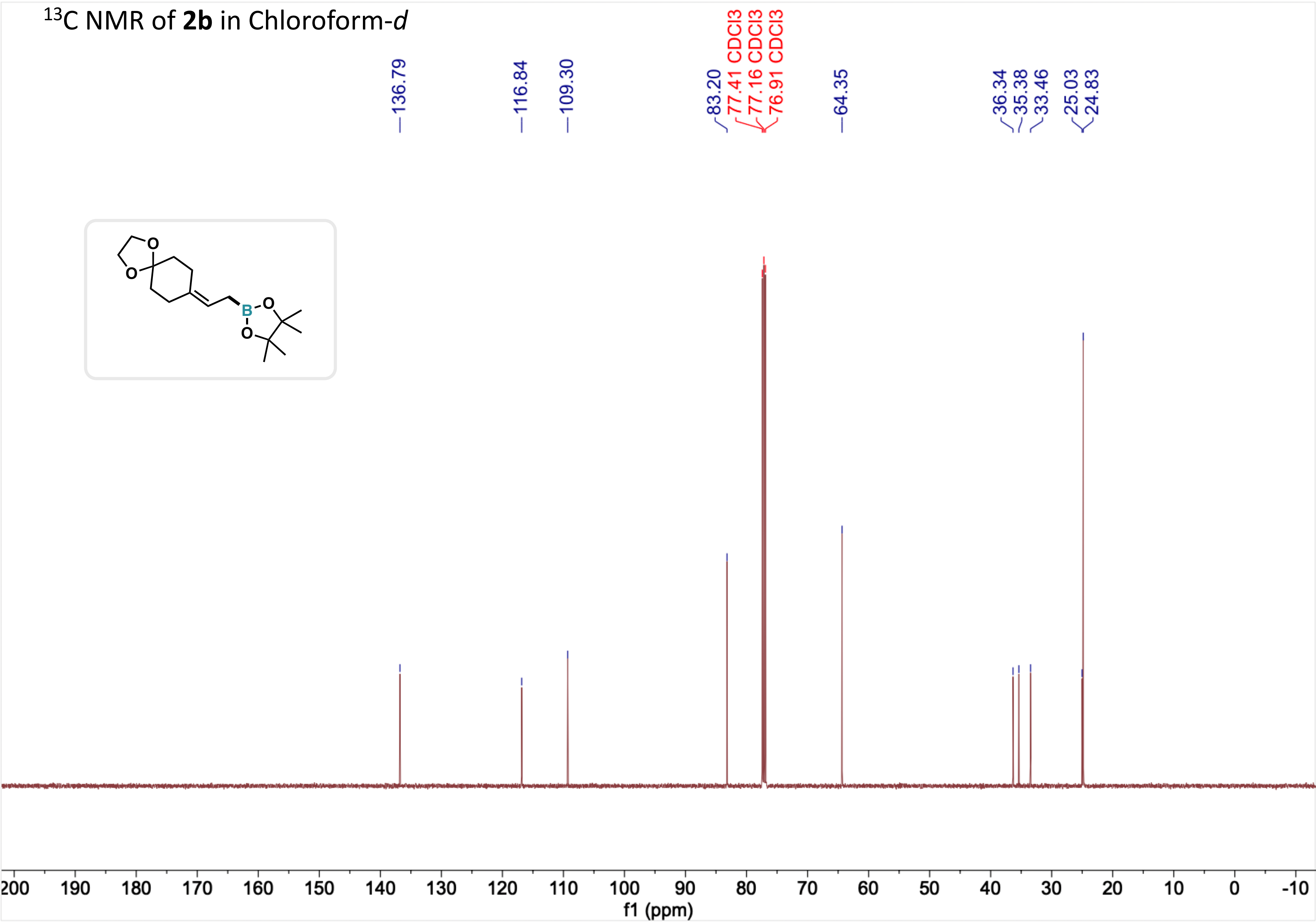

^{11}B NMR of **2b** in Chloroform-*d*

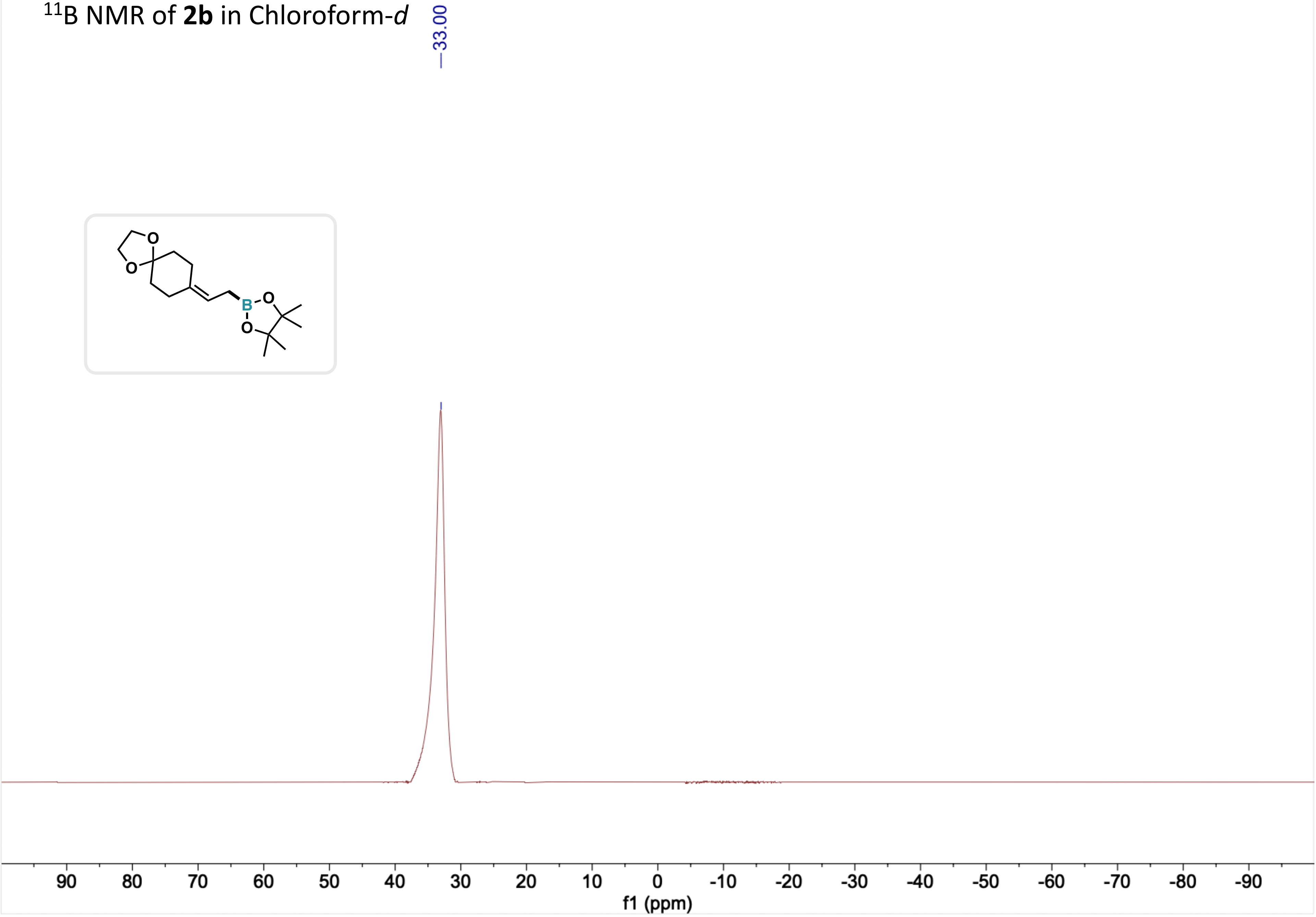

^{1}H NMR of **2c** in Chloroform-*d*

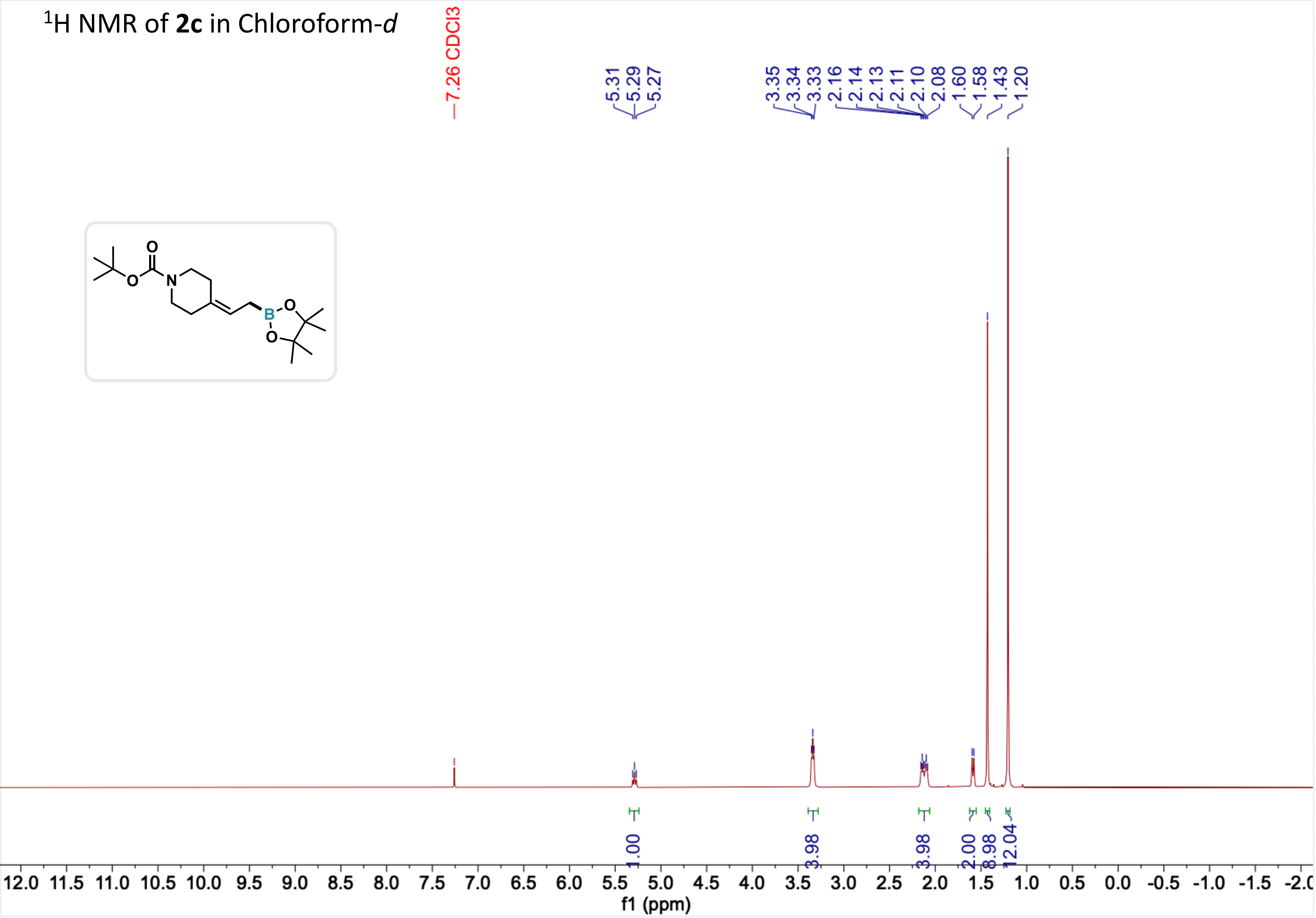

^{13}C NMR of **2c** in Chloroform-*d*

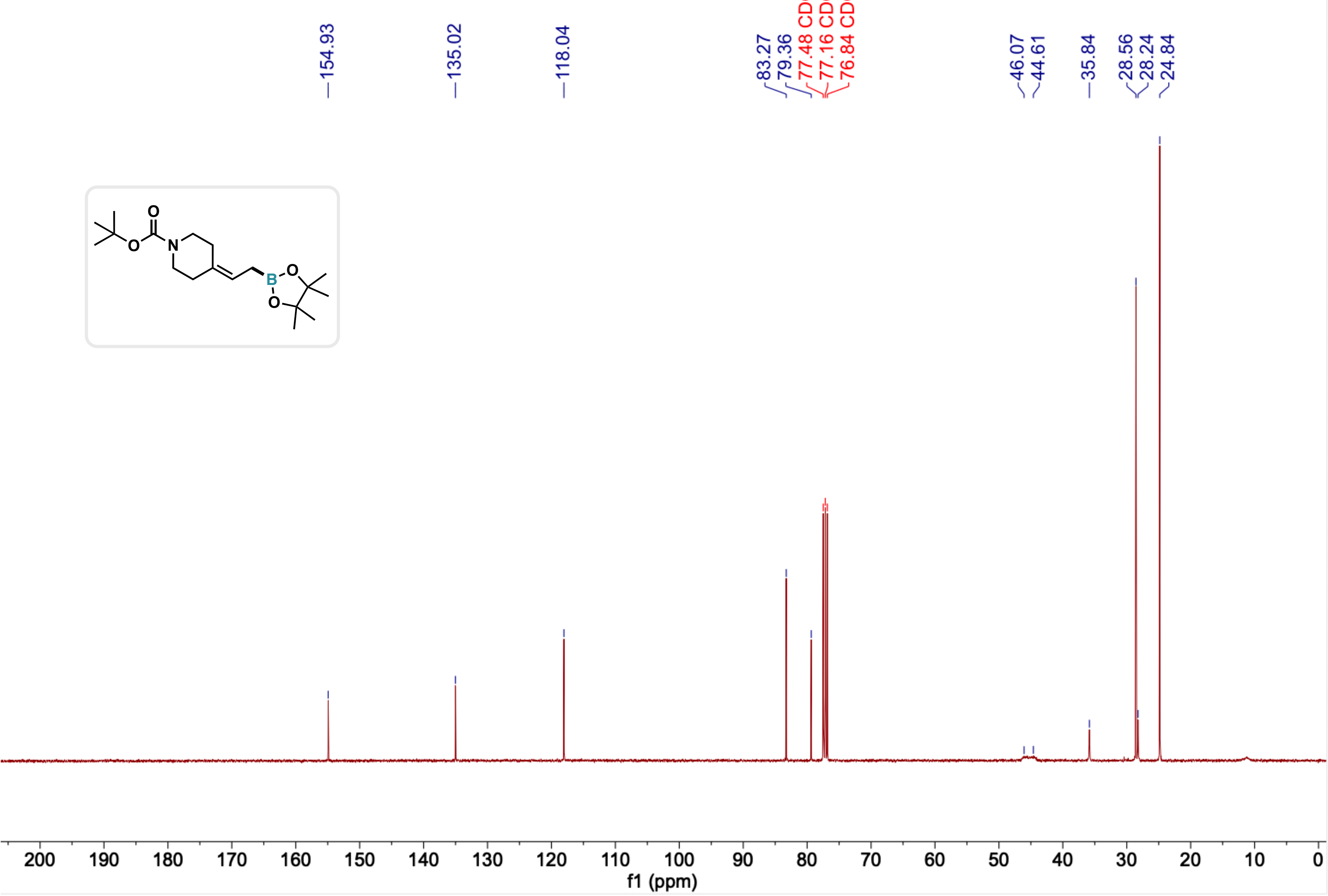

^{11}B NMR of **2c** in Chloroform-*d*

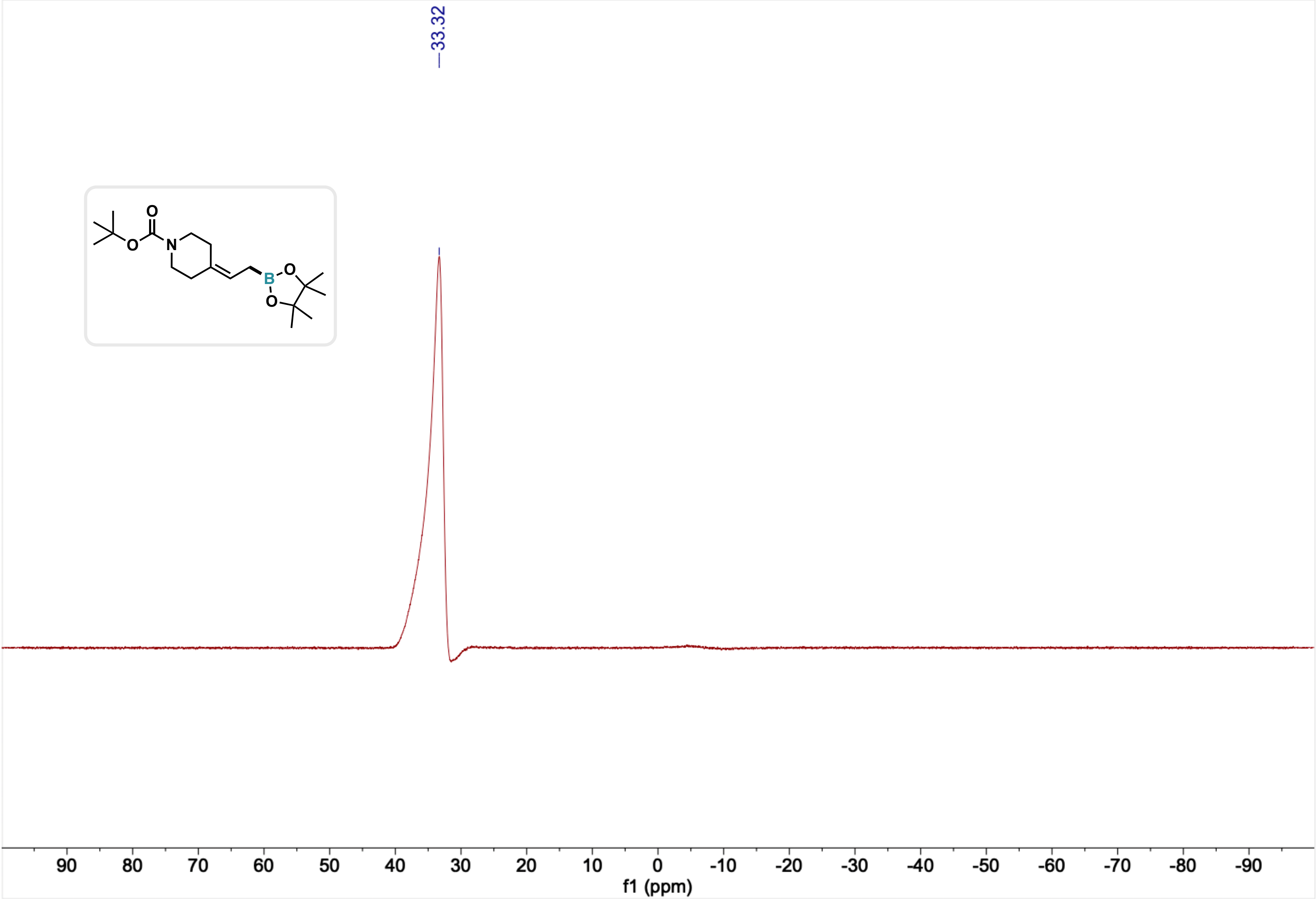

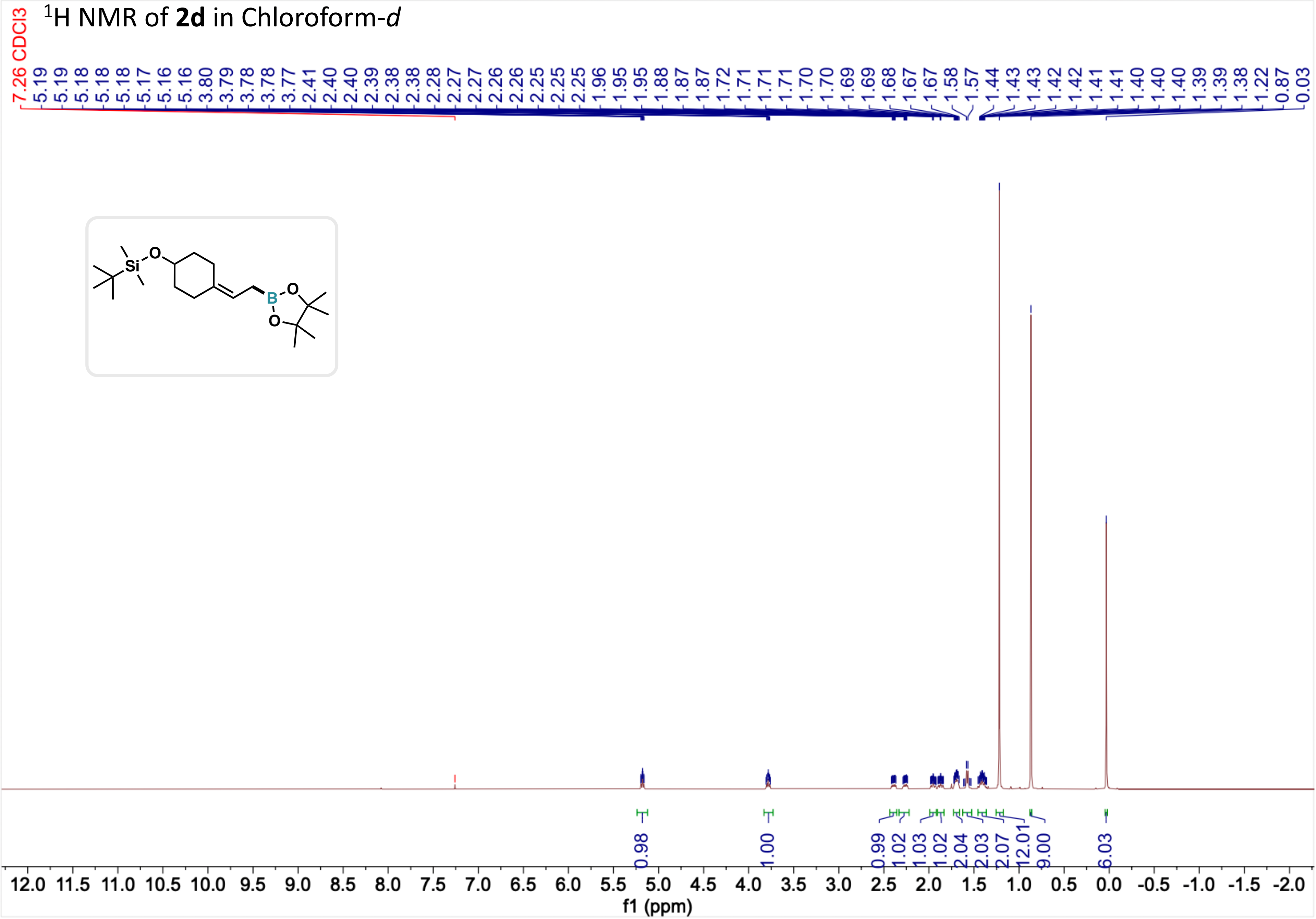

^{1}H NMR of **2d** in Chloroform-*d*
7.26 CDCl3
5.19
5.19
5.18
5.18
5.18
5.17
5.16
5.16
3.80
3.79
3.78
3.78
3.77
2.41
2.40
2.40
2.39
2.38
2.38
2.28
2.27
2.27
2.26
2.26
2.25
2.25
2.25
1.96
1.95
1.95
1.88
1.87
1.87
1.72
1.71
1.71
1.71
1.70
1.70
1.69
1.69
1.68
1.67
1.67
1.58
1.57
1.44
1.43
1.43
1.42
1.42
1.41
1.41
1.40
1.40
1.40
1.39
1.39
1.38
1.22
0.87
0.03
Si
O
B
O
O
0.98
1.00
0.99
1.02
1.03
1.02
2.04
2.03
2.07
12.01
9.00
6.03
12.0 11.5 11.0 10.5 10.0 9.5 9.0 8.5 8.0 7.5 7.0 6.5 6.0 5.5 5.0 4.5 4.0 3.5 3.0 2.5 2.0 1.5 1.0 0.5 0.0 -0.5 -1.0 -1.5 -2.0
f1 (ppm)

^{13}C NMR of **2d** in Chloroform-*d*

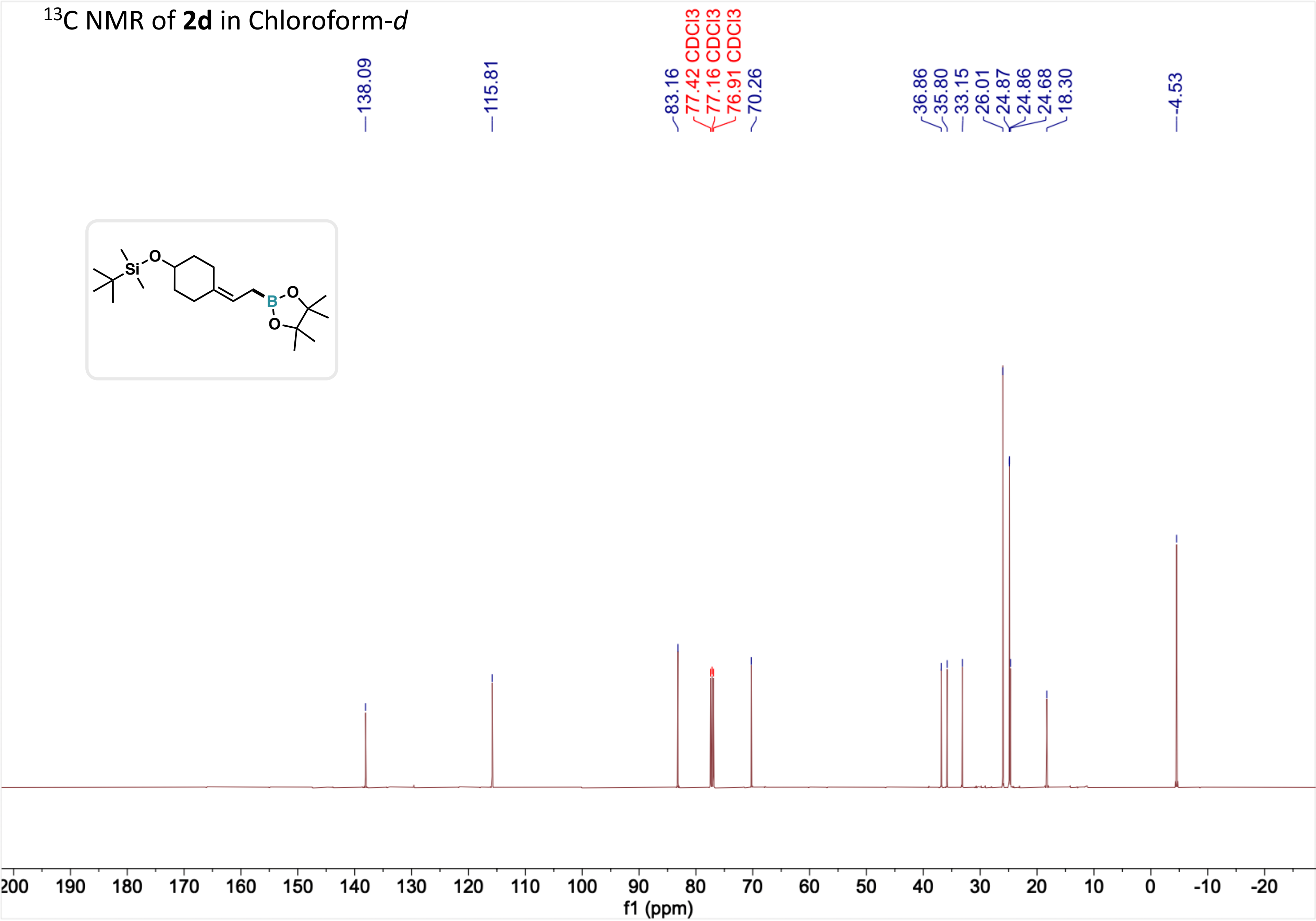

^{11}B NMR of **2d** in Chloroform-*d*

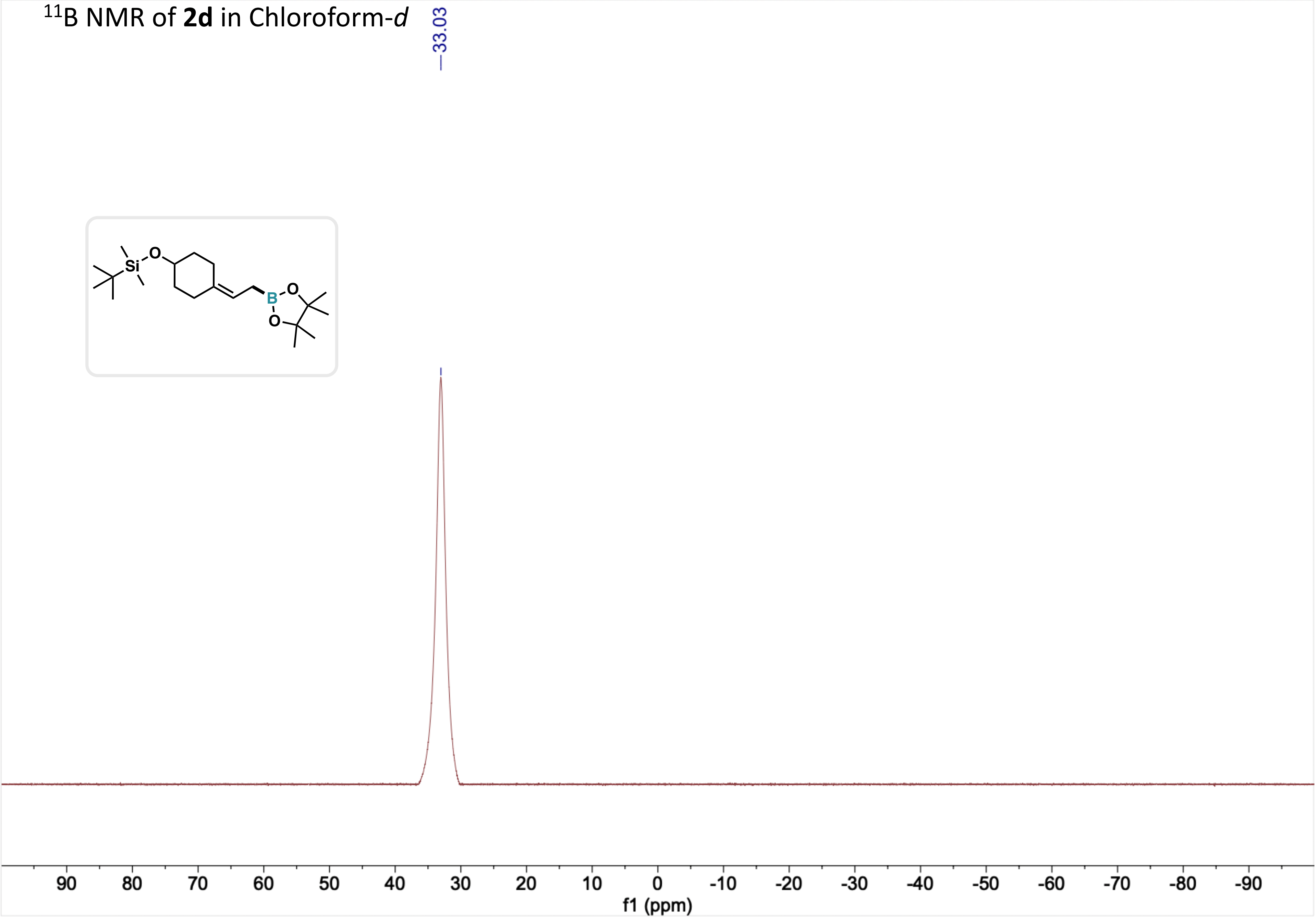

^{1}H NMR of **2e** in Chloroform-*d*

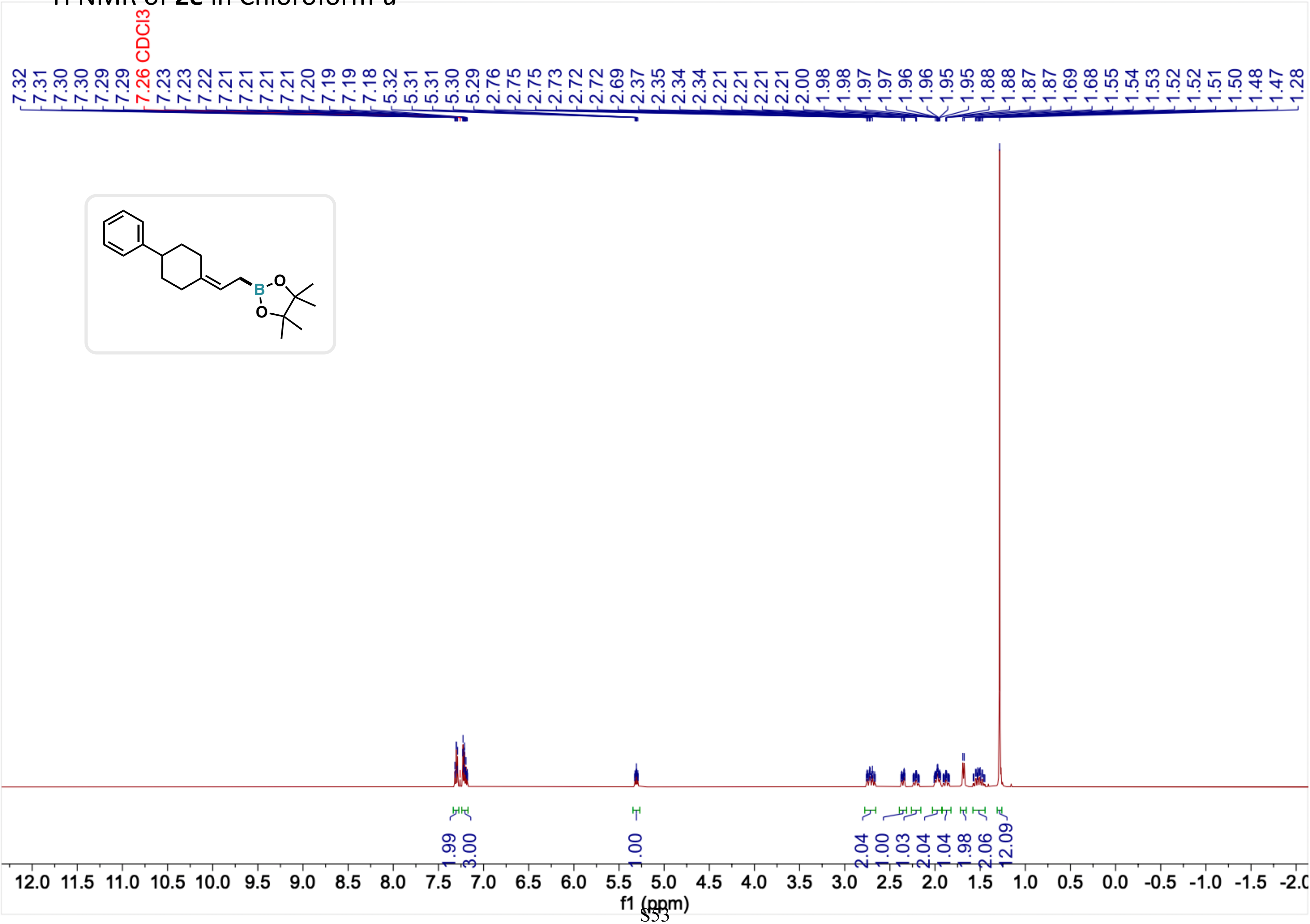

^{13}C NMR of **2e** in Chloroform-*d*

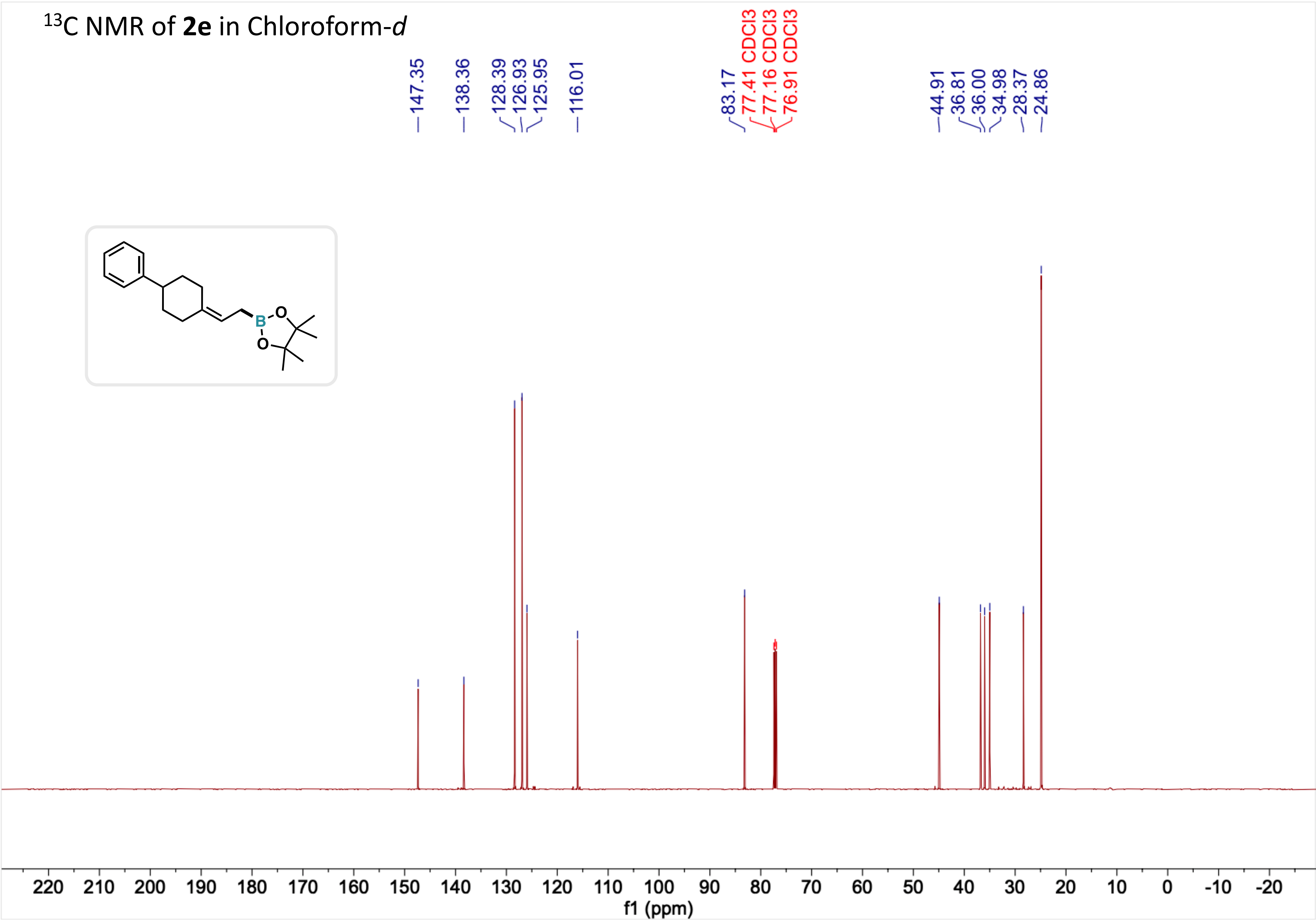

^{11}B NMR of **2e** in Chloroform-*d*

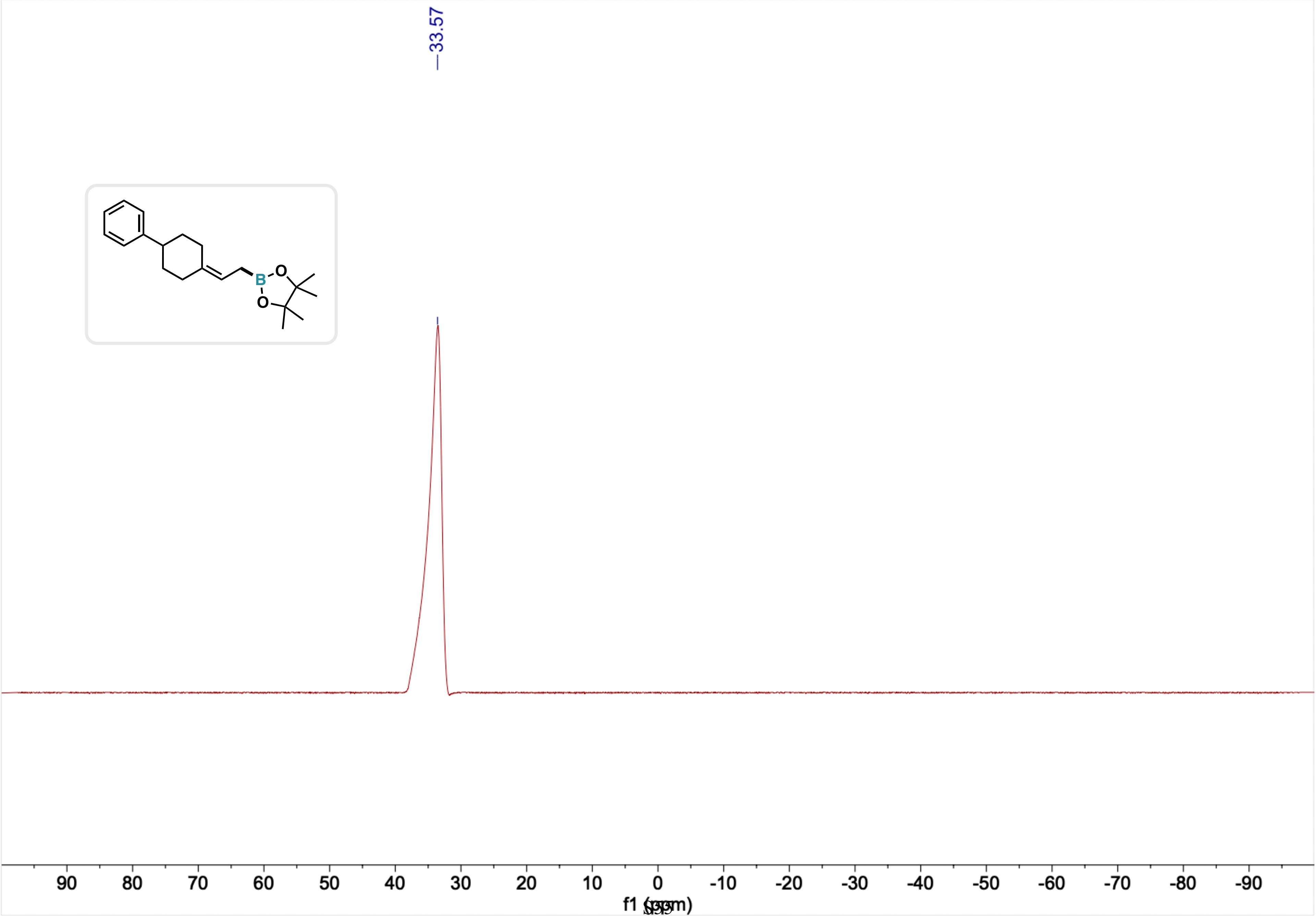

^{1}H NMR of **2f** in Chloroform-*d*

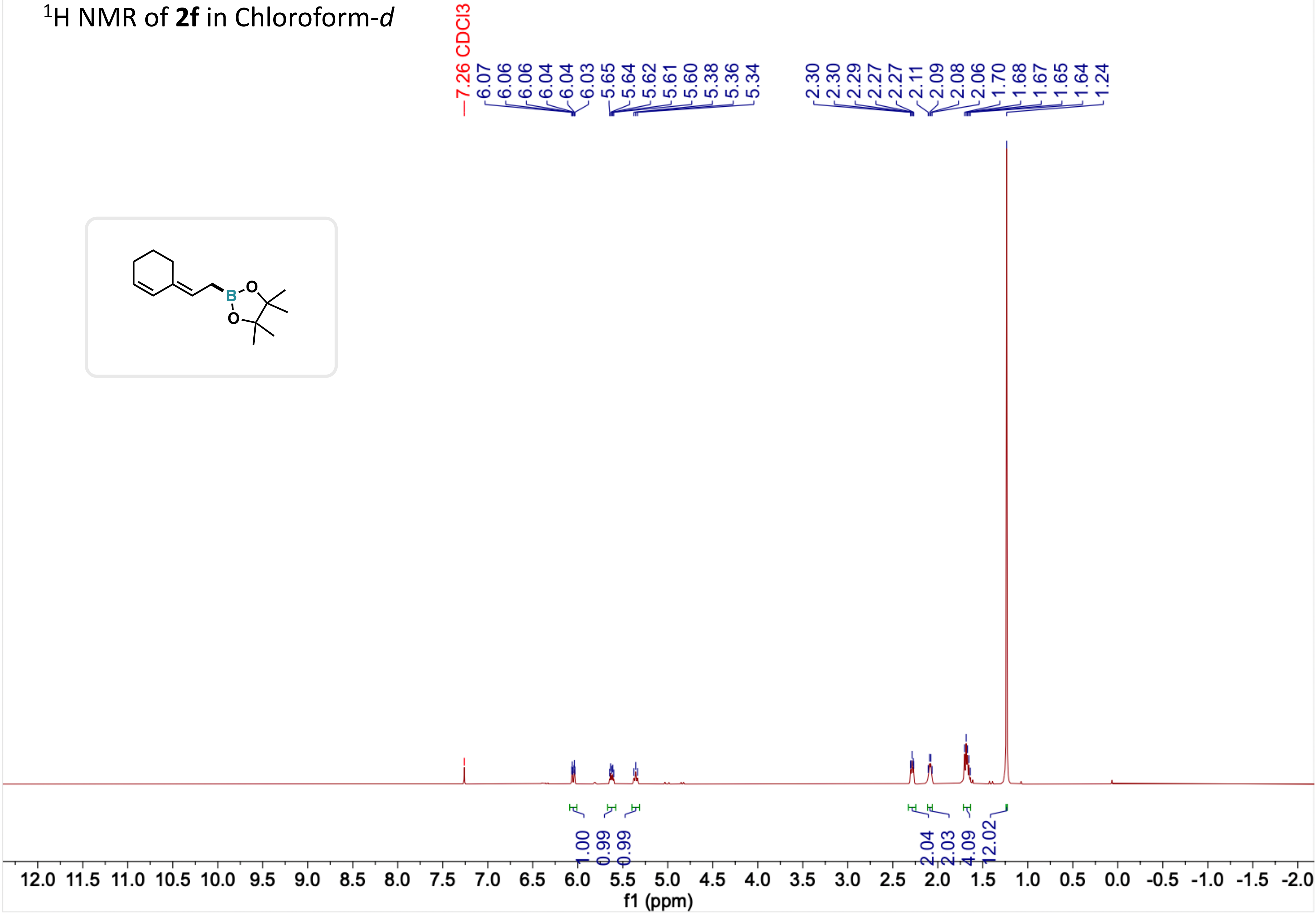

^{13}C NMR of **2f** in Chloroform-*d*

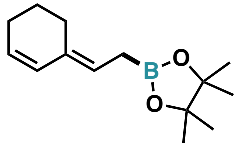

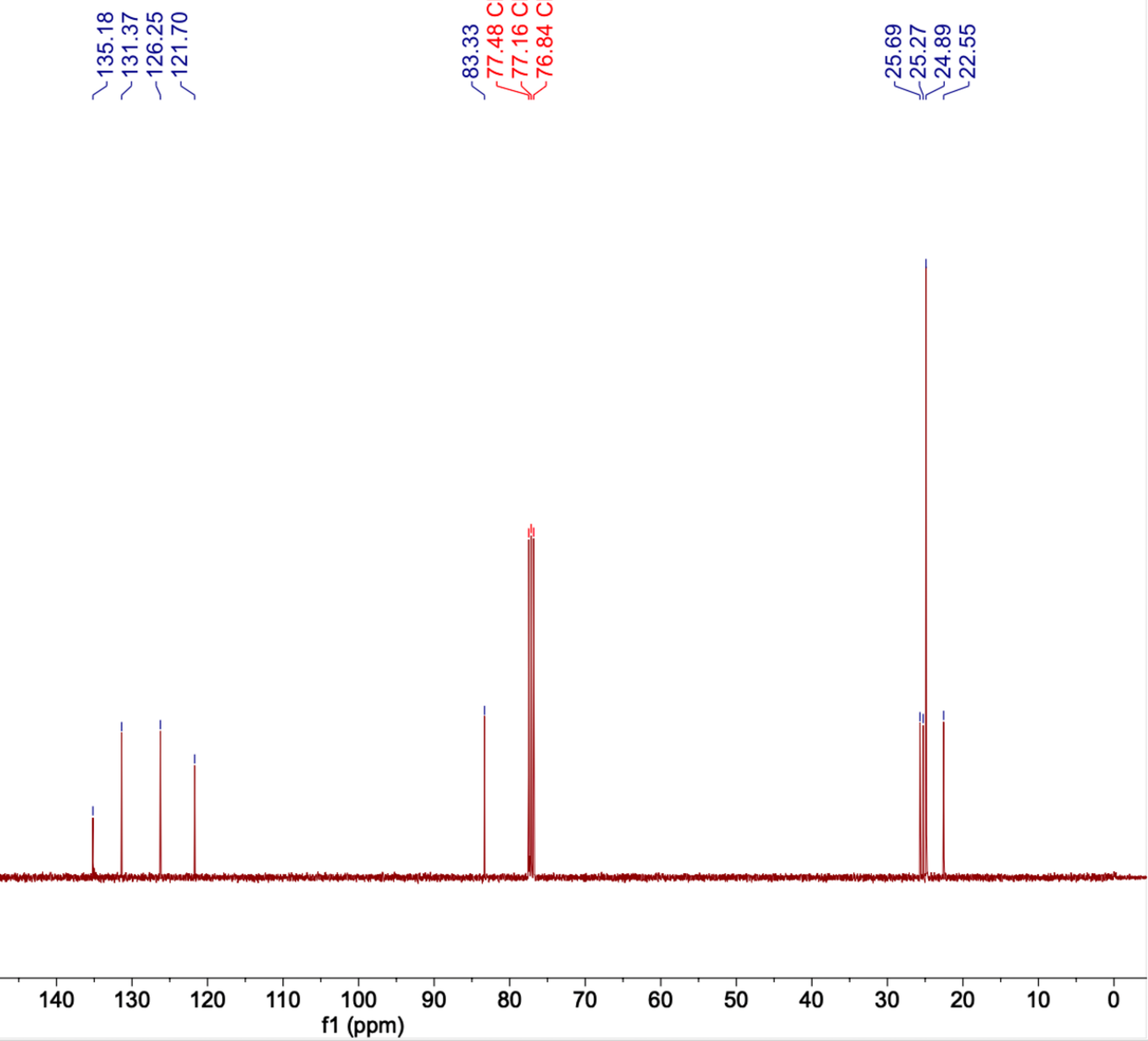

^{11}B NMR of **2f** in Chloroform-*d*

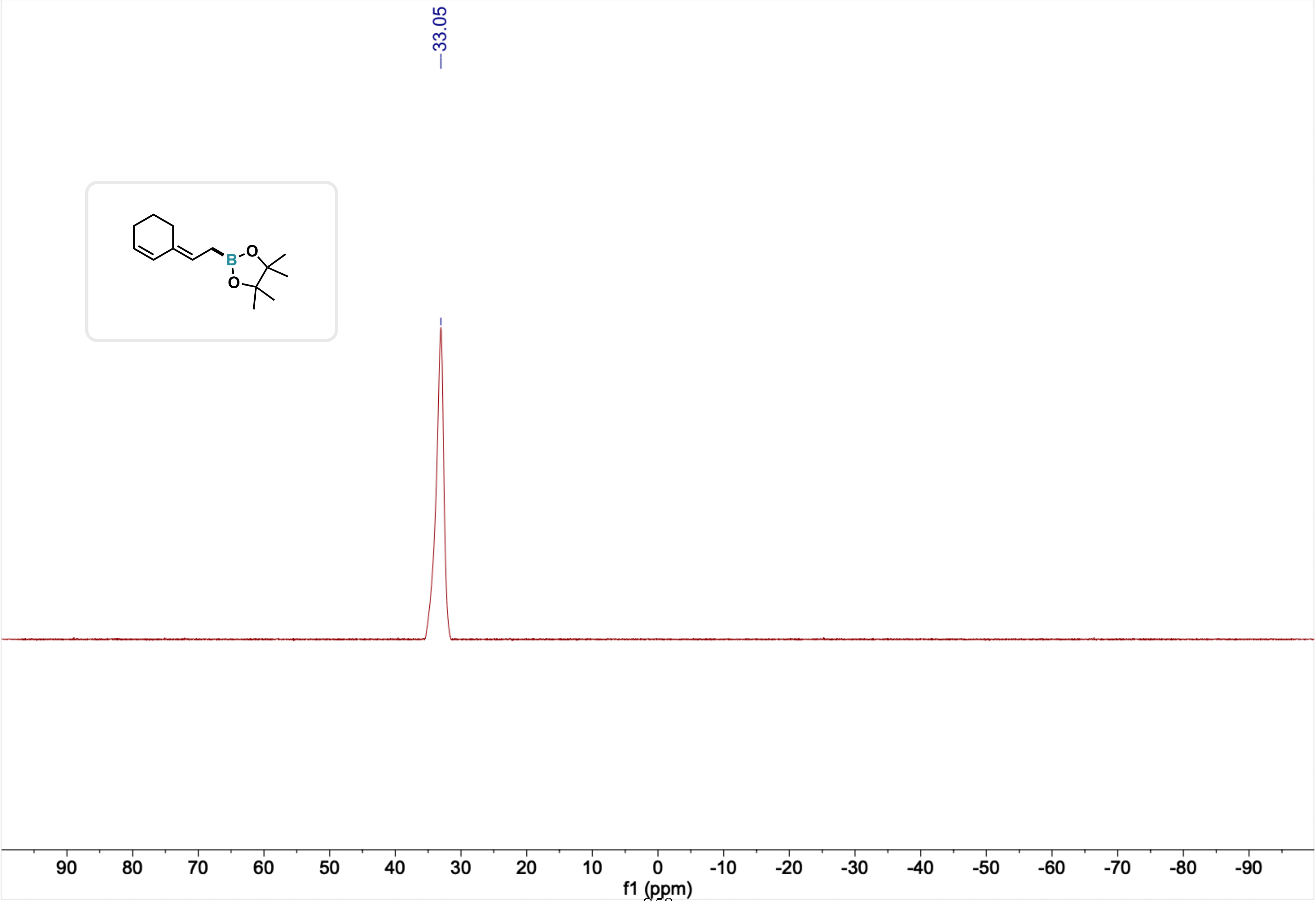

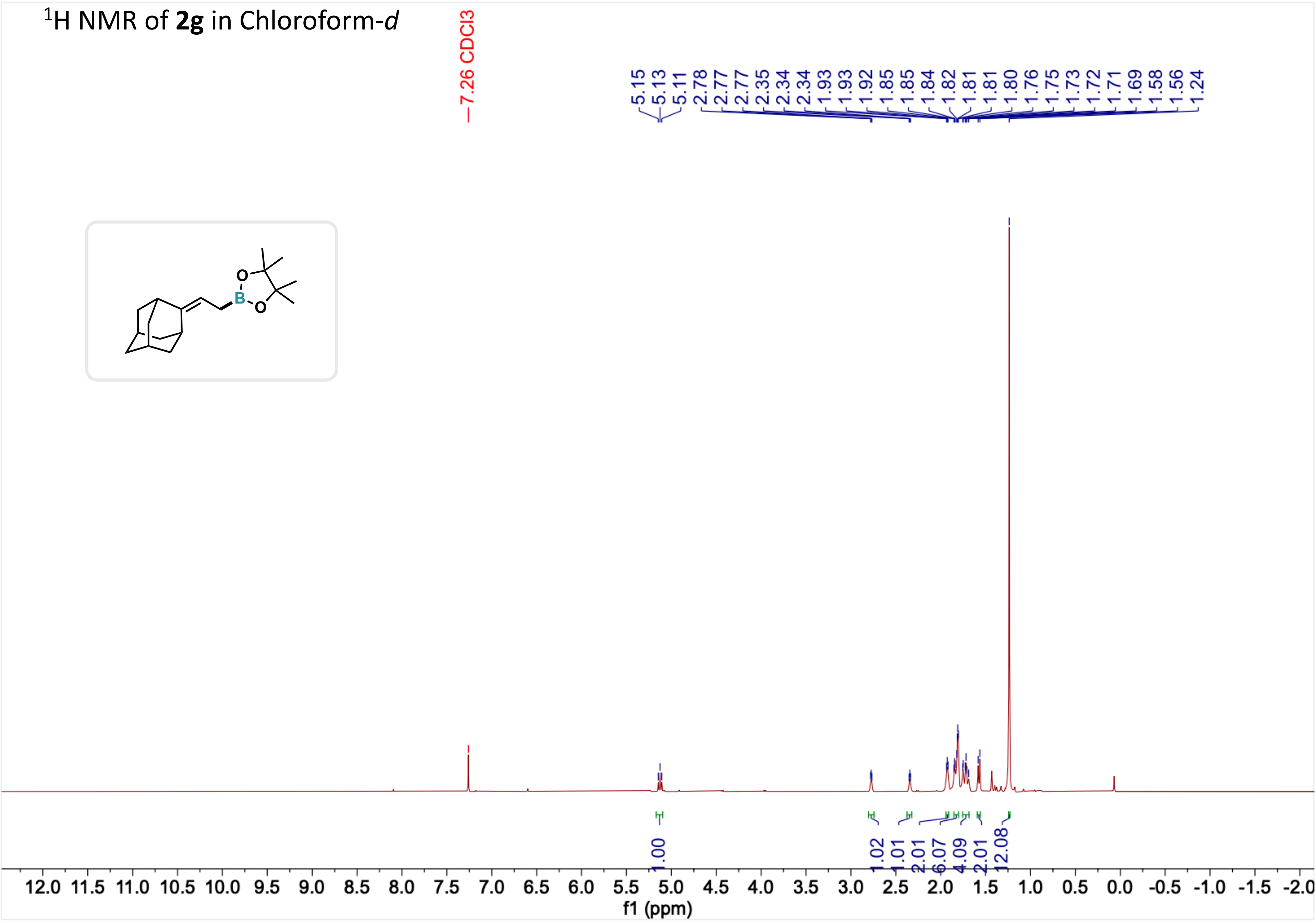

^{1}H NMR of 2g in Chloroform-d
7.26 CDCl3
5.15
5.13
5.11
2.78
2.77
2.77
2.35
2.34
2.34
1.93
1.93
1.92
1.85
1.85
1.84
1.82
1.81
1.81
1.80
1.76
1.75
1.73
1.72
1.71
1.69
1.58
1.56
1.24
O
B
O
1.00
1.02
1.01
2.01
6.07
4.09
2.01
12.08
12.0 11.5 11.0 10.5 10.0 9.5 9.0 8.5 8.0 7.5 7.0 6.5 6.0 5.5 5.0 4.5 4.0 3.5 3.0 2.5 2.0 1.5 1.0 0.5 0.0 -0.5 -1.0 -1.5 -2.0
f1 (ppm)

^{13}C NMR of **2g** in Chloroform-*d*

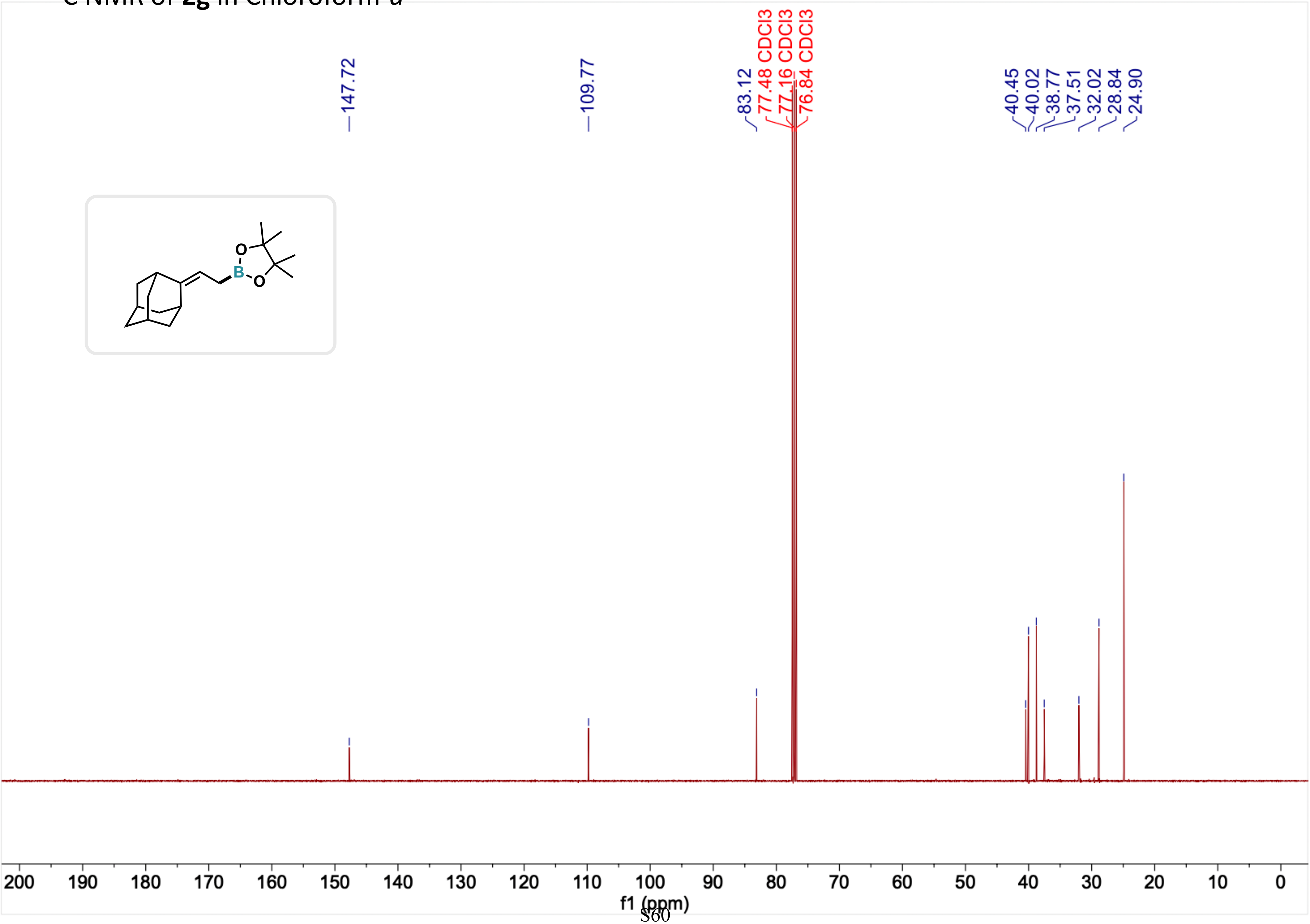

^{11}B NMR of **2g** in Chloroform-*d*

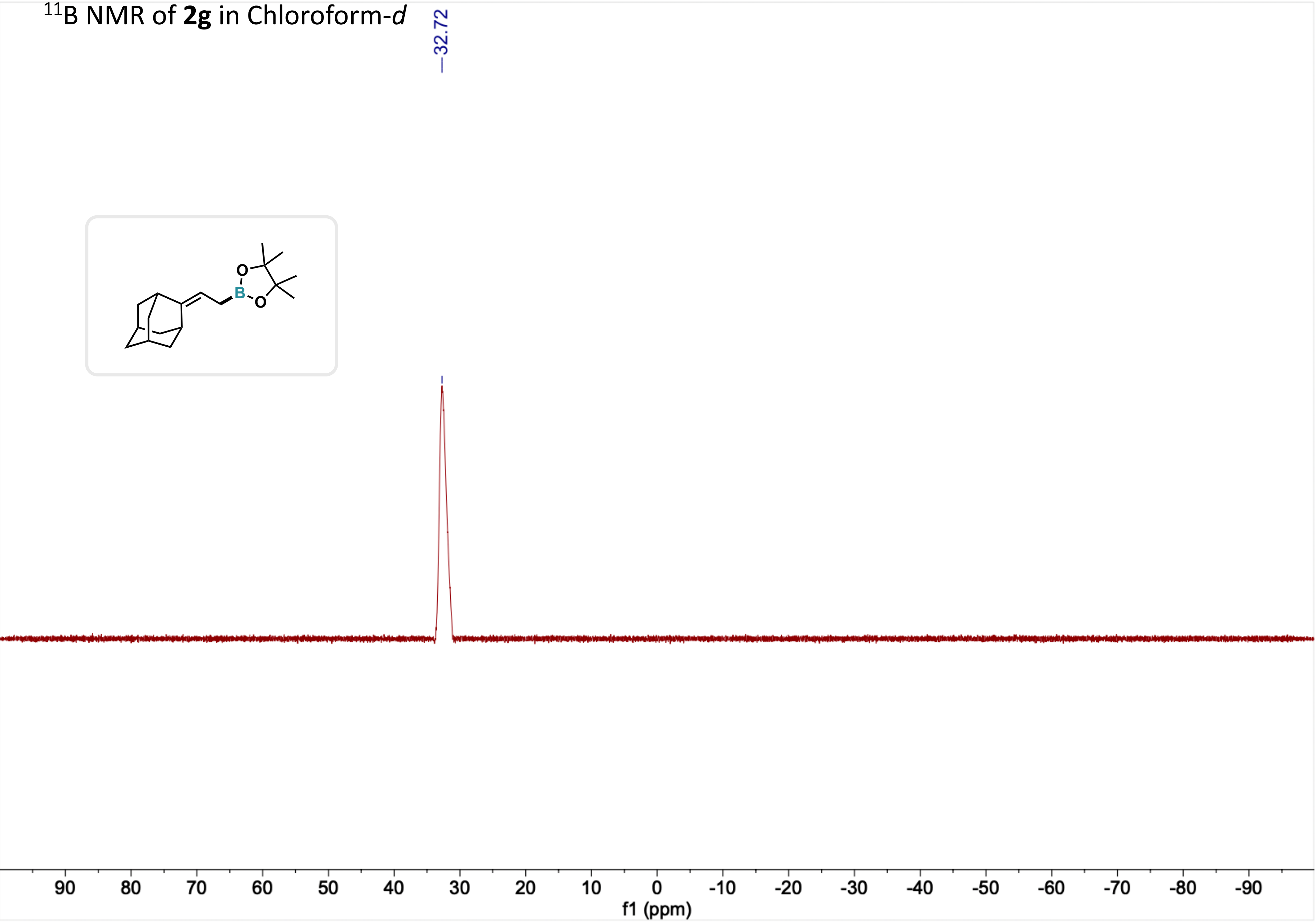

^{1}H NMR of **2h** in Chloroform-*d*

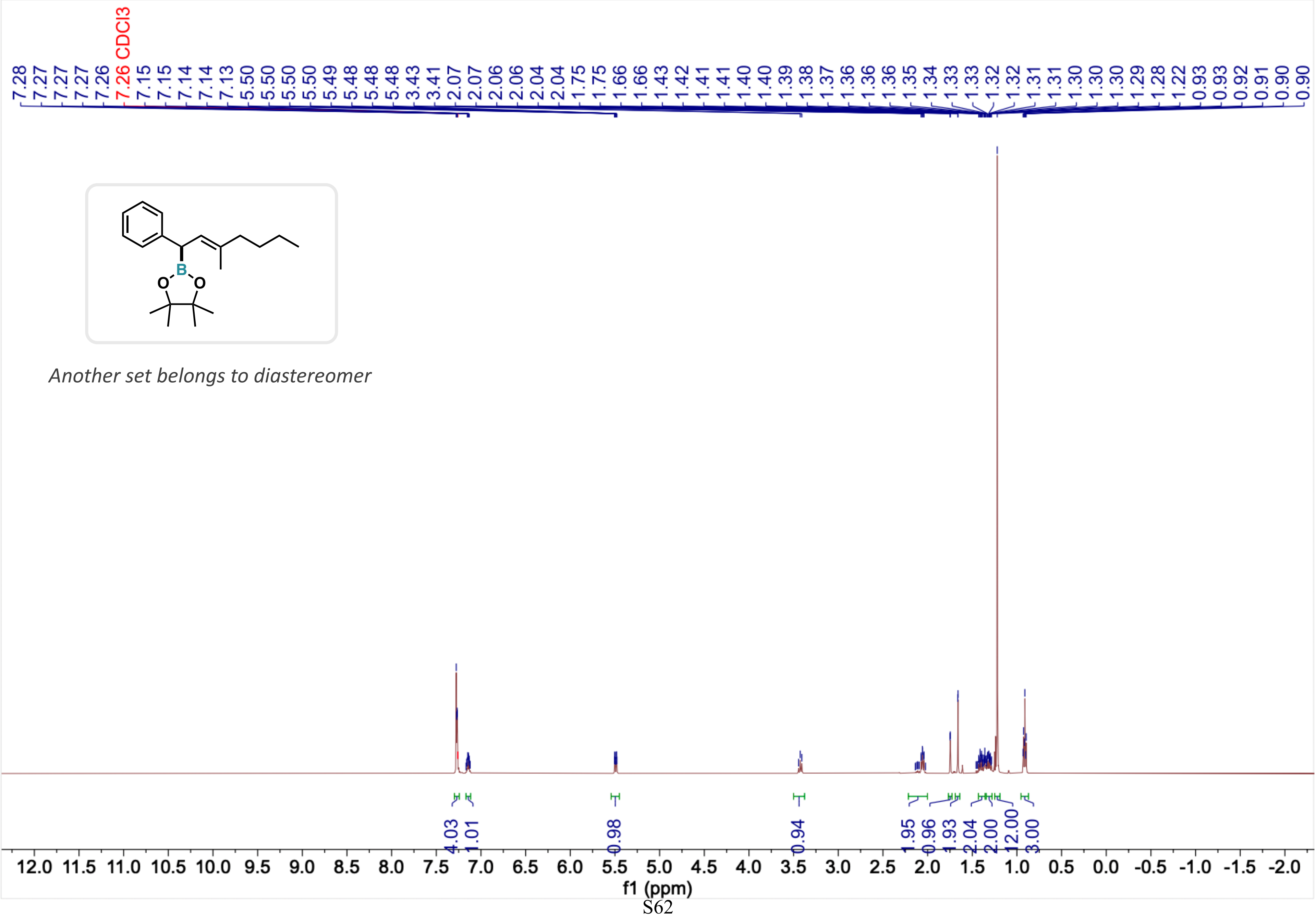

^{13}C NMR of **2h** in Chloroform-*d*

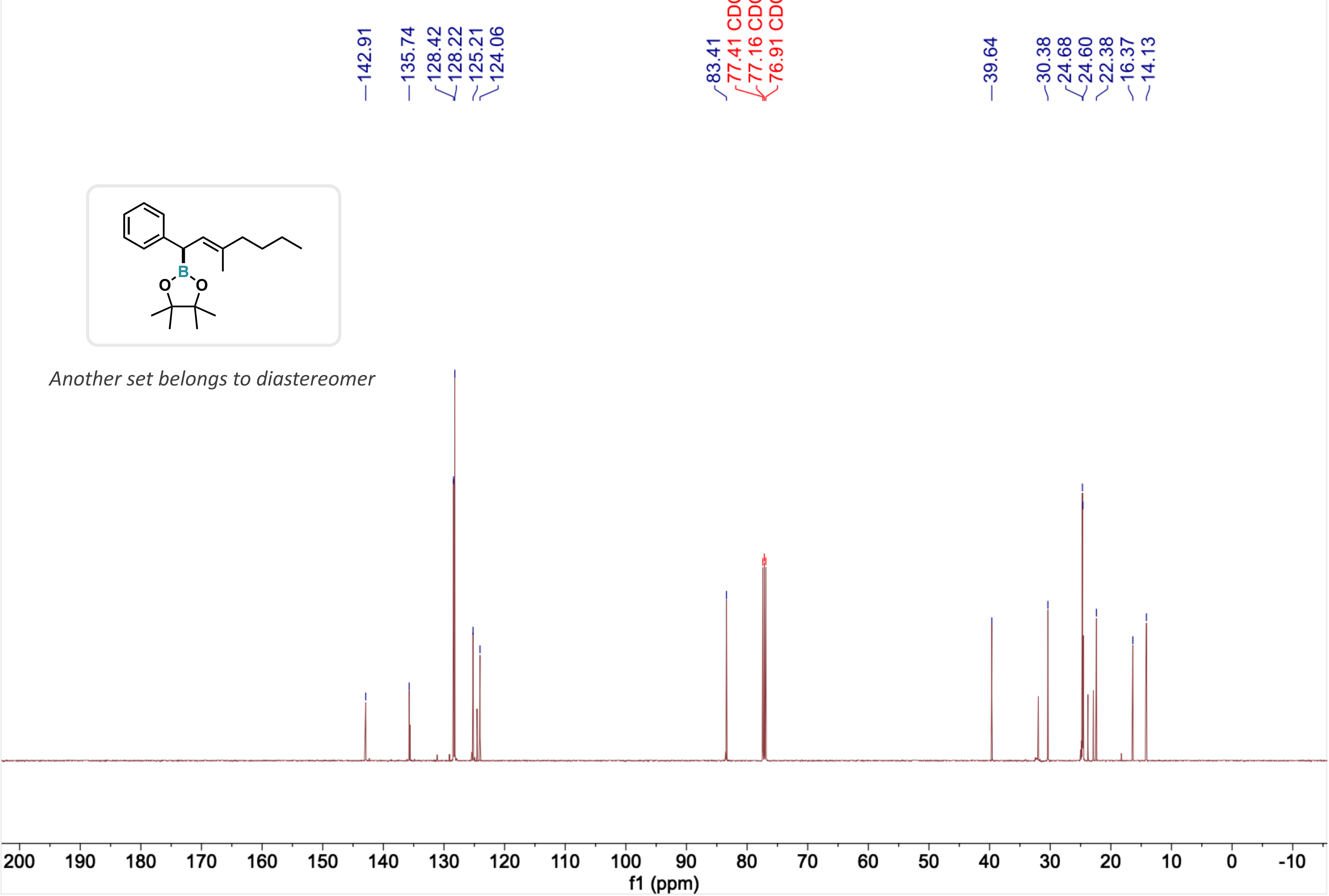

^{11}B NMR of **2h** in Chloroform-*d*

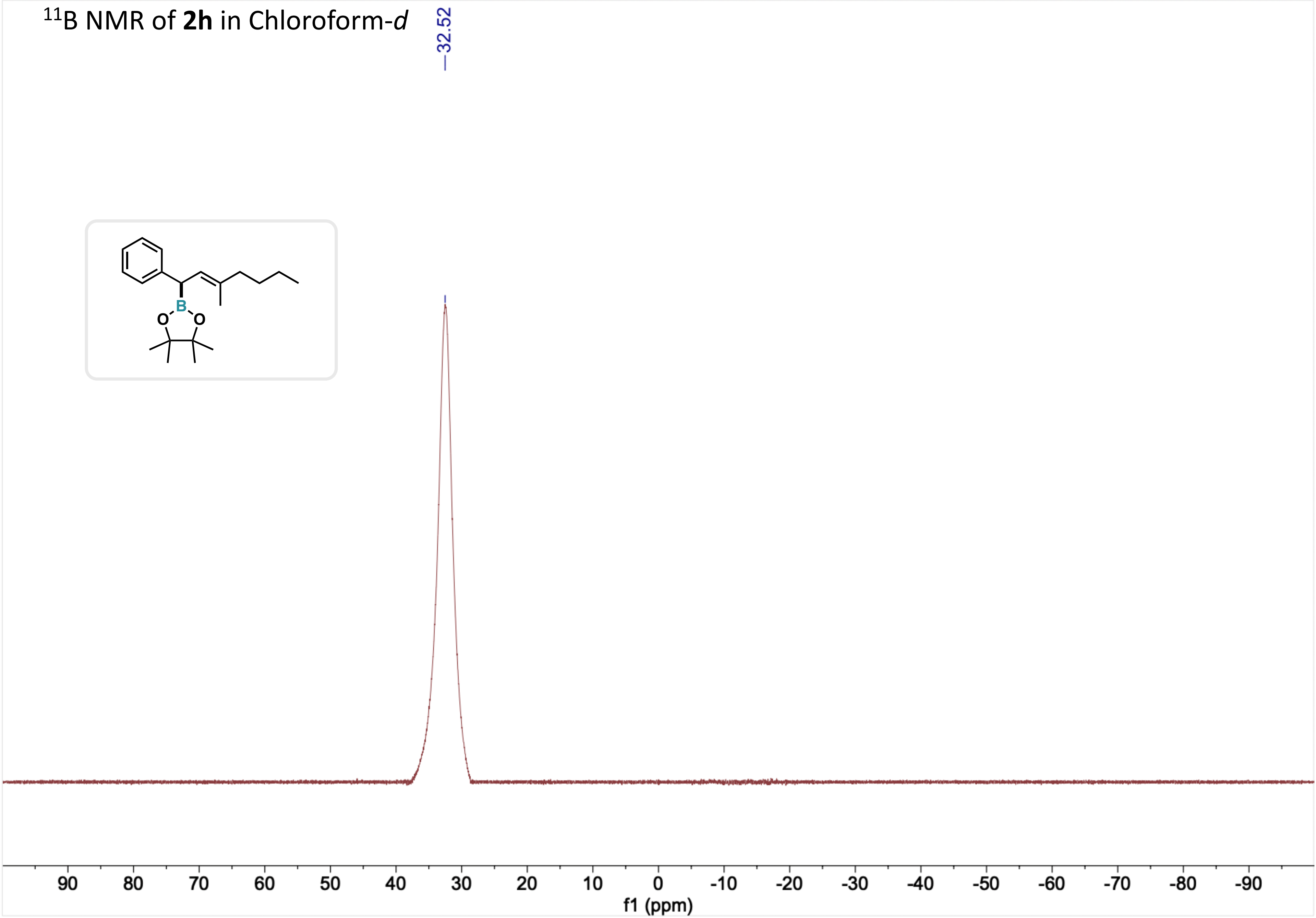

^1H NMR of **2i** in Chloroform-*d*

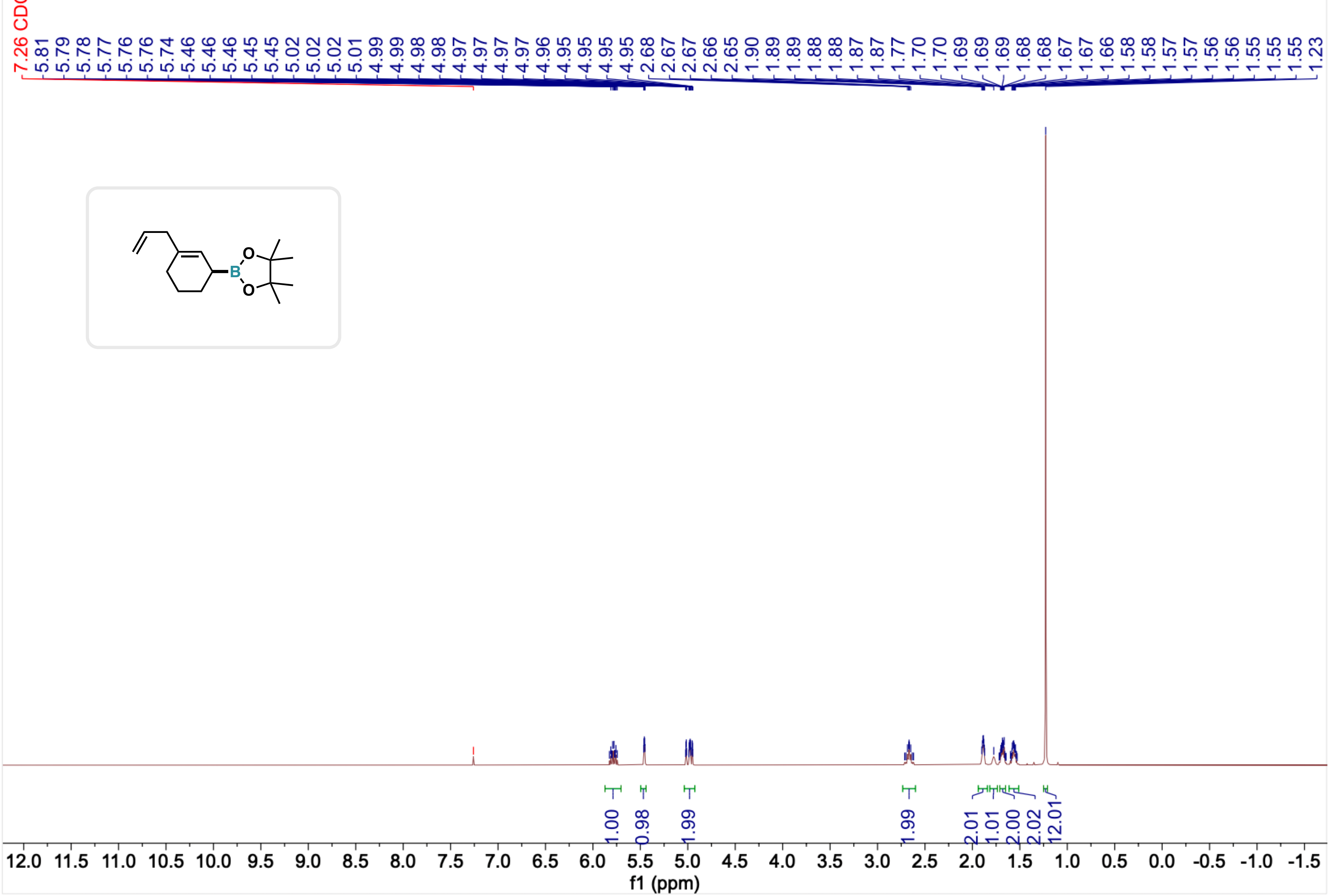

¹³C NMR of **2i** in Chloroform-*d*

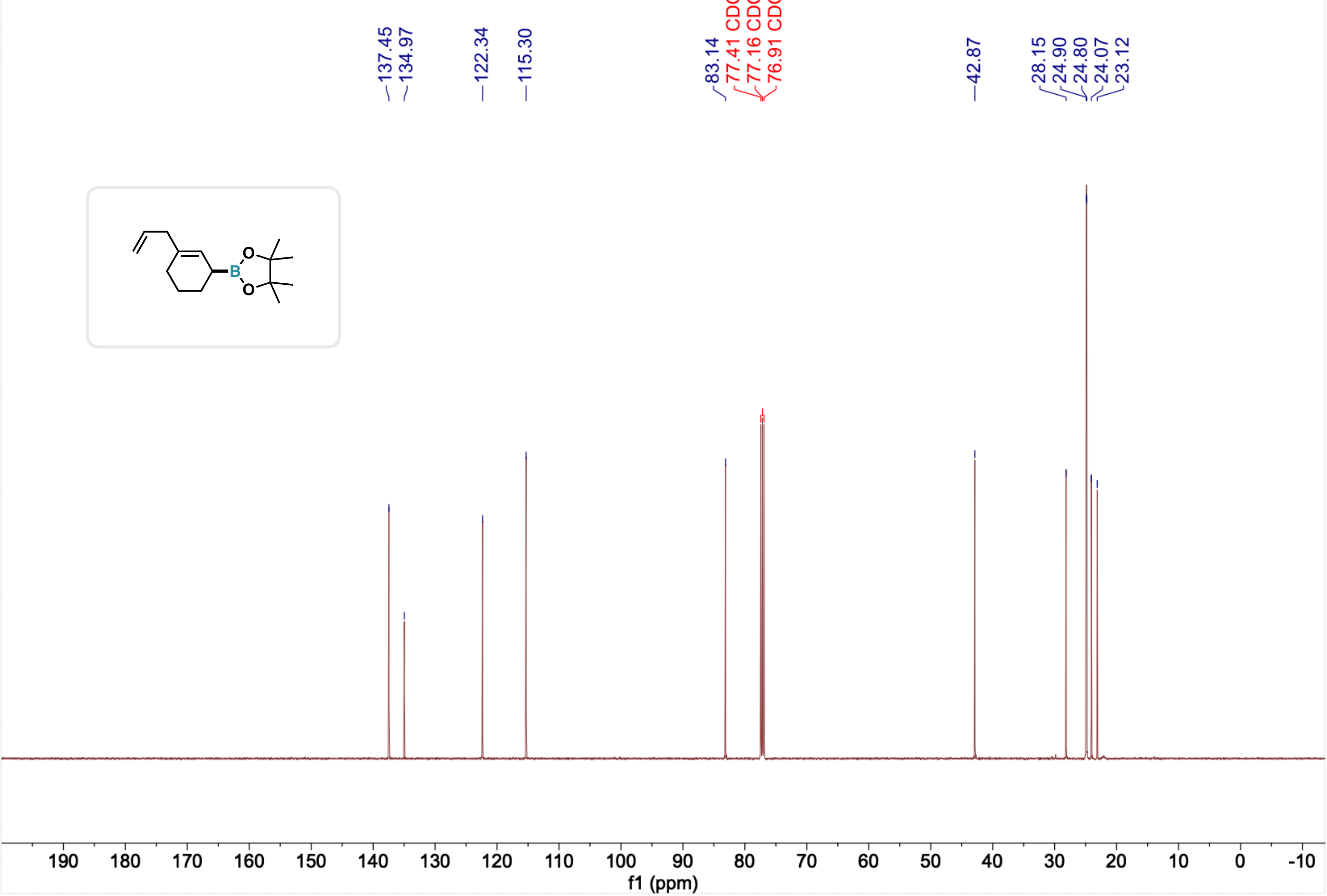

^{11}B NMR of **2i** in Chloroform-*d*

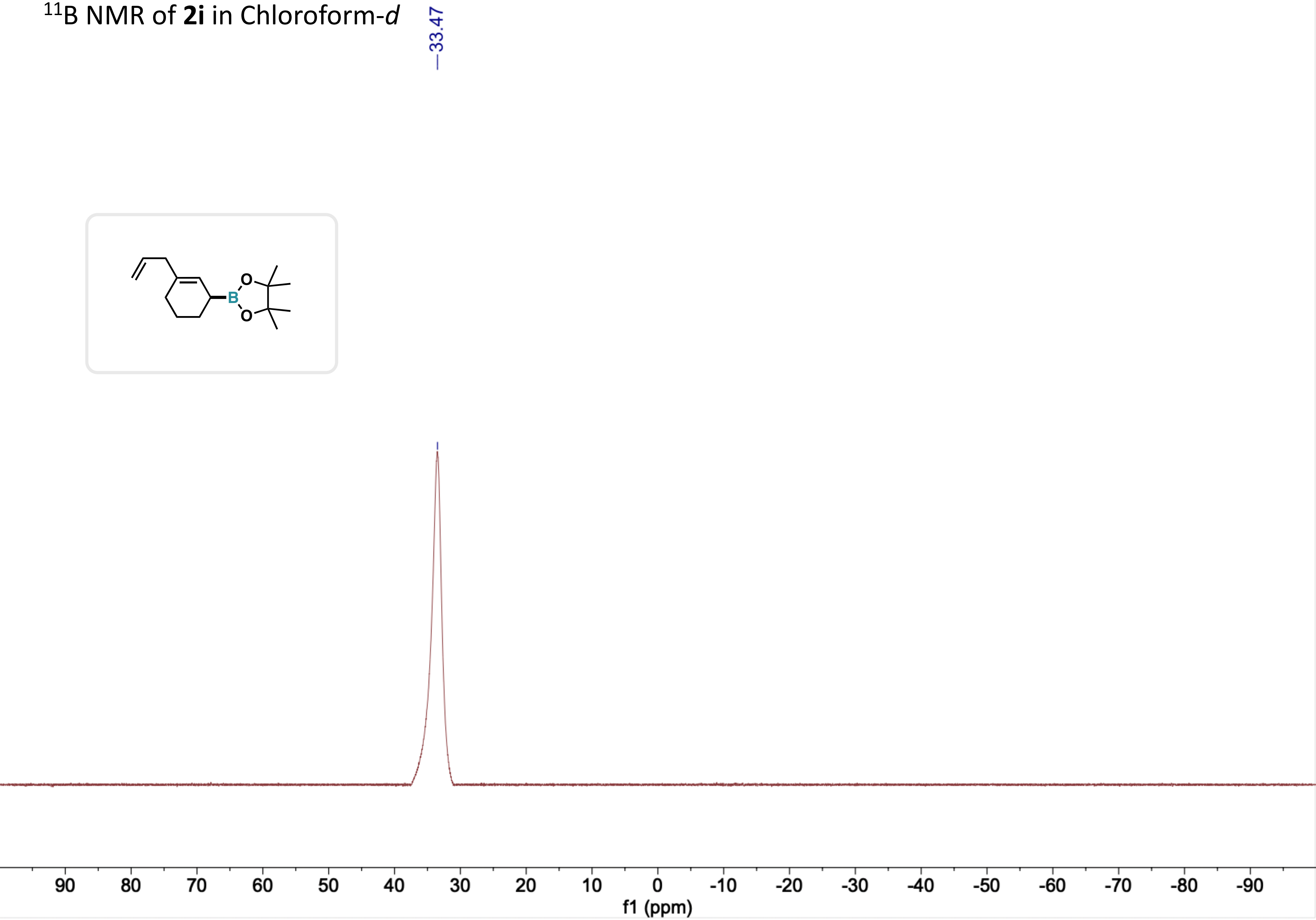

^{1}H NMR of **2j** in Chloroform-*d*

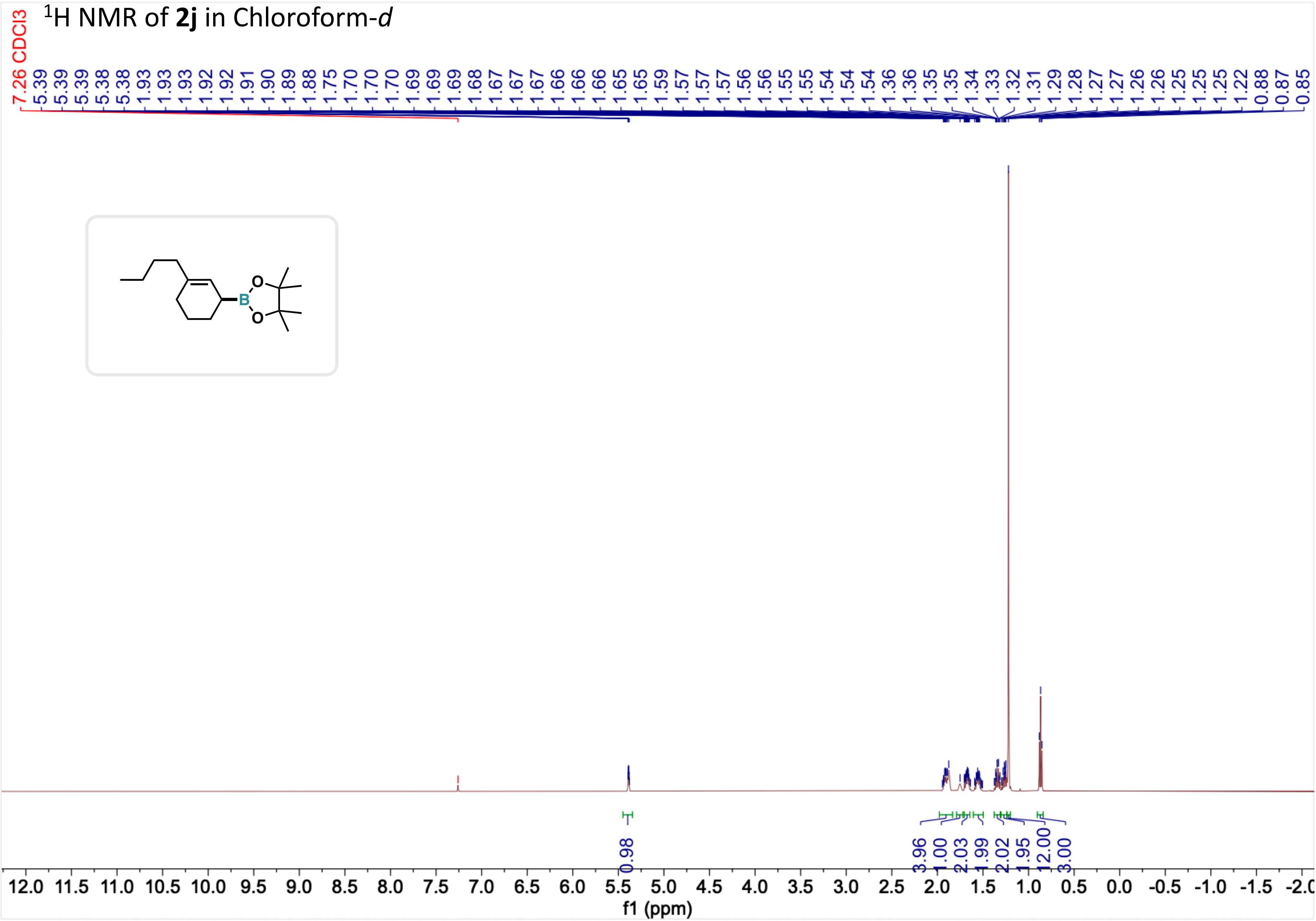

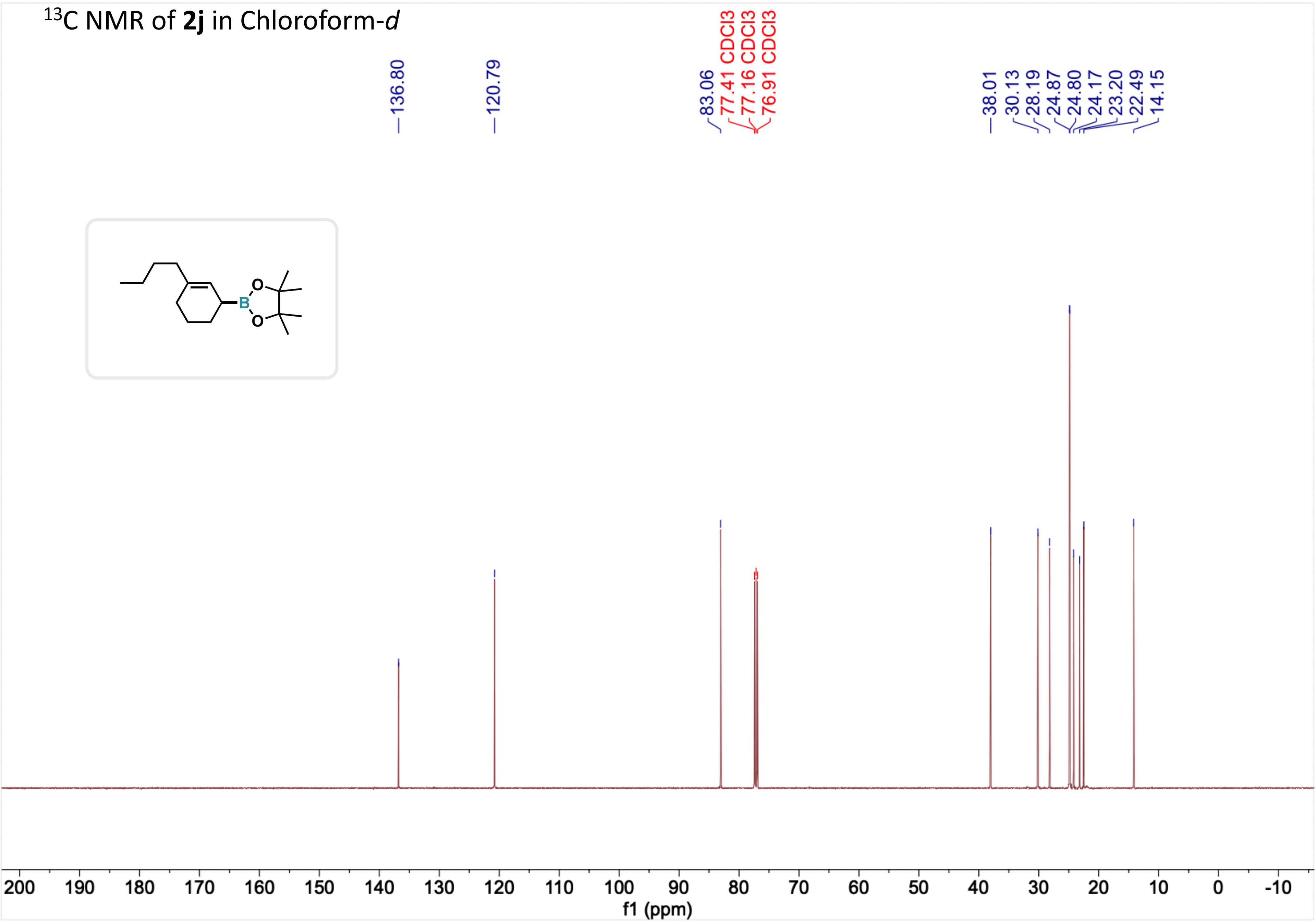

13C NMR of 2j in Chloroform-d
136.80
120.79
83.06
77.41 CDCl3
77.16 CDCl3
76.91 CDCl3
38.01
30.13
28.19
24.87
24.80
24.17
23.20
22.49
14.15
B
O
O
200 190 180 170 160 150 140 130 120 110 100 90 80 70 60 50 40 30 20 10 0 -10
f1 (ppm)

^{11}B NMR of **2j** in Chloroform-*d*

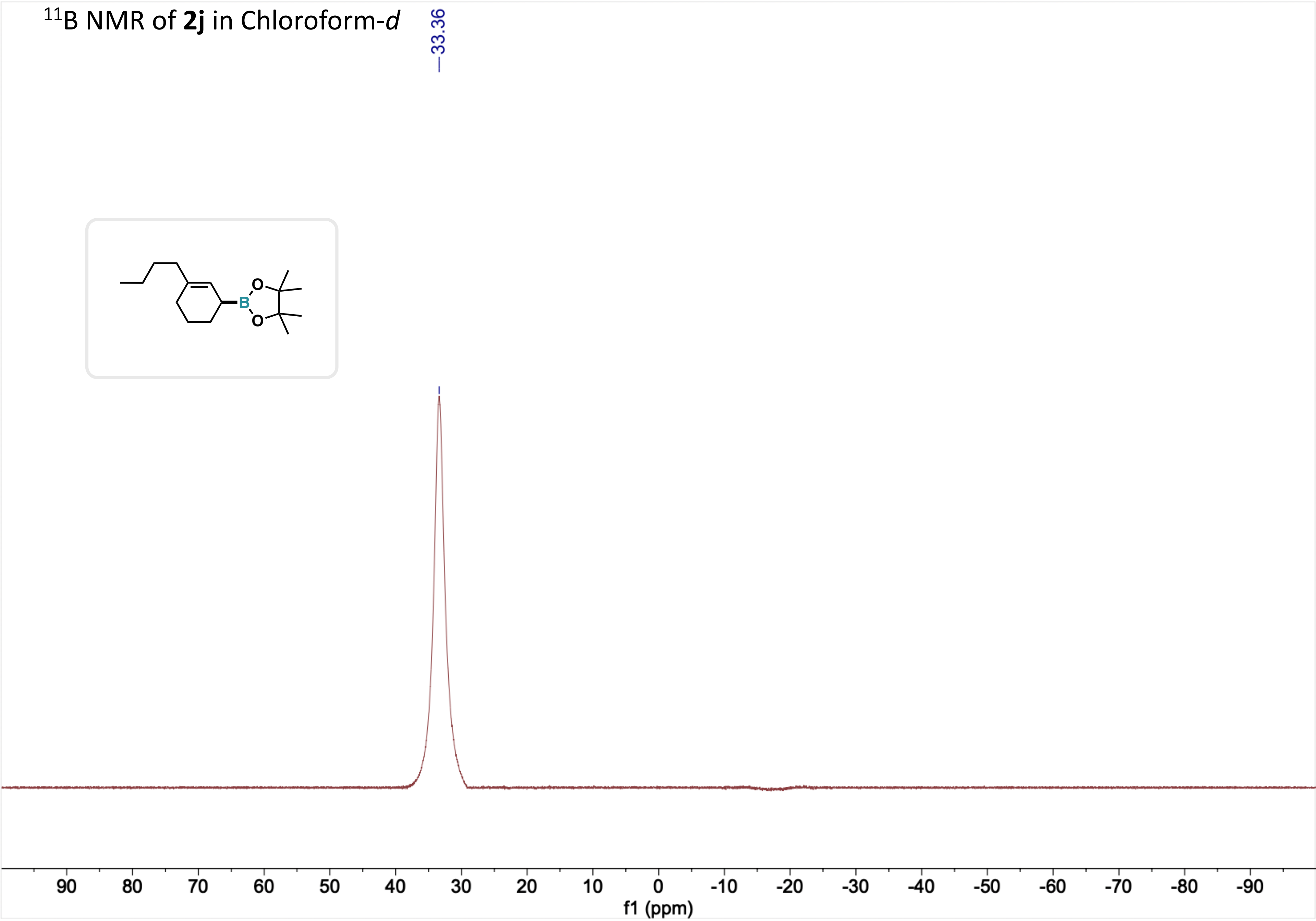

^{1}H NMR of **2k** in Chloroform-*d*

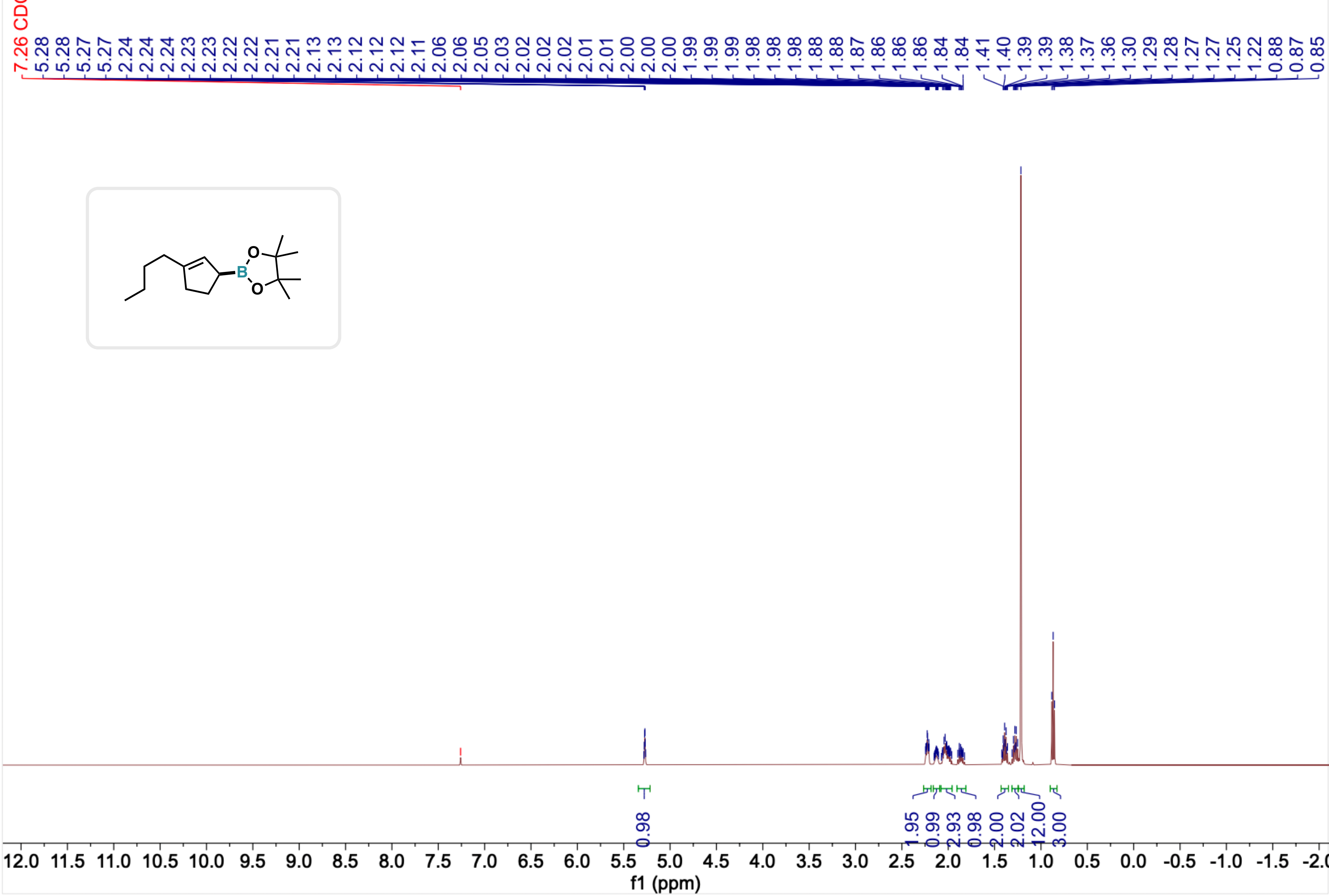

^{13}C NMR of **2k** in Chloroform-*d*

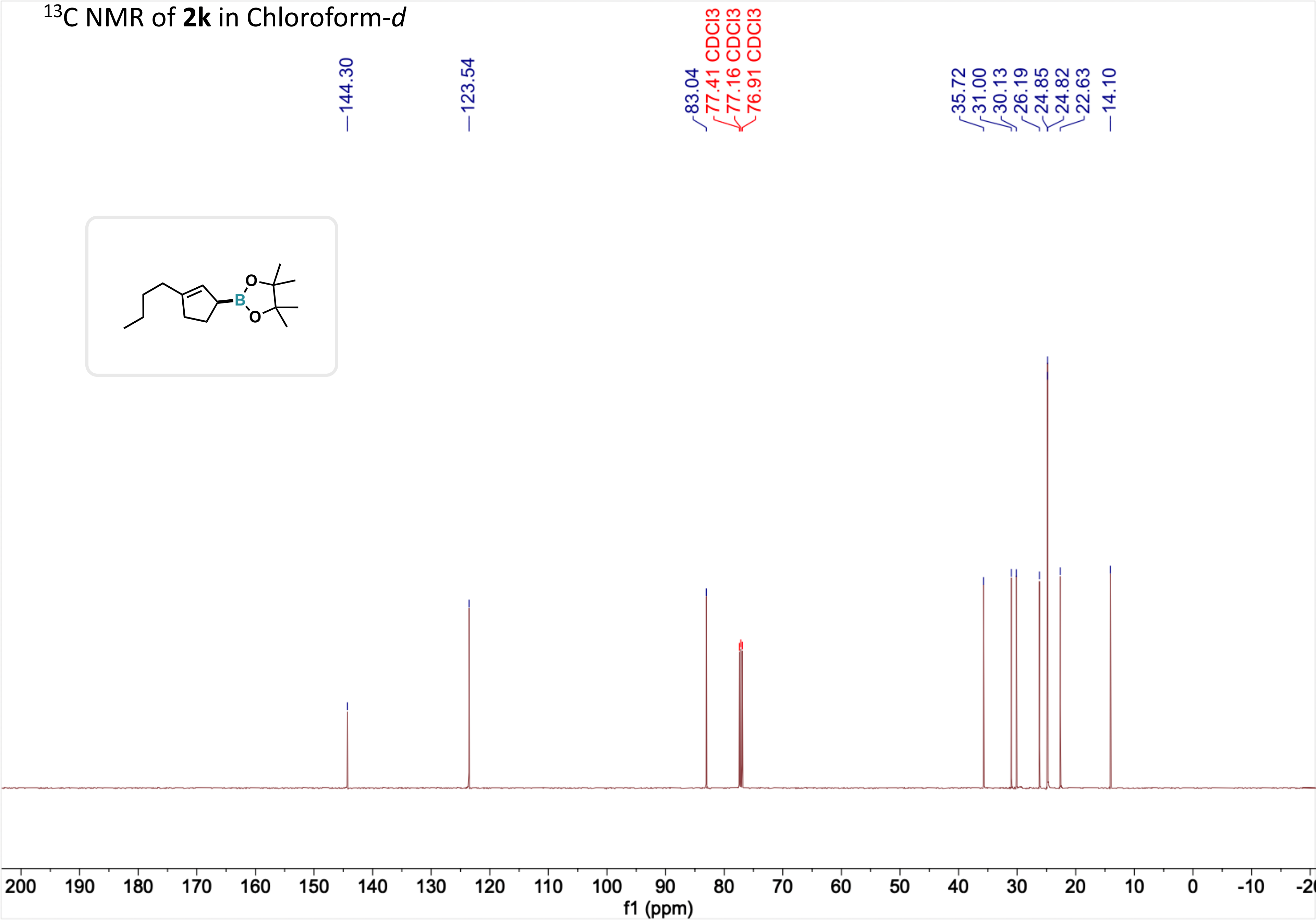

^{11}B NMR of **2k** in Chloroform-*d*

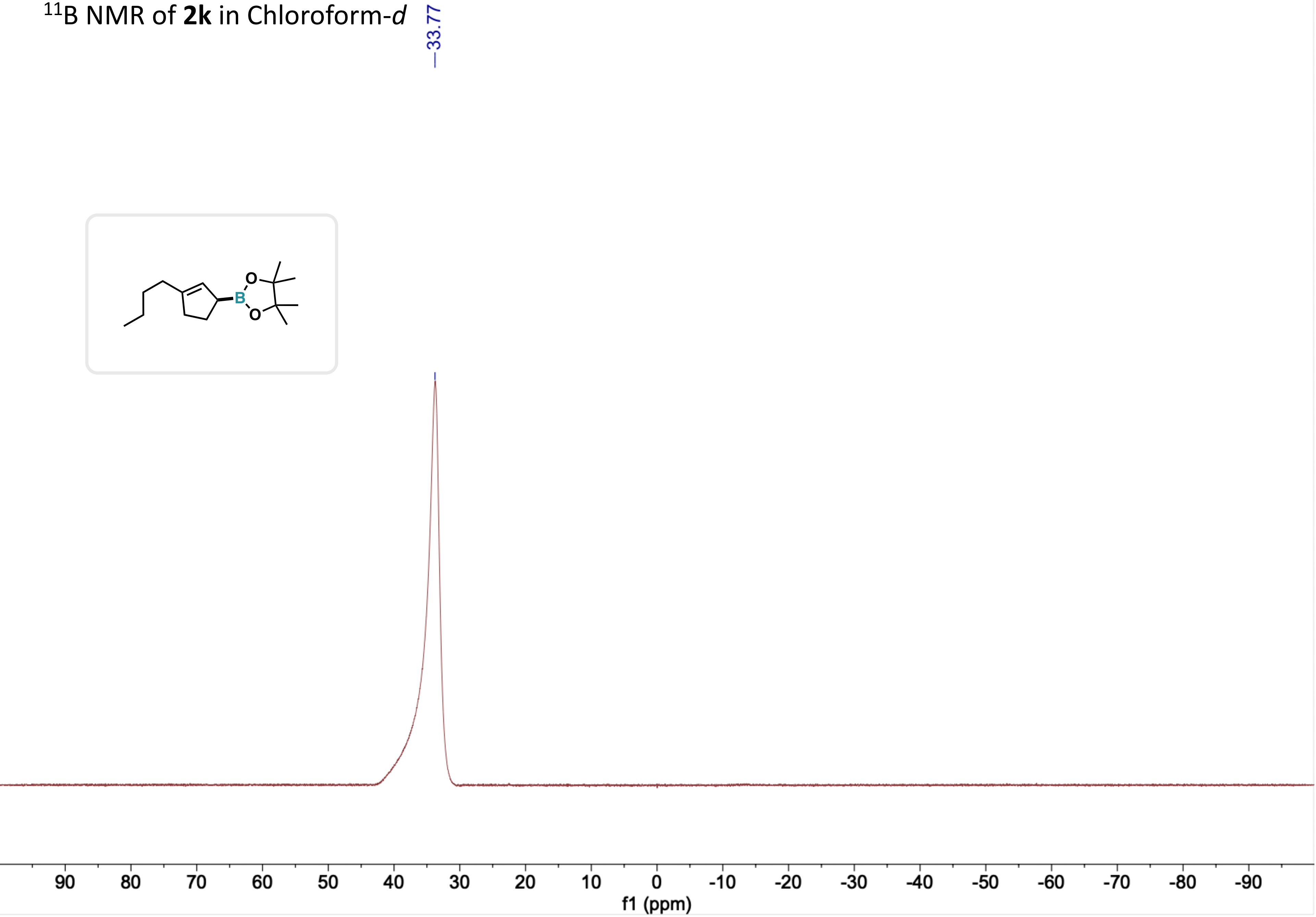

^{1}H NMR of **2l** in Chloroform-*d*

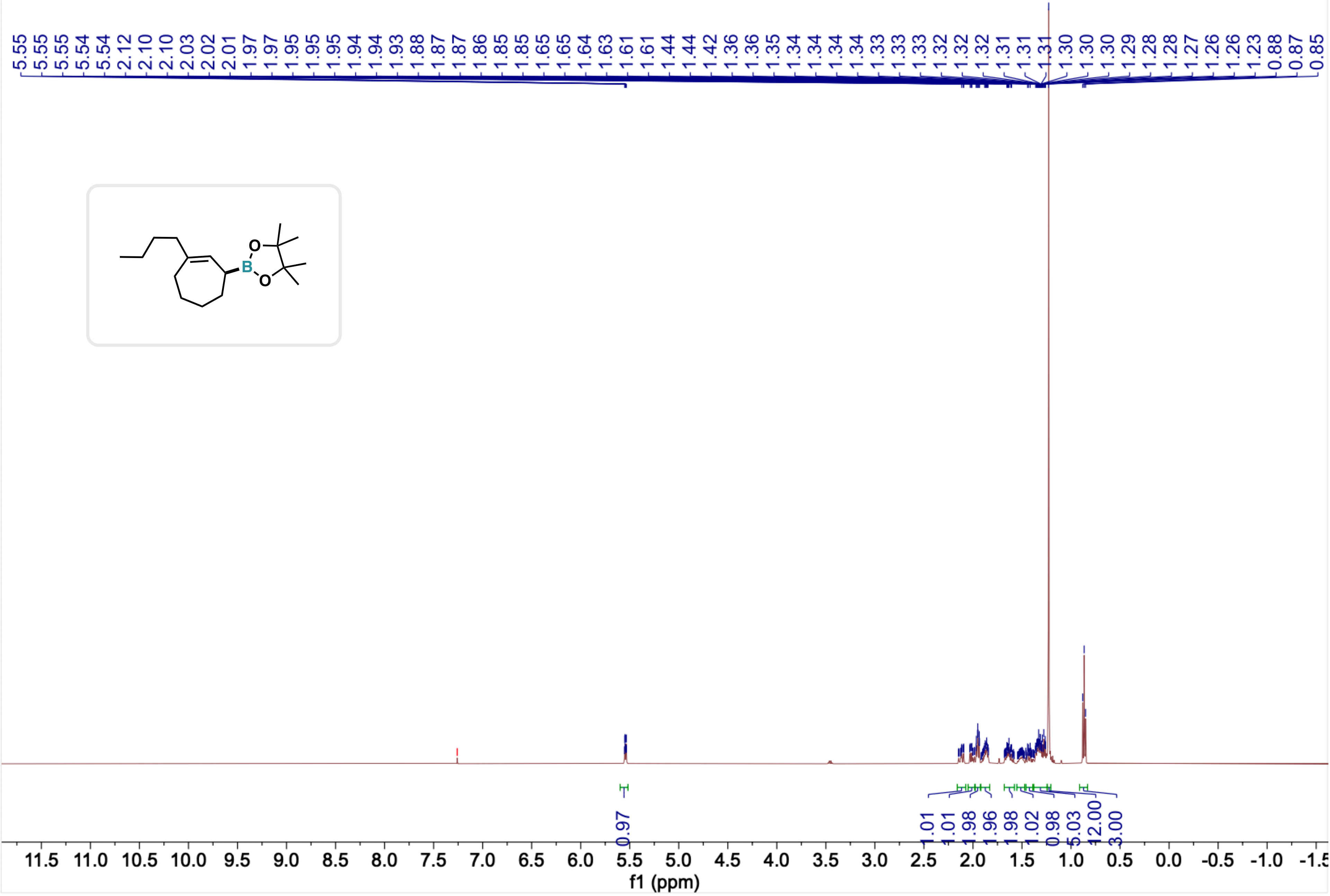

^{13}C NMR of **2l** in Chloroform-*d*

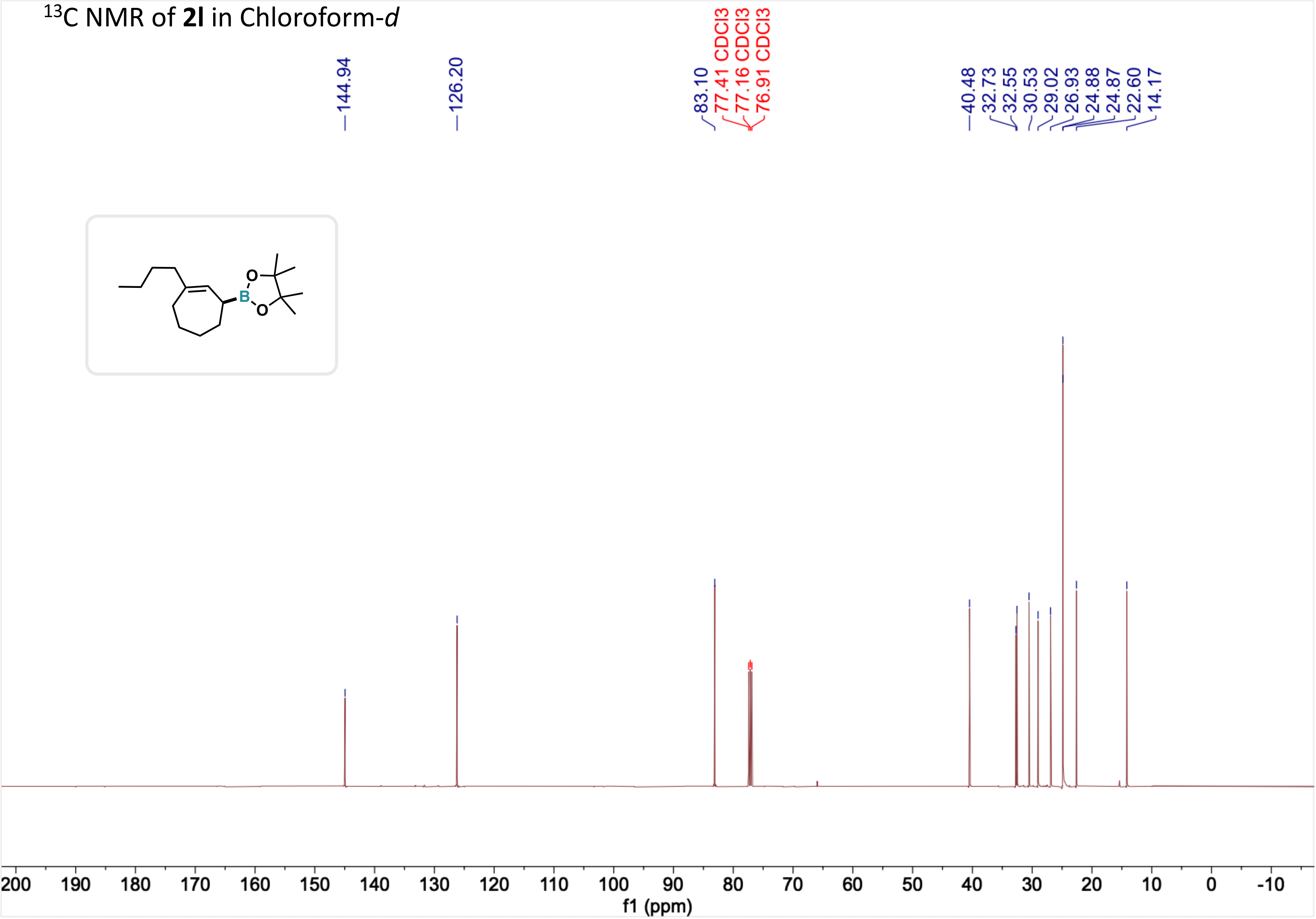

^{11}B NMR of **2l** in Chloroform-*d*

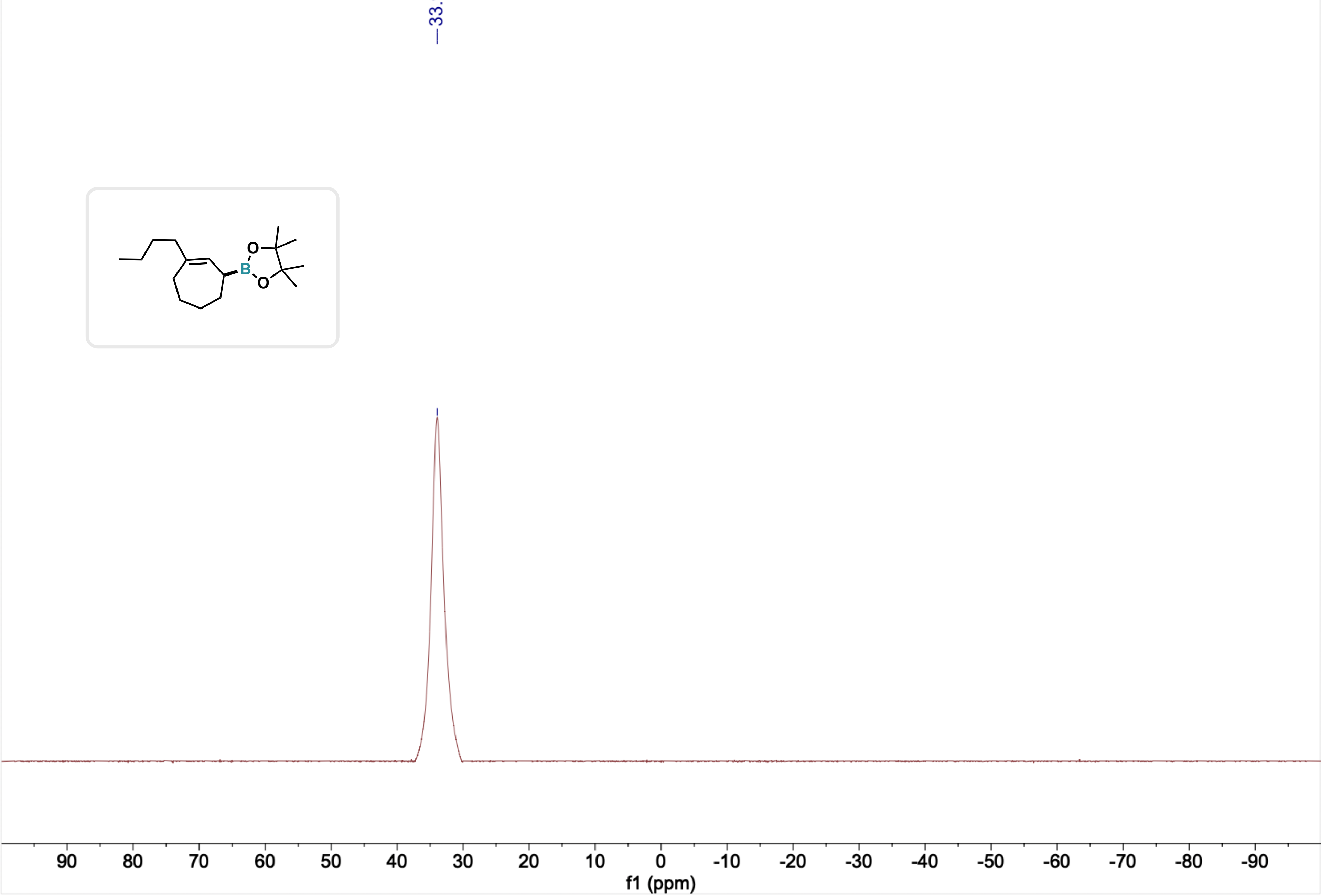

^{1}H NMR of **2m** in Chloroform-*d*

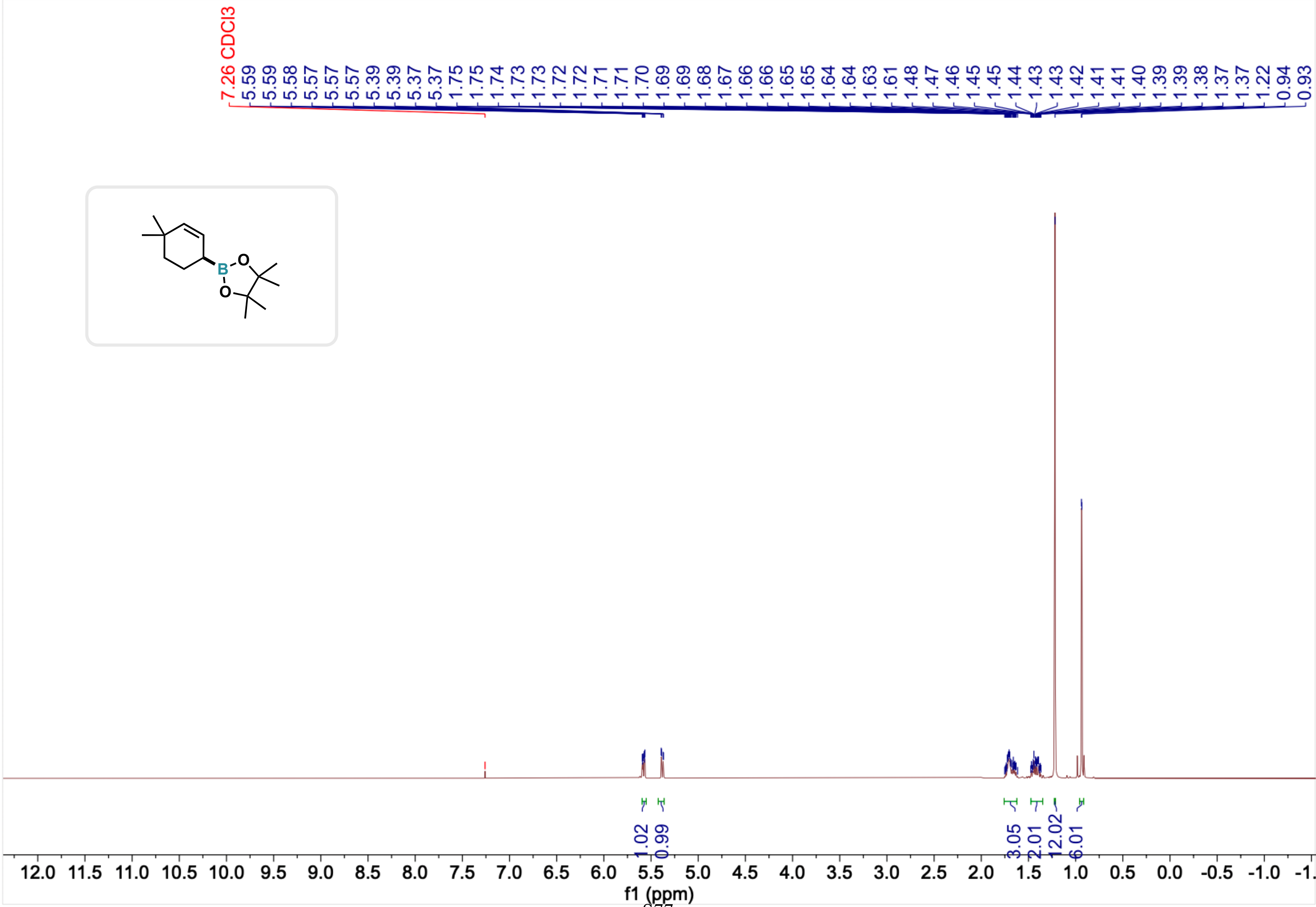

^{13}C NMR of **2m** in Chloroform-*d*

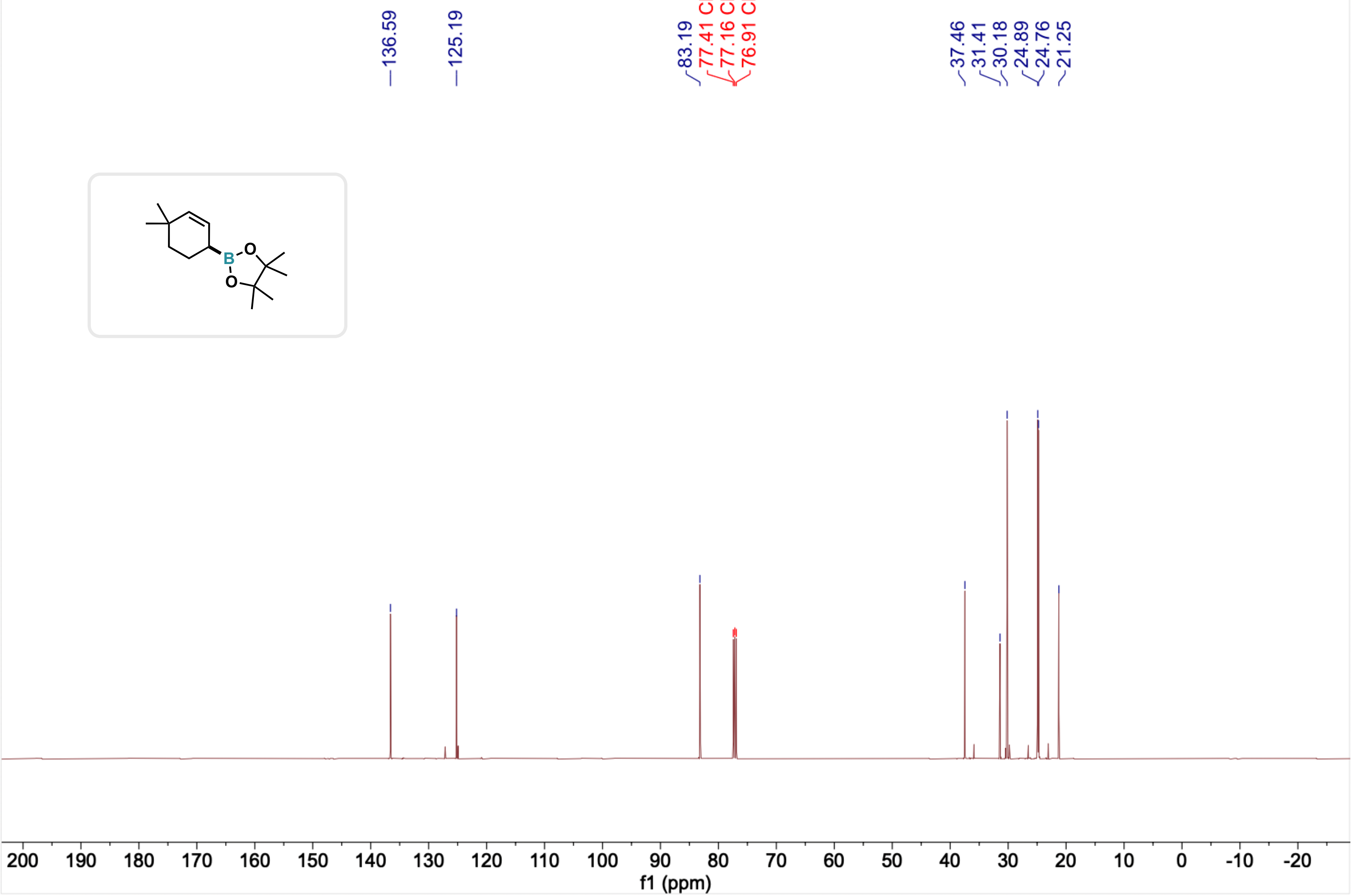

^{11}B NMR of **2m** in Chloroform-*d*

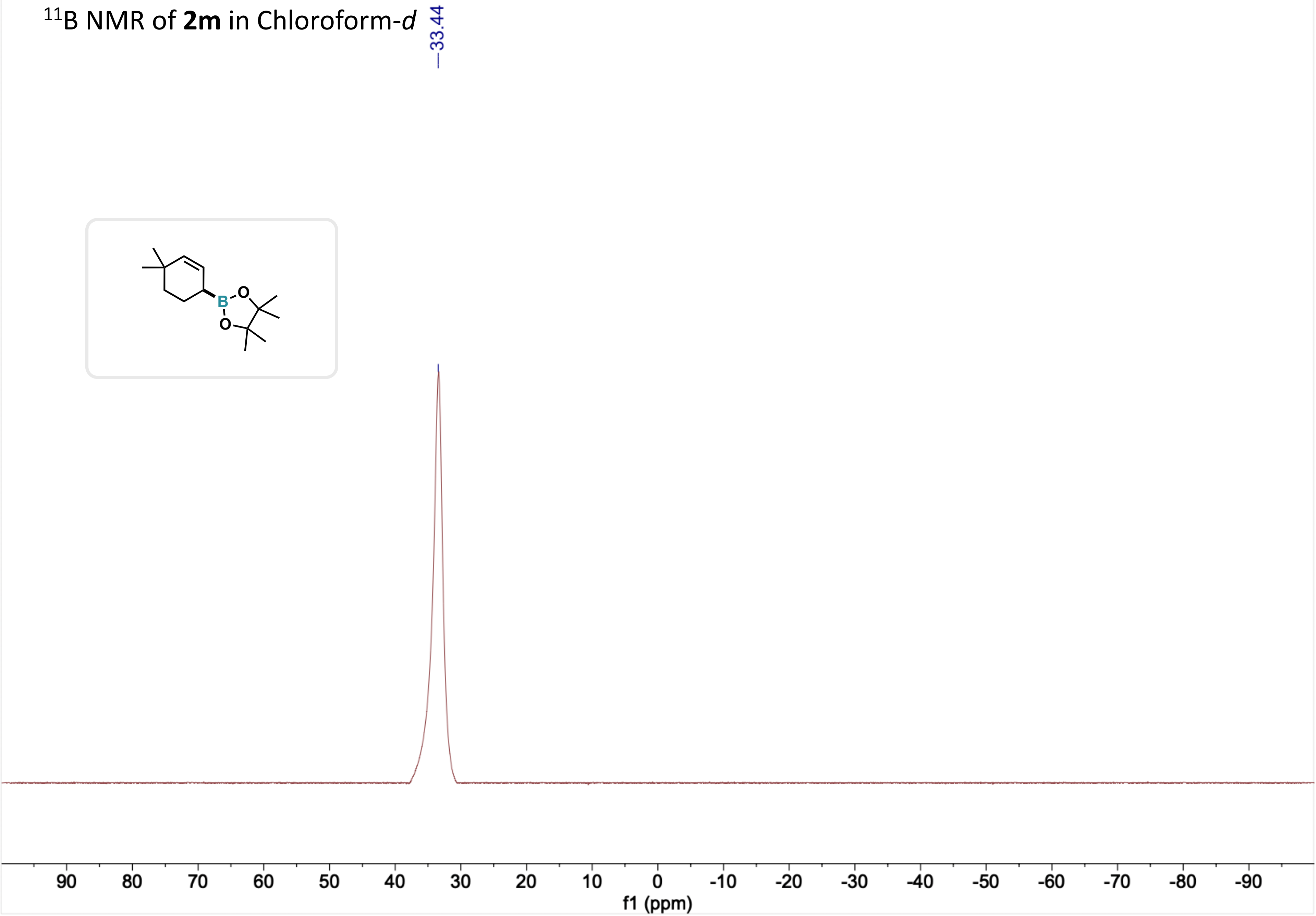

^1H NMR of **2n** in Chloroform-*d*

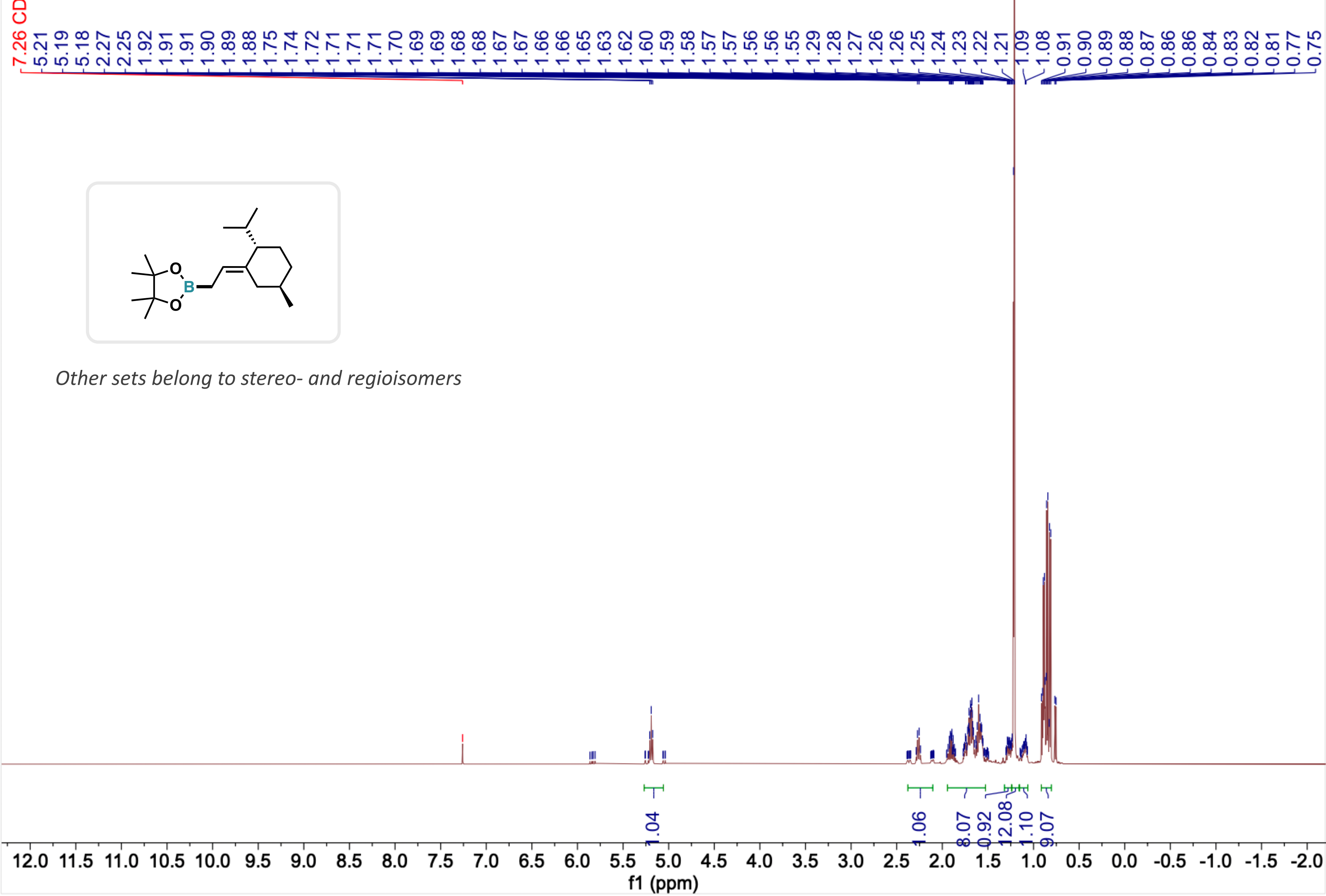

^{13}C NMR of **2n** in Chloroform-*d*

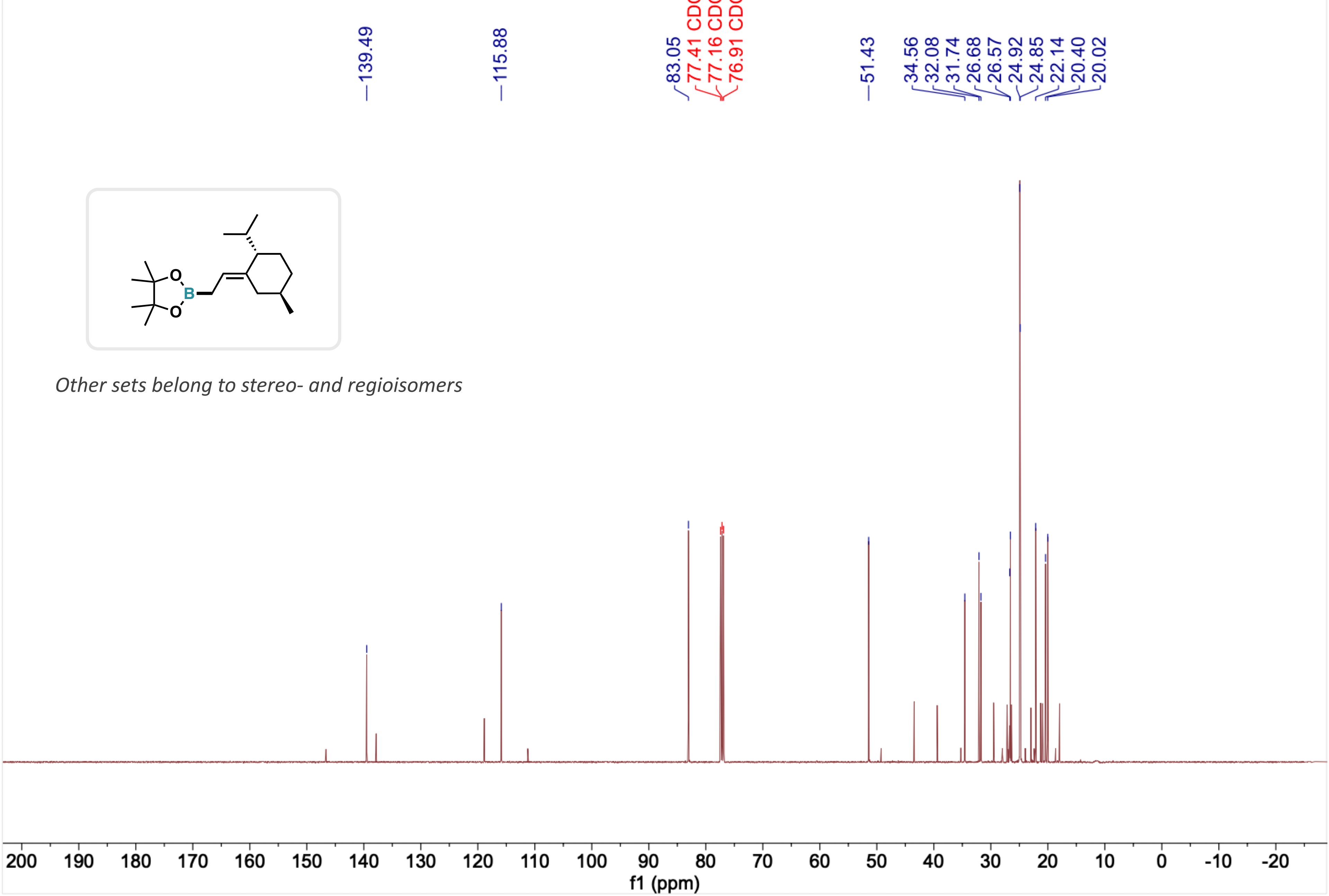

^{11}B NMR of **2n** in Chloroform-*d*

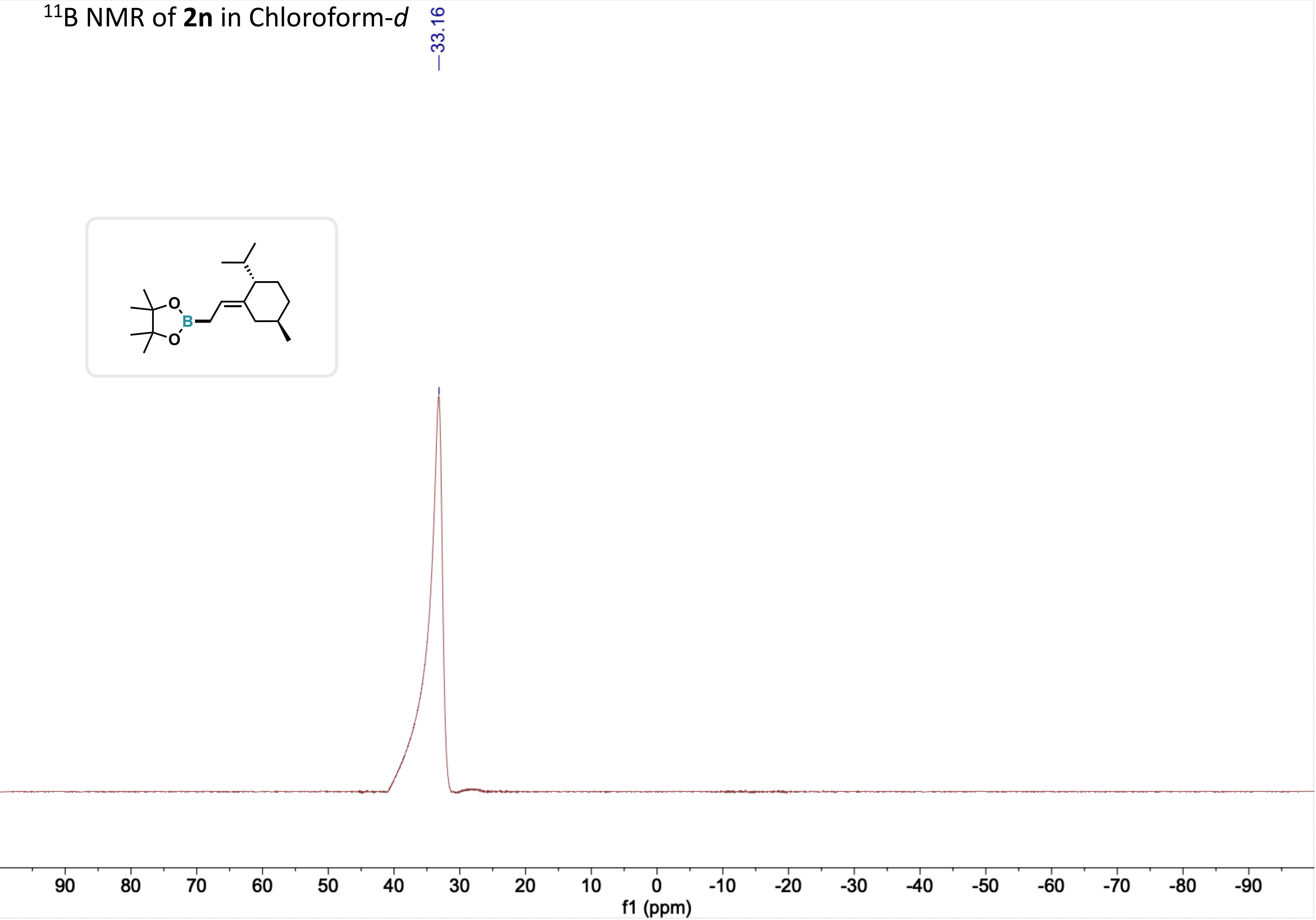

^{1}H NMR of **2o** in Chloroform-*d*

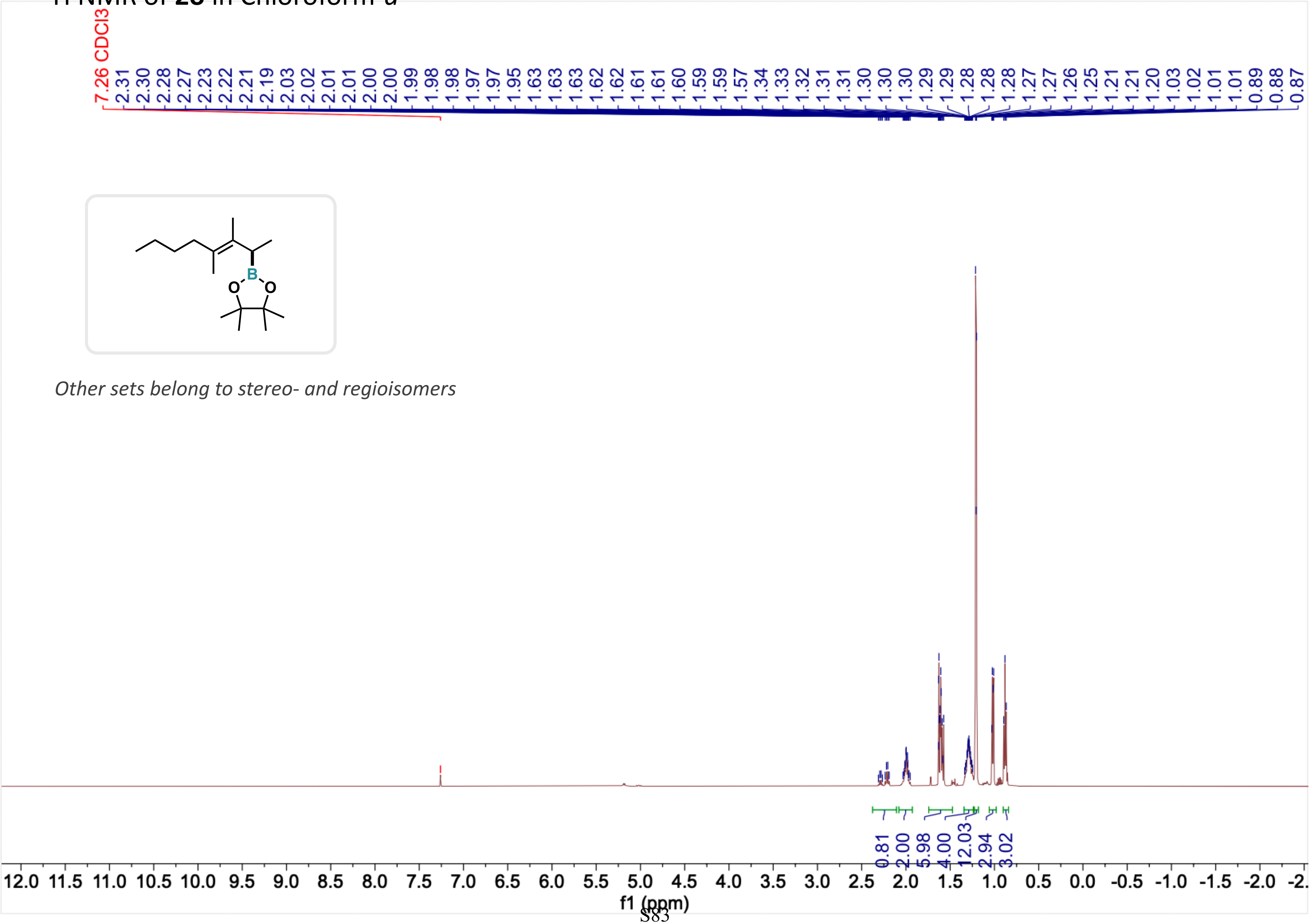

^{13}C NMR of **2o** in Chloroform-*d*

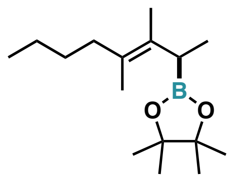

Other sets belong to stereo- and regioisomers

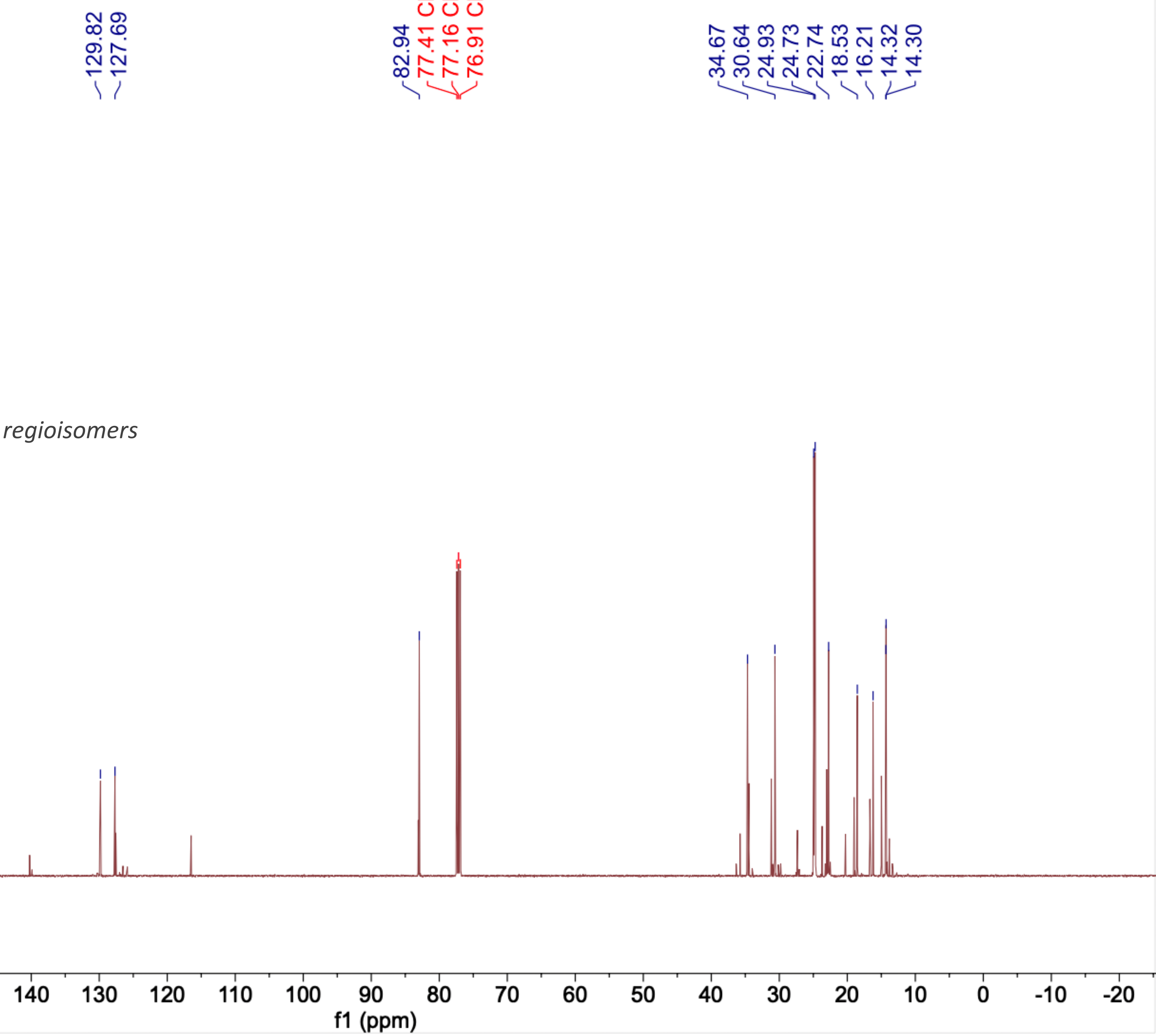

^{11}B NMR of **2o** in Chloroform-*d*

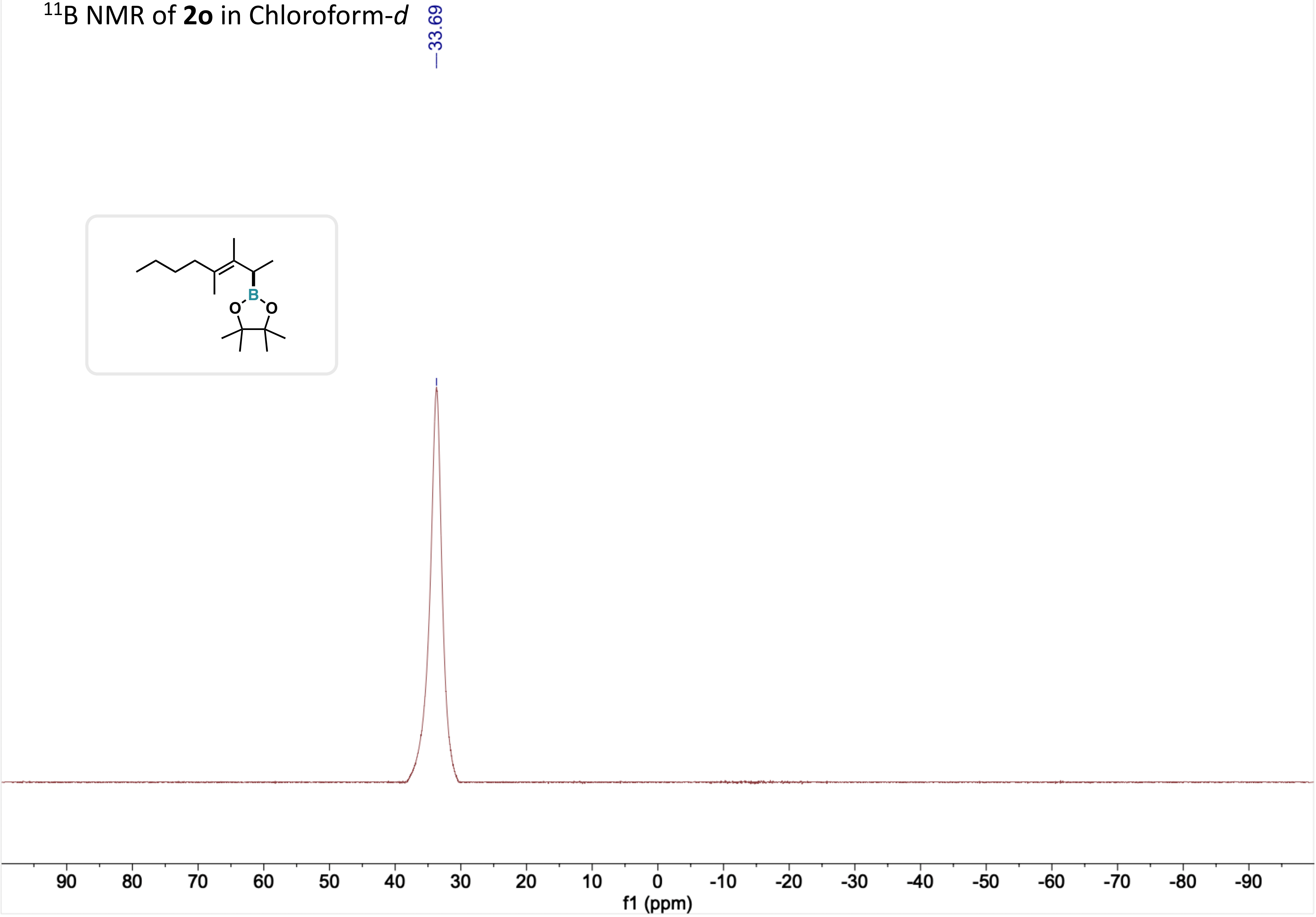

^{1}H NMR of **2p** in Chloroform-*d*

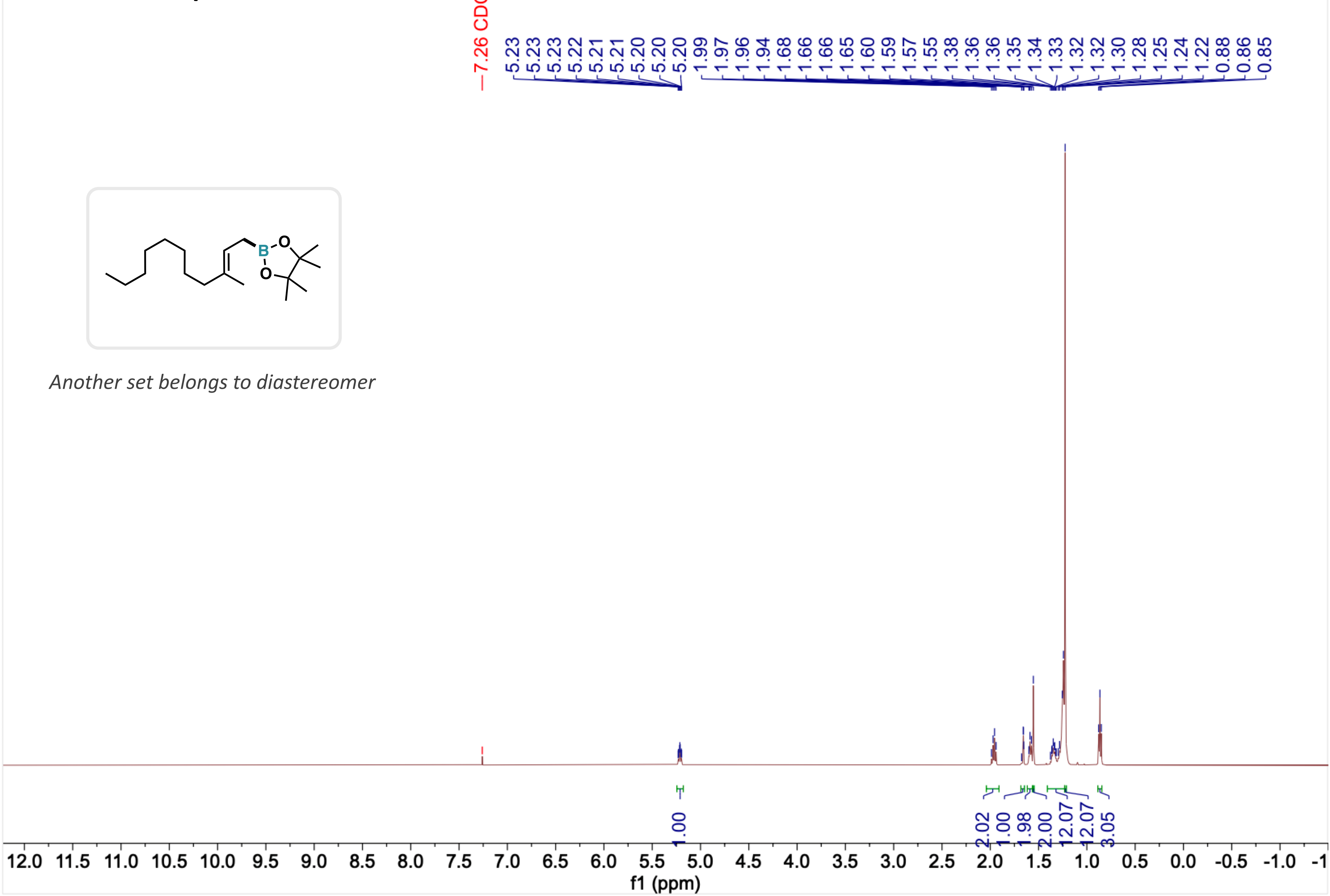

^{13}C NMR of **2p** in Chloroform-*d*

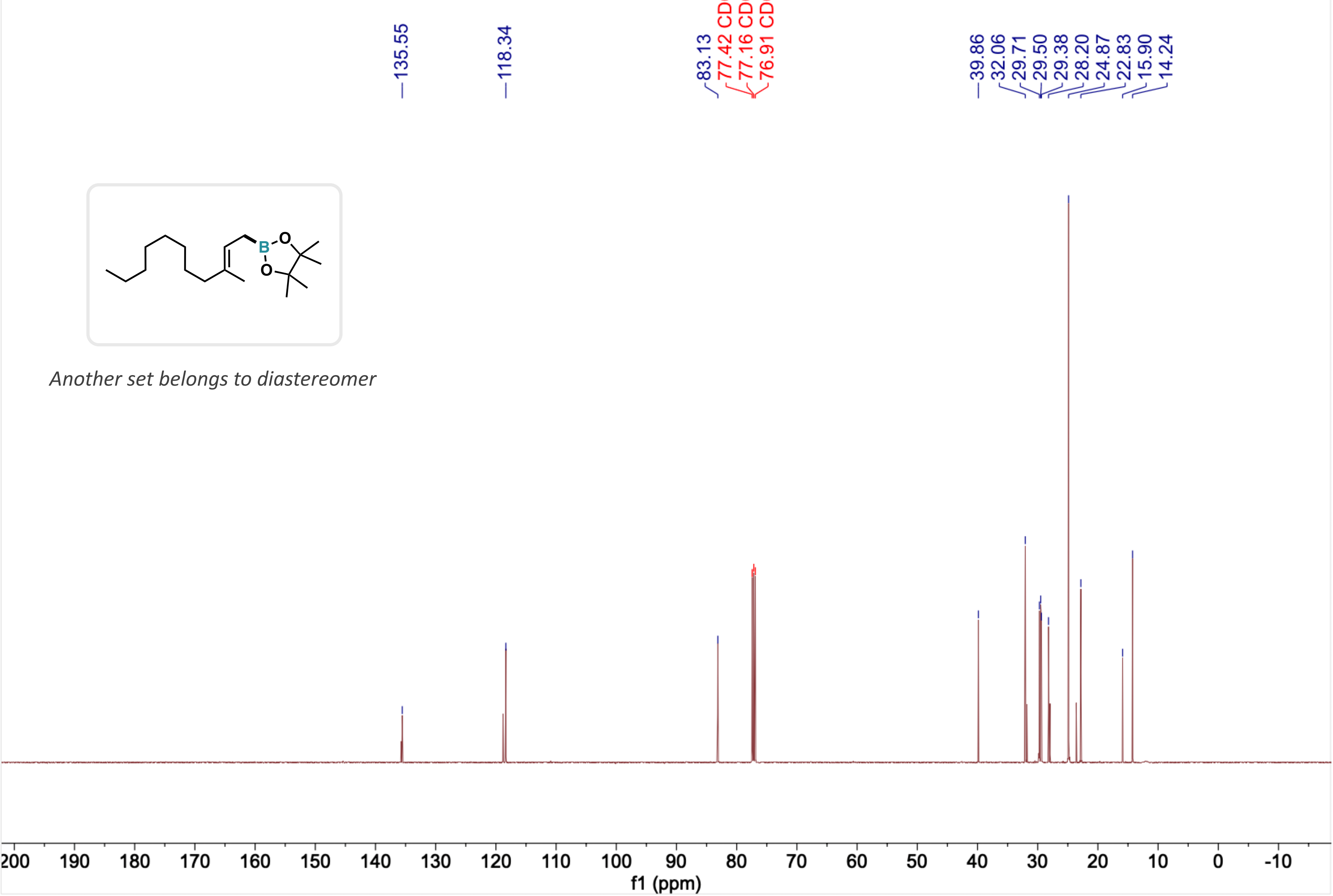

^{11}B NMR of **2p** in Chloroform-*d*

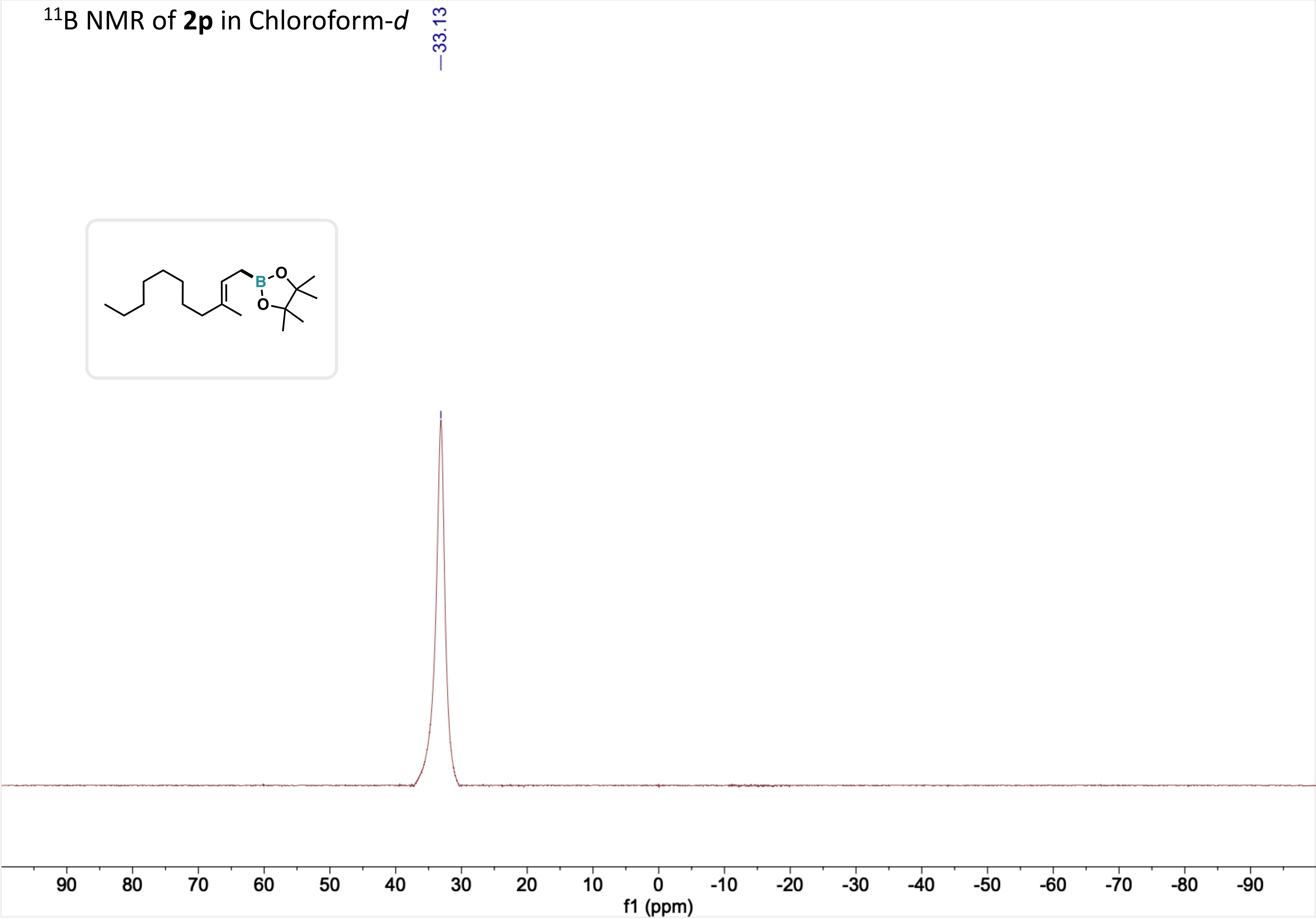

^{1}H NMR of **2q** in Chloroform-*d*

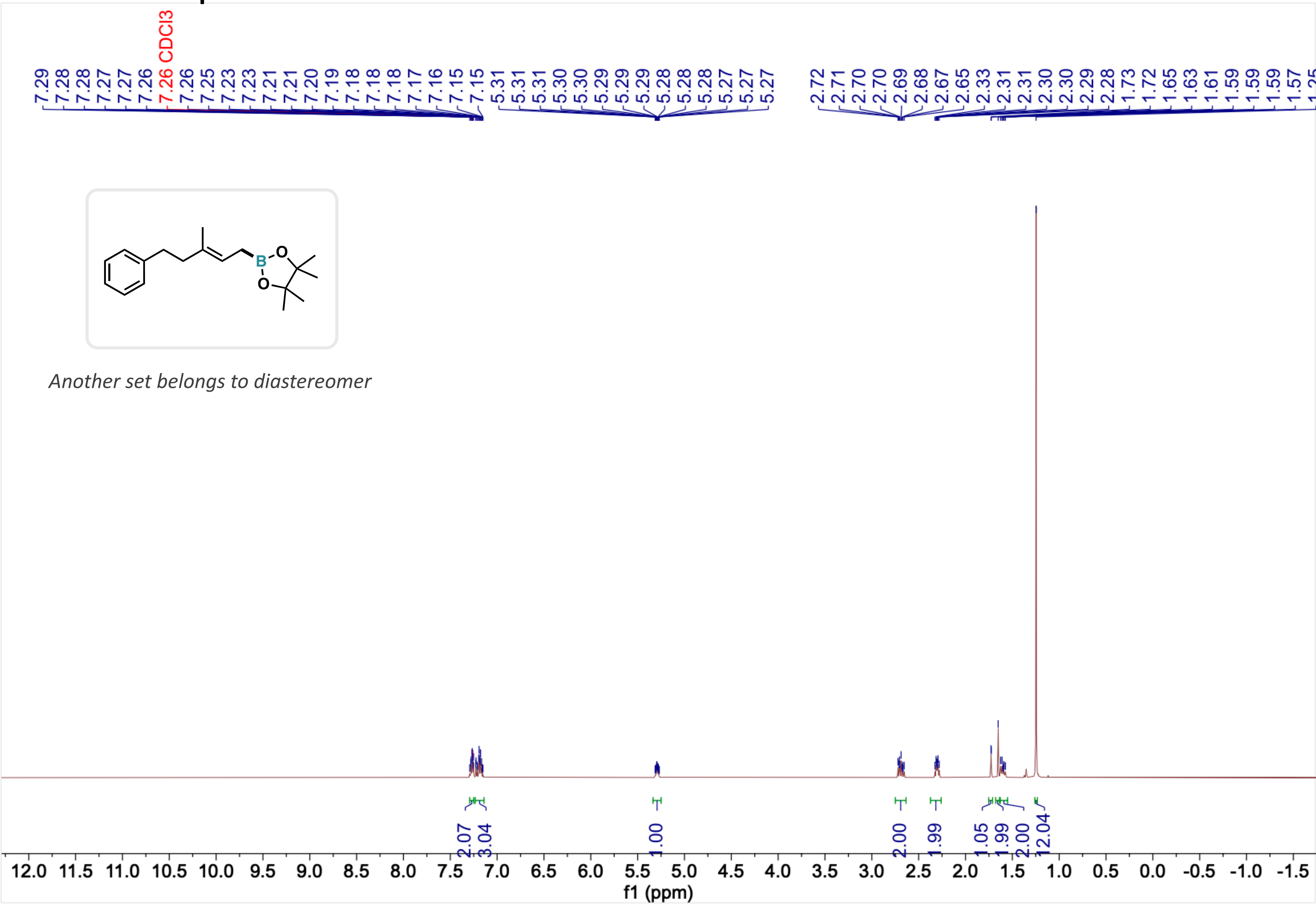

^{13}C NMR of **2q** in Chloroform-*d*

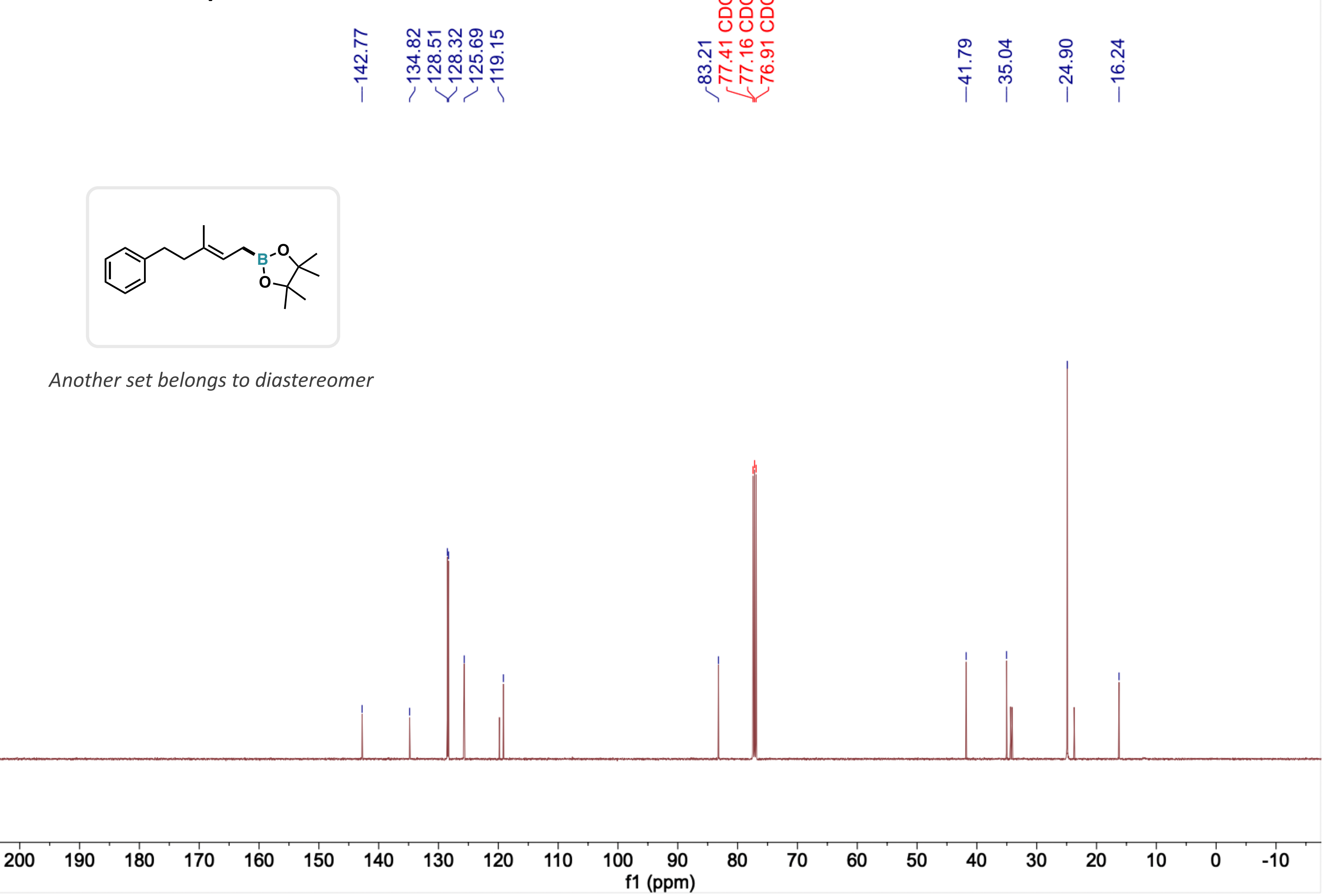

^{11}B NMR of **2q** in Chloroform-*d*

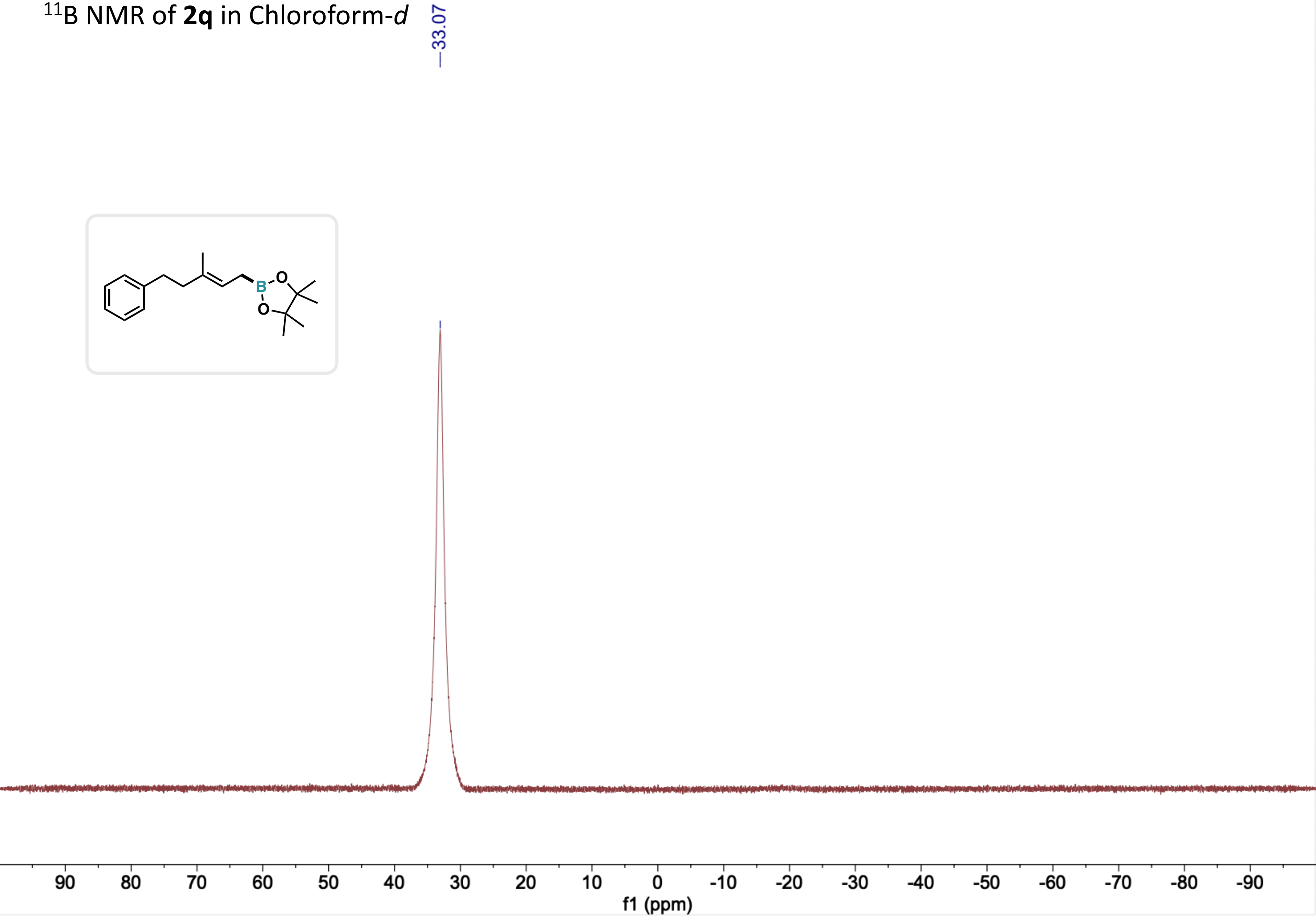

^{1}H NMR of **2r** in Chloroform-*d*

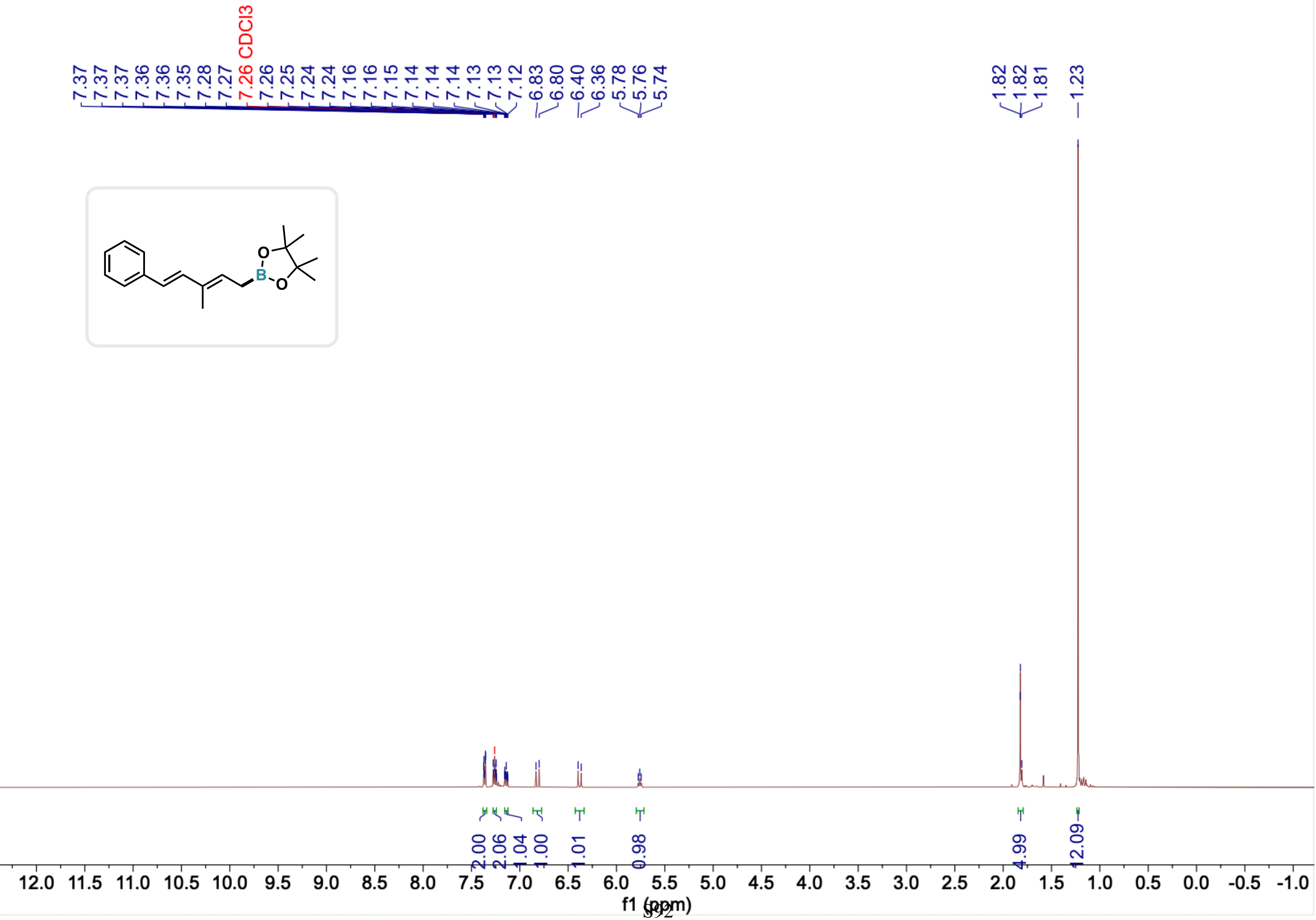

^{13}C NMR of **2r** in Chloroform-*d*

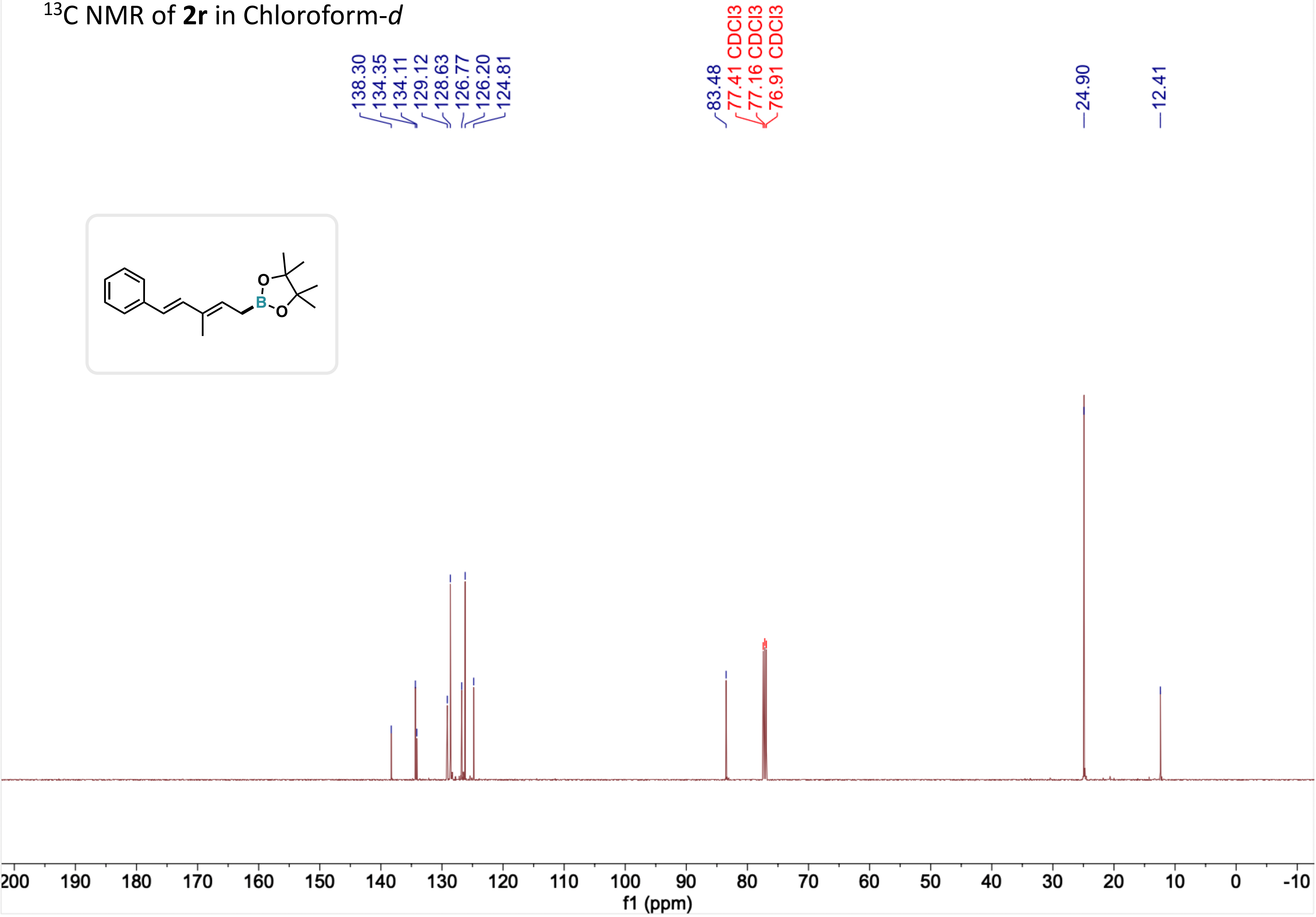

^{11}B NMR of **2r** in Chloroform-*d*

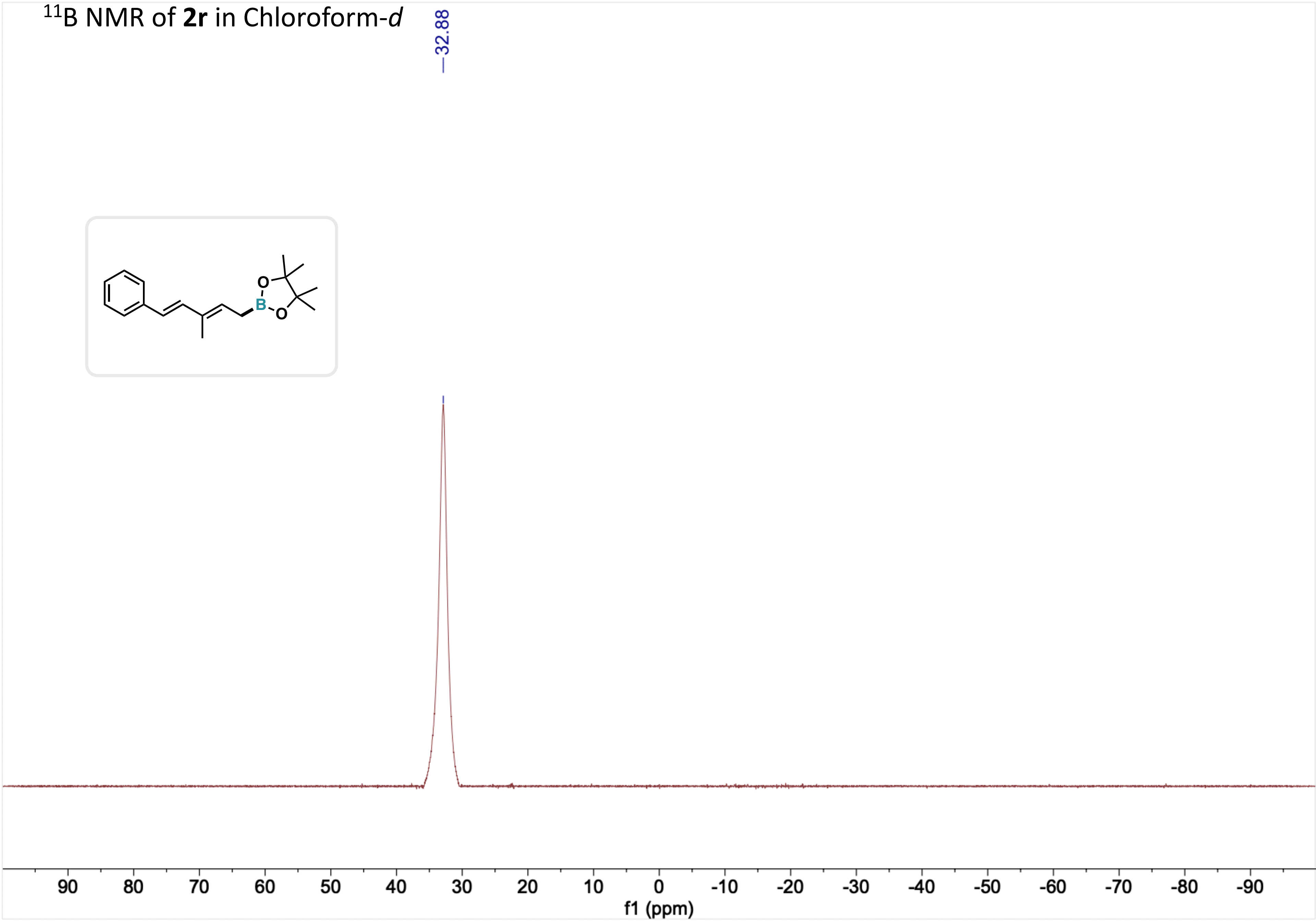

^{1}H NMR of **2s** in Chloroform-*d*

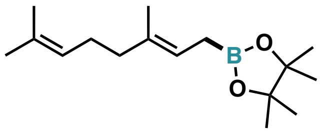

Another set belongs to diastereomer

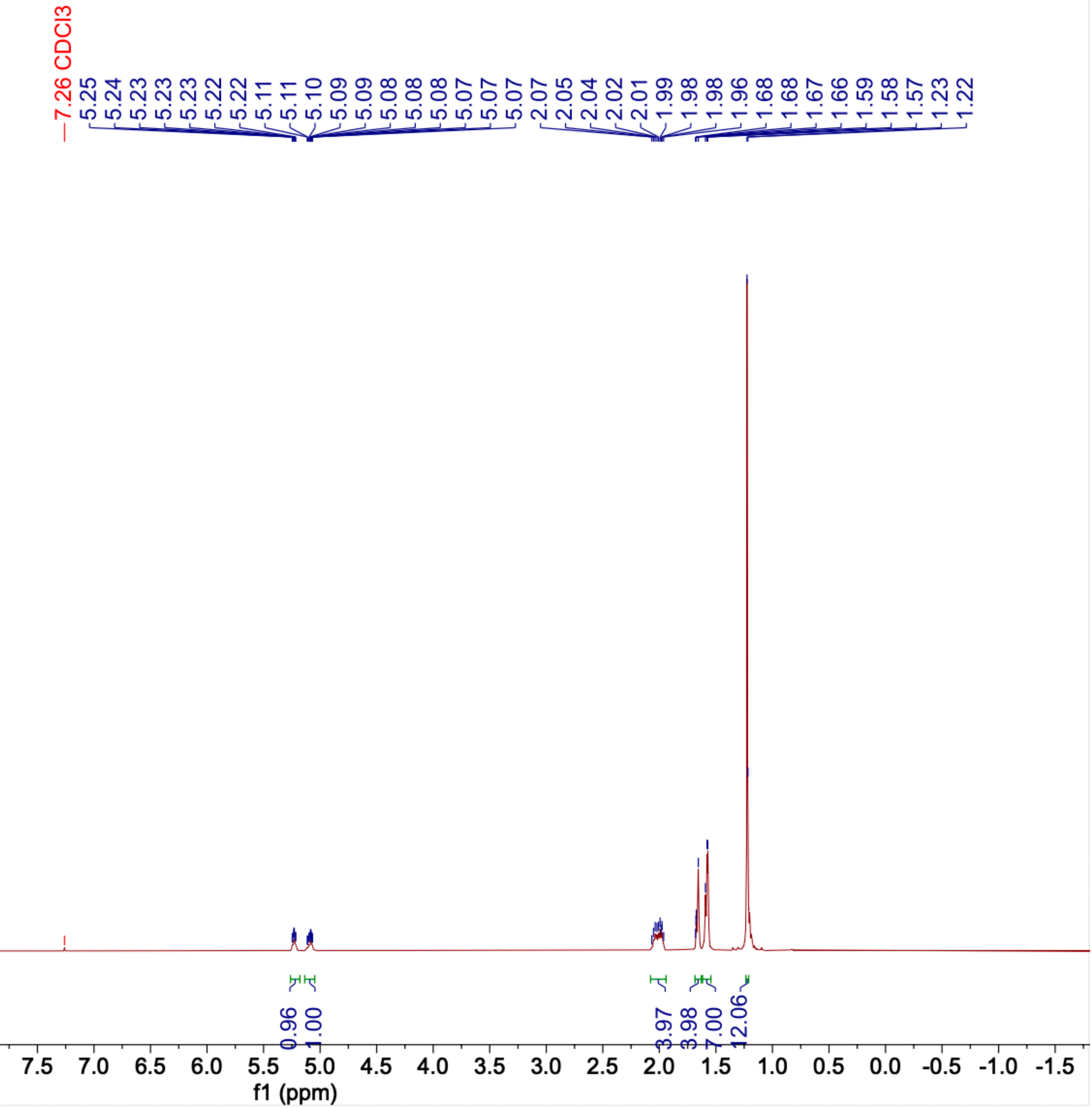

^{13}C NMR of **2s** in Chloroform-*d*

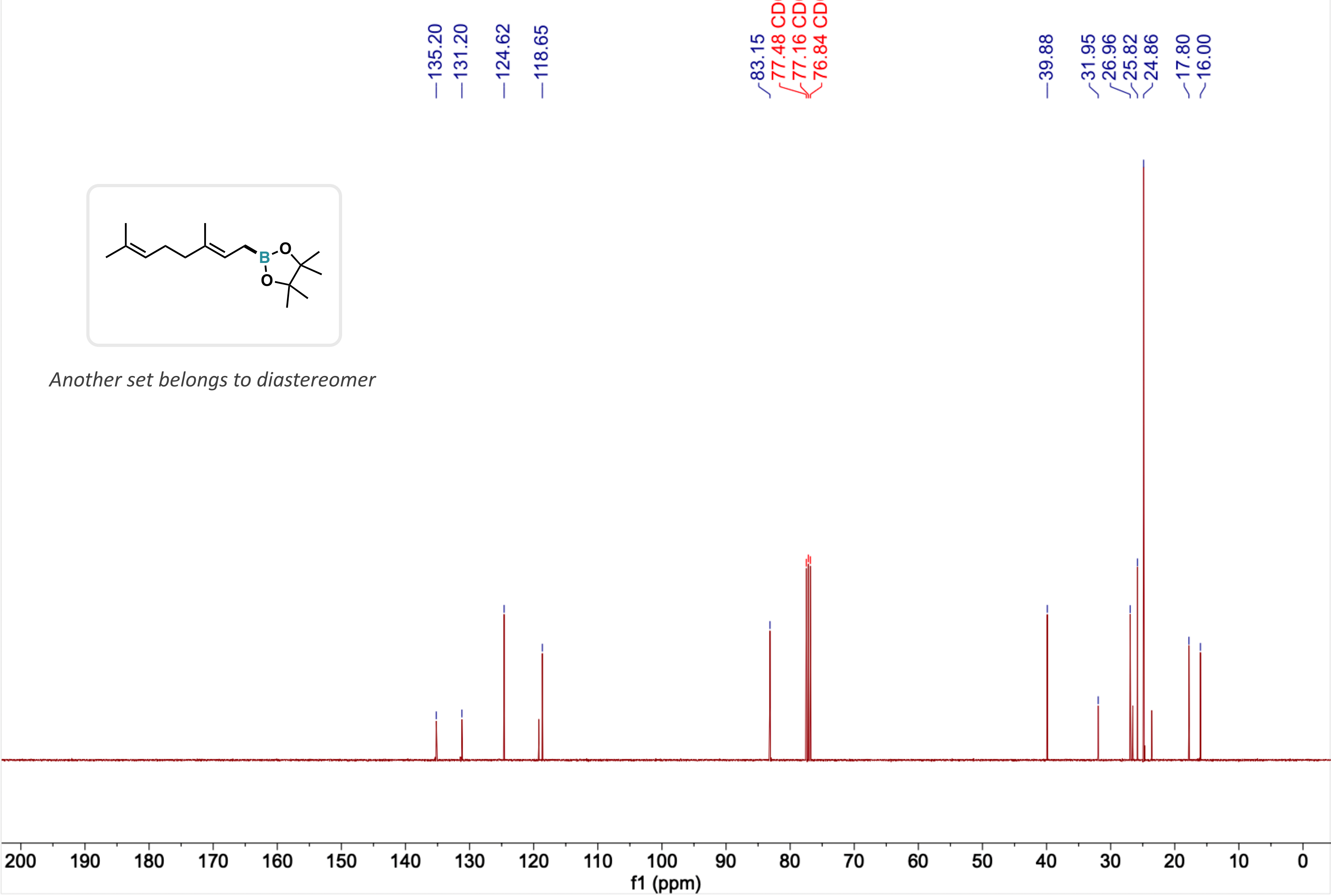

^{11}B NMR of **2s** in Chloroform-*d*

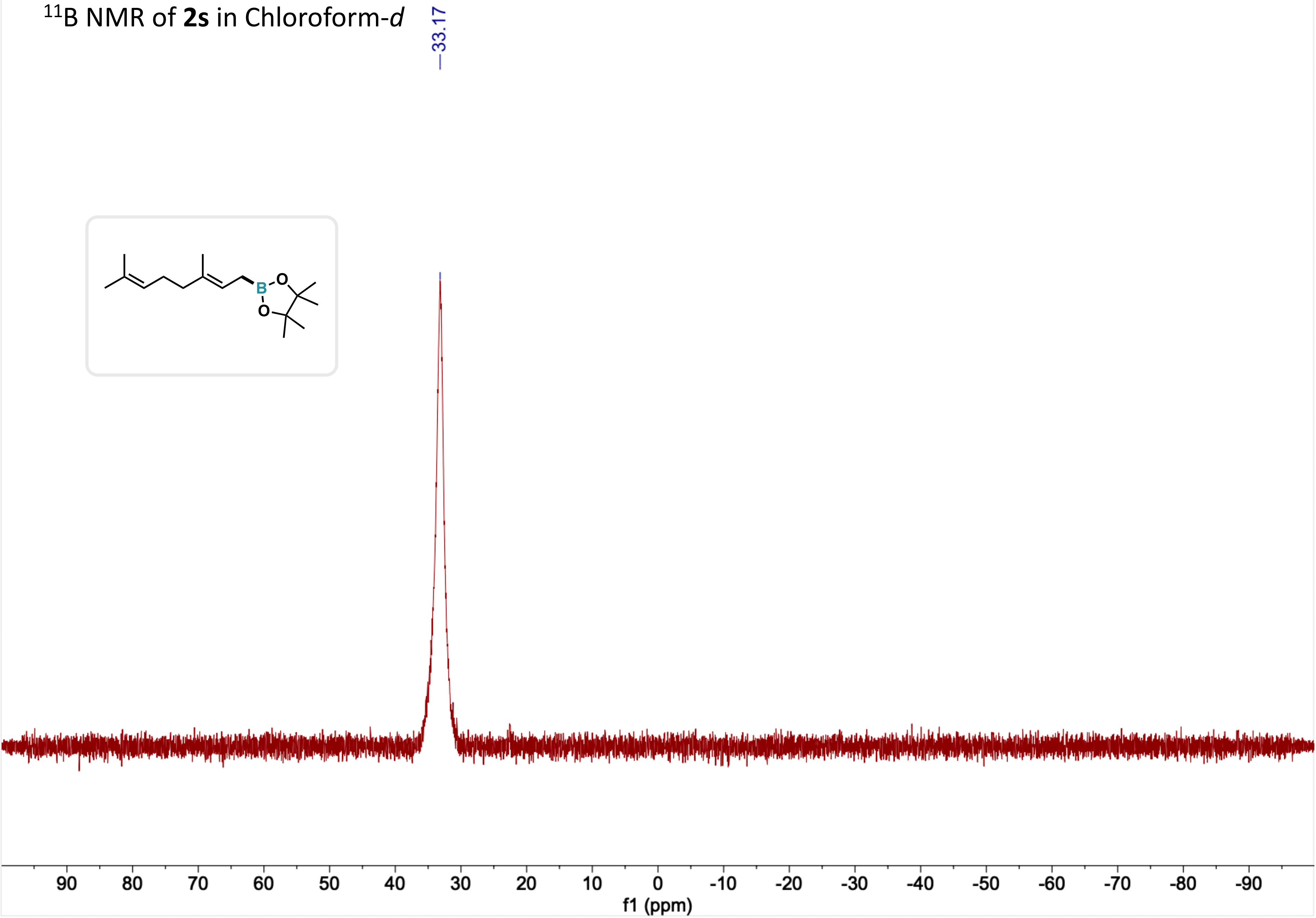

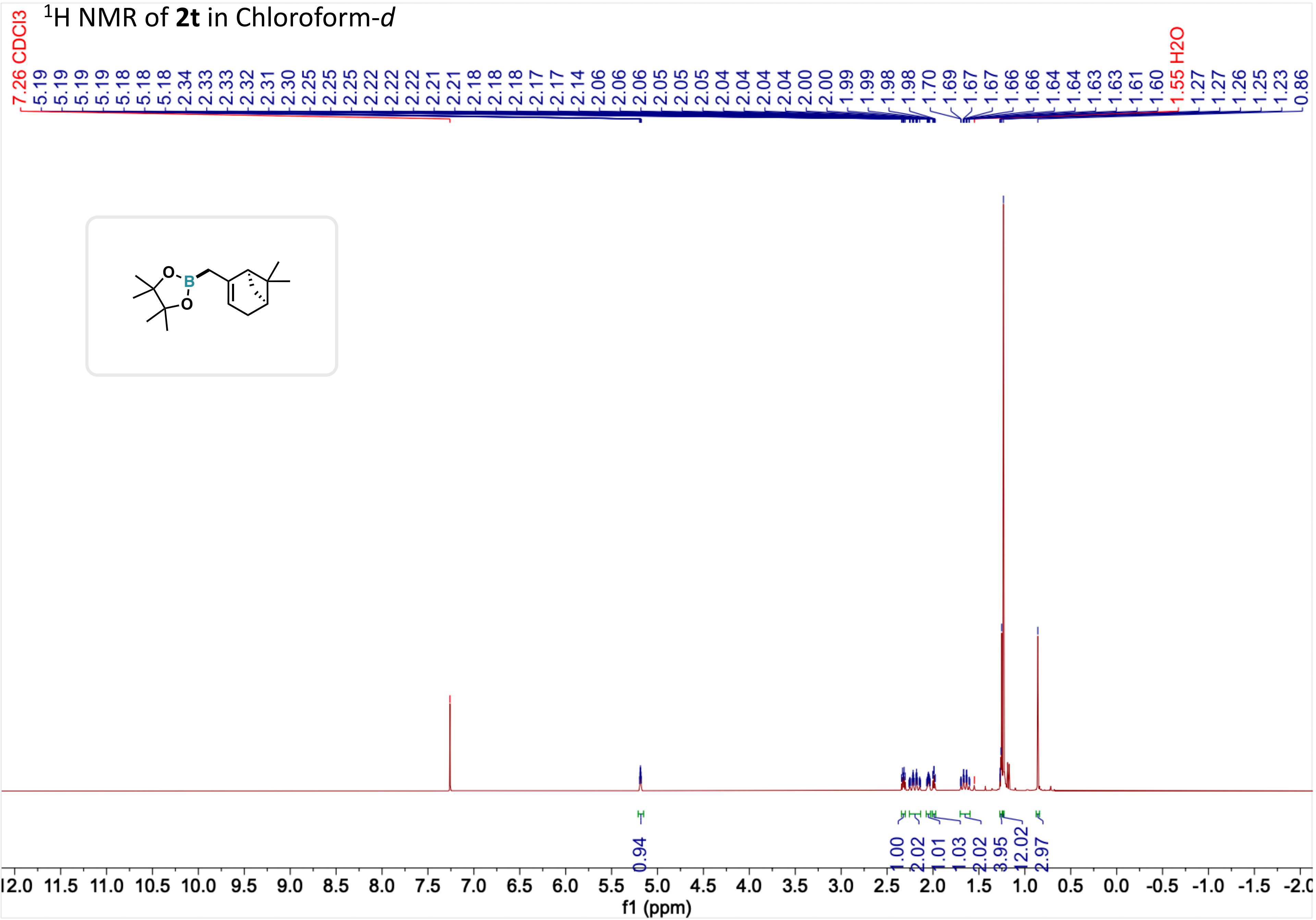

^{1}H NMR of **2t** in Chloroform-*d*
7.26 CDCl3
5.19
5.19
5.19
5.19
5.18
5.18
5.18
2.34
2.33
2.33
2.32
2.31
2.30
2.25
2.25
2.25
2.22
2.22
2.22
2.21
2.21
2.18
2.18
2.18
2.17
2.17
2.14
2.06
2.06
2.06
2.05
2.05
2.05
2.04
2.04
2.04
2.04
2.00
2.00
1.99
1.99
1.98
1.98
1.70
1.69
1.67
1.67
1.66
1.66
1.64
1.64
1.63
1.63
1.61
1.60
1.55 H2O
1.27
1.27
1.26
1.25
1.23
0.86
0.94
1.00
2.02
1.01
1.03
2.02
3.95
12.02
2.97
f1 (ppm)

^{13}C NMR of **2t** in Chloroform-*d*

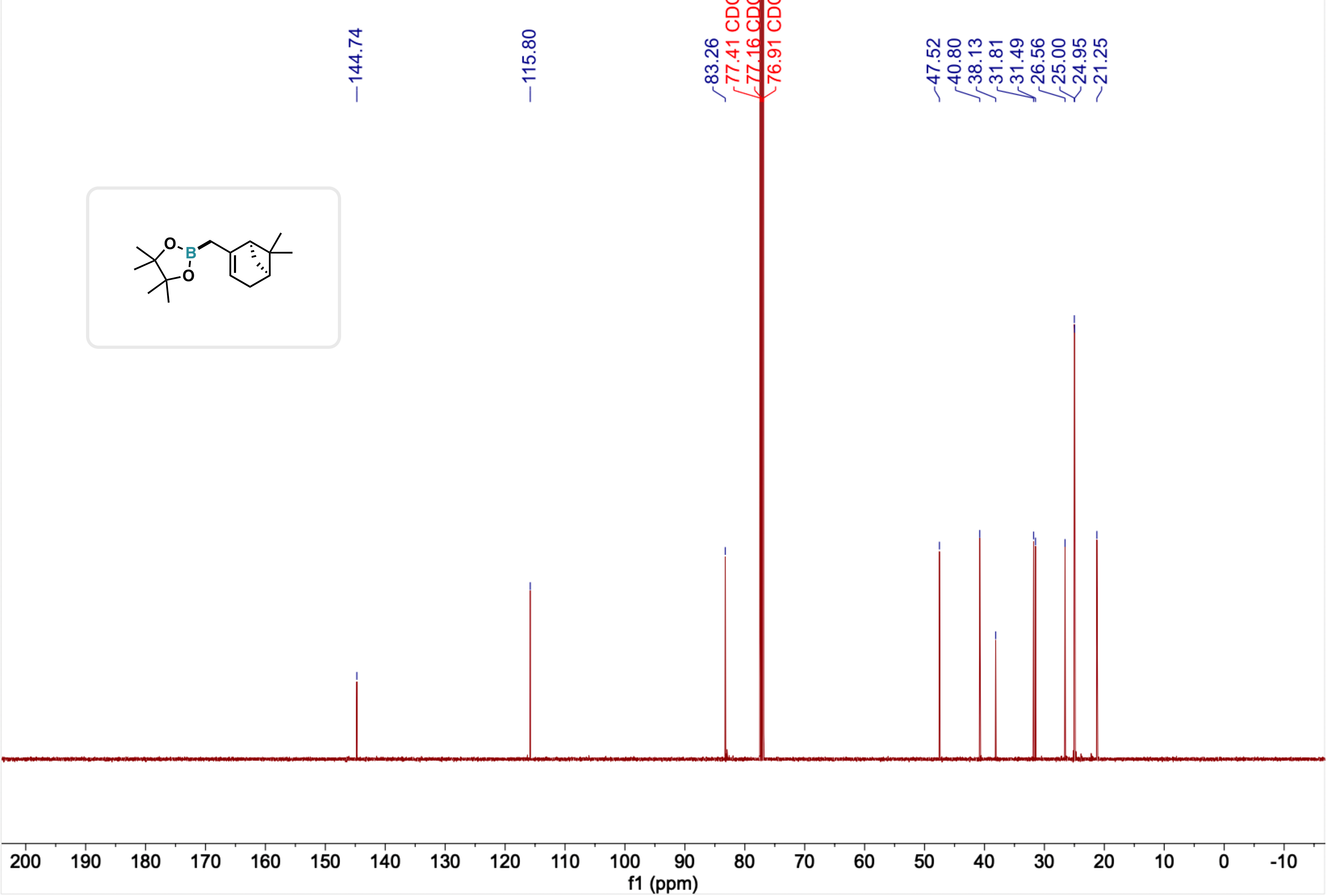

^{11}B NMR of **2t** in Chloroform-*d*

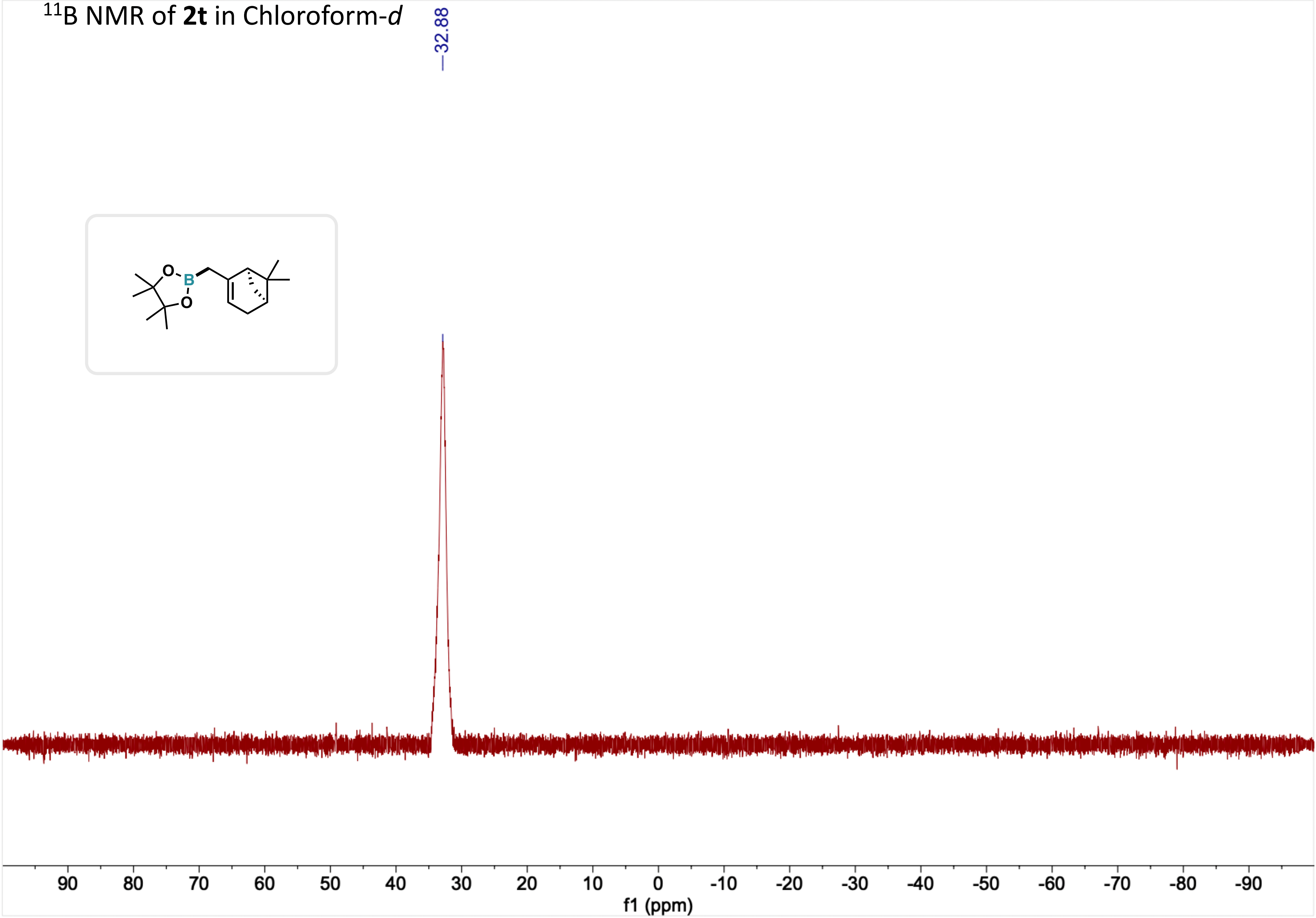

^{1}H NMR of **2u** in Chloroform-*d*

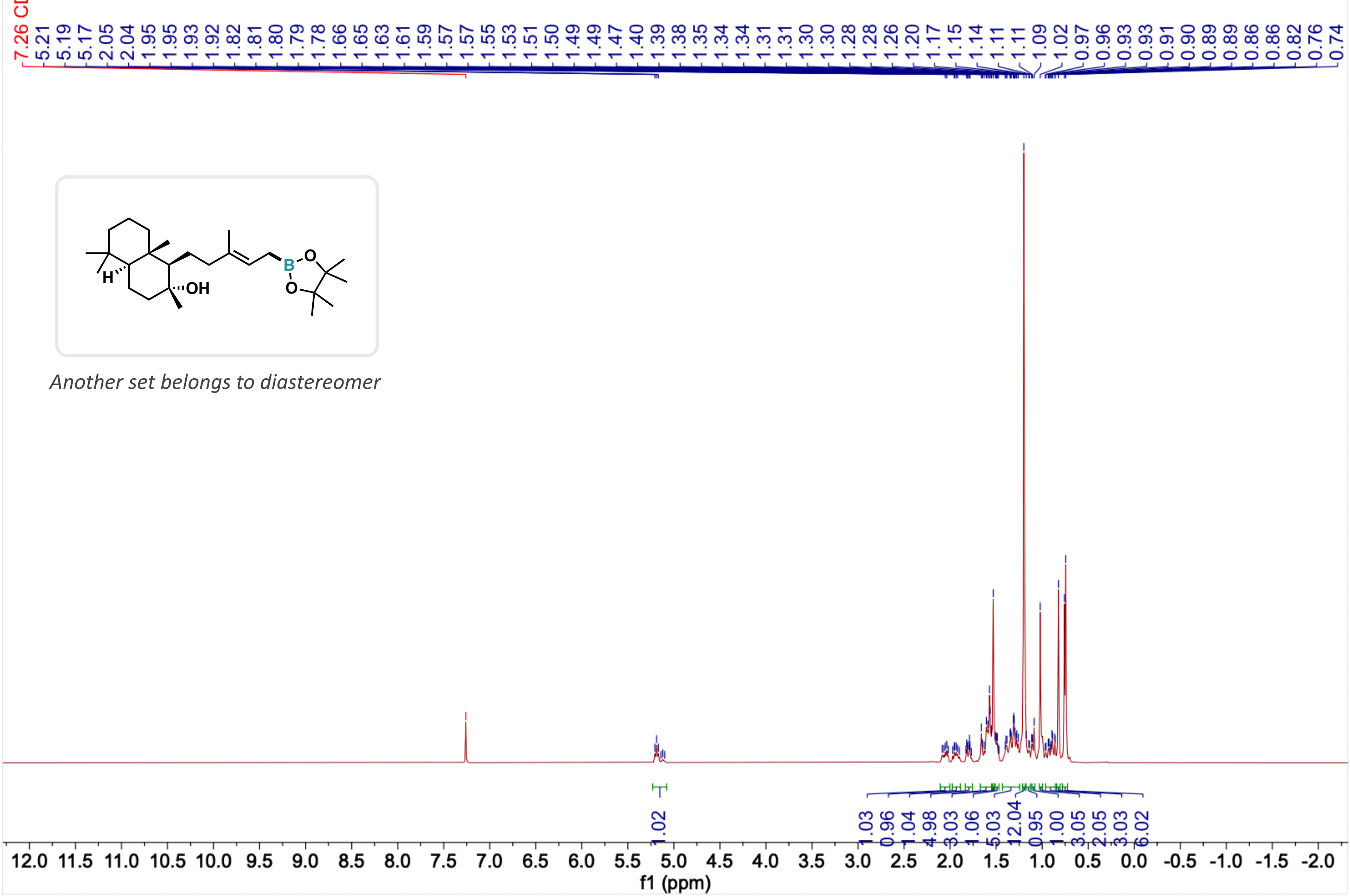

Another set belongs to diastereomer

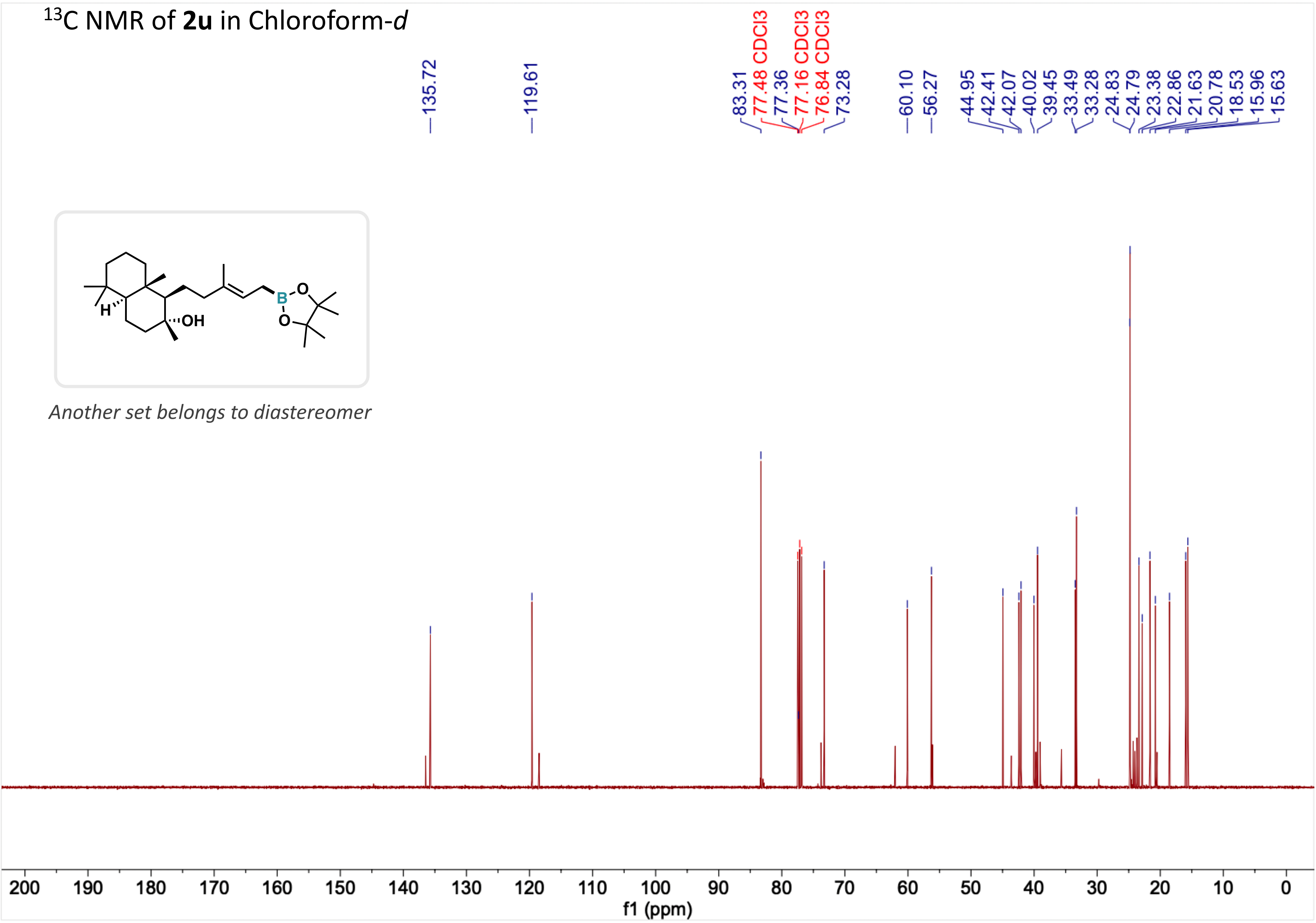

^{13}C NMR of **2u** in Chloroform-*d*
135.72
119.61
83.31
77.48 CDCl3
77.36
77.16 CDCl3
76.84 CDCl3
73.28
60.10
56.27
44.95
42.41
42.07
40.02
39.45
33.49
33.28
24.83
24.79
23.38
22.86
21.63
20.78
18.53
15.96
15.63
H
OH
B
O
O
Another set belongs to diastereomer
200
190
180
170
160
150
140
130
120
110
100
90
80
70
60
50
40
30
20
10
0
f1 (ppm)

^{11}B NMR of **2u** in Chloroform-*d*

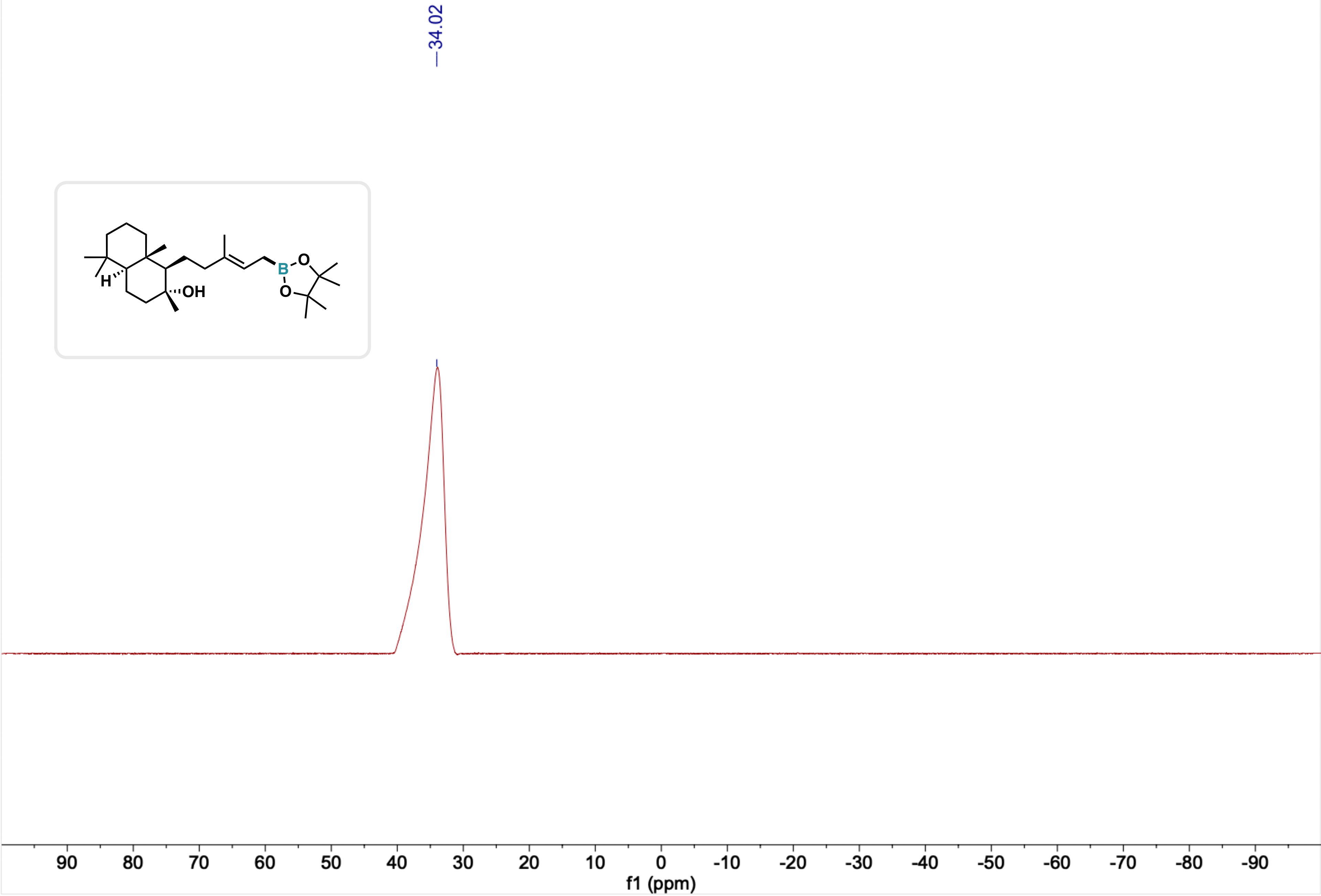

^{1}H NMR of **2v** in Chloroform-*d*

Another set belongs to diastereomer

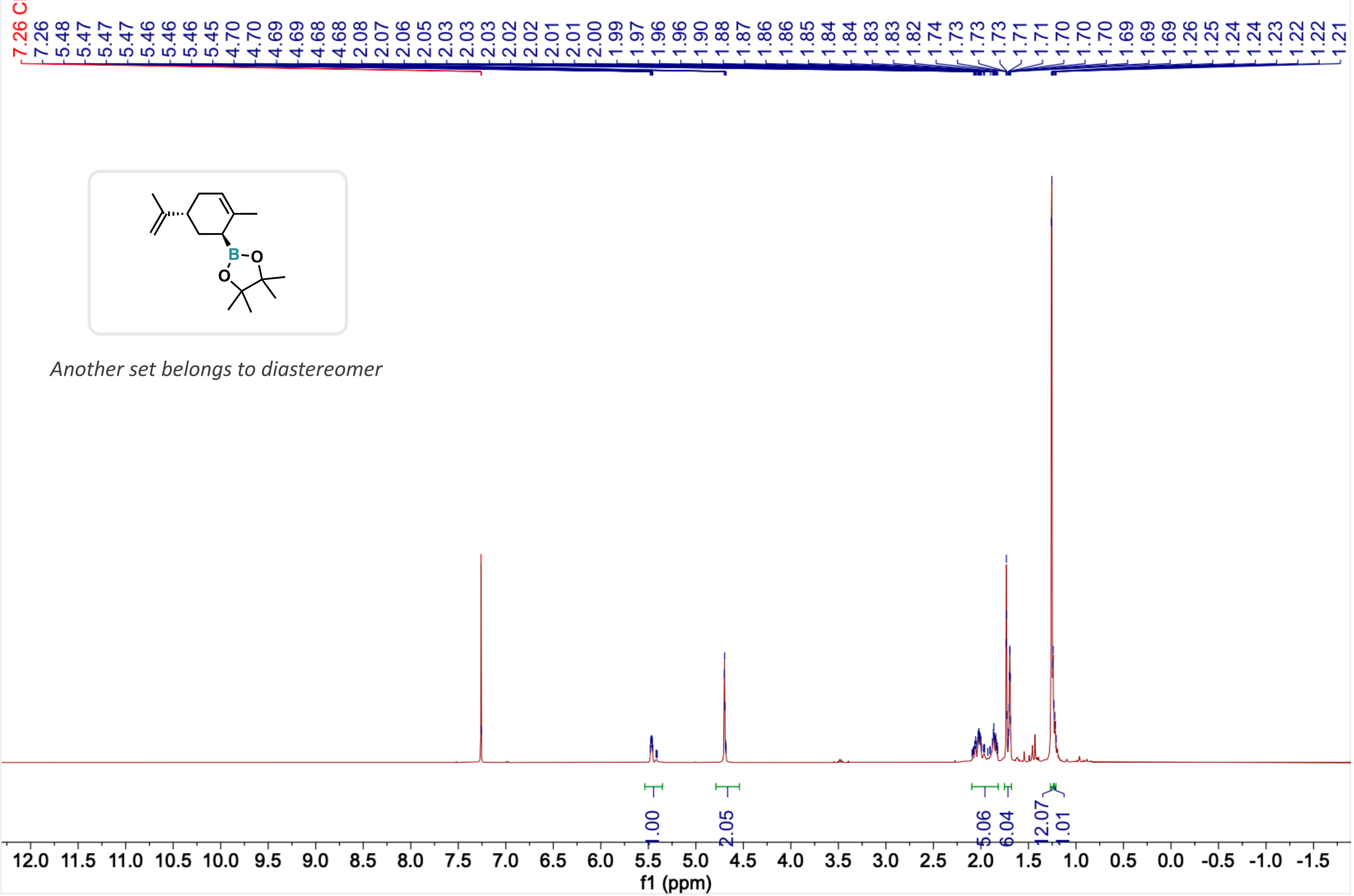

¹³C NMR of **2v** in Chloroform-*d*

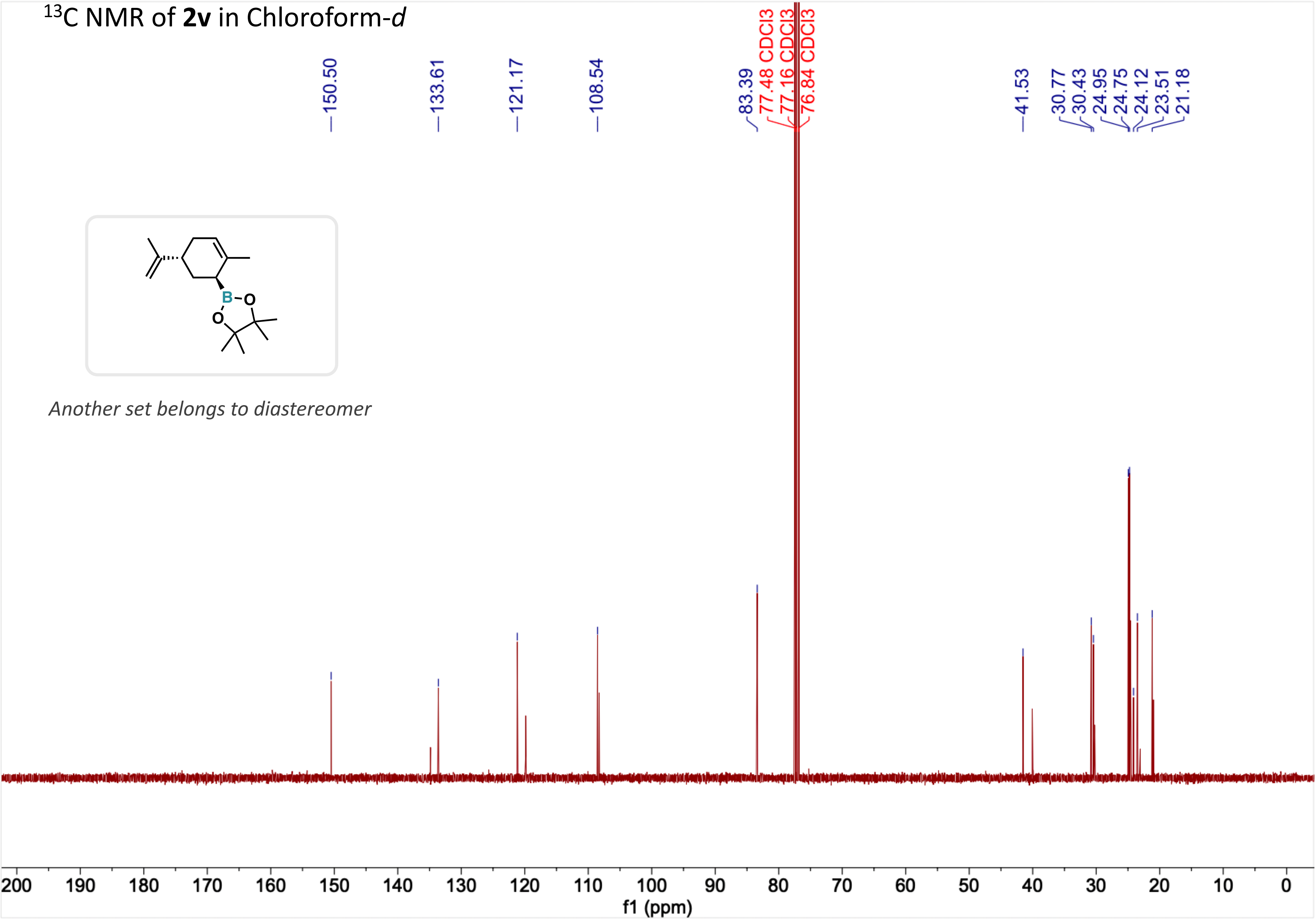

^{11}B NMR of **2v** in Chloroform-*d*

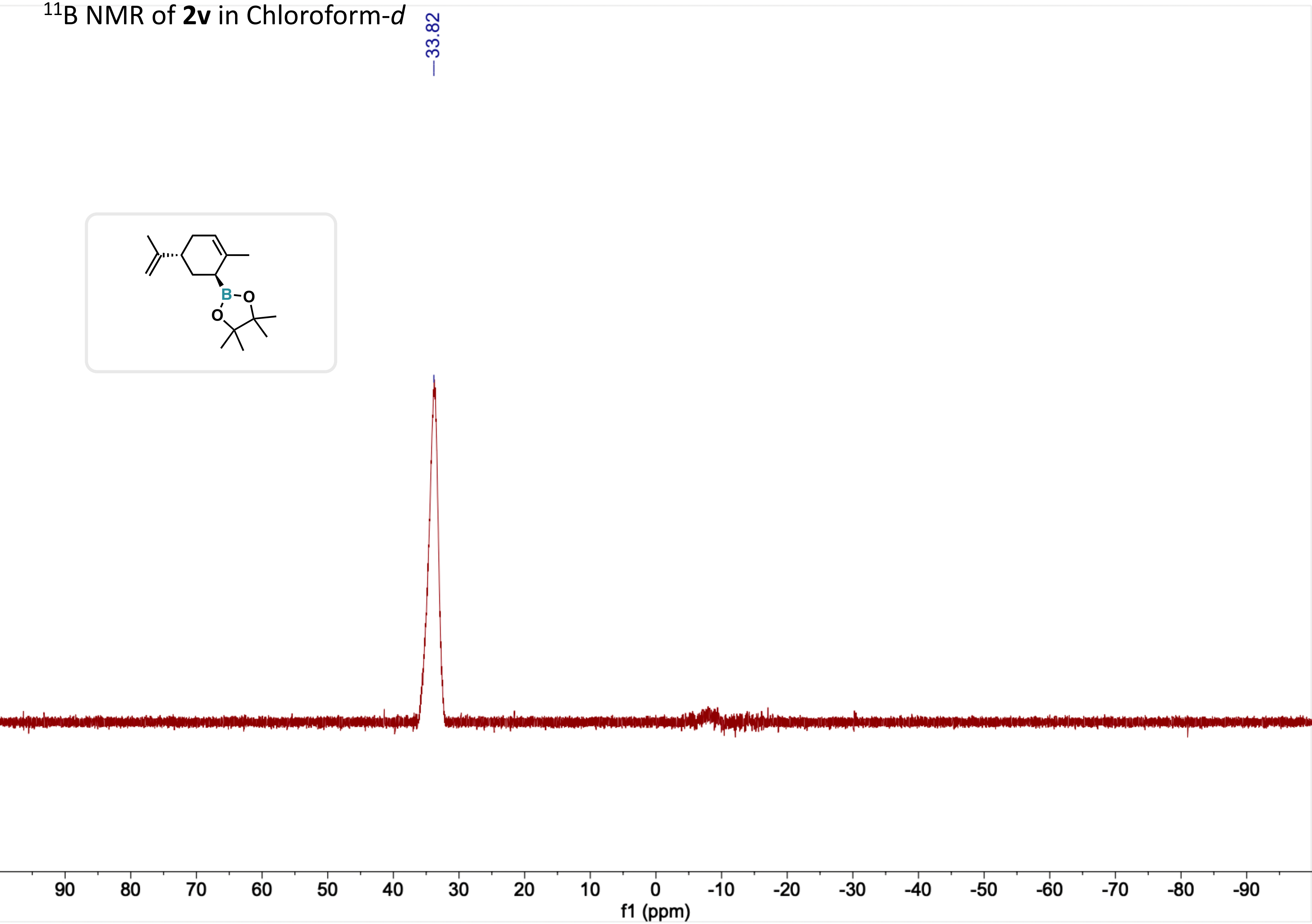

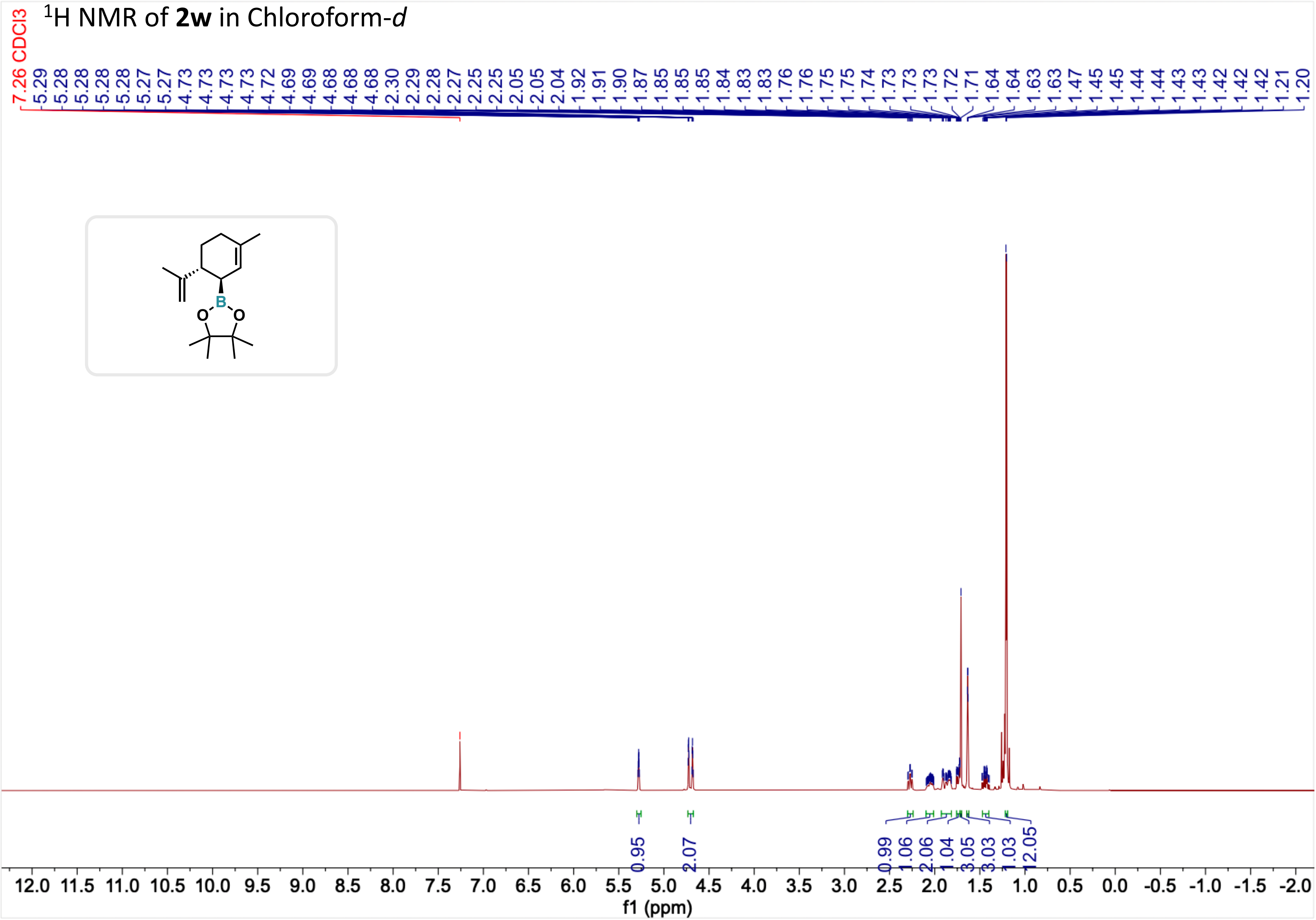

¹H NMR of 2w in Chloroform-d
7.26 CDCl3
5.29 5.28 5.28 5.28 5.28 5.27 5.27 4.73 4.73 4.73 4.73 4.72 4.69 4.69 4.68 4.68 4.68 2.30 2.29 2.28 2.27 2.25 2.25 2.05 2.05 2.04 1.92 1.91 1.90 1.87 1.85 1.85 1.85 1.84 1.83 1.83 1.76 1.76 1.75 1.75 1.74 1.73 1.73 1.73 1.72 1.71 1.64 1.64 1.63 1.63 1.47 1.45 1.45 1.44 1.44 1.43 1.43 1.42 1.42 1.42 1.21 1.20
B
O
O
0.95
2.07
0.99
1.06
2.06
1.04
3.05
3.03
1.03
12.05
12.0 11.5 11.0 10.5 10.0 9.5 9.0 8.5 8.0 7.5 7.0 6.5 6.0 5.5 5.0 4.5 4.0 3.5 3.0 2.5 2.0 1.5 1.0 0.5 0.0 -0.5 -1.0 -1.5 -2.0
f1 (ppm)

^{13}C NMR of **2w** in Chloroform-*d*

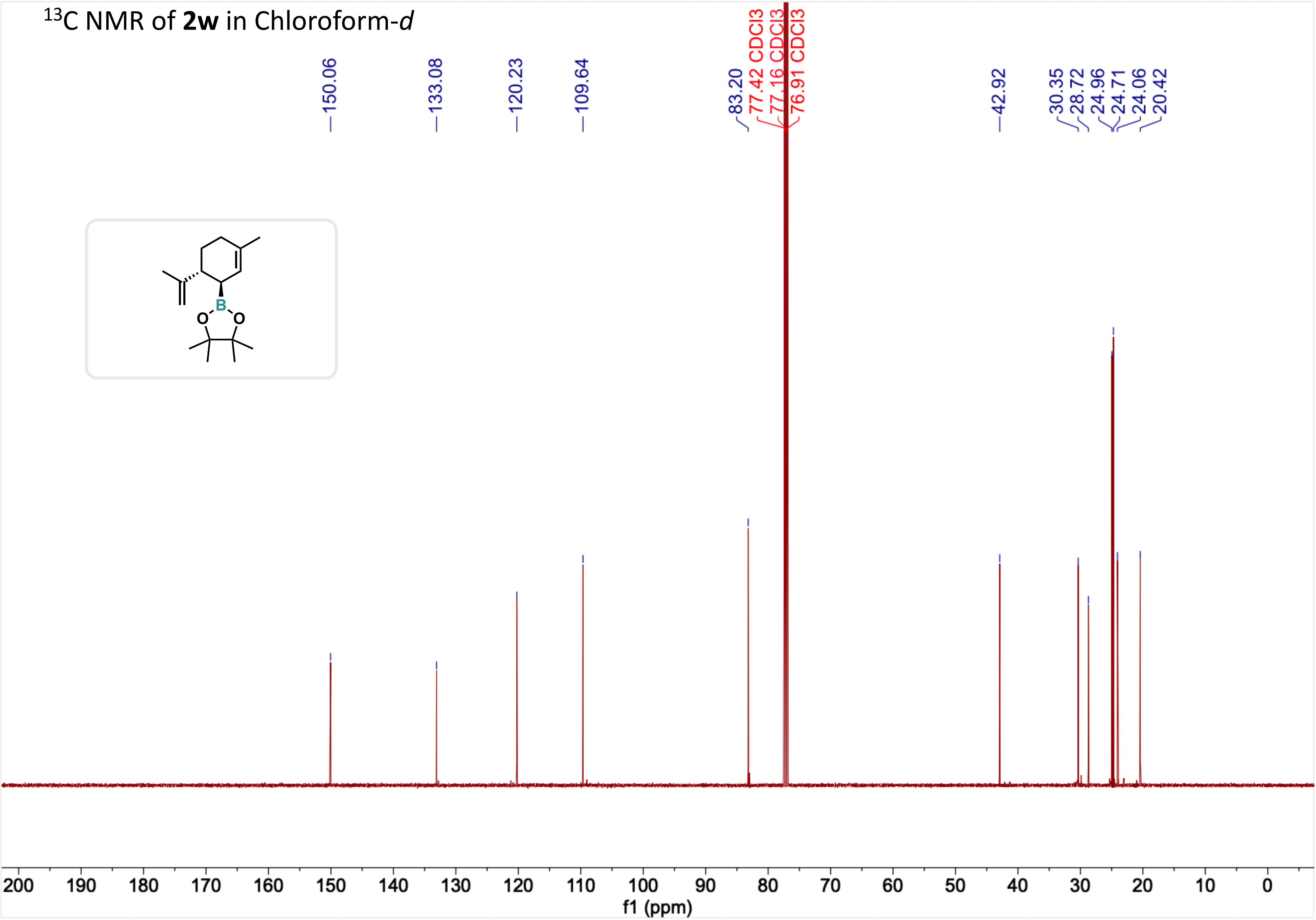

^{11}B NMR of **2w** in Chloroform-*d*

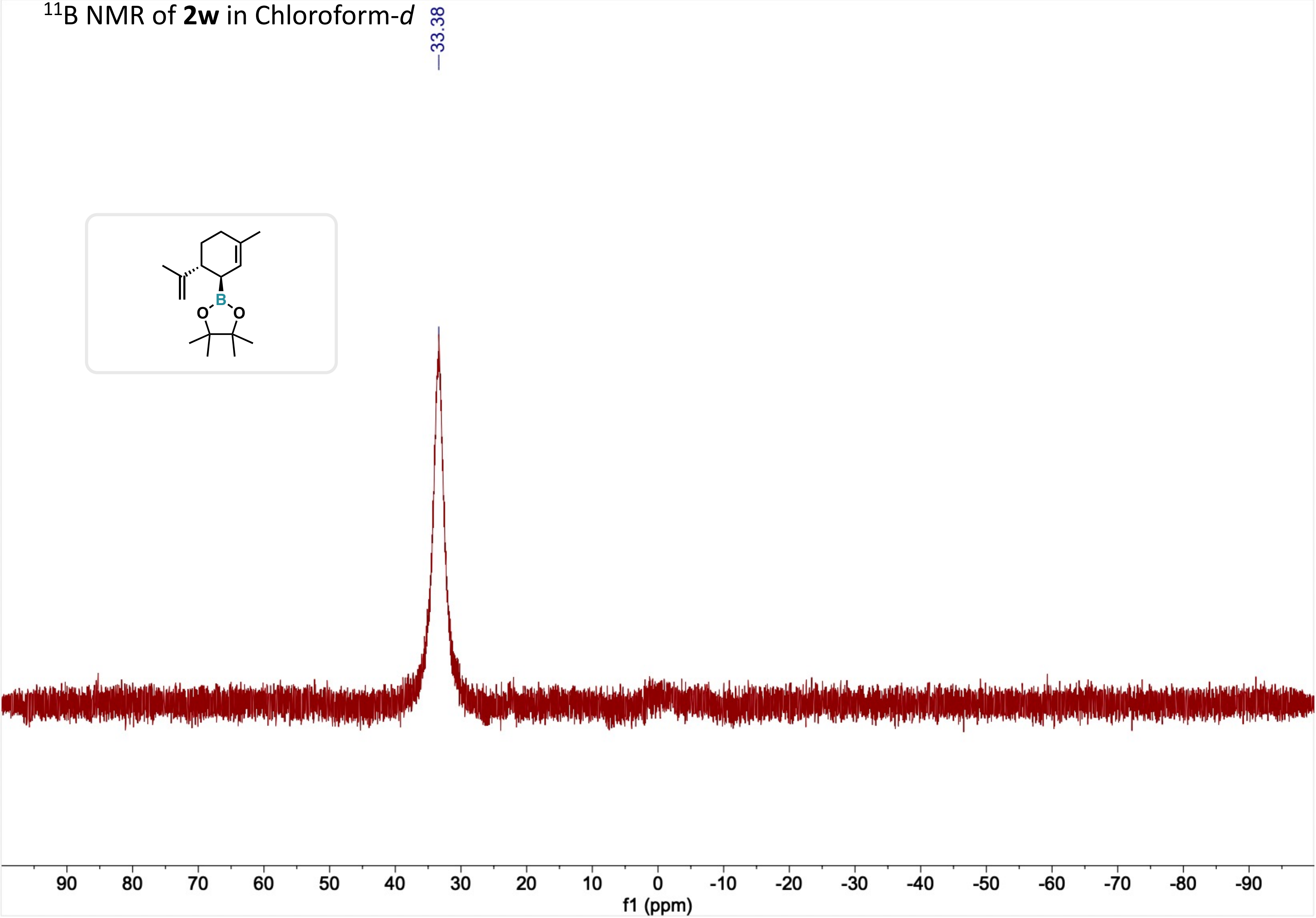

^1H-^1H gCOSY NMR of **2w** in Chloroform-*d*

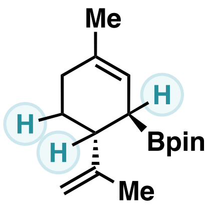

relative configuration determined based on *J* couplings on axial Hs

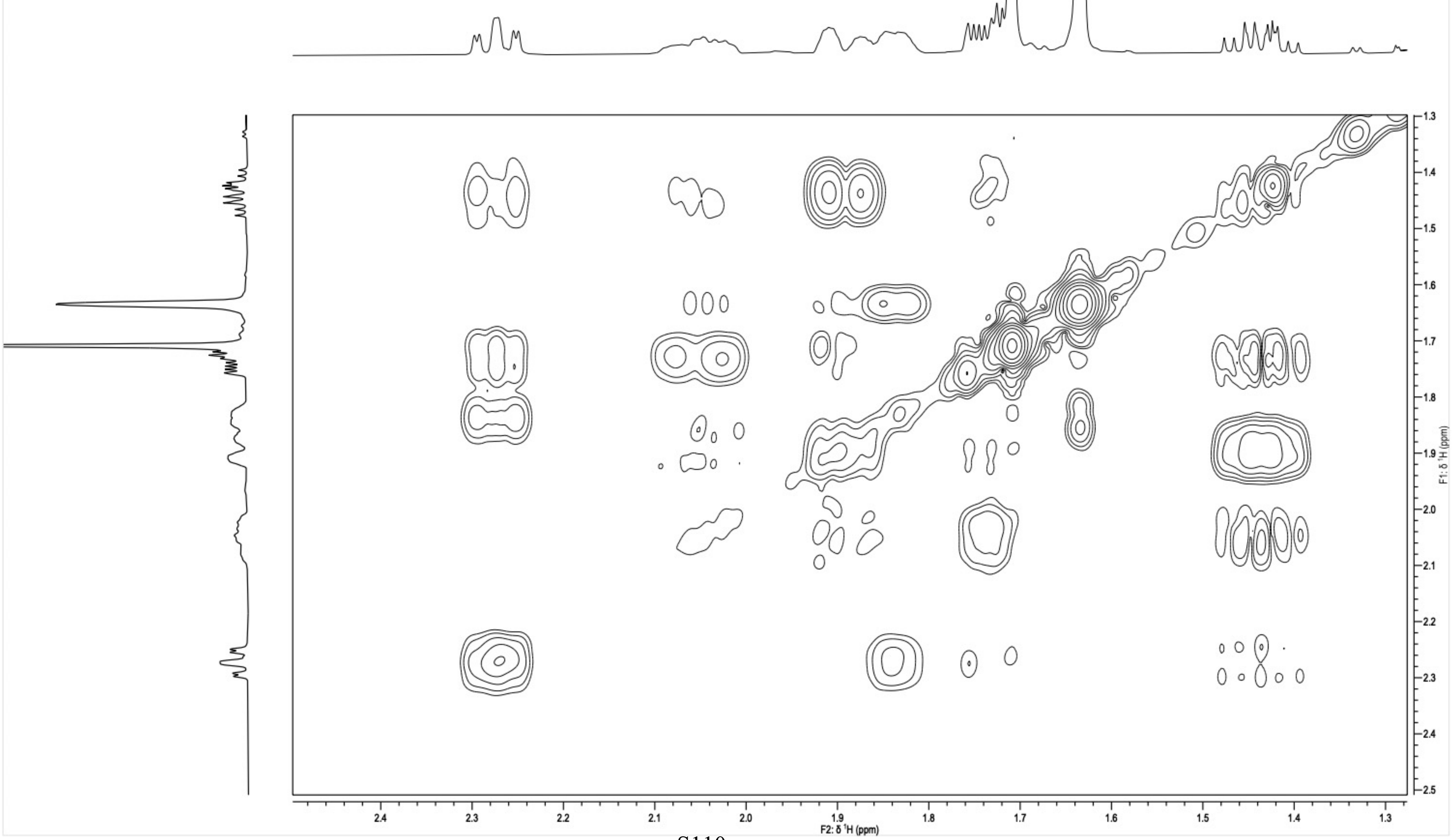

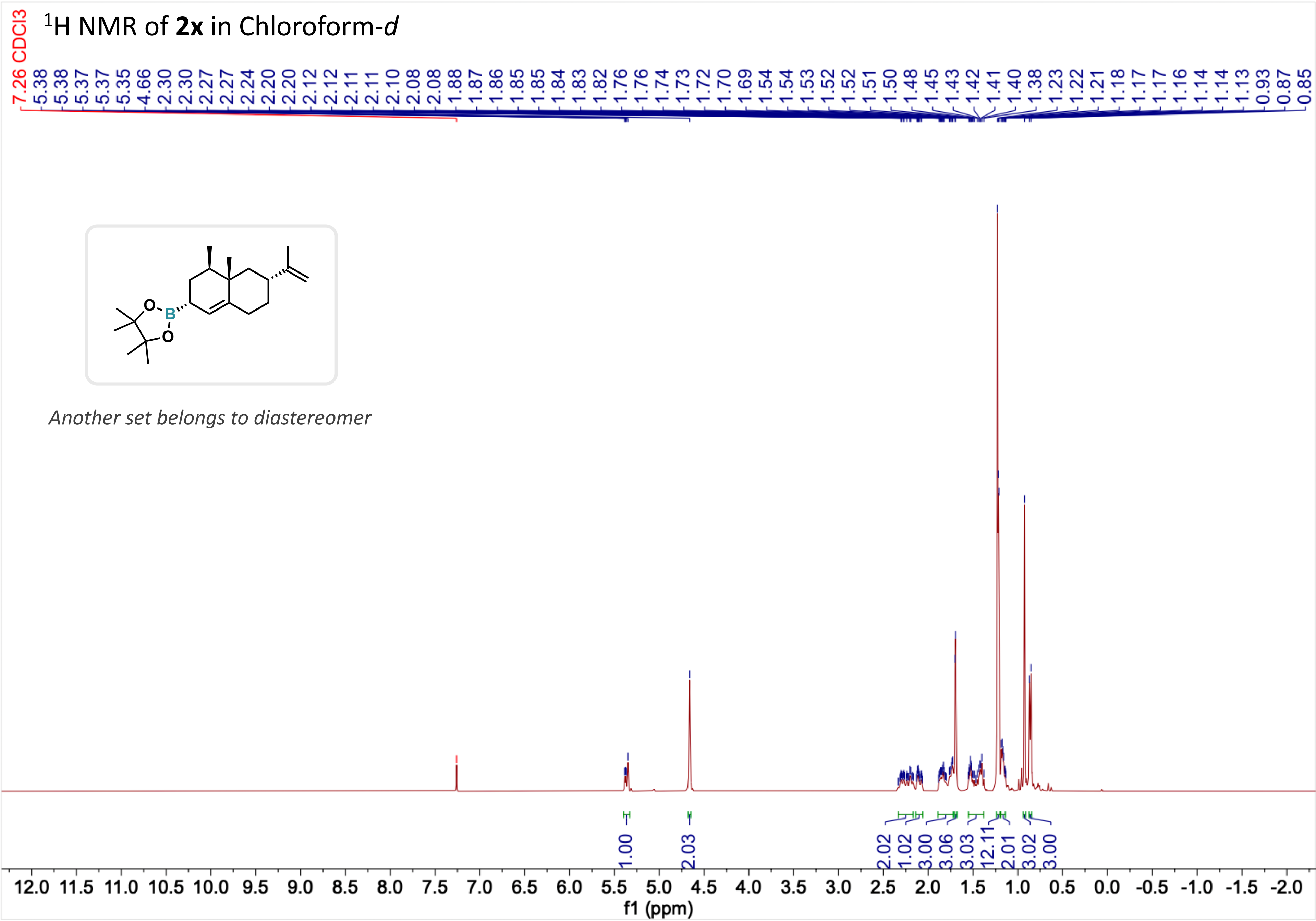

1H NMR of 2x in Chloroform-d
7.26 CDCl3
5.38 5.38 5.37 5.37 5.35 4.66 2.30 2.30 2.27 2.27 2.24 2.20 2.20 2.12 2.12 2.11 2.11 2.10 2.08 2.08 1.88 1.87 1.86 1.85 1.85 1.84 1.83 1.82 1.76 1.76 1.74 1.73 1.72 1.70 1.69 1.54 1.54 1.53 1.52 1.52 1.51 1.50 1.48 1.45 1.43 1.42 1.41 1.40 1.38 1.23 1.22 1.21 1.18 1.17 1.17 1.16 1.14 1.14 1.13 0.93 0.87 0.85
O
B
O
Another set belongs to diastereomer
1.00 2.03 2.02 1.02 3.00 3.06 3.03 12.11 2.01 3.02 3.00
12.0 11.5 11.0 10.5 10.0 9.5 9.0 8.5 8.0 7.5 7.0 6.5 6.0 5.5 5.0 4.5 4.0 3.5 3.0 2.5 2.0 1.5 1.0 0.5 0.0 -0.5 -1.0 -1.5 -2.0
f1 (ppm)

^{13}C NMR of **2x** in Chloroform-*d*

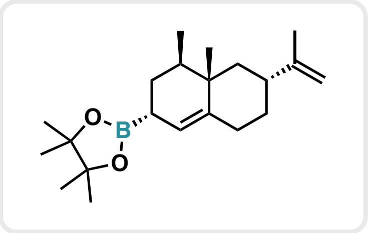

Another set belongs to diastereomer

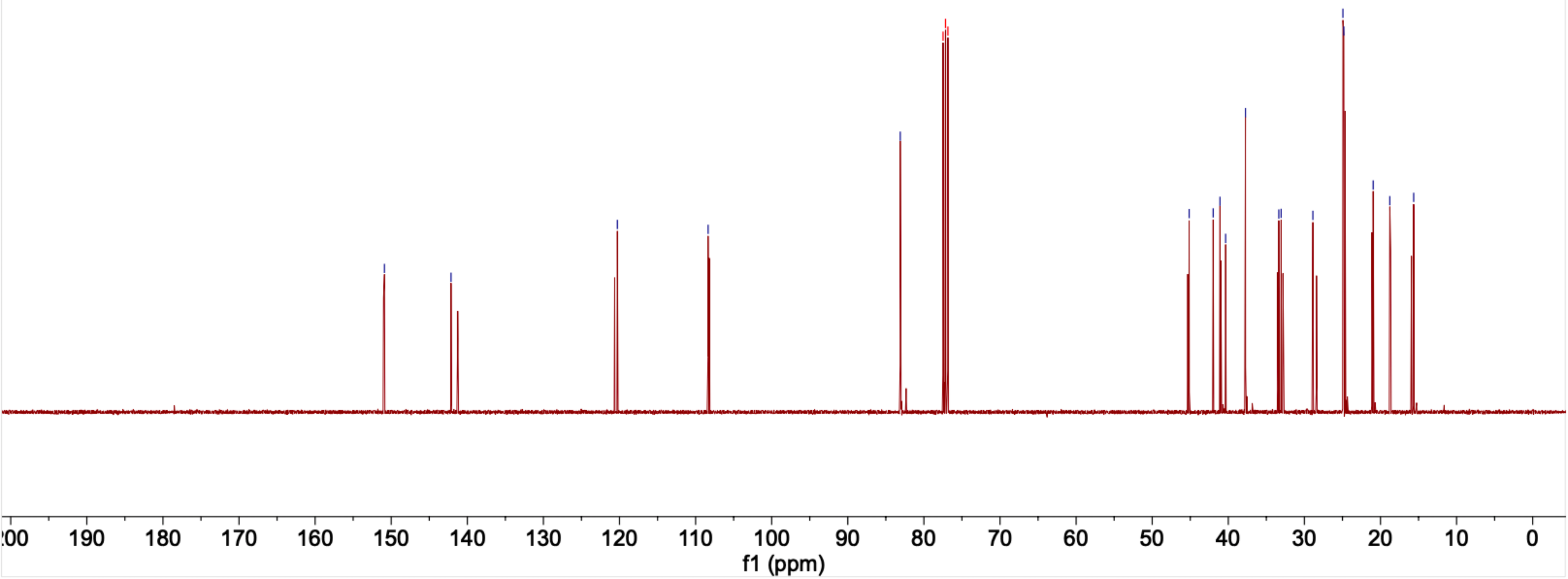

^{11}B NMR of **2x** in Chloroform-*d*

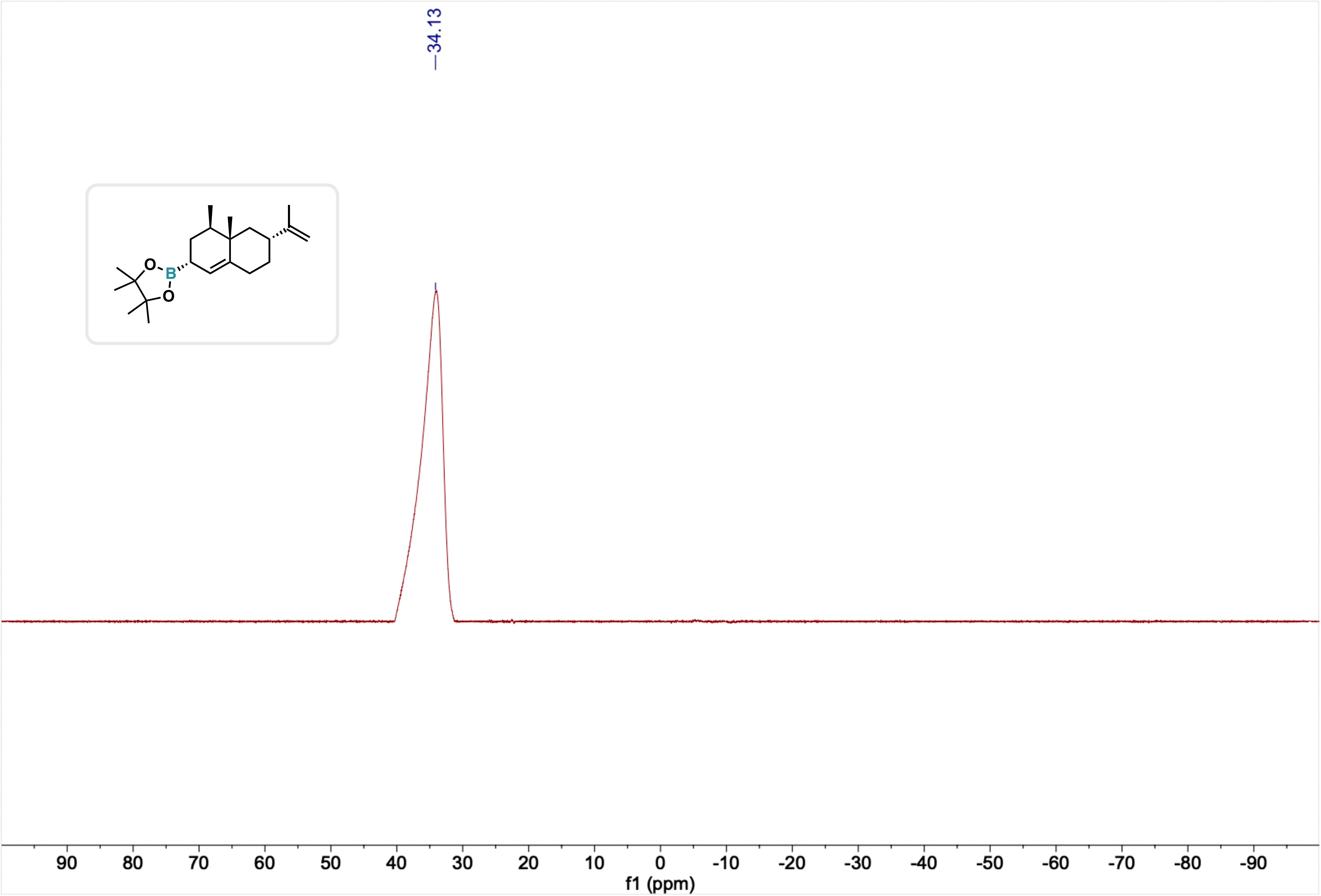

^{1}H NMR of **2y** in Chloroform-*d*

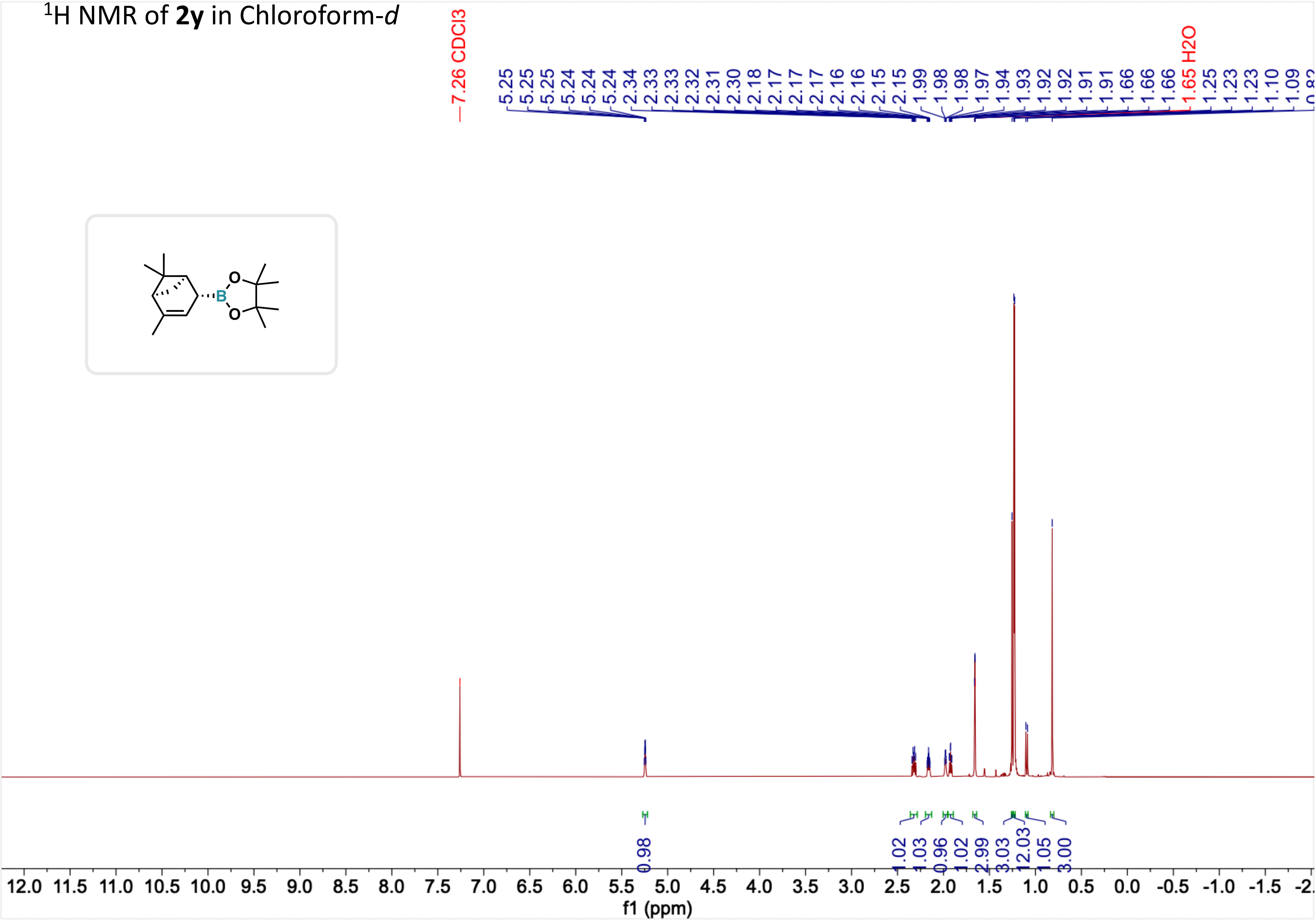

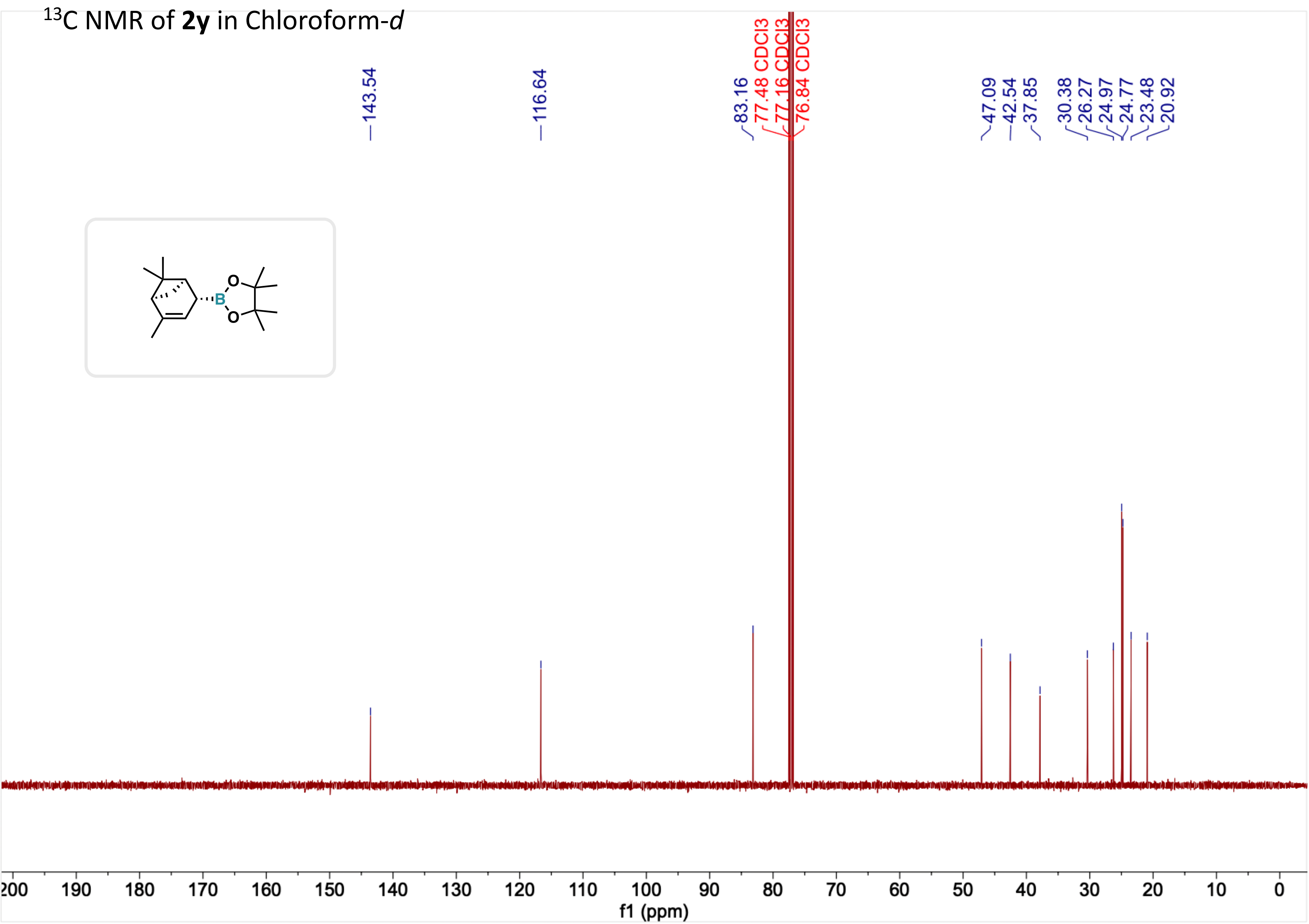

13C NMR of 2y in Chloroform-d
143.54
116.64
83.16
77.48 CDCl3
77.16 CDCl3
76.84 CDCl3
47.09
42.54
37.85
30.38
26.27
24.97
24.77
23.48
20.92
O
B
O
200 190 180 170 160 150 140 130 120 110 100 90 80 70 60 50 40 30 20 10 0
f1 (ppm)

^{11}B NMR of **2y** in Chloroform-*d*

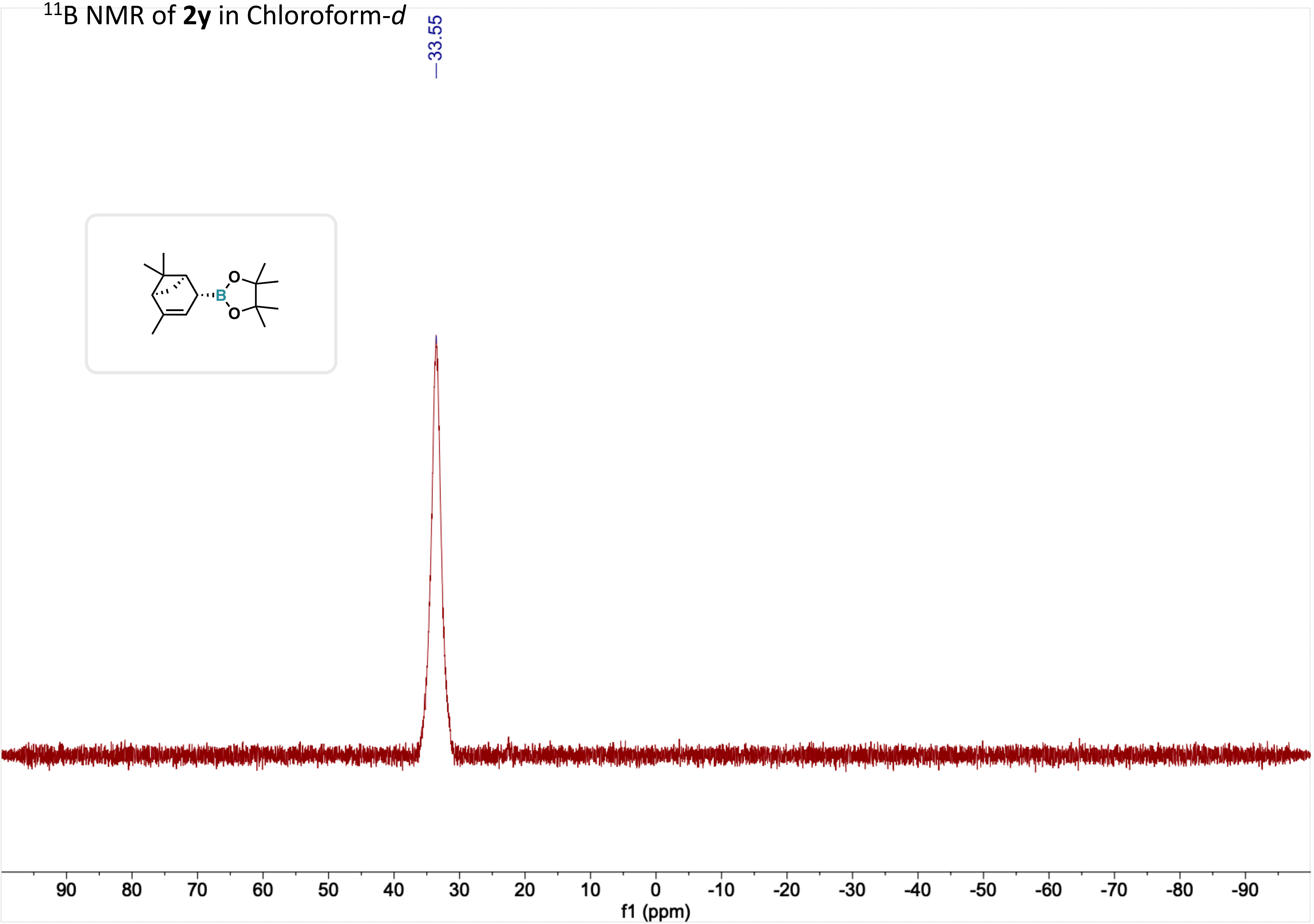

^{1}H NMR of **2z** in Chloroform-*d*

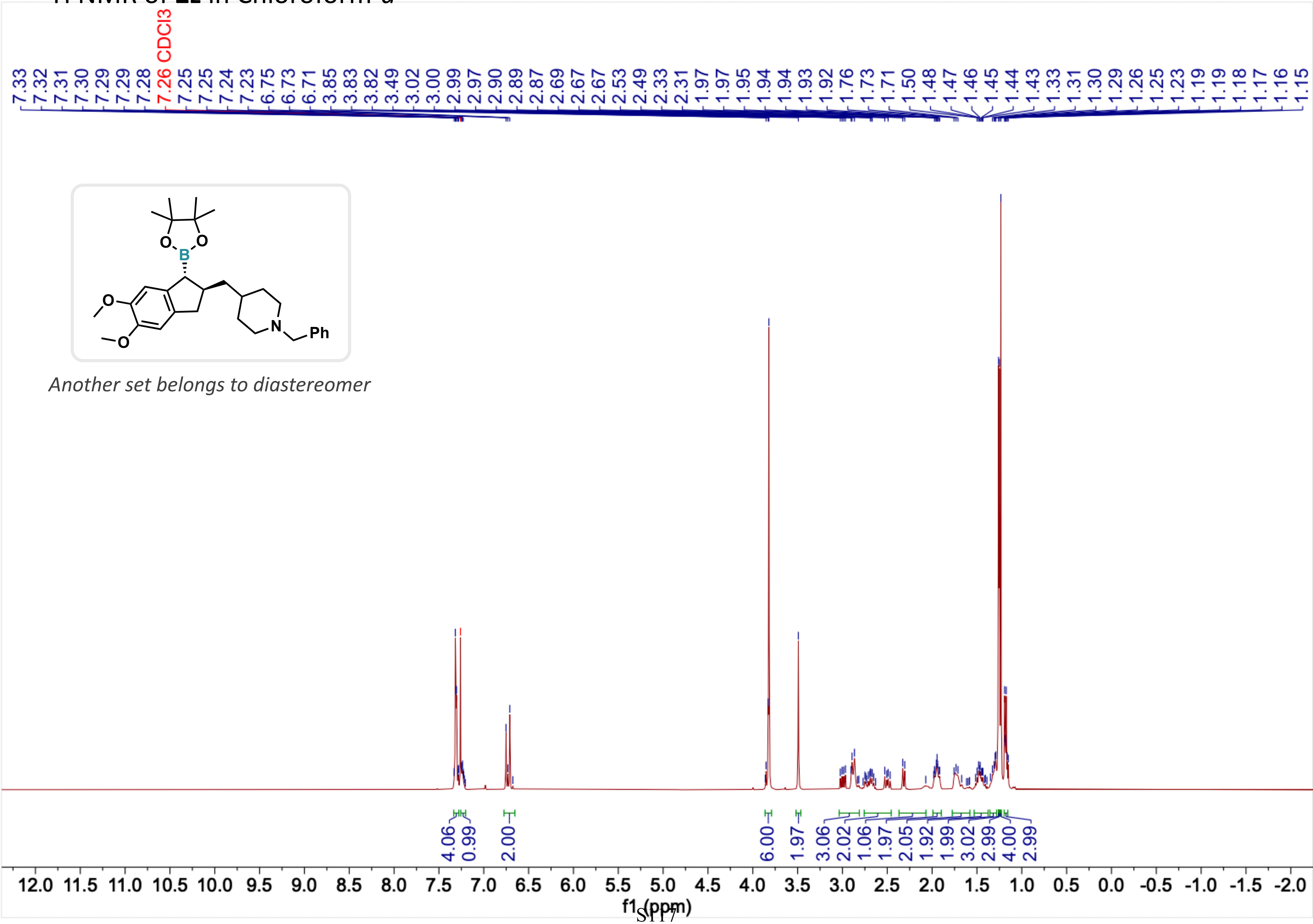

^{13}C NMR of **2z** in Chloroform-*d*

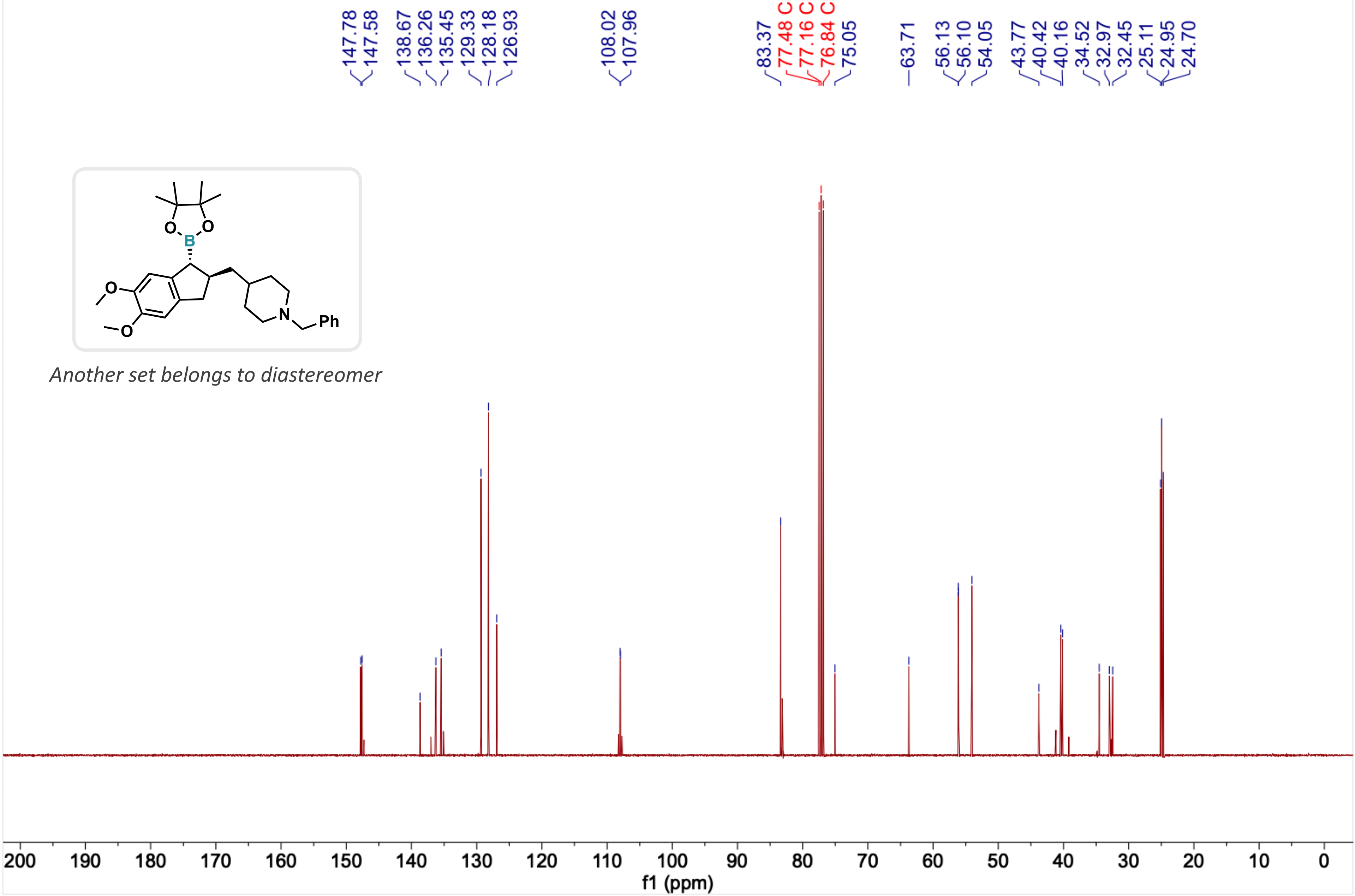

^{11}B NMR of **2z** in Chloroform-*d*

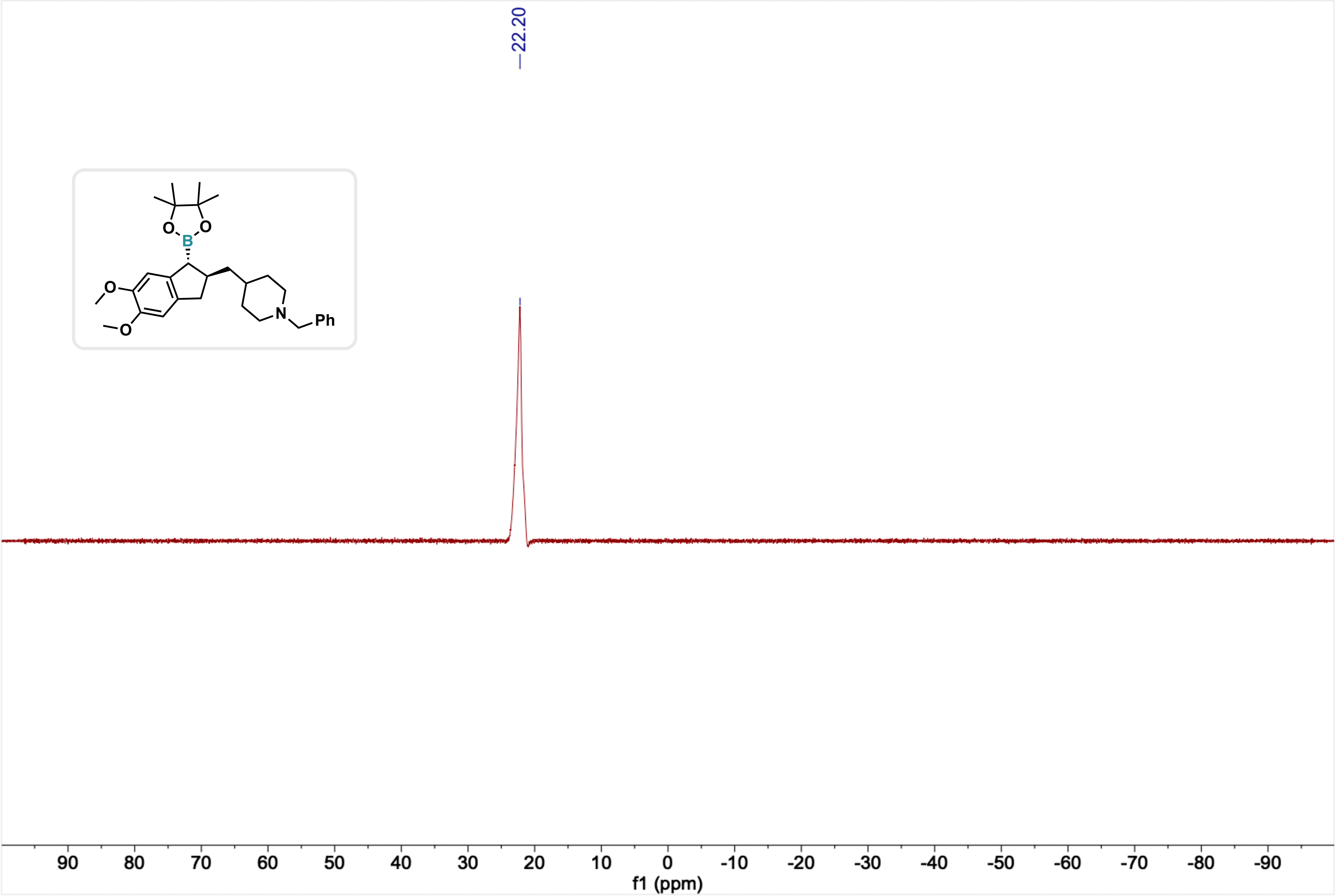

^{1}H NMR of **2aa** in Chloroform-*d*

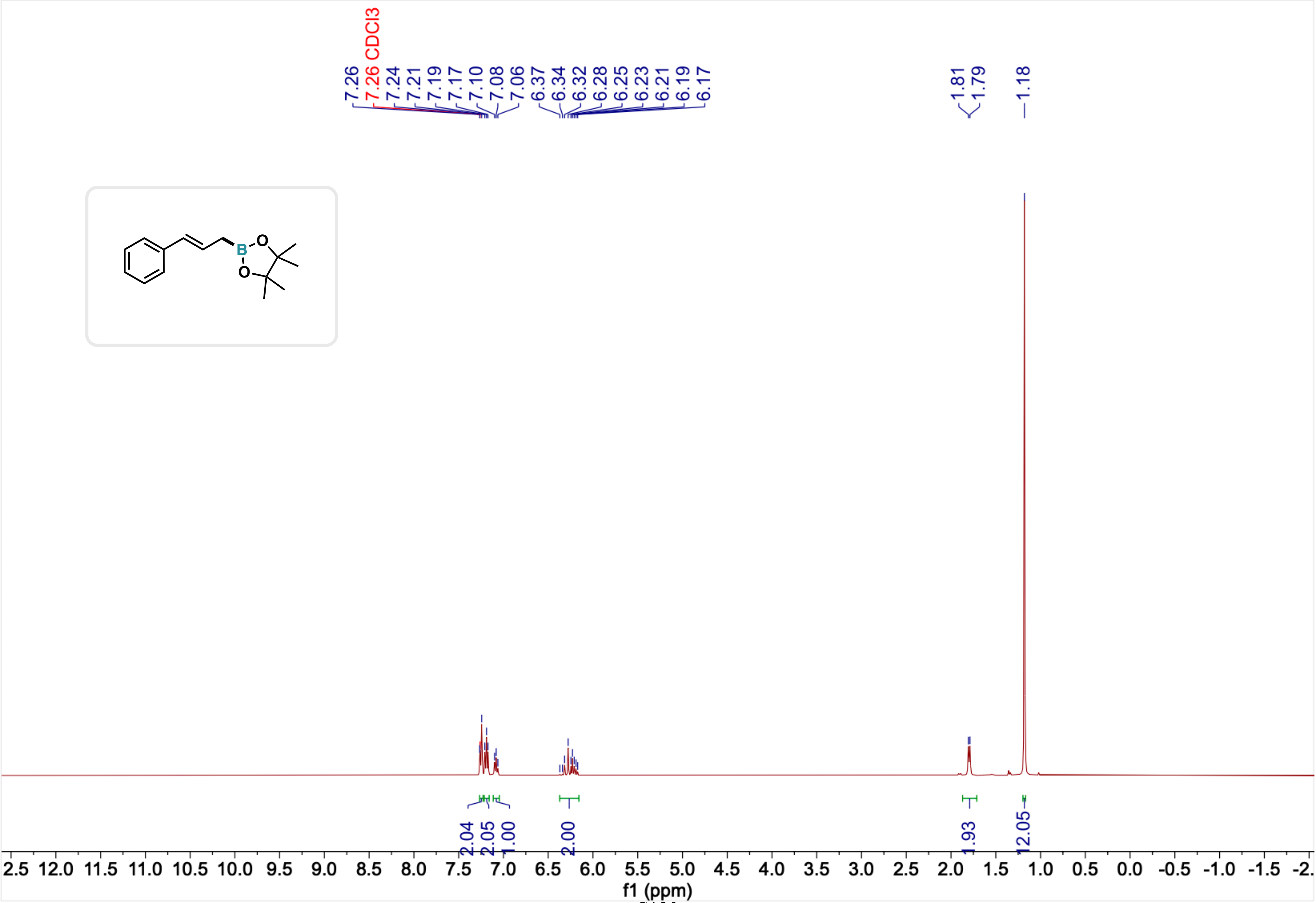

^{13}C NMR of **2aa** in Chloroform-*d*

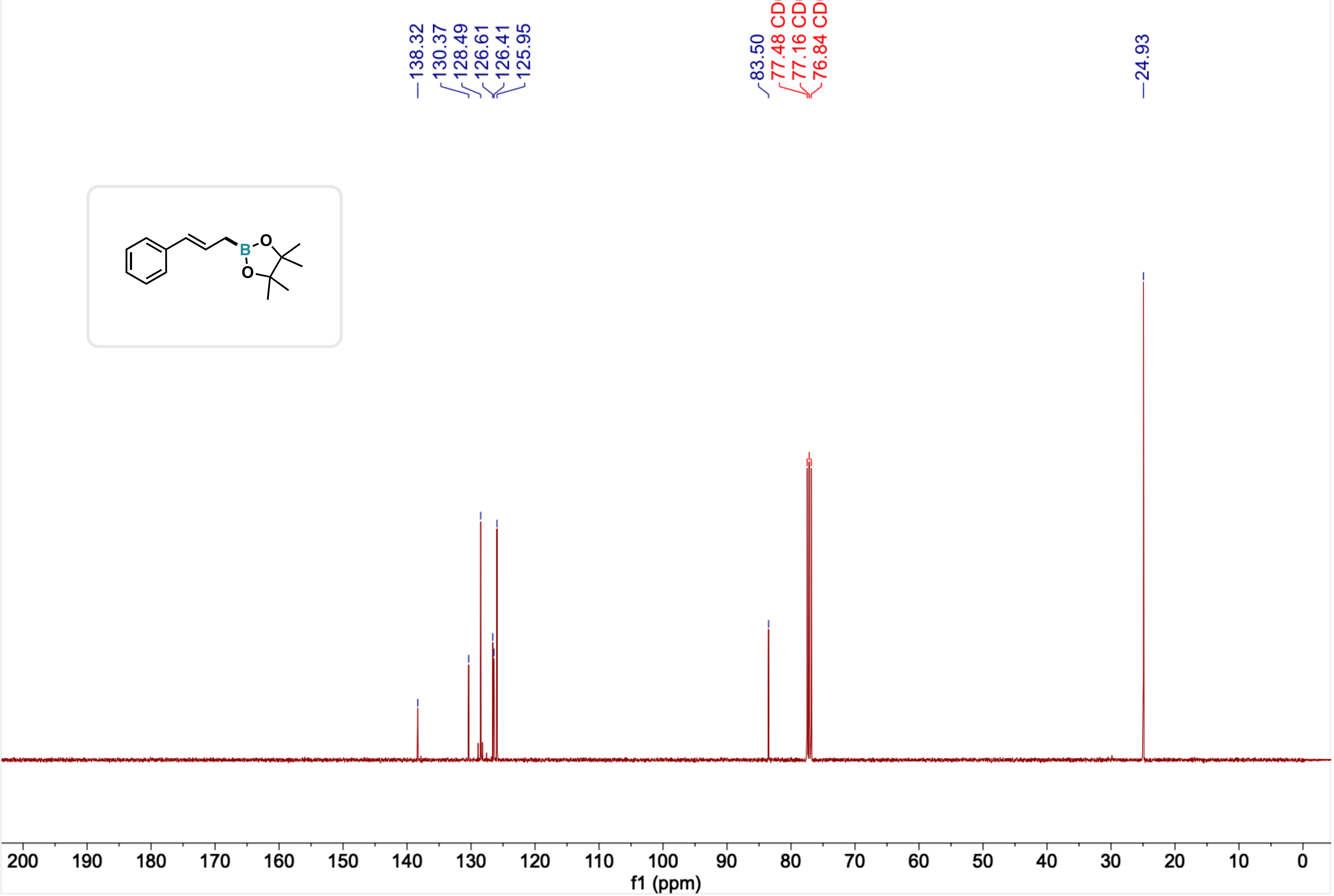

^{11}B NMR of **2aa** in Chloroform-*d*

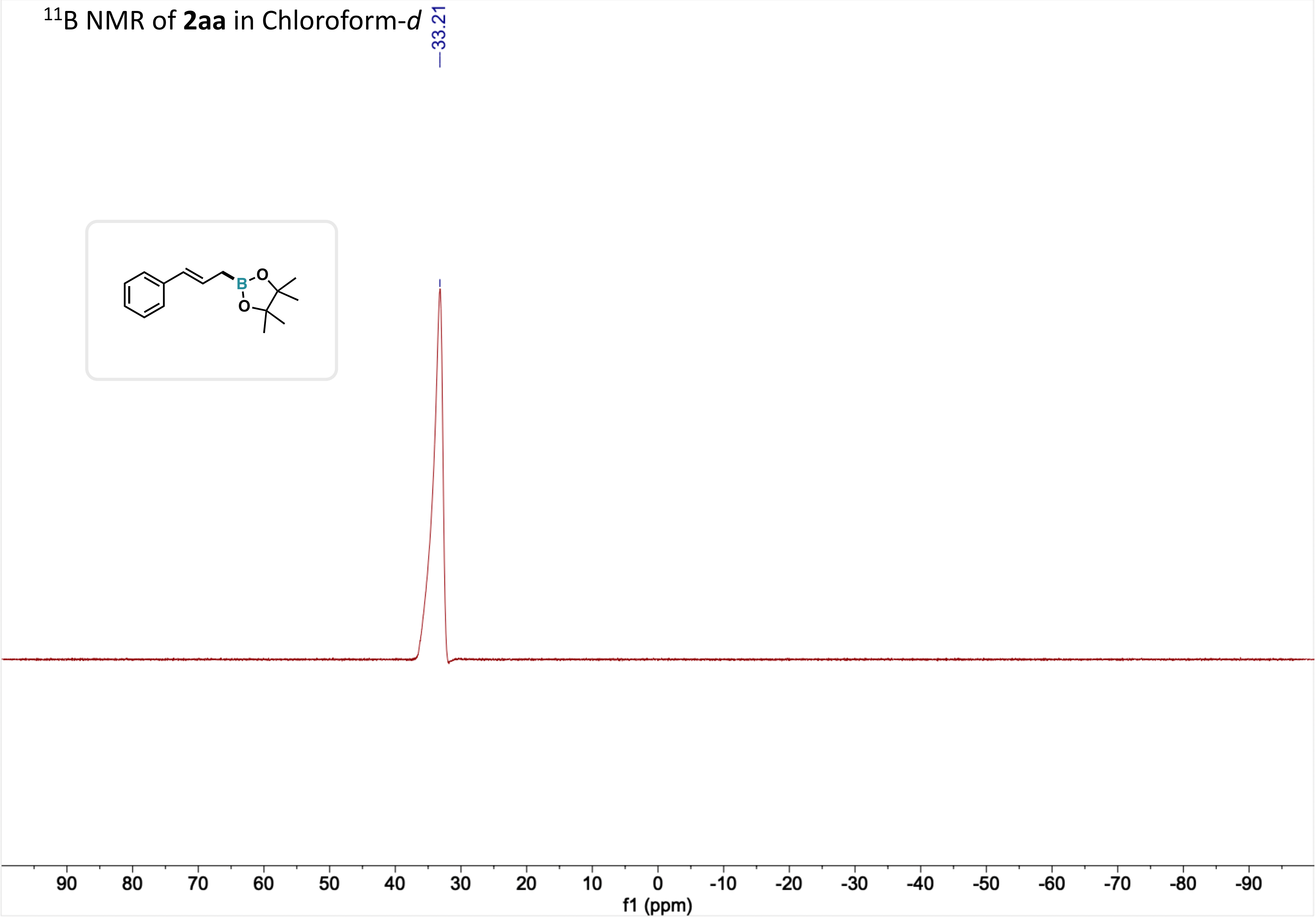

^{1}H NMR of **2ab** in Chloroform-*d*

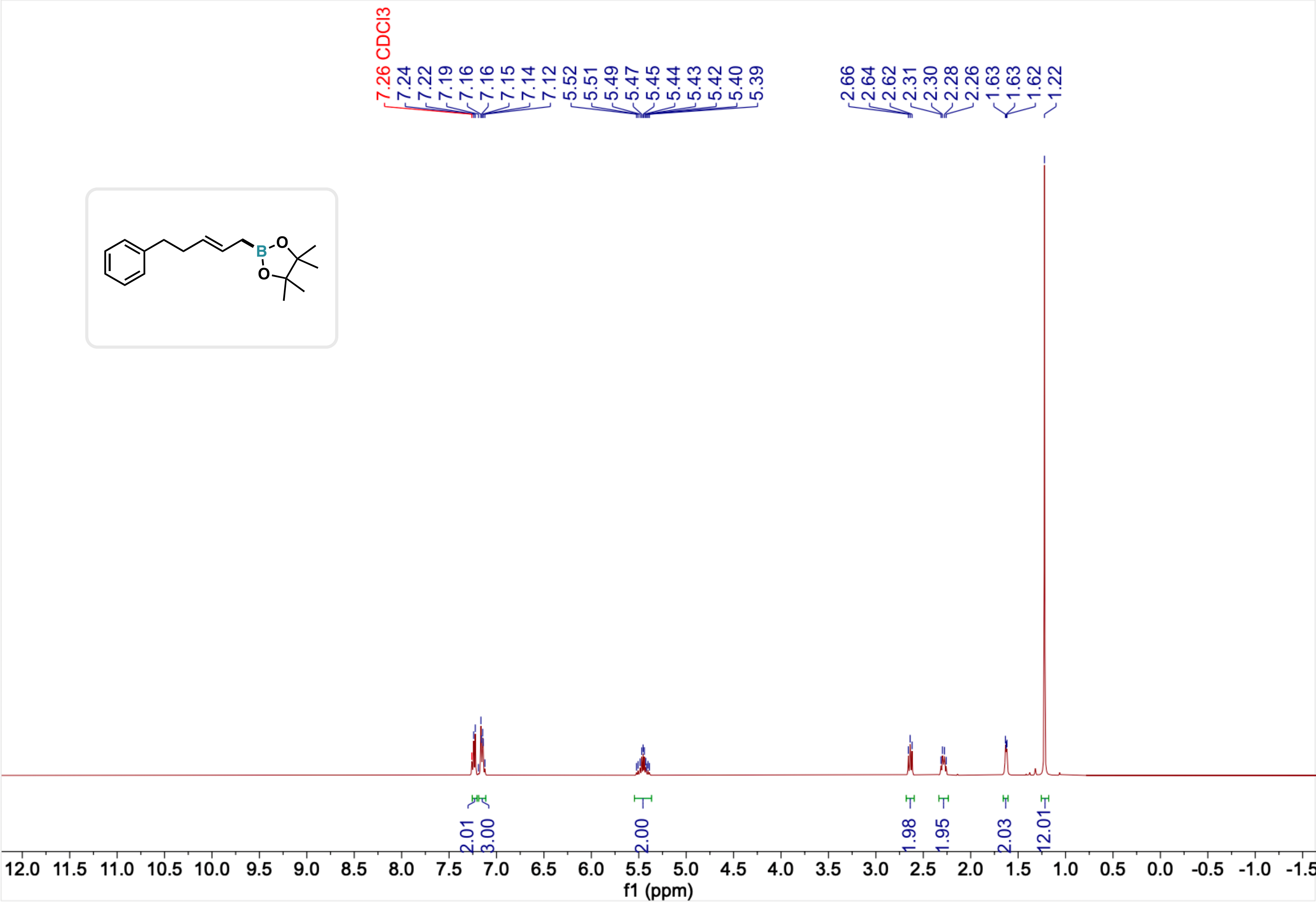

^{13}C NMR of **2ab** in Chloroform-*d*

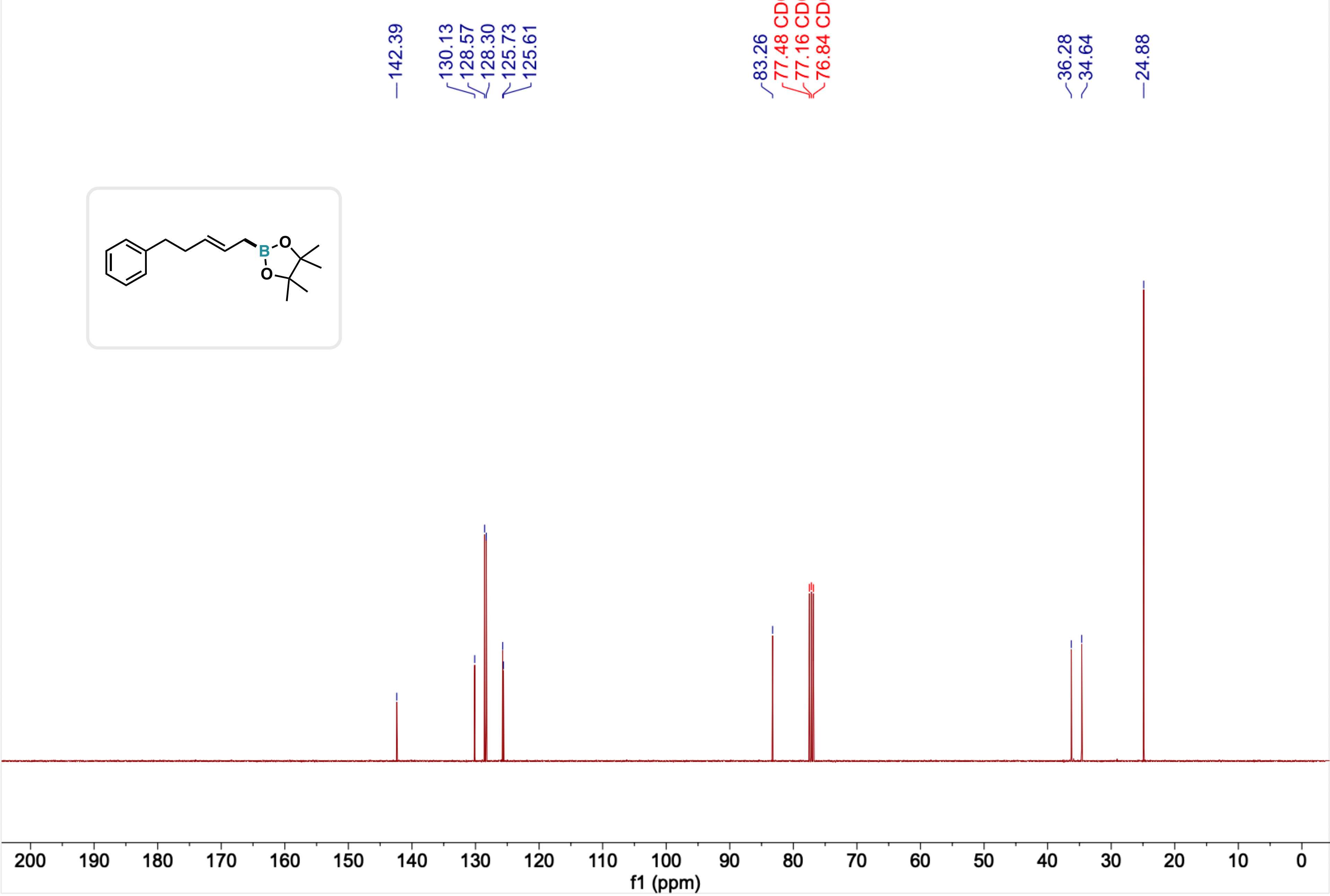

^{11}B NMR of **2ab** in Chloroform-*d*

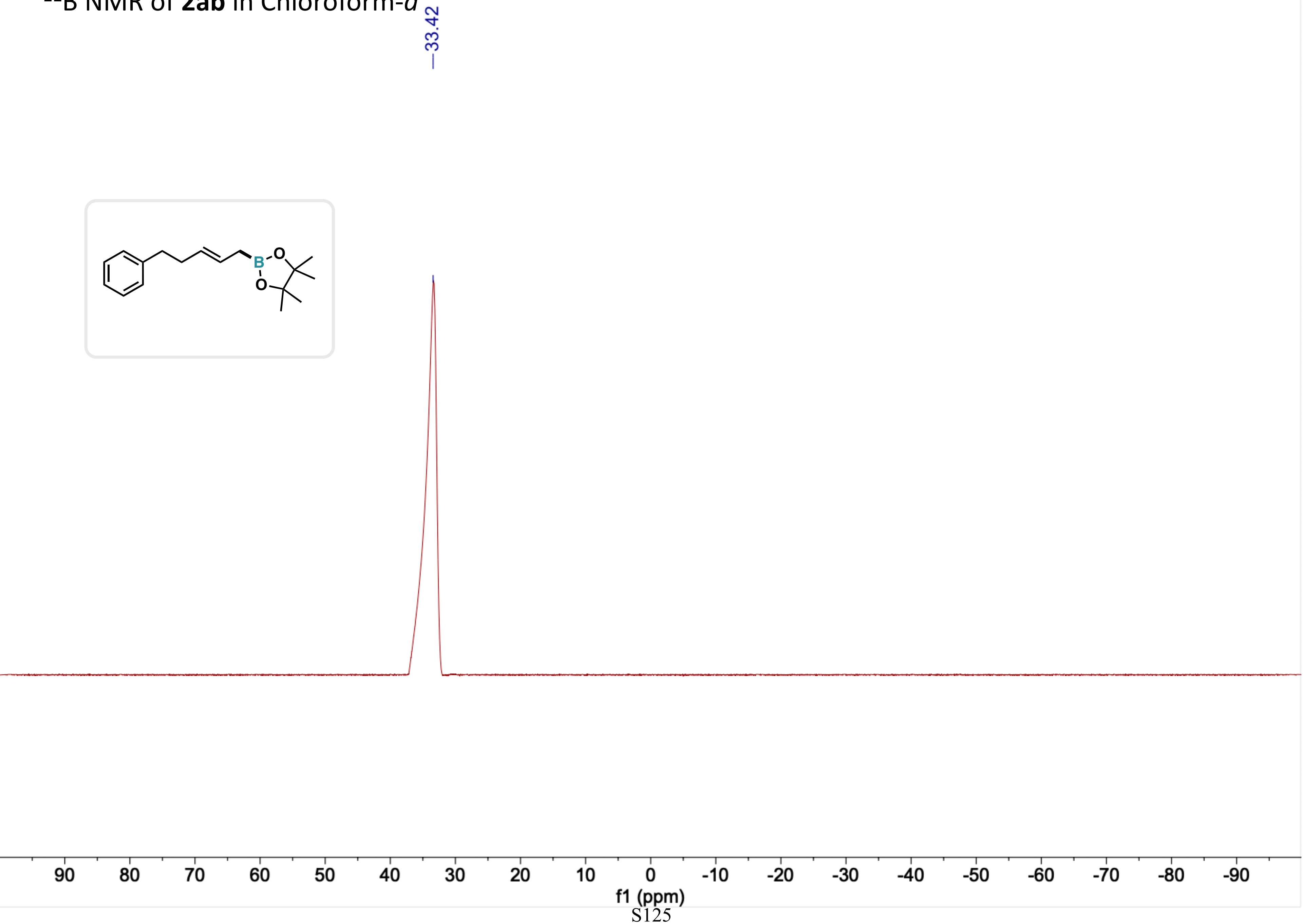

^{1}H NMR of **2ac** in Chloroform-*d*

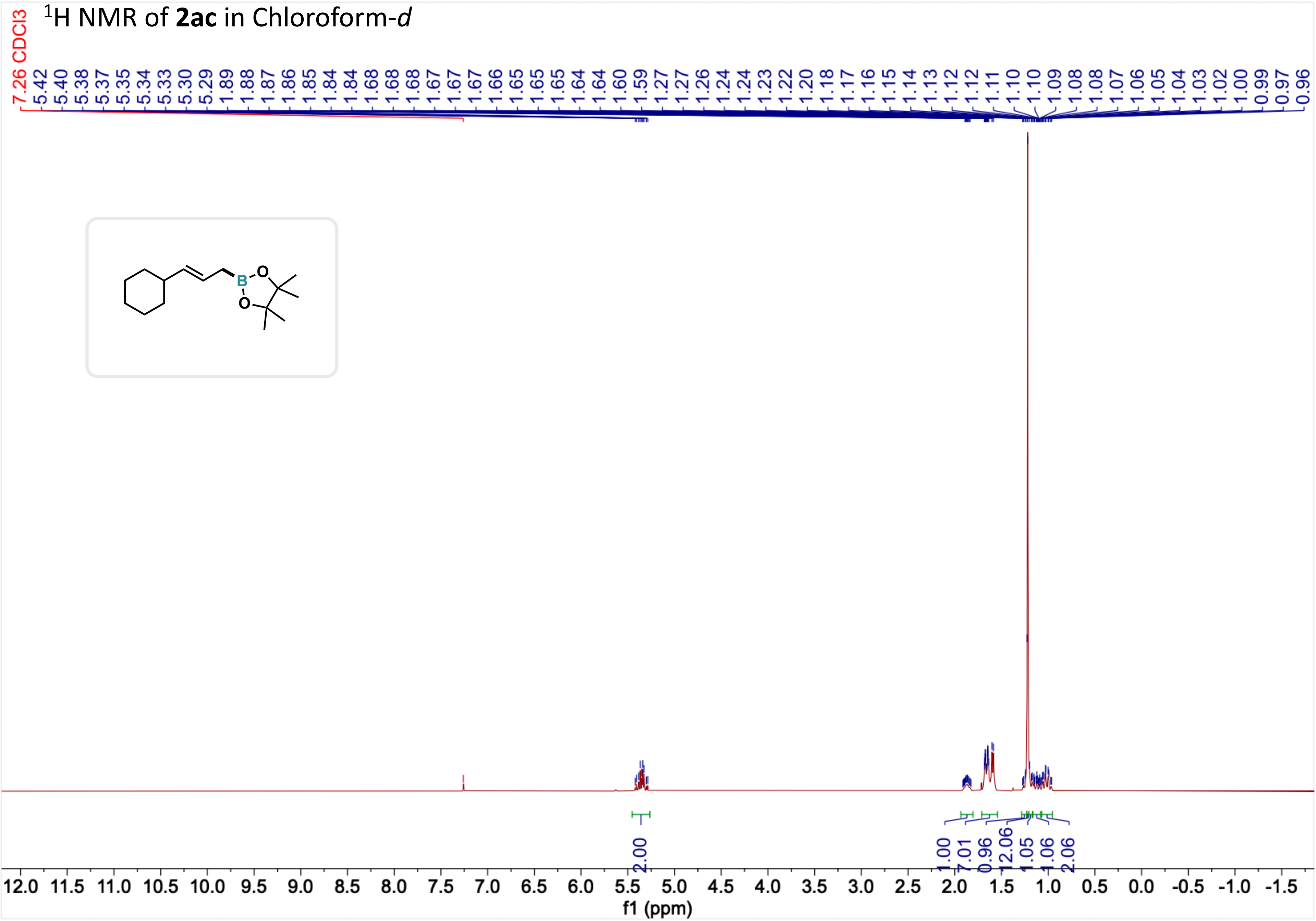

^{13}C NMR of **2ac** in Chloroform-*d*

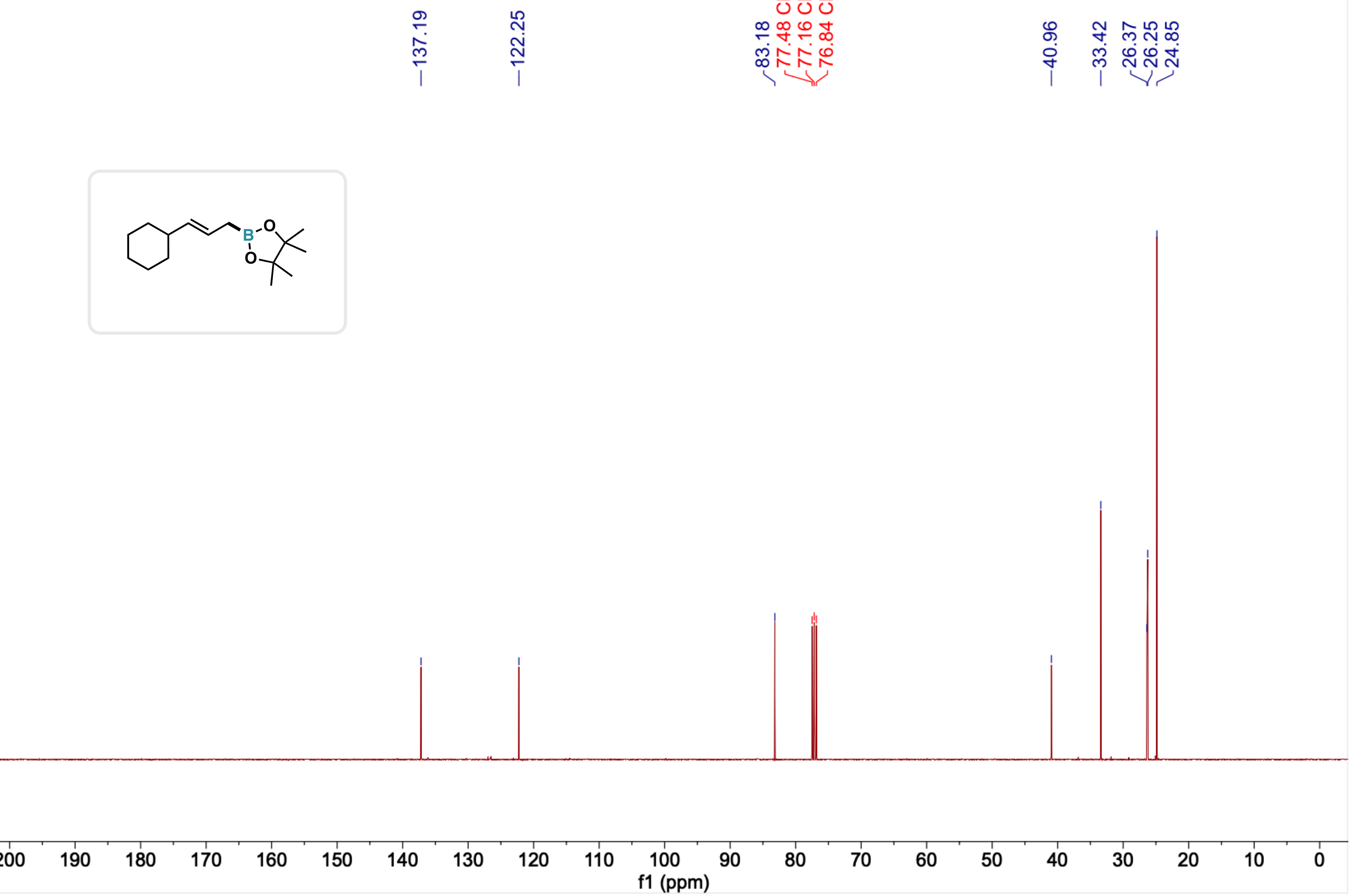

^{11}B NMR of **2ac** in Chloroform-*d*

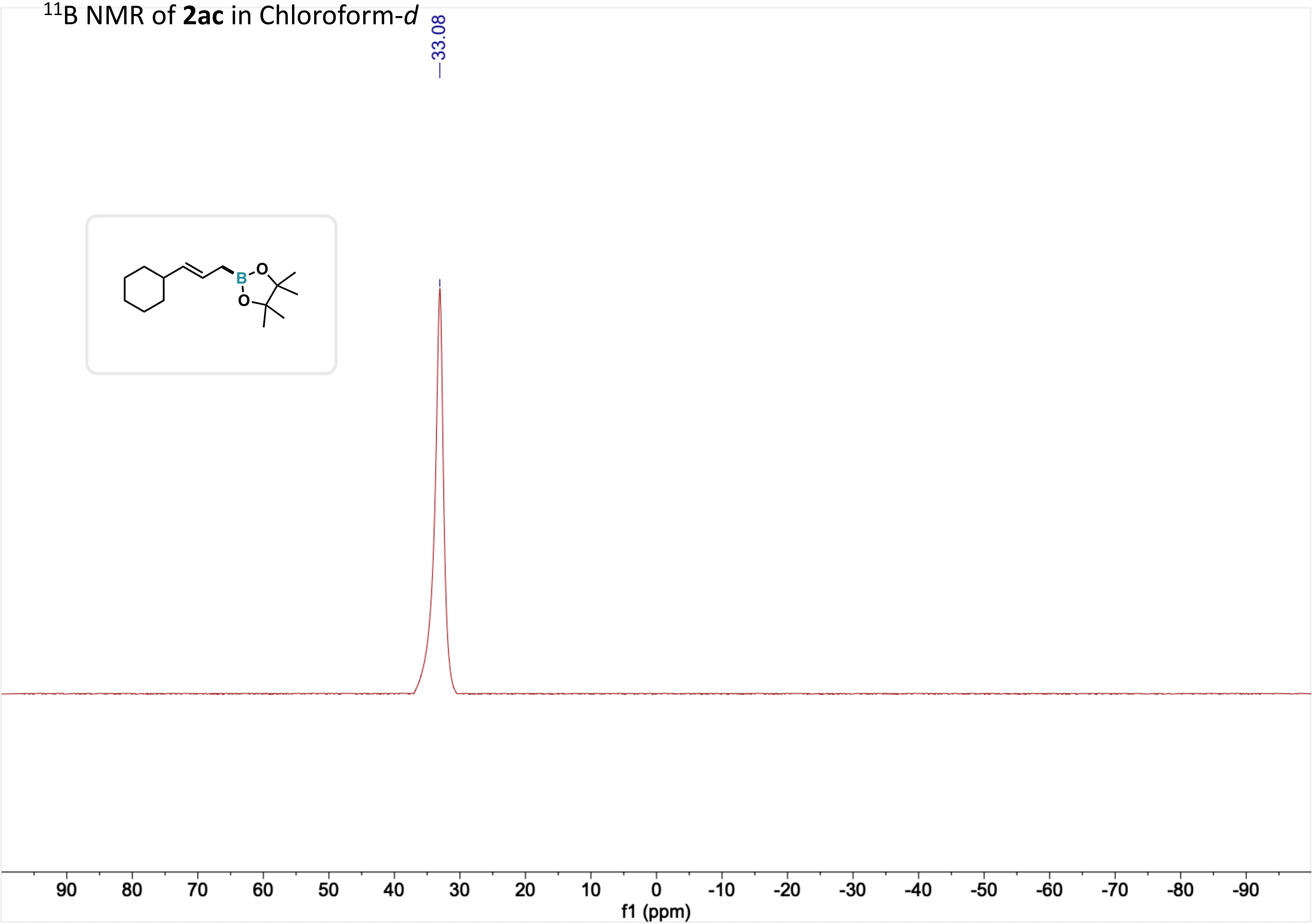

^{1}H NMR of **2ad** in Chloroform-*d*

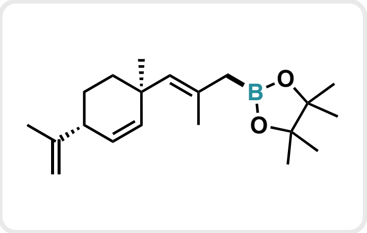

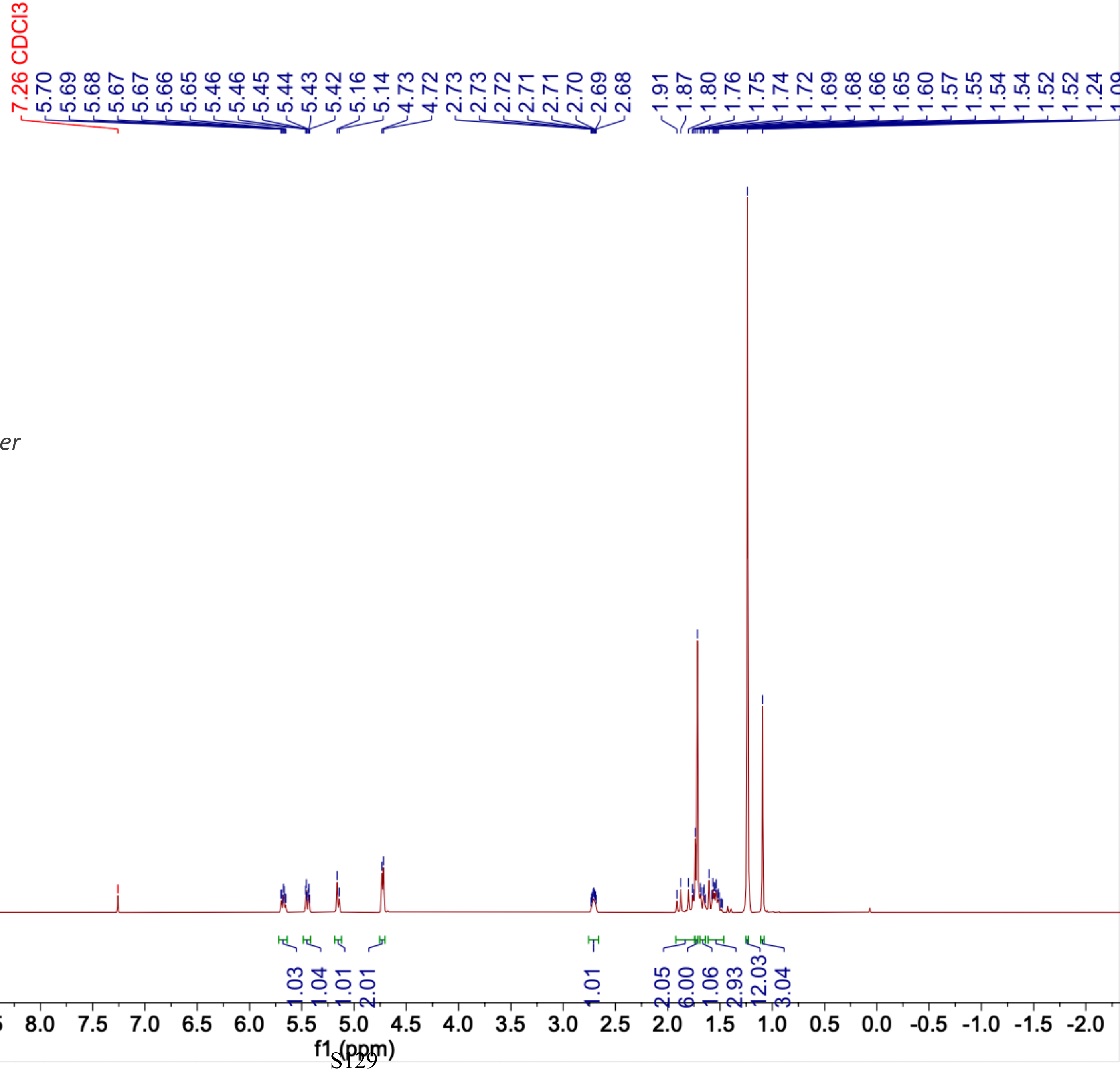

Another set belongs to diastereomer

^{13}C NMR of **2ad** in Chloroform-*d*

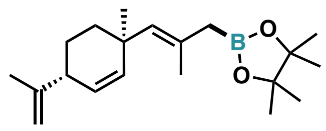

Another set belongs to diastereomer

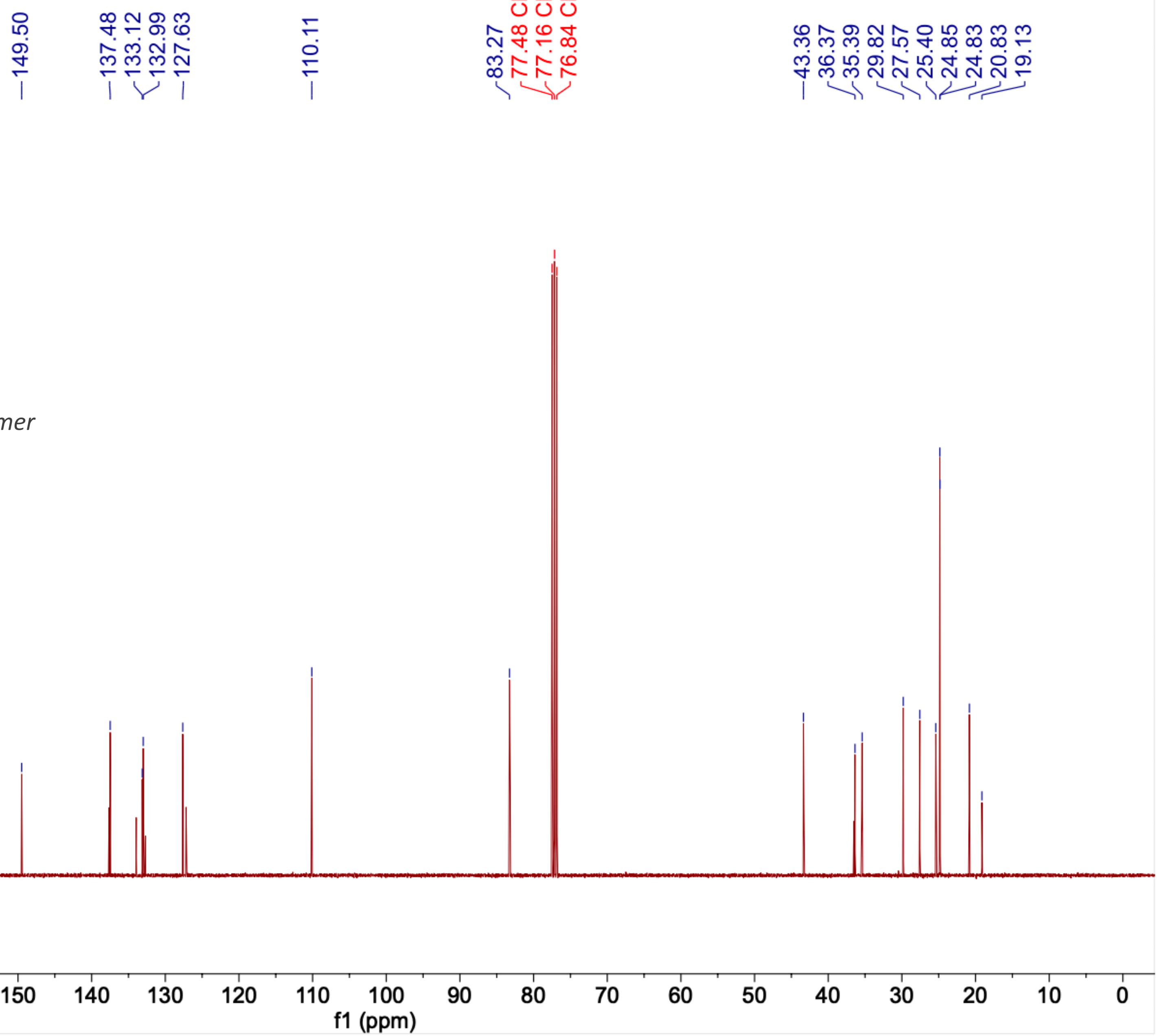

^{11}B NMR of **2ad** in Chloroform-*d*

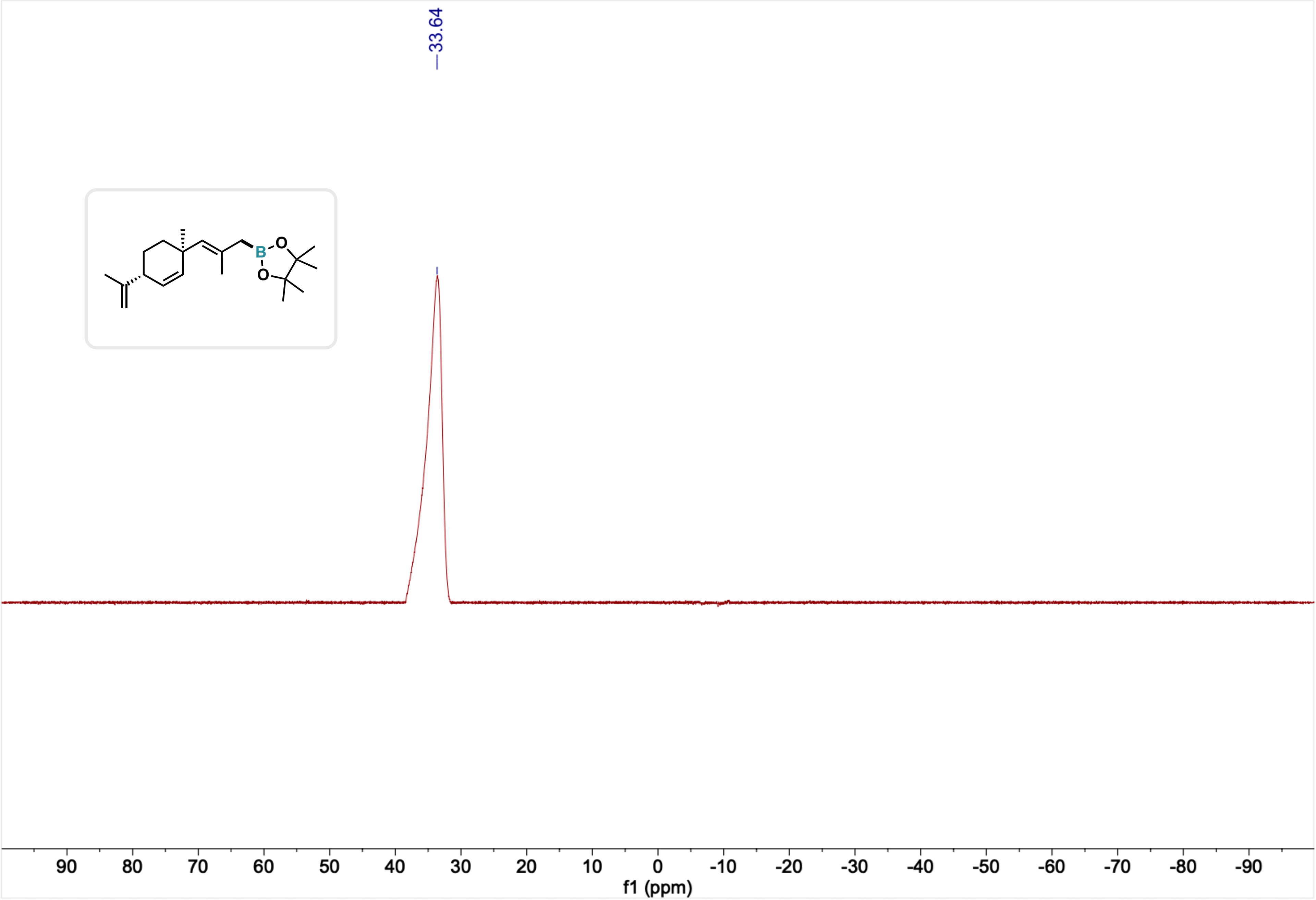

^{1}H NMR of **3a** in Chloroform-*d*

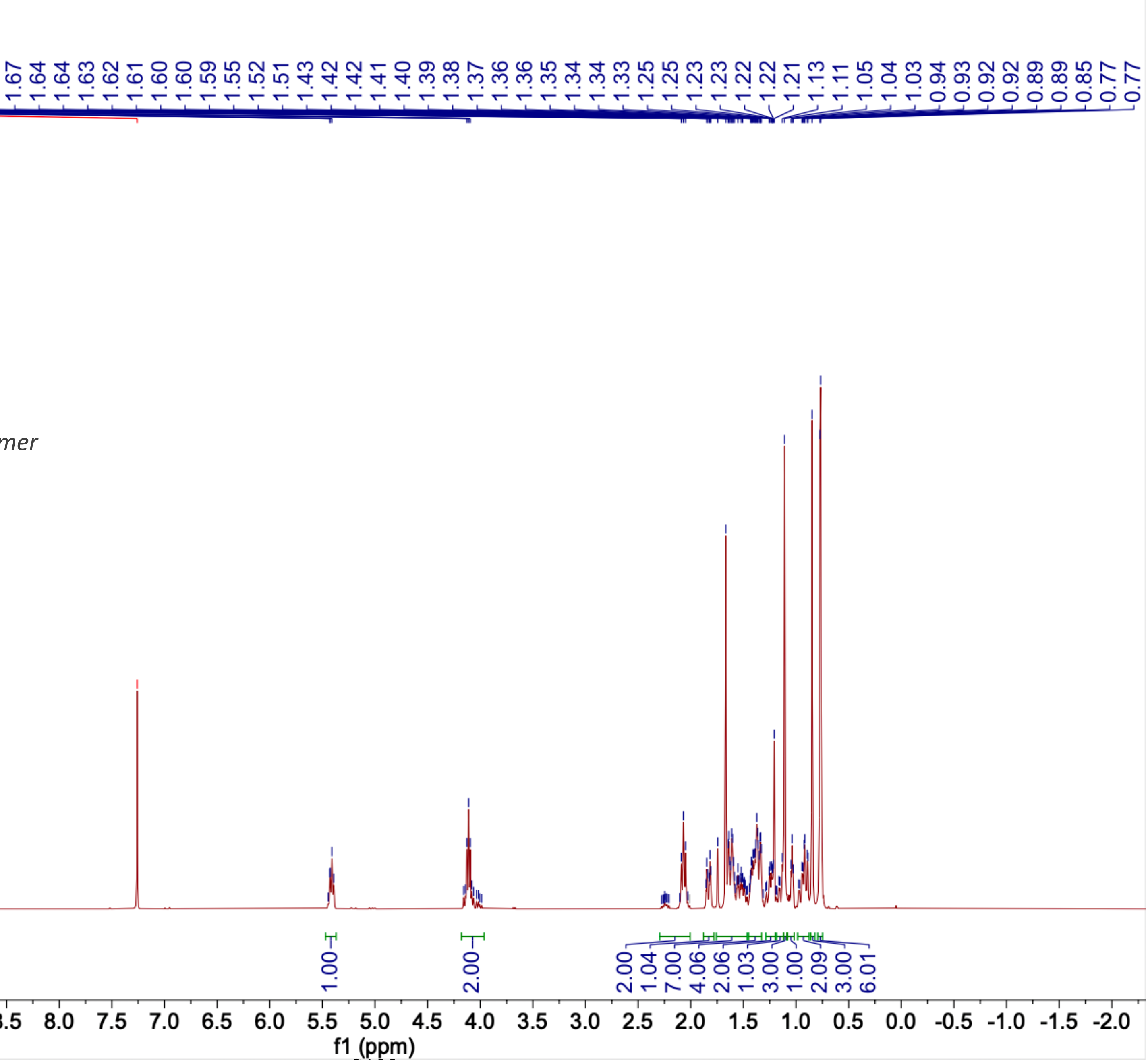

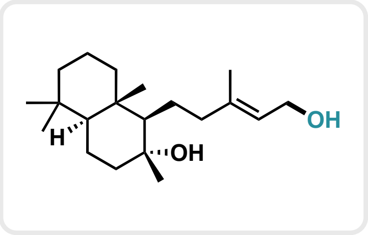

Another set belongs to diastereomer

^{13}C NMR of **3a** in Chloroform-*d*

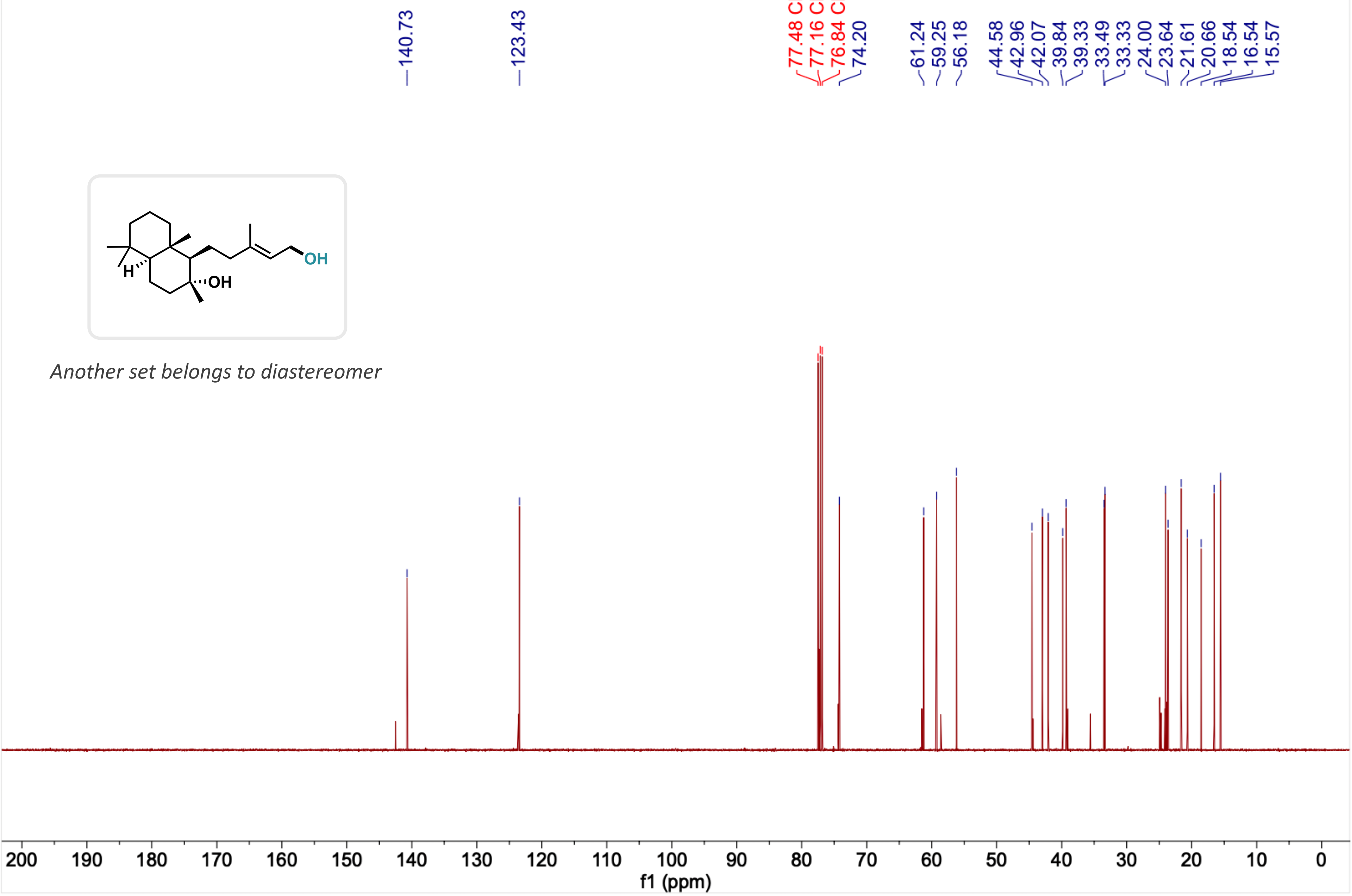

^{1}H NMR of **3b** in Chloroform-*d*

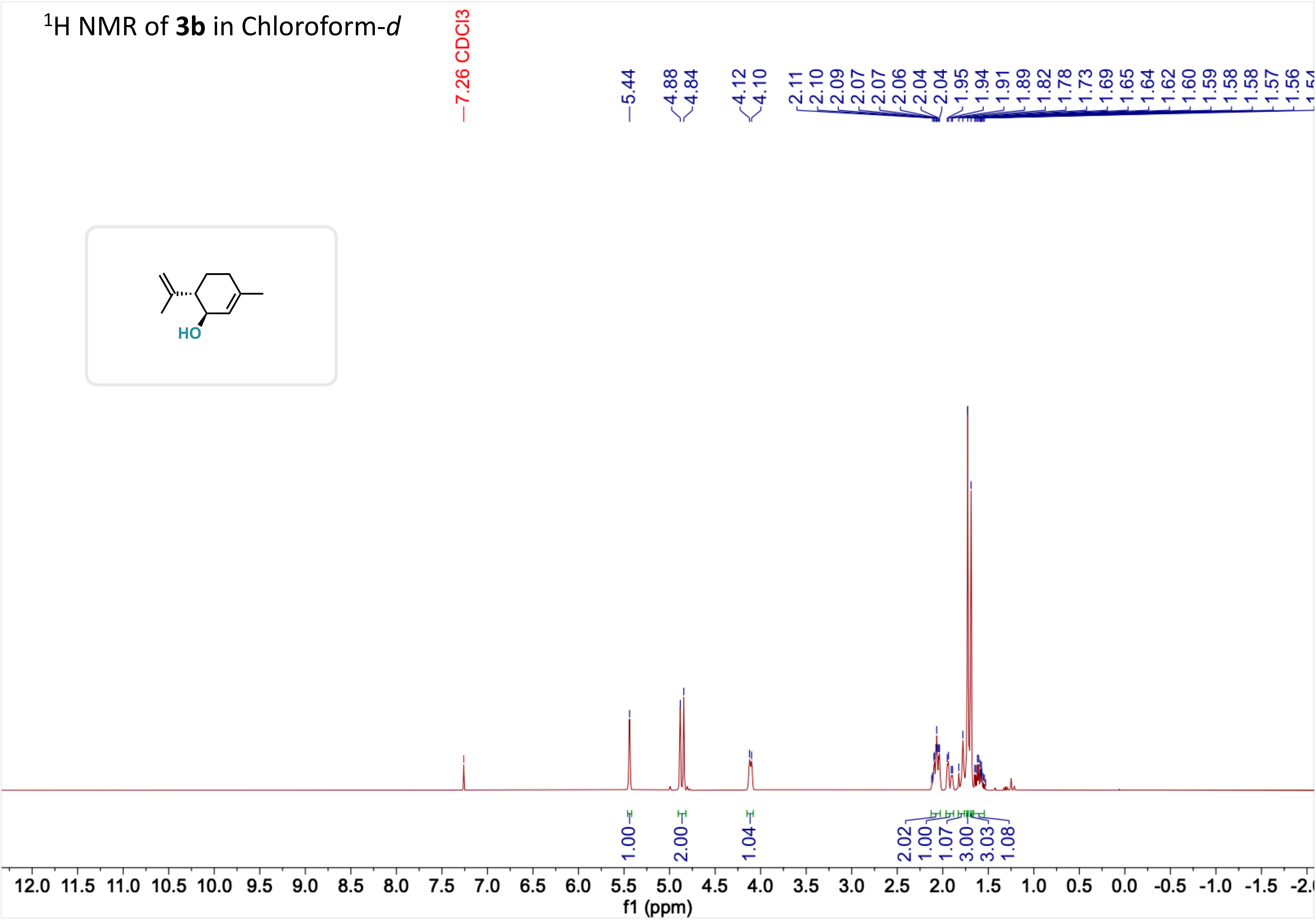

^{13}C NMR of **3b** in Chloroform-*d*

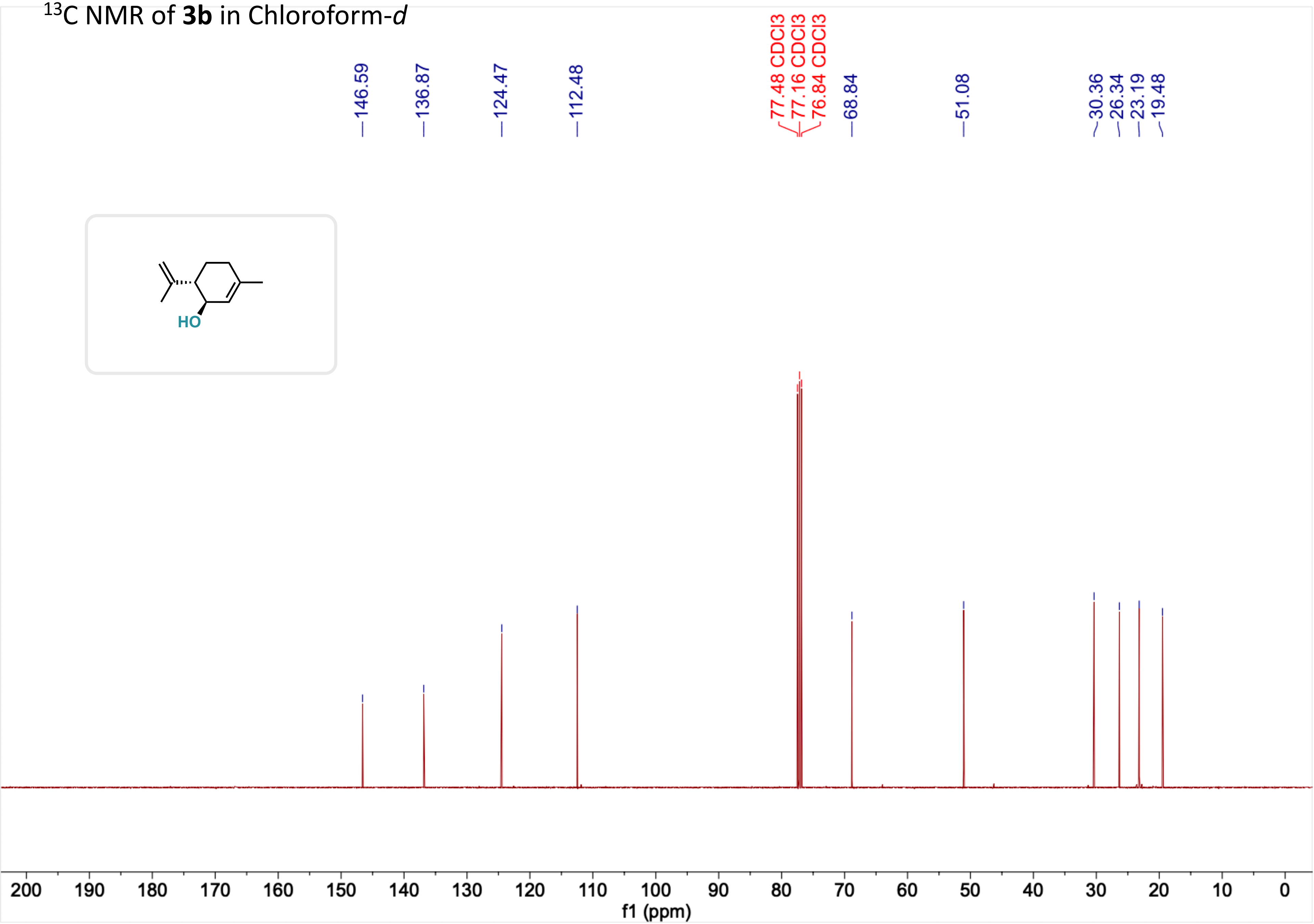

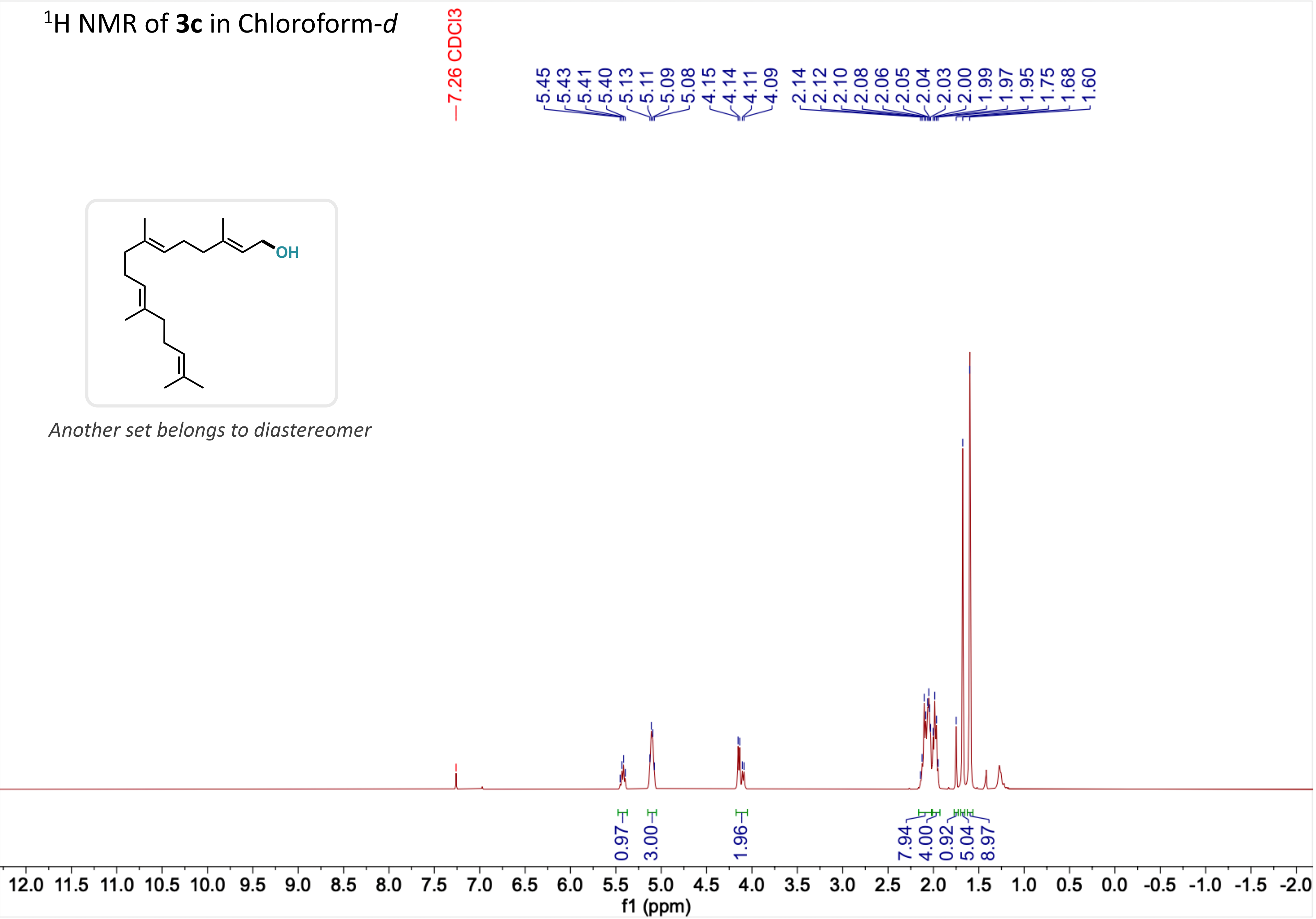

1H NMR of 3c in Chloroform-d
7.26 CDCl3
5.45
5.43
5.41
5.40
5.13
5.11
5.09
5.08
4.15
4.14
4.11
4.09
2.14
2.12
2.10
2.08
2.06
2.05
2.04
2.03
2.00
1.99
1.97
1.95
1.75
1.68
1.60
OH
Another set belongs to diastereomer
0.97
3.00
1.96
7.94
4.00
0.92
5.04
8.97
12.0 11.5 11.0 10.5 10.0 9.5 9.0 8.5 8.0 7.5 7.0 6.5 6.0 5.5 5.0 4.5 4.0 3.5 3.0 2.5 2.0 1.5 1.0 0.5 0.0 -0.5 -1.0 -1.5 -2.0
f1 (ppm)

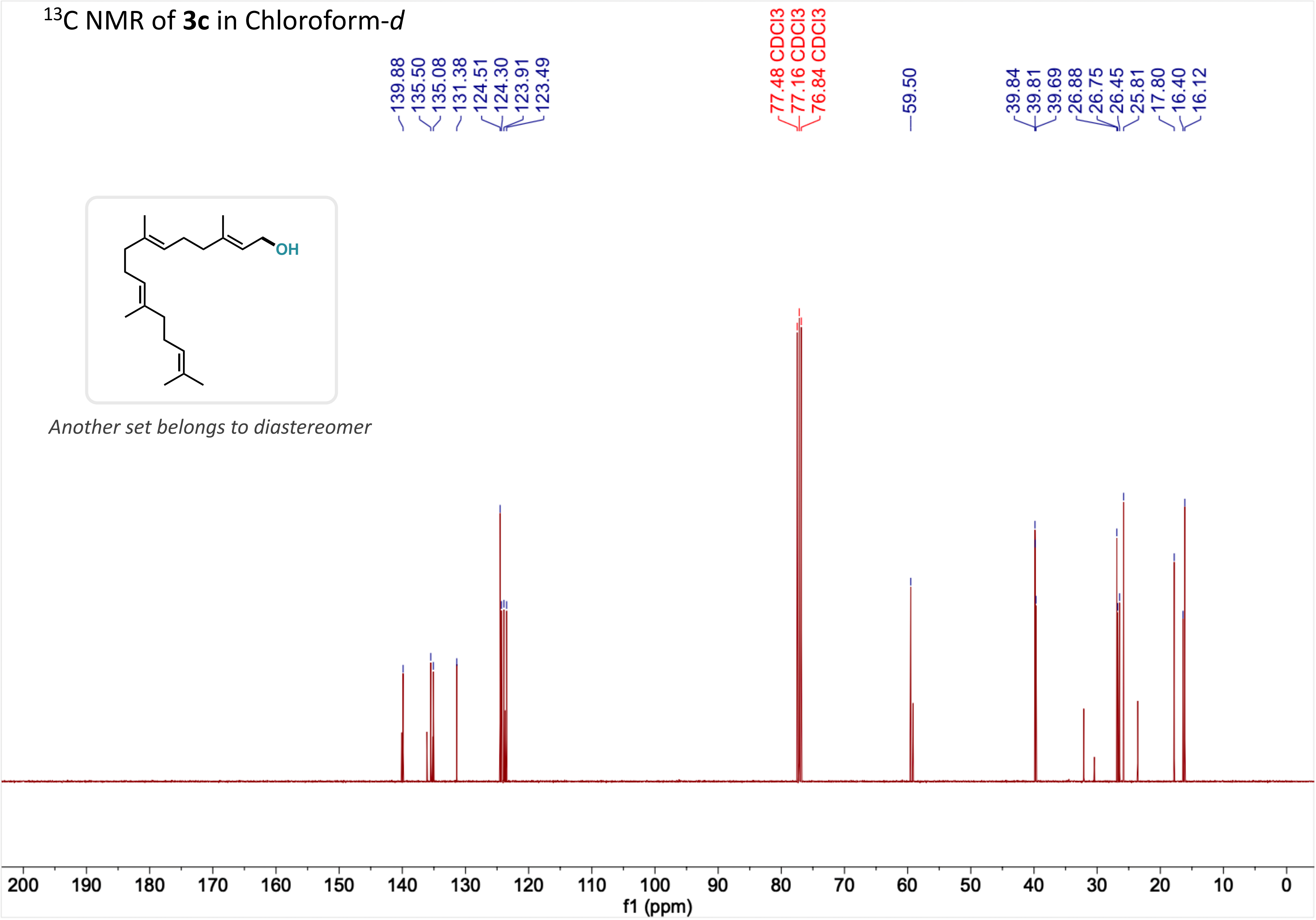

13C NMR of 3c in Chloroform-d
139.88
135.50
135.08
131.38
124.51
124.30
123.91
123.49
77.48 CDCl3
77.16 CDCl3
76.84 CDCl3
59.50
39.84
39.81
39.69
26.88
26.75
26.45
25.81
17.80
16.40
16.12
OH
Another set belongs to diastereomer
200 190 180 170 160 150 140 130 120 110 100 90 80 70 60 50 40 30 20 10 0
f1 (ppm)

^{1}H NMR of **4a** in Chloroform-*d*

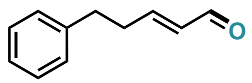

Another set belongs to diastereomer

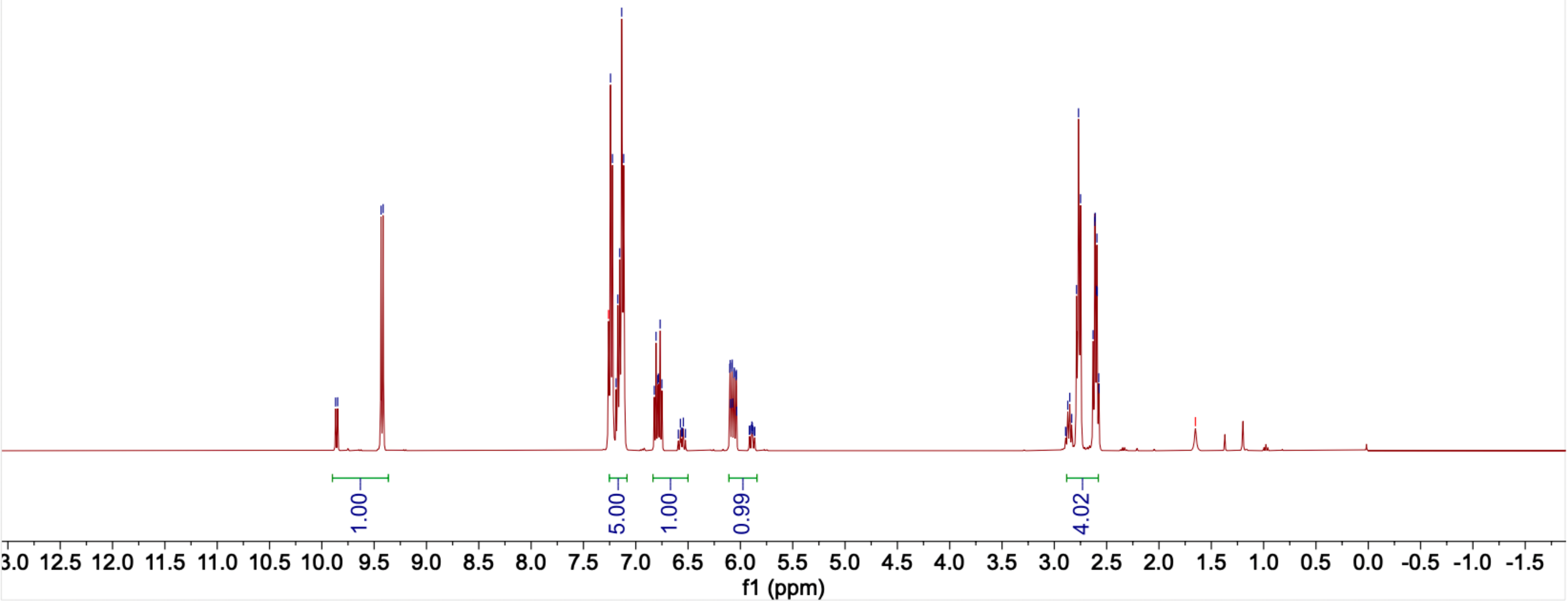

^{13}C NMR of **4a** in Chloroform-*d*

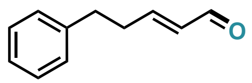

Another set belongs to diastereomer

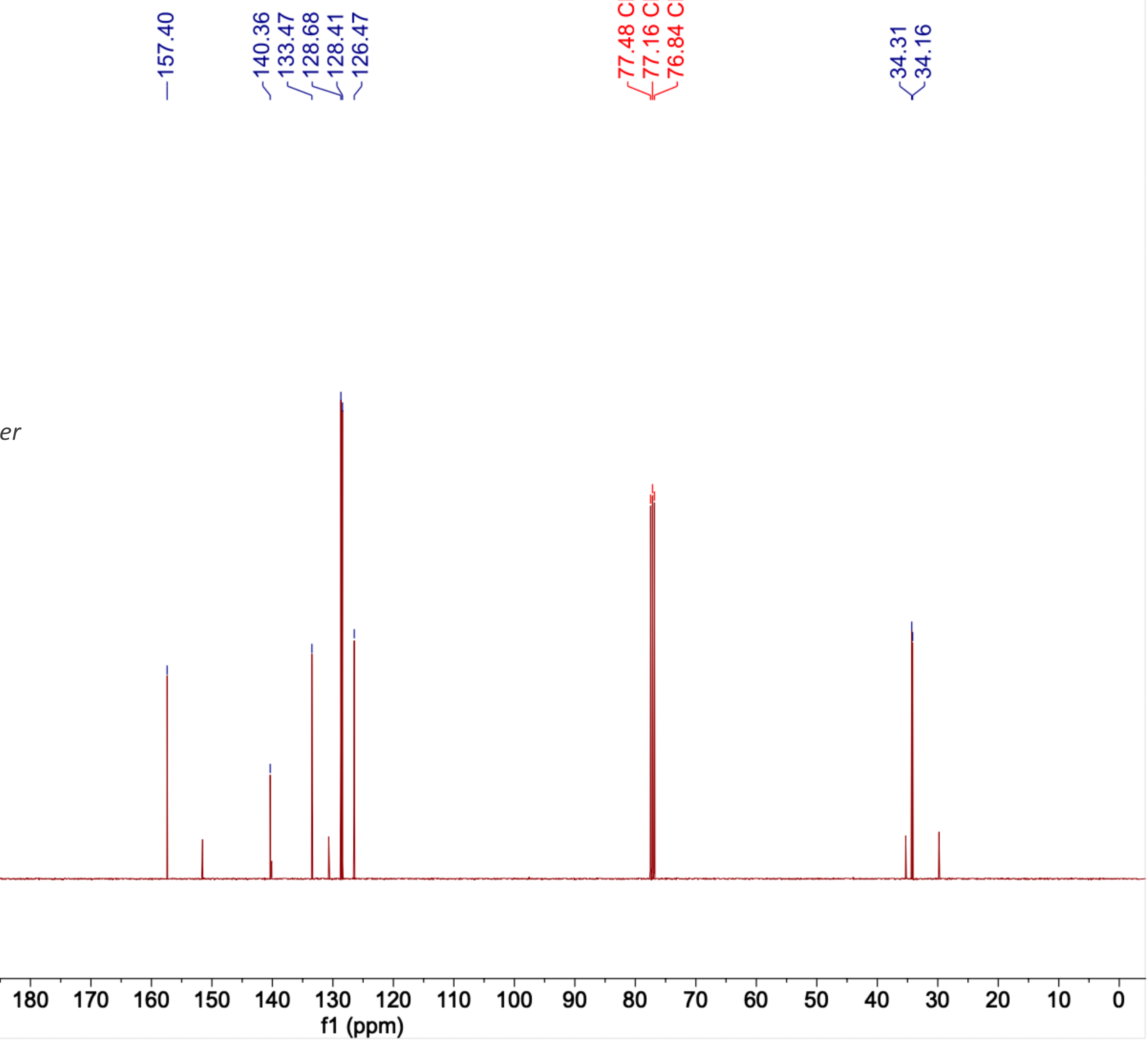

^{1}H NMR of **4b** in Chloroform-*d*

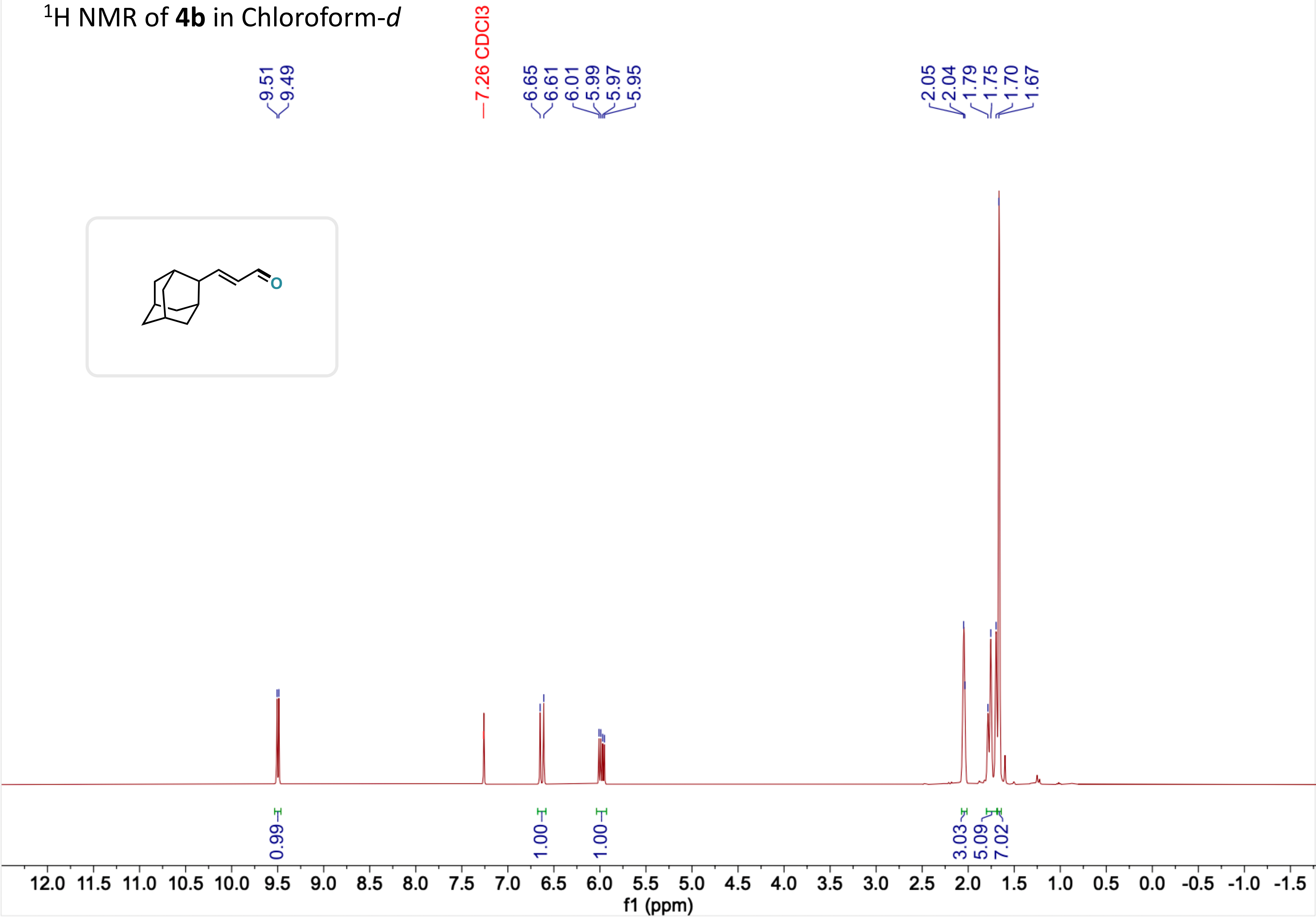

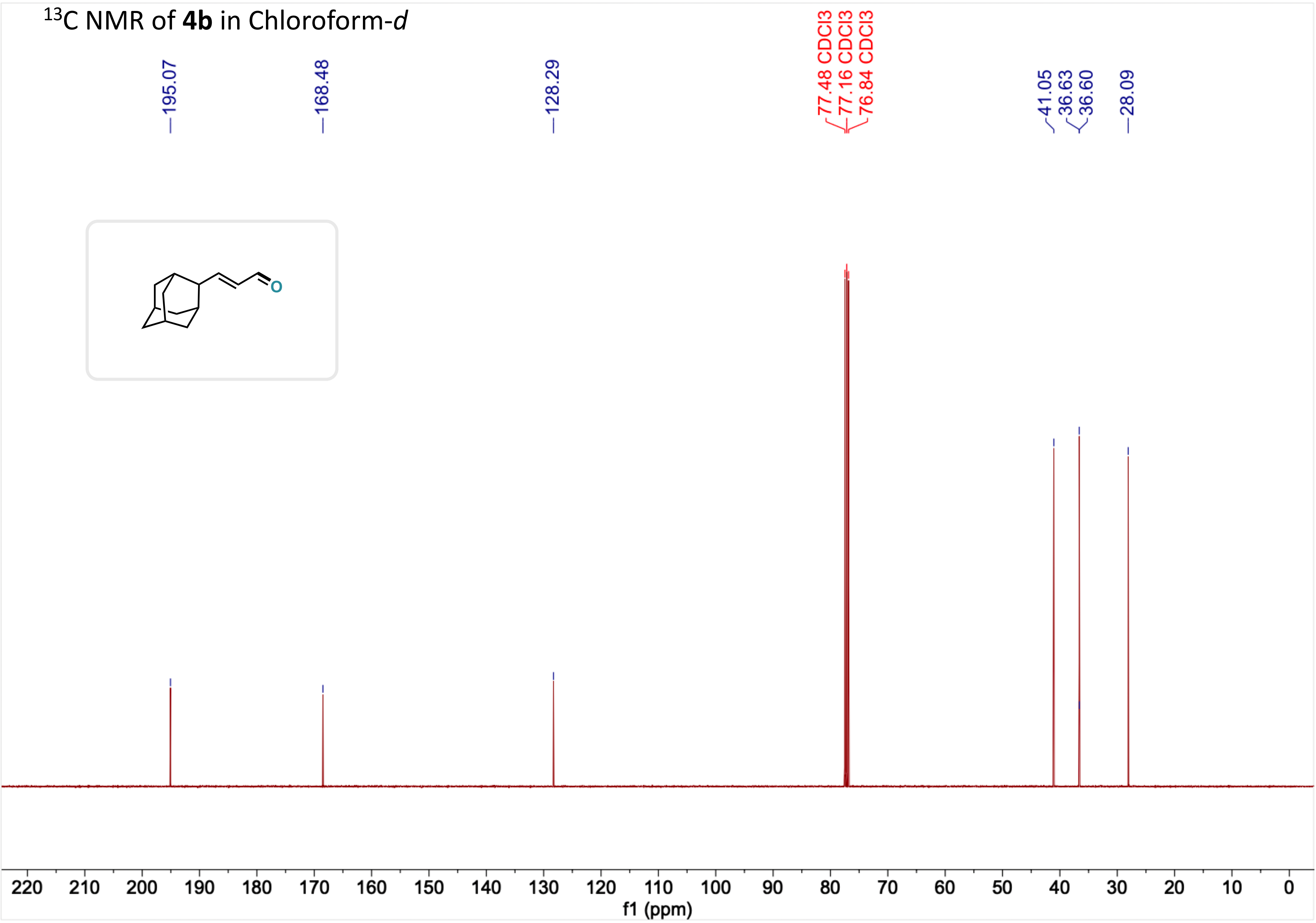

^{13}C NMR of **4b** in Chloroform-*d*
195.07
168.48
128.29
77.48 CDCl3
77.16 CDCl3
76.84 CDCl3
41.05
36.63
36.60
28.09
O
220 210 200 190 180 170 160 150 140 130 120 110 100 90 80 70 60 50 40 30 20 10 0
f1 (ppm)

^{1}H NMR of **4c** in Chloroform-*d*

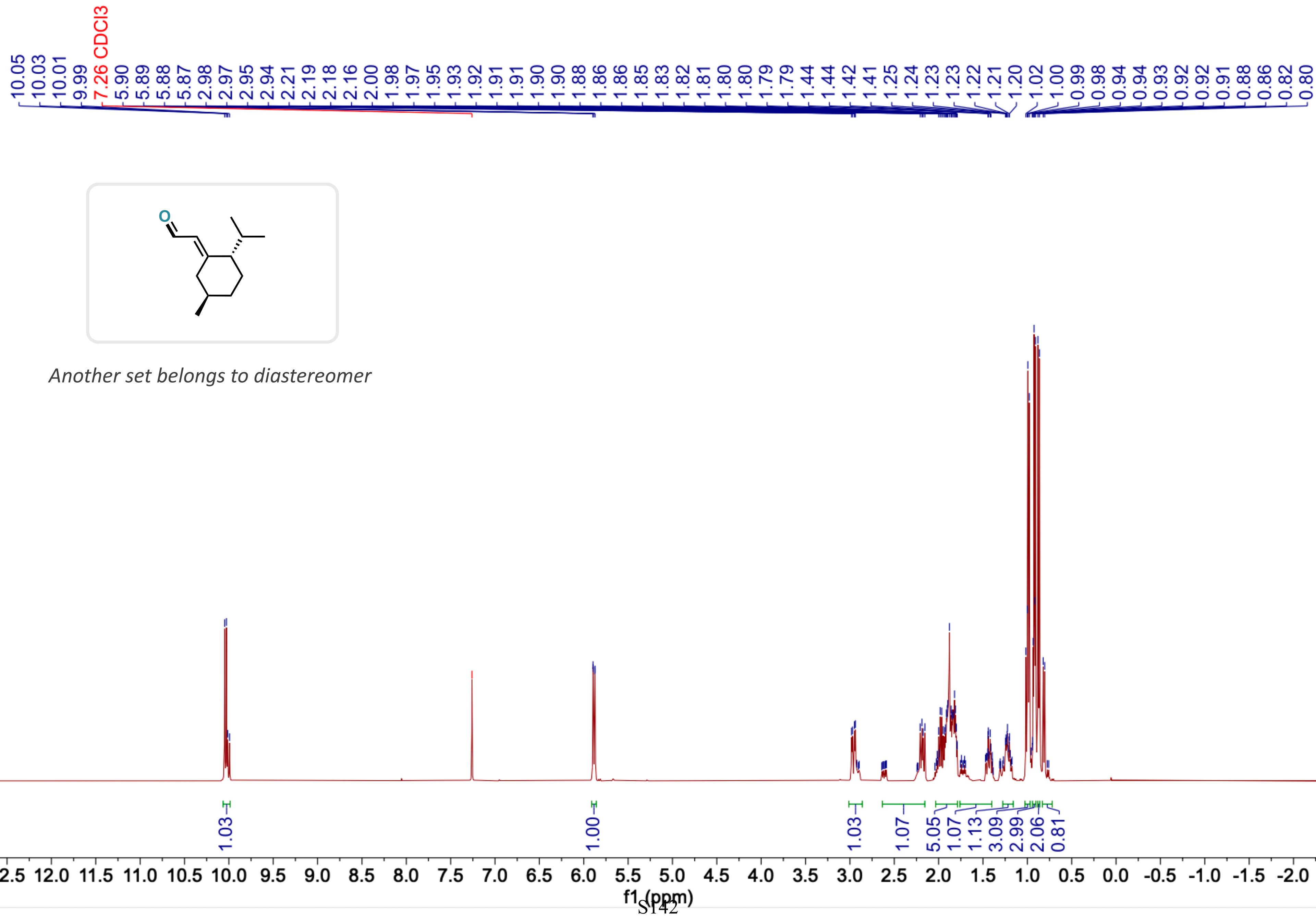

Another set belongs to diastereomer

^{13}C NMR of **4c** in Chloroform-*d*

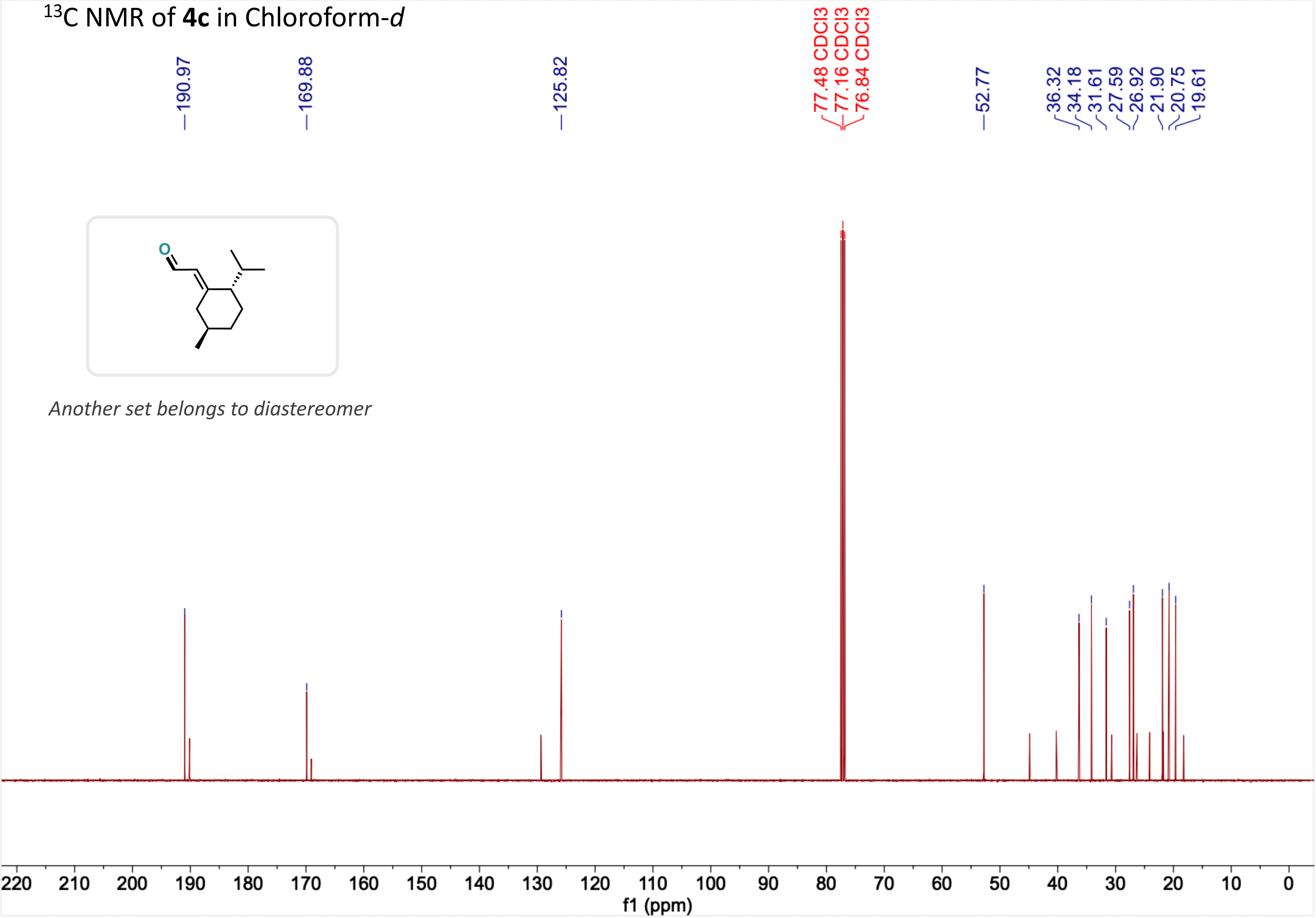

^{1}H NMR of **4d** in Chloroform-*d*

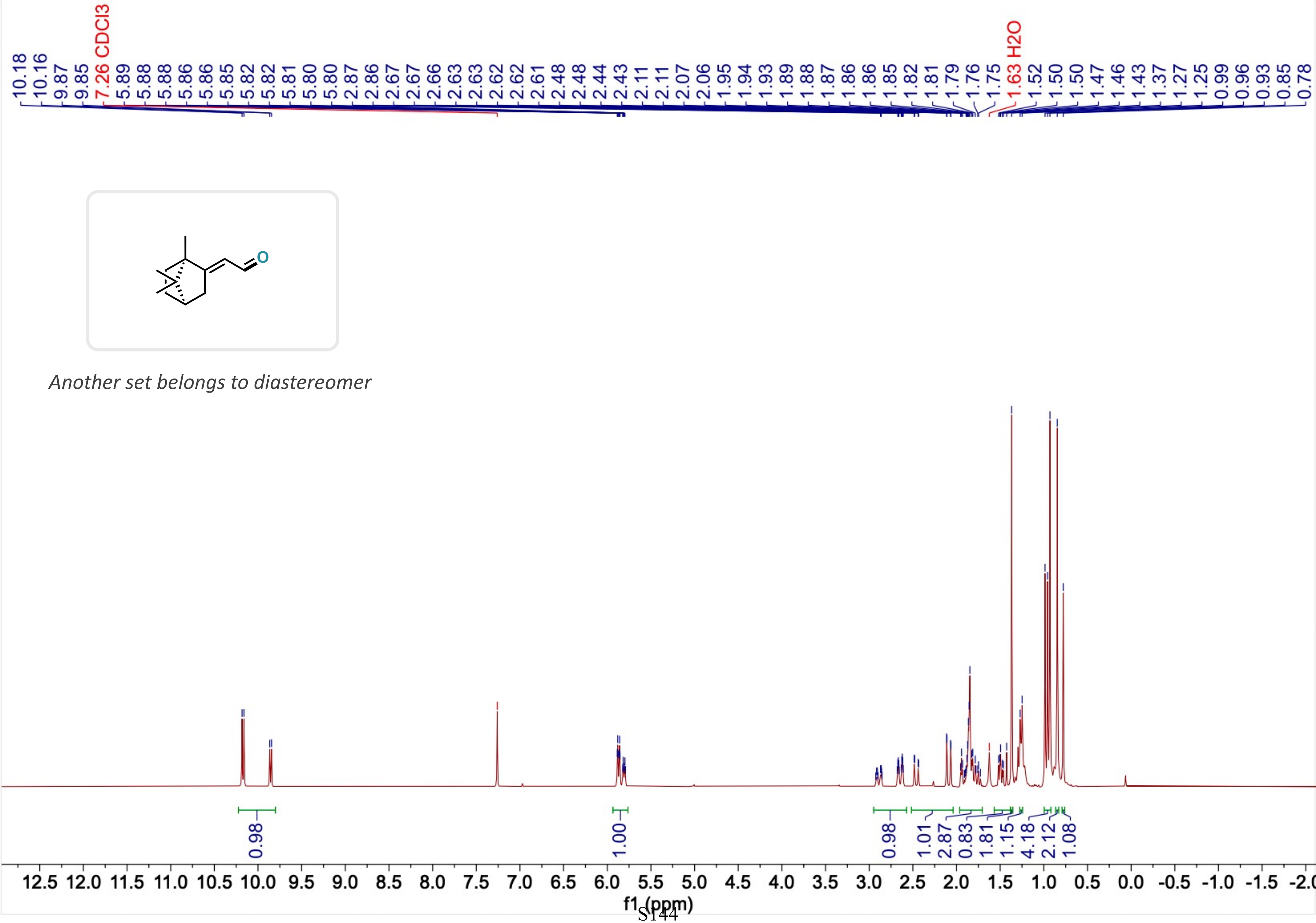

Another set belongs to diastereomer

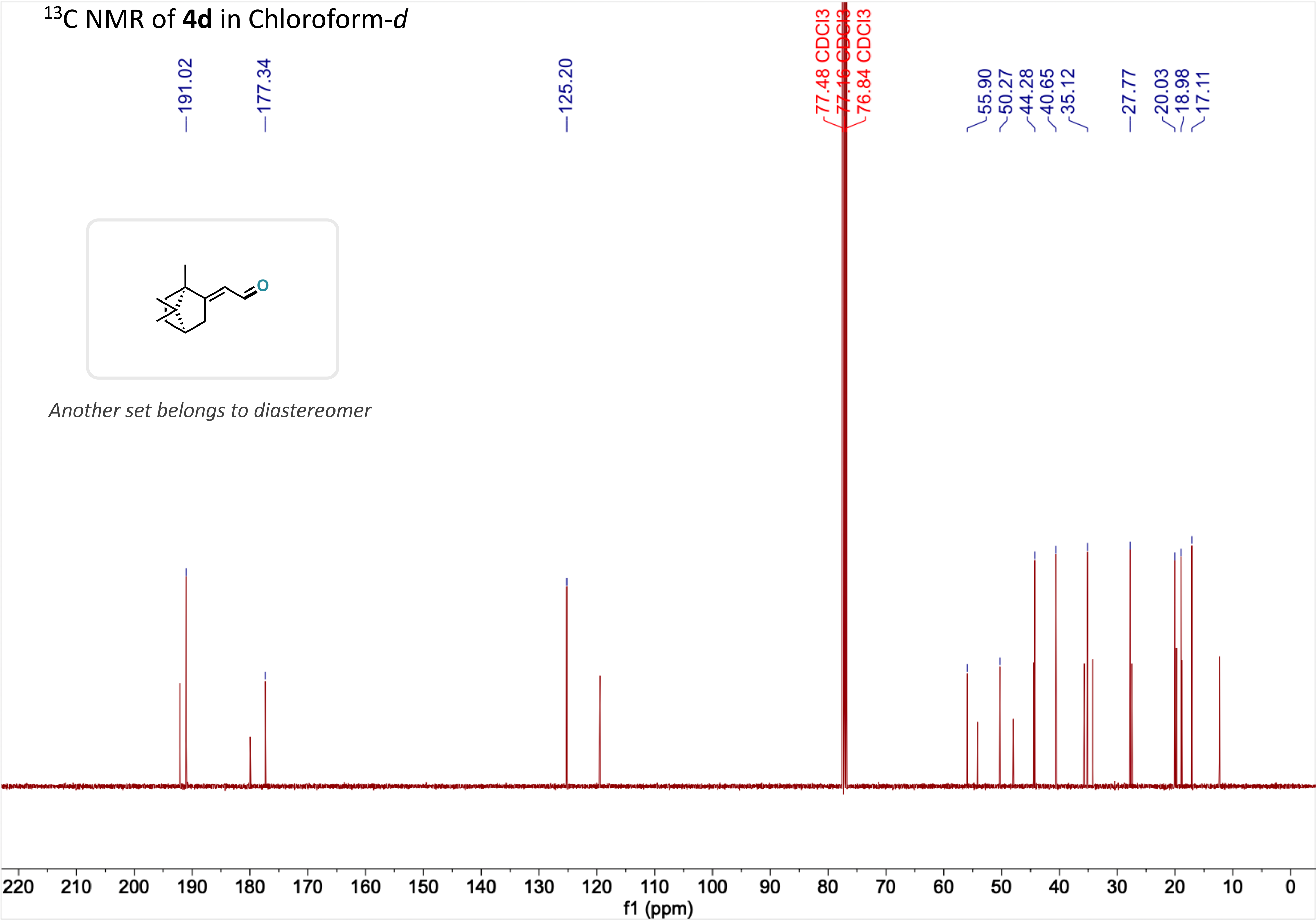
13C NMR of 4d in Chloroform-d
191.02
177.34
125.20
77.48 CDCl3
77.16 CDCl3
76.84 CDCl3
55.90
50.27
44.28
40.65
35.12
27.77
20.03
18.98
17.11
O
Another set belongs to diastereomer
220 210 200 190 180 170 160 150 140 130 120 110 100 90 80 70 60 50 40 30 20 10 0
f1 (ppm)

^{1}H NMR of **4e** in Chloroform-*d*

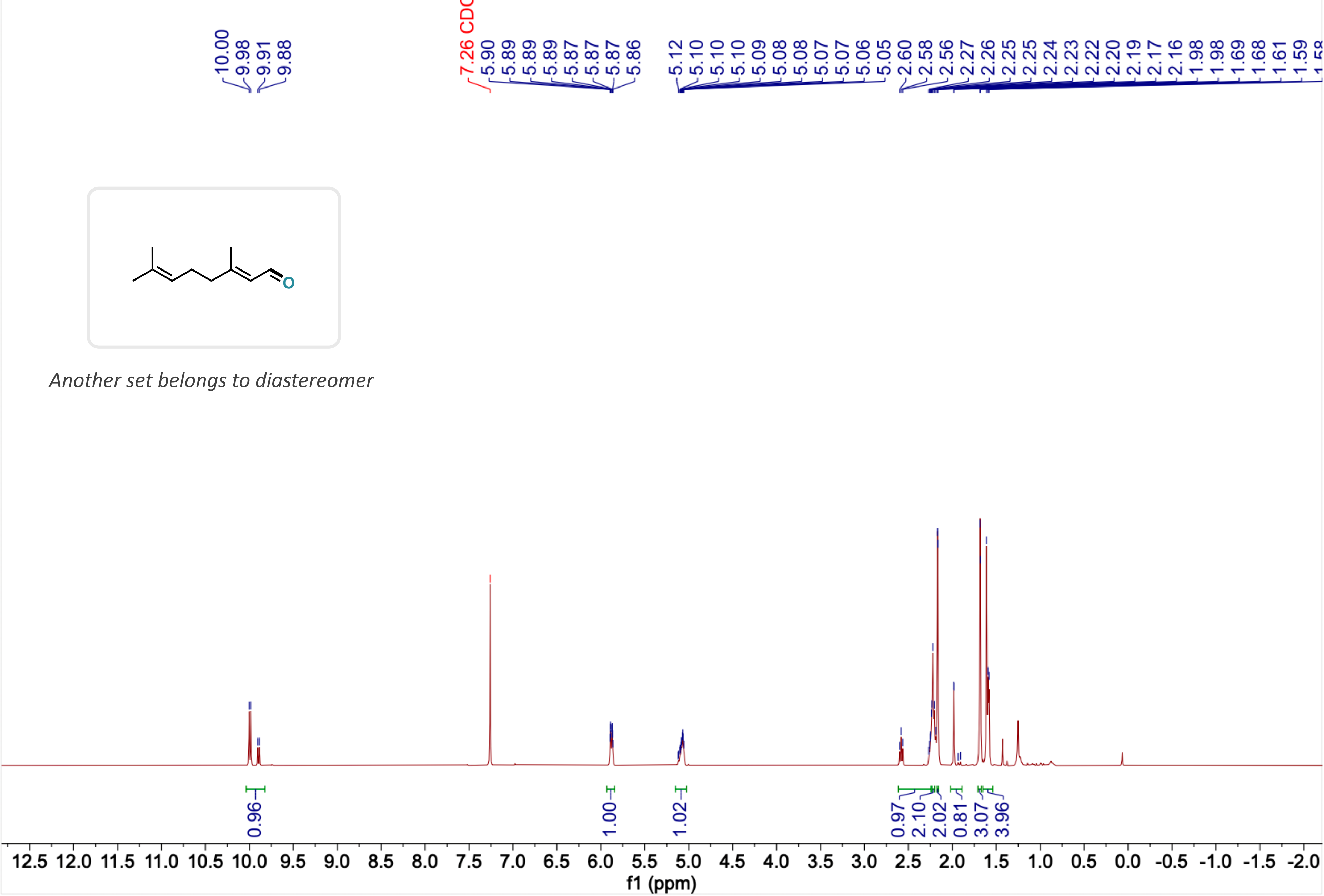

^{13}C NMR of **4e** in Chloroform-*d*

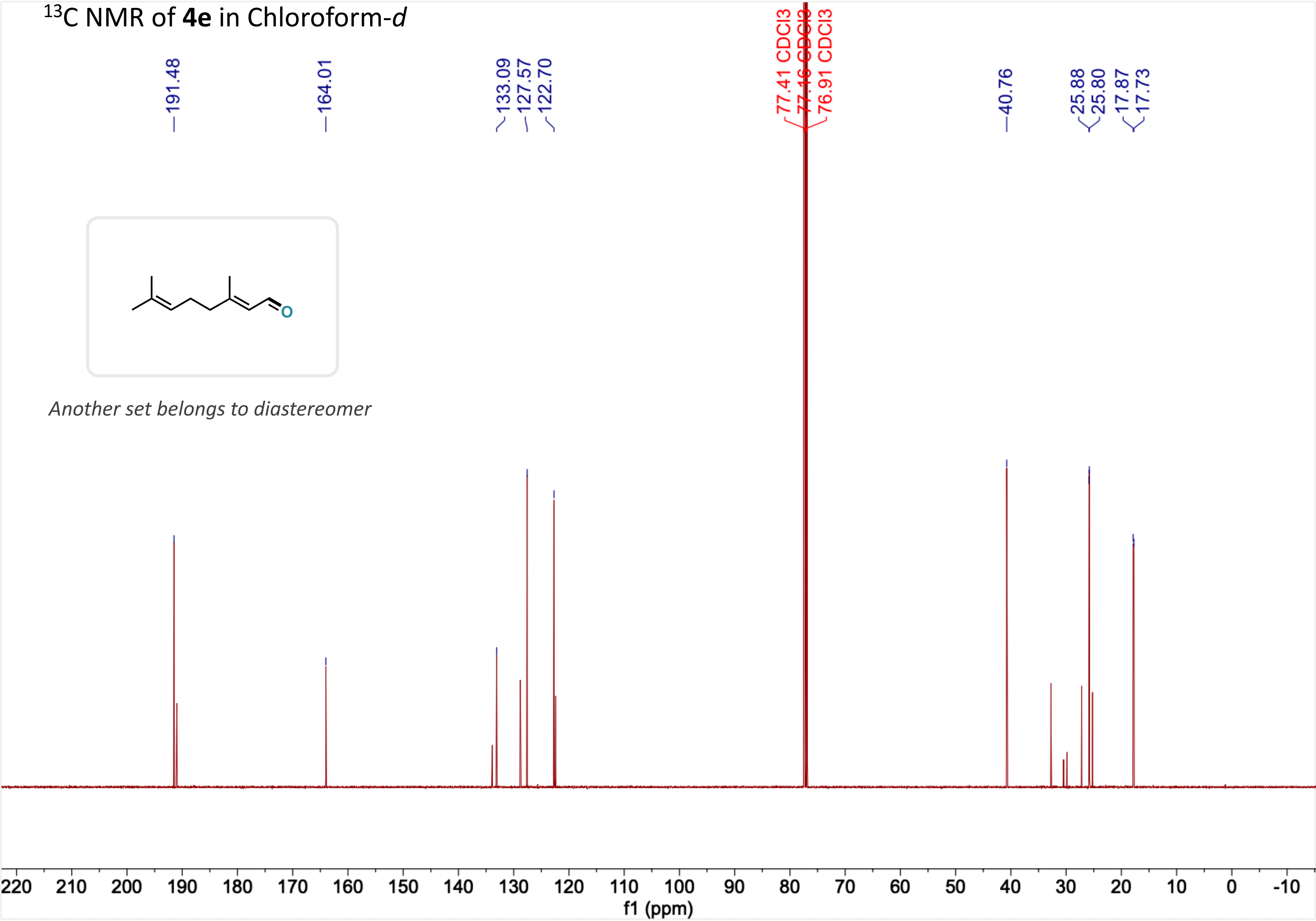

^{1}H NMR of **4f** in Chloroform-*d*

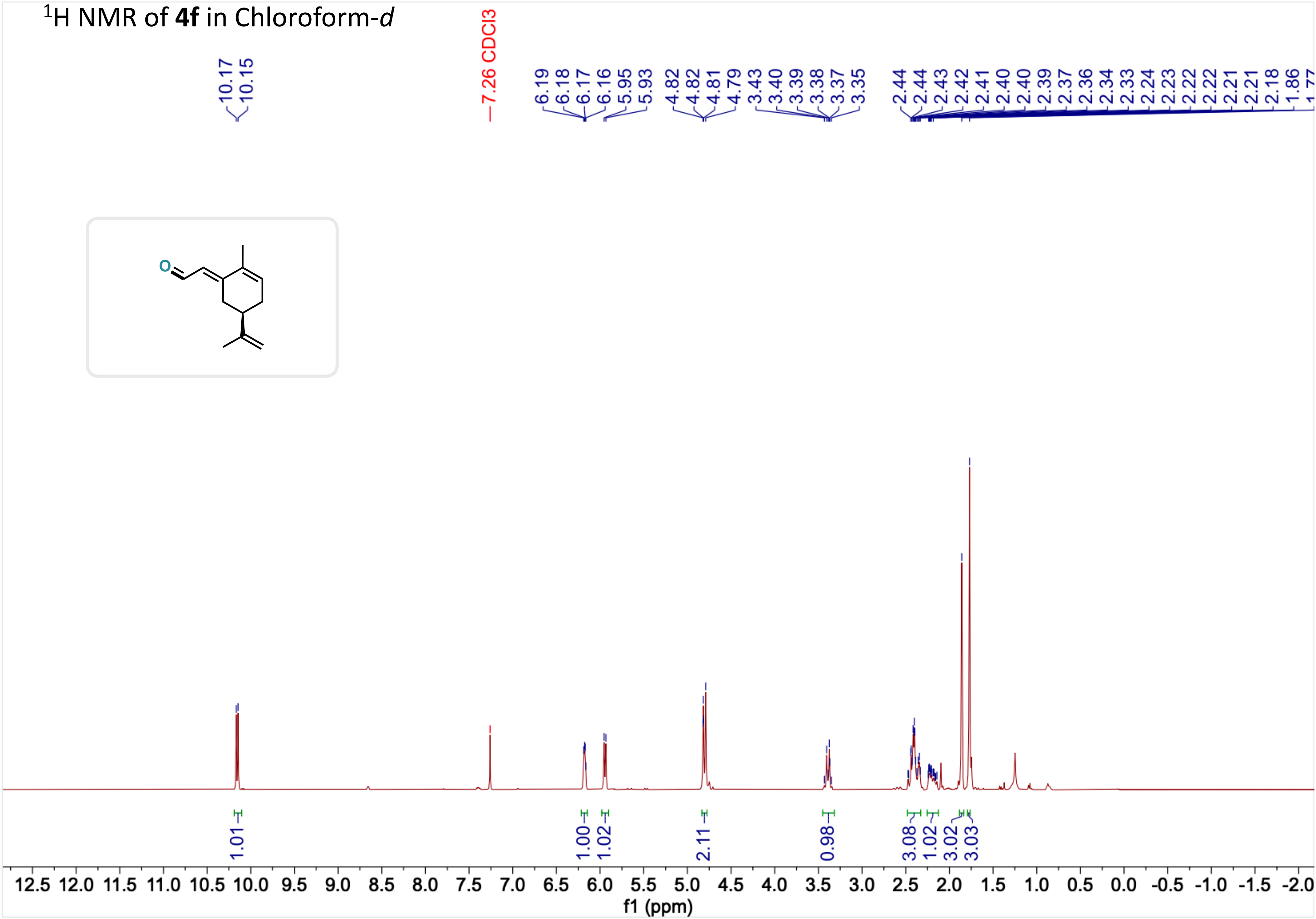

^{13}C NMR of **4f** in Chloroform-*d*

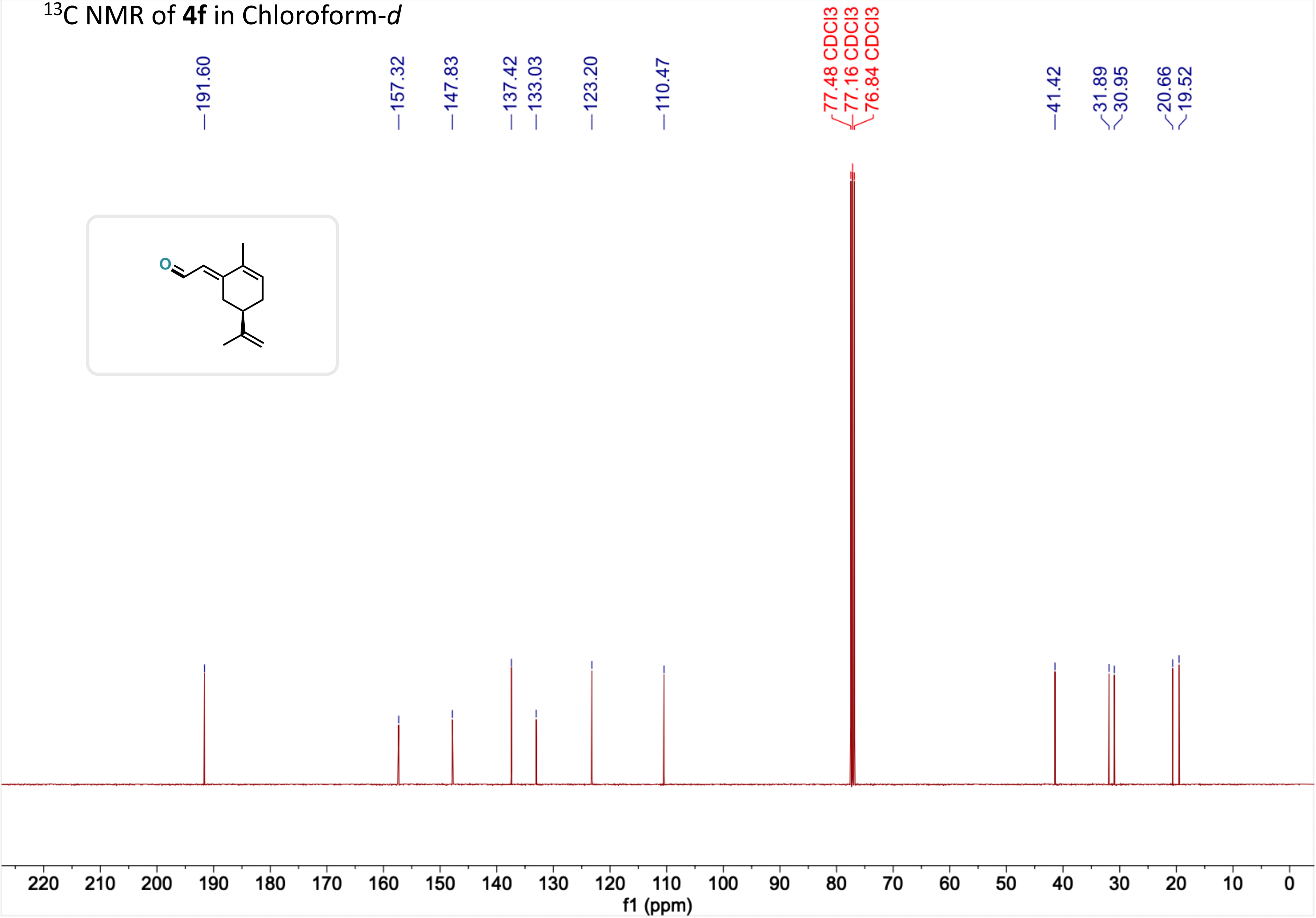

^{1}H NMR of **5a** in Chloroform-*d*

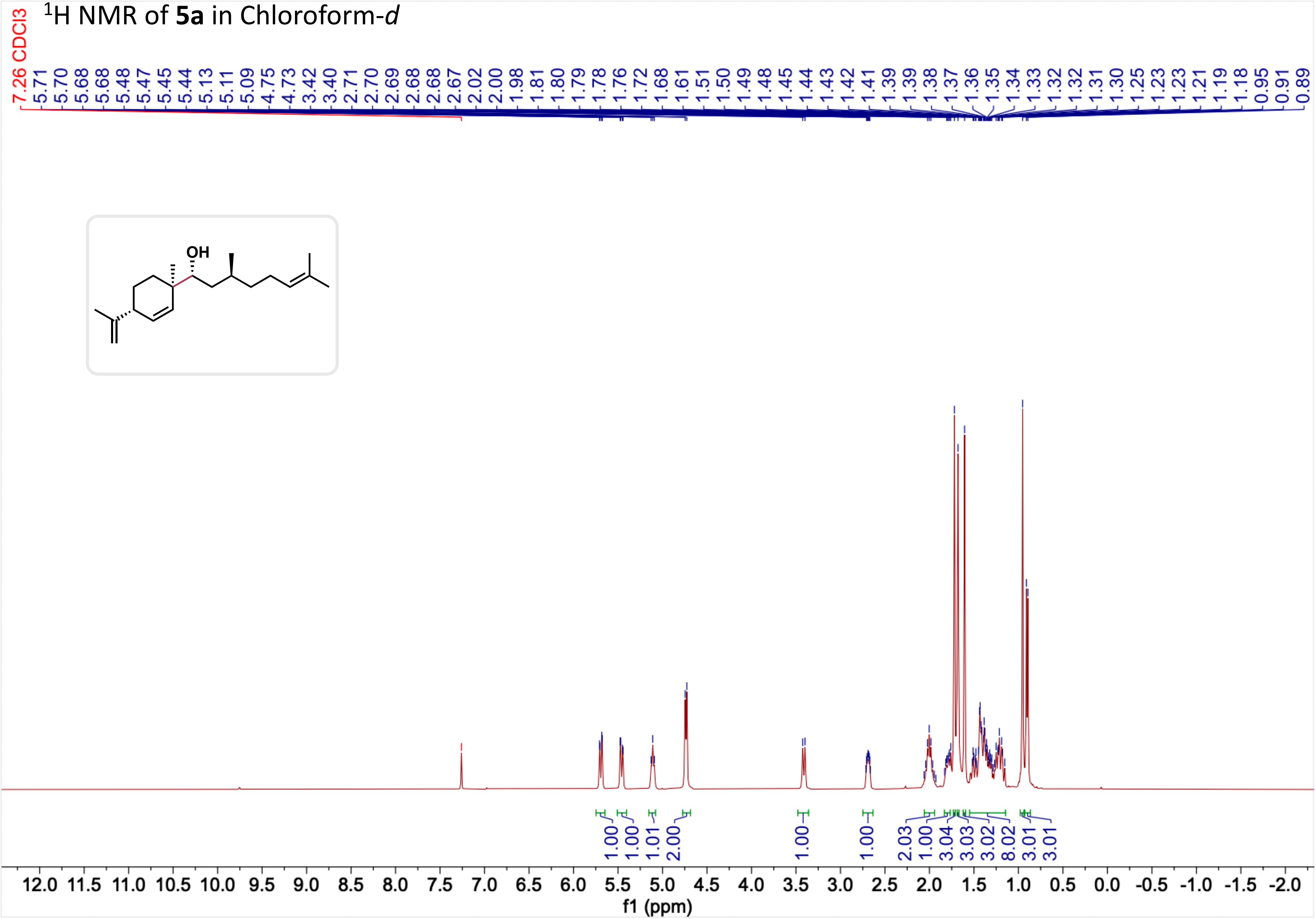

^{13}C NMR of **5a** in Chloroform-*d*

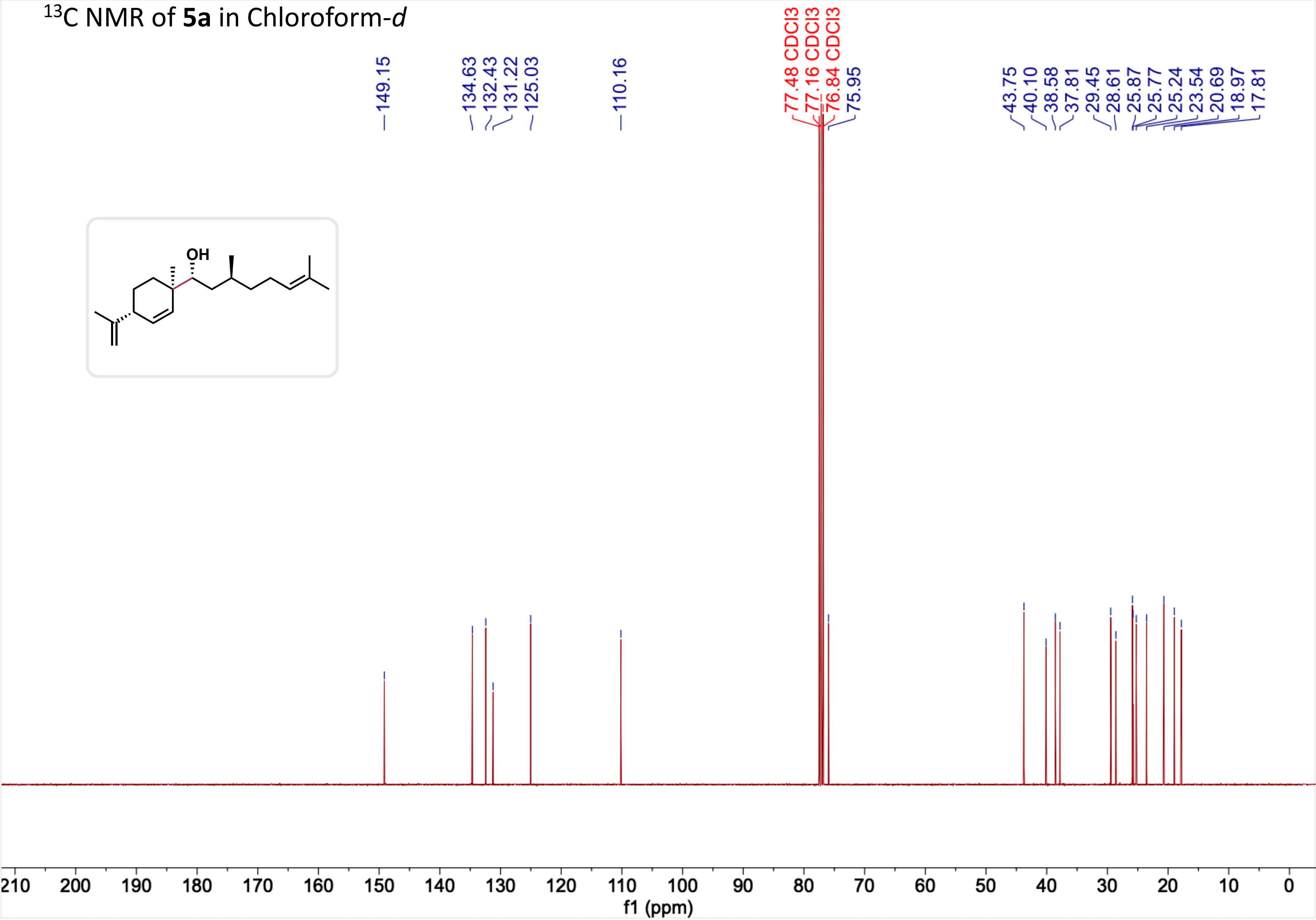

^{1}H-^{1}H gCOSY NMR of **5a** in Chloroform-*d*

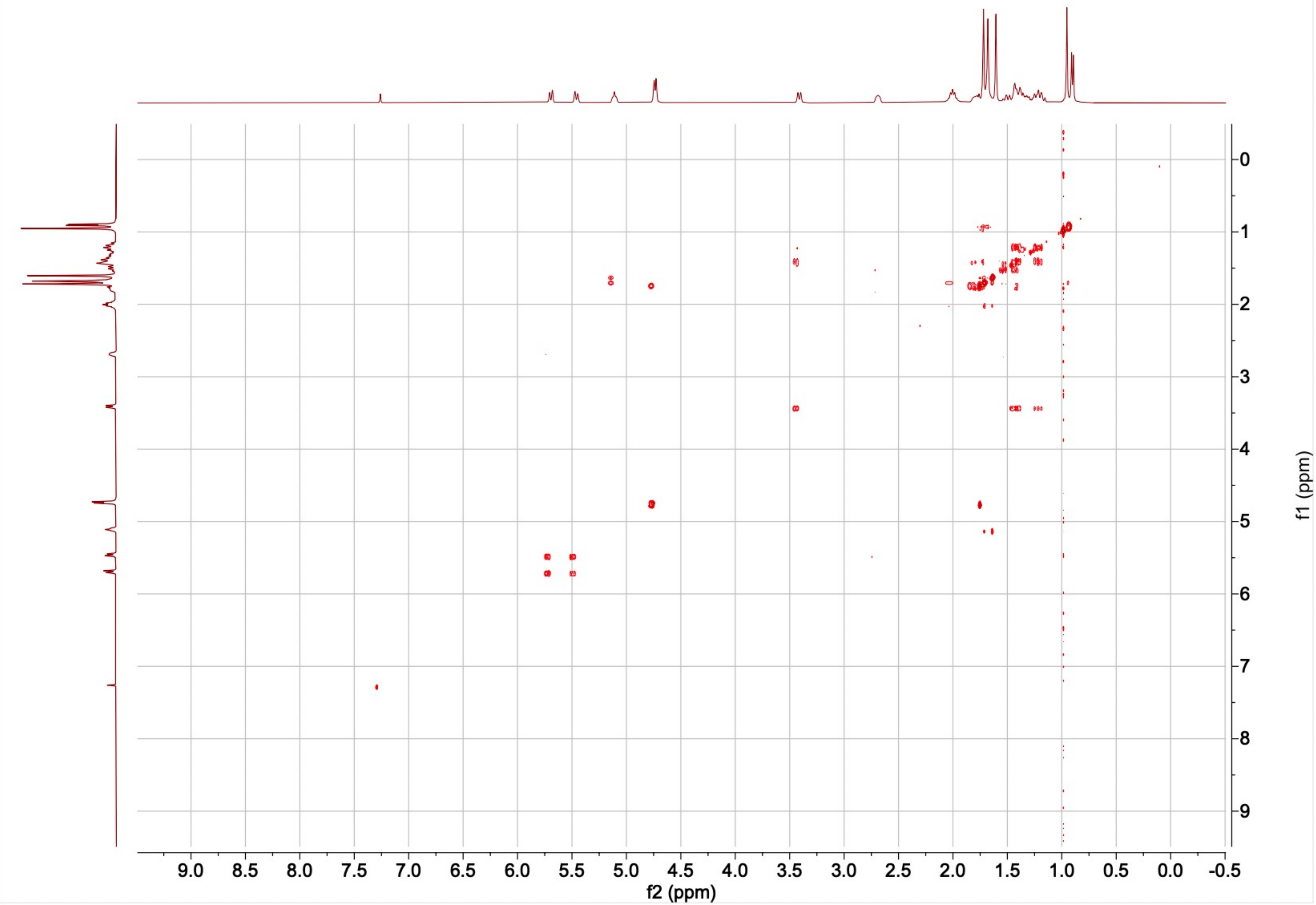

^{1}H-^{13}C HMBC NMR of **5a** in Chloroform-*d*

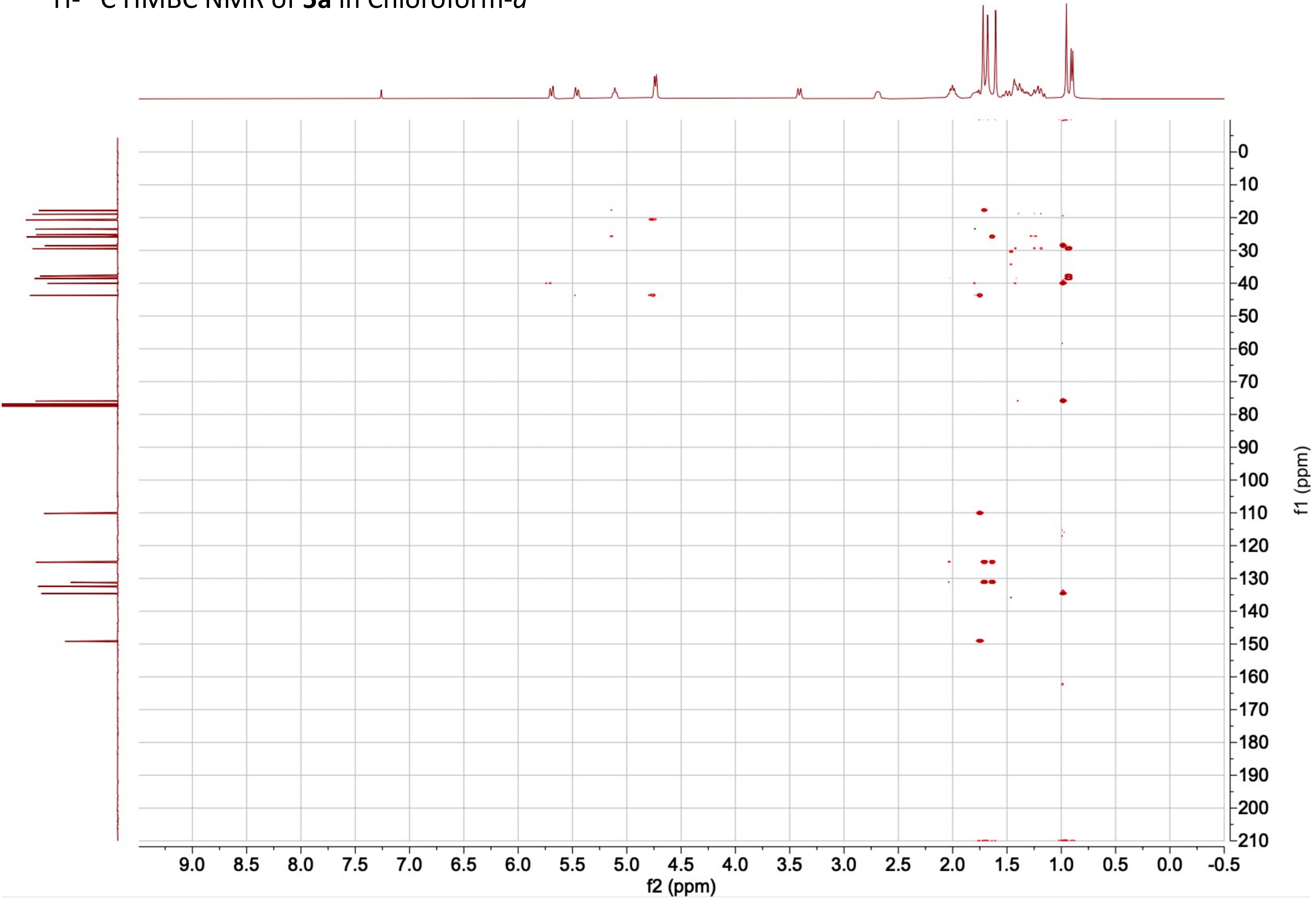

^{1}H-^{13}C HSQC NMR of **5a** in Chloroform-*d*

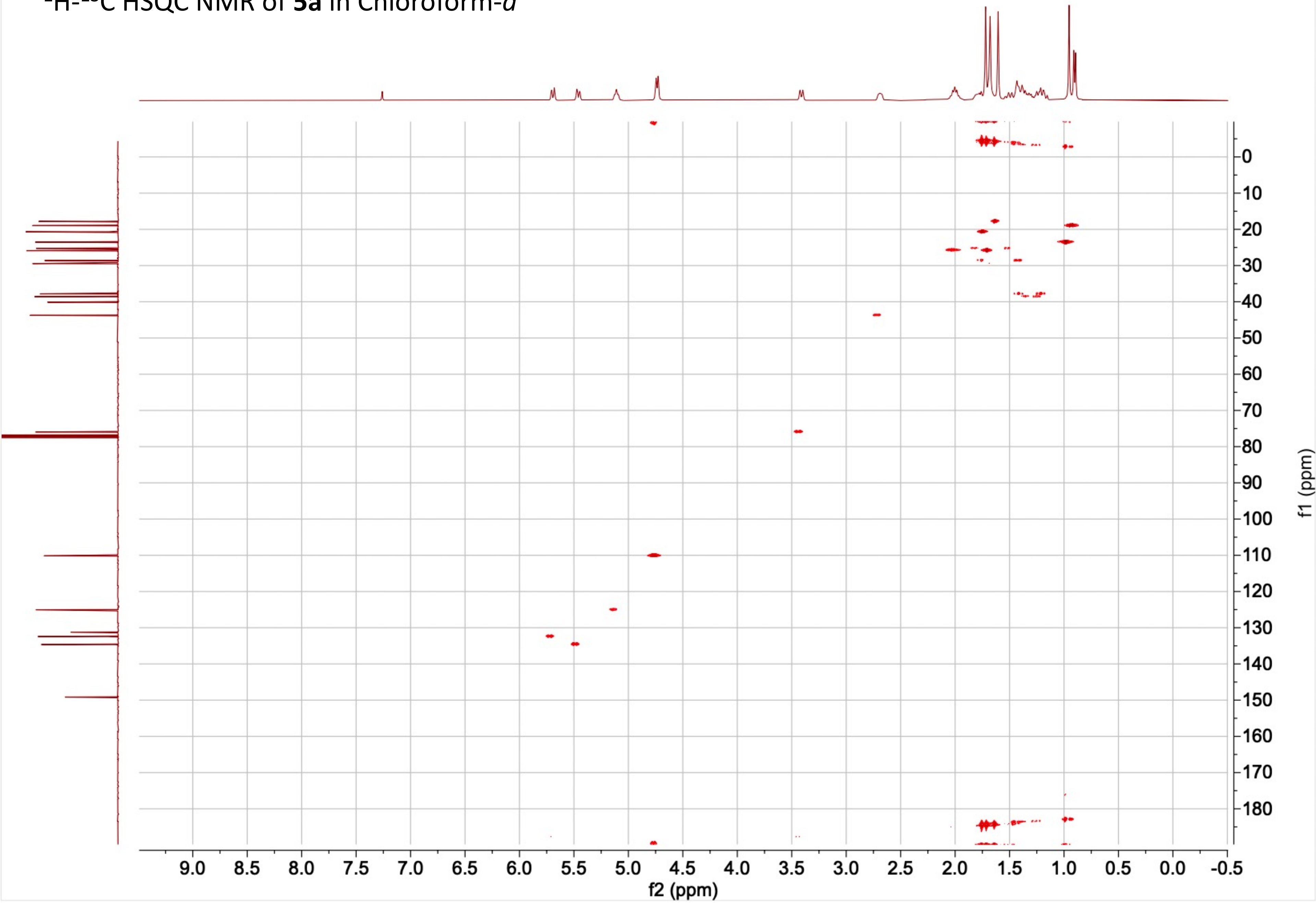

^{1}H-^{1}H NOESY NMR of **5a** in Chloroform-*d*

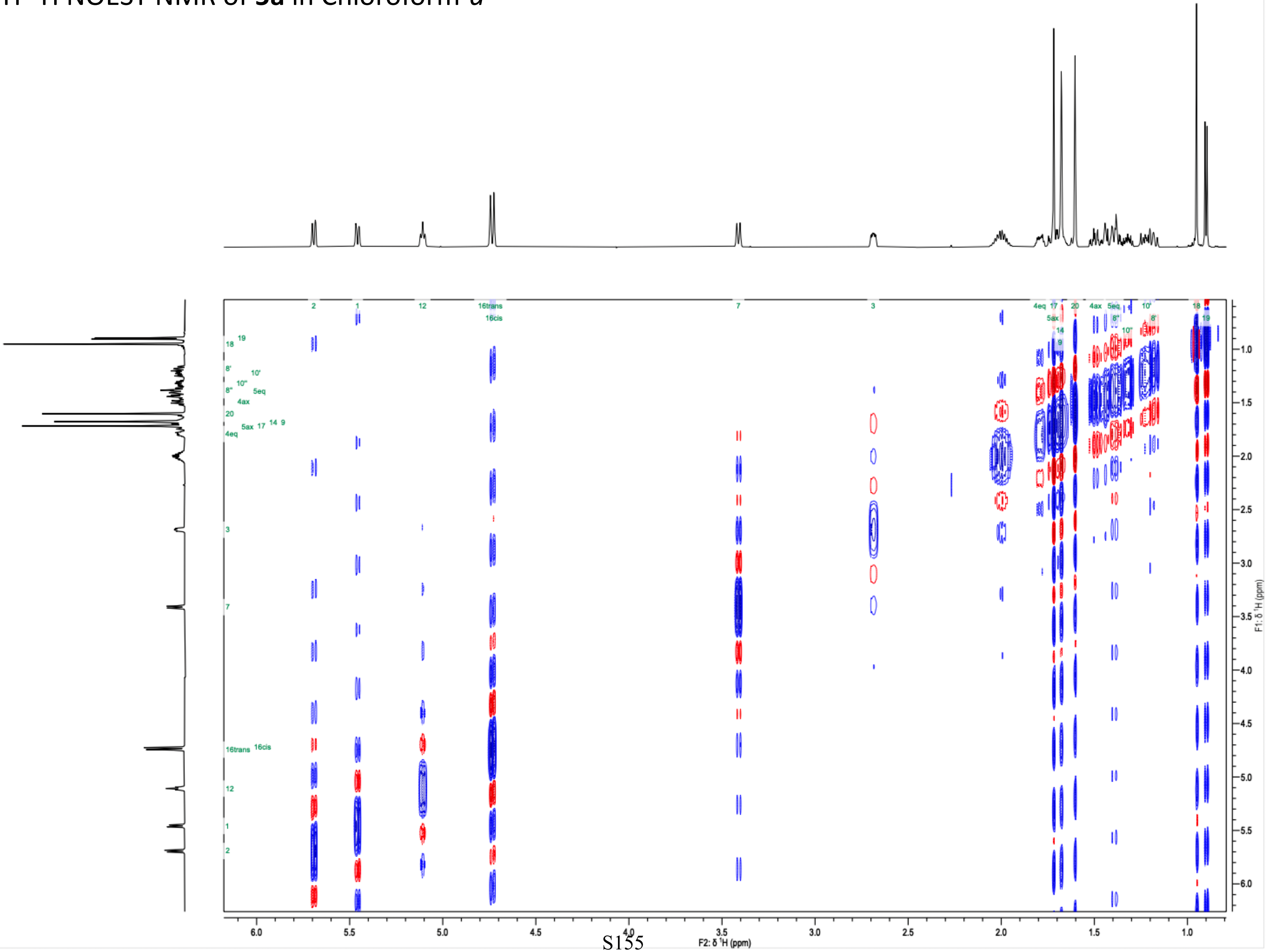

Relative Configuration of **5a** Determined by 2D NMR

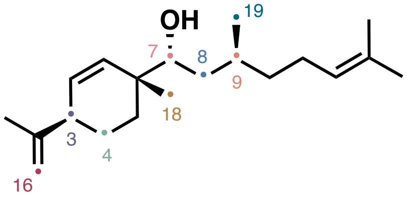

All chemical shifts could be assigned based on 2D COSY, HSQC and HMBC. The relative stereo configuration was determined based on multiplet analysis and 2D NOESY.

The vinyl H's were assigned based on chem shift and splitting. H-3 [2.69 (ddt, *J* = 10.4, 5.4, 2.7 Hz, 1H)] was identified by COSY from H-16's. H-3 has one large coupling constant indicating that it is in the axial position with one axial neighbor, so the isopropenyl group is equatorial. COSY correlations from H-3 helped identify H-4ax 1.49 (app. tdd, *J* = 12.8, 9.9, 3.1 Hz, 1H) and H-4eq 1.79 (dtdd, *J* = 12.8, 5.2, 3.2, 1.0 Hz, 1H). H-4ax gives and nOe correlation to CH3-18 indicating that the methyl is axial, so it is syn to the isopropenyl group and the stereocenter has R configuration. H-8' 1.38 (ddd, *J* = 13.8, 10.4, 3.0 Hz, 1H) and H-8'' 1.18 (ddd, *J* = 13.9, 10.5, 1.7 Hz, 1H) are both ddd's with two large J's. This shows that 1) rotation around the C7-C8 and C8-C9 bonds are restricted, and 2) each H8 is antiperiplanar to one neighboring H, which means that the OH and Me-19 are anti making C7 R.

^{1}H NMR of **5b** in Chloroform-*d*

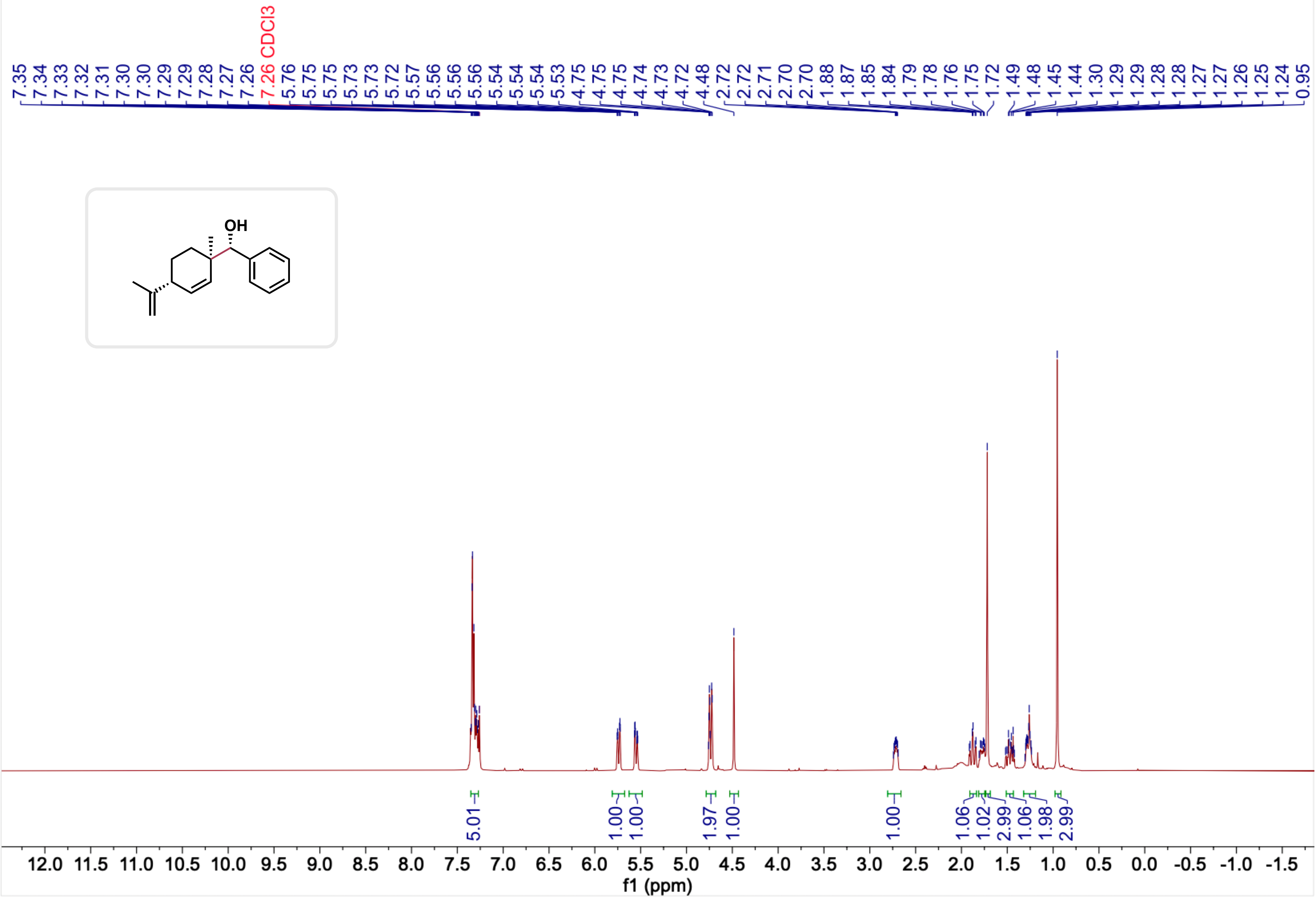

^{13}C NMR of **5b** in Chloroform-*d*

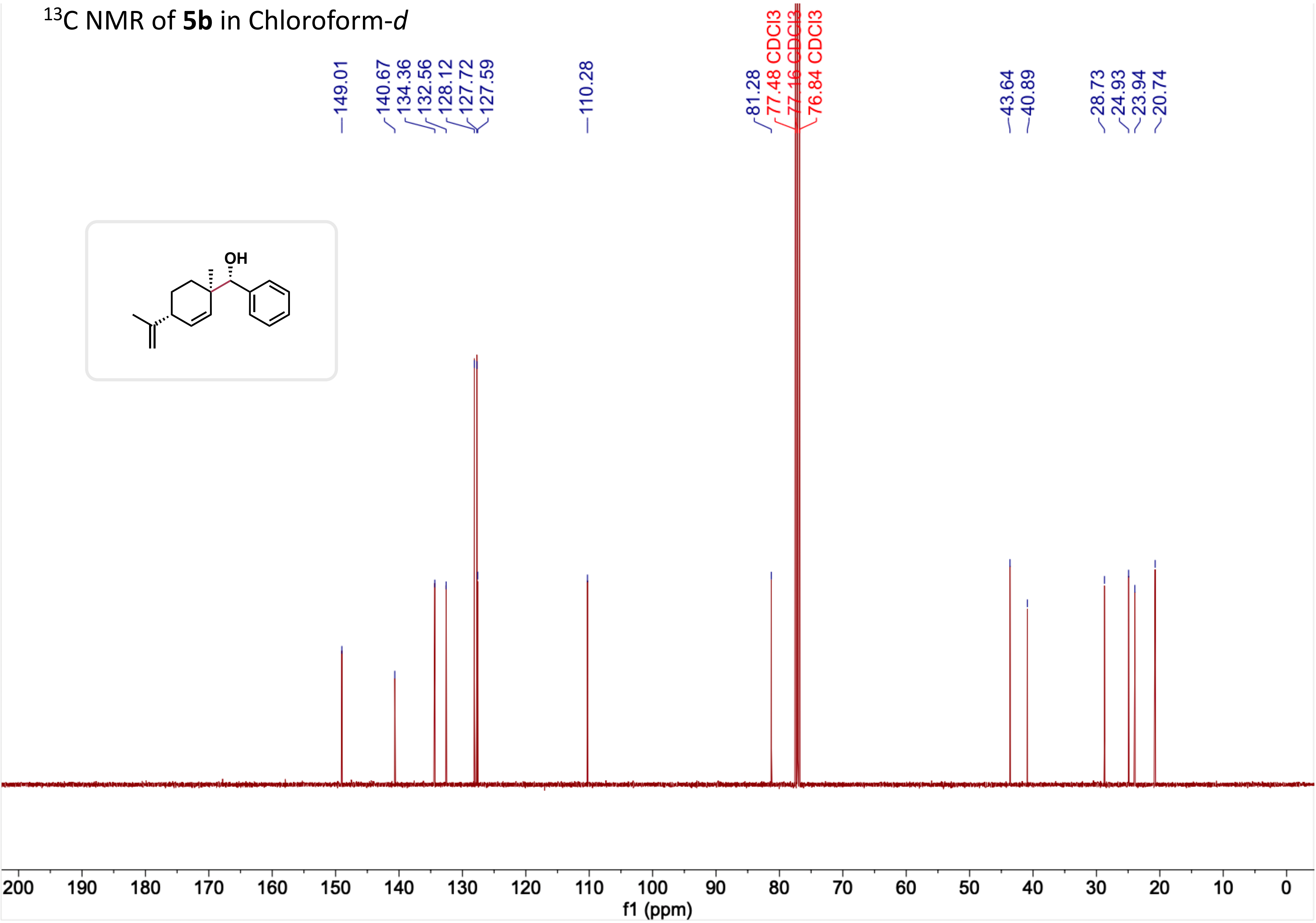

^1H NMR of **5c** in Chloroform-*d*

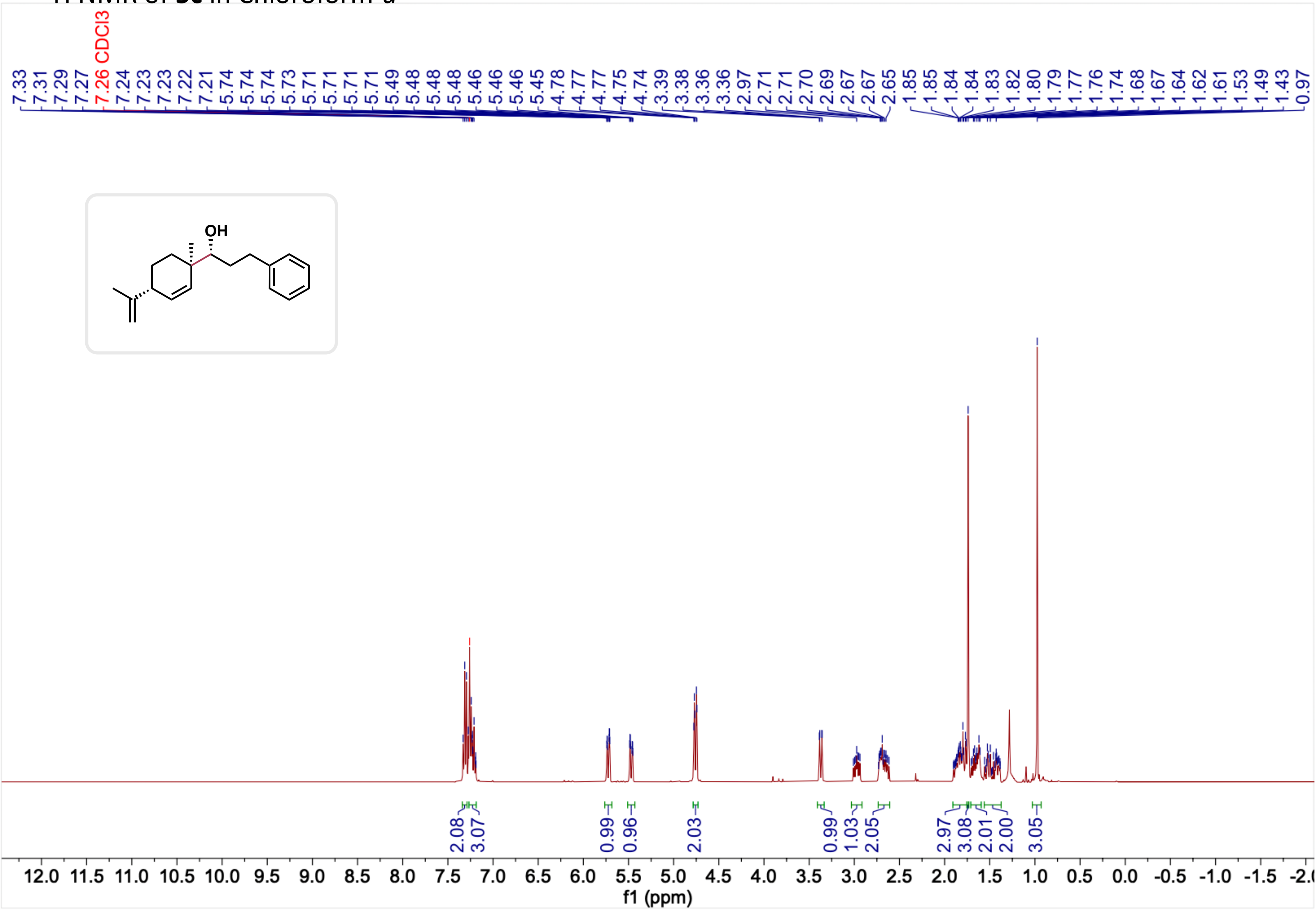

^{13}C NMR of **5c** in Chloroform-*d*

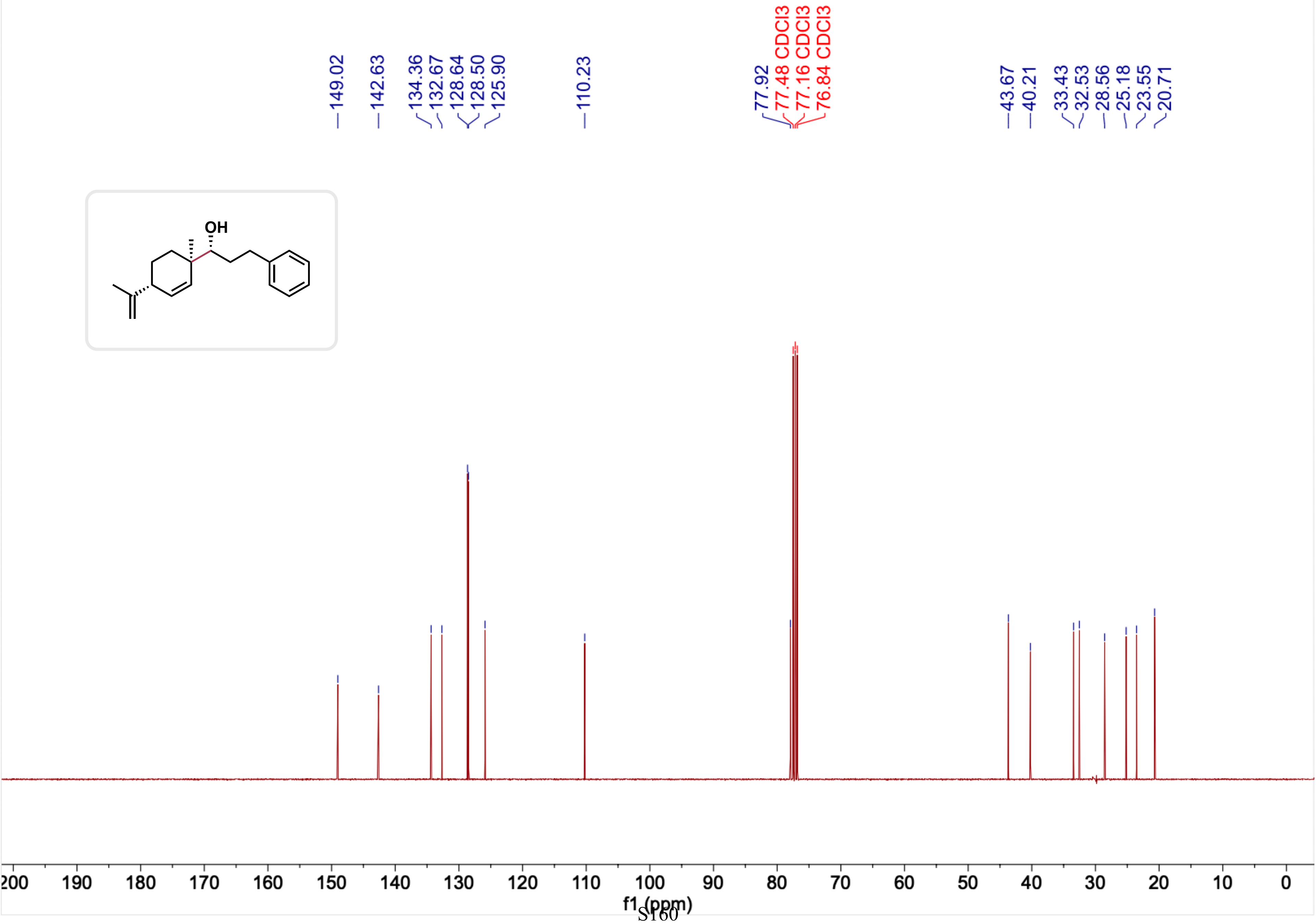

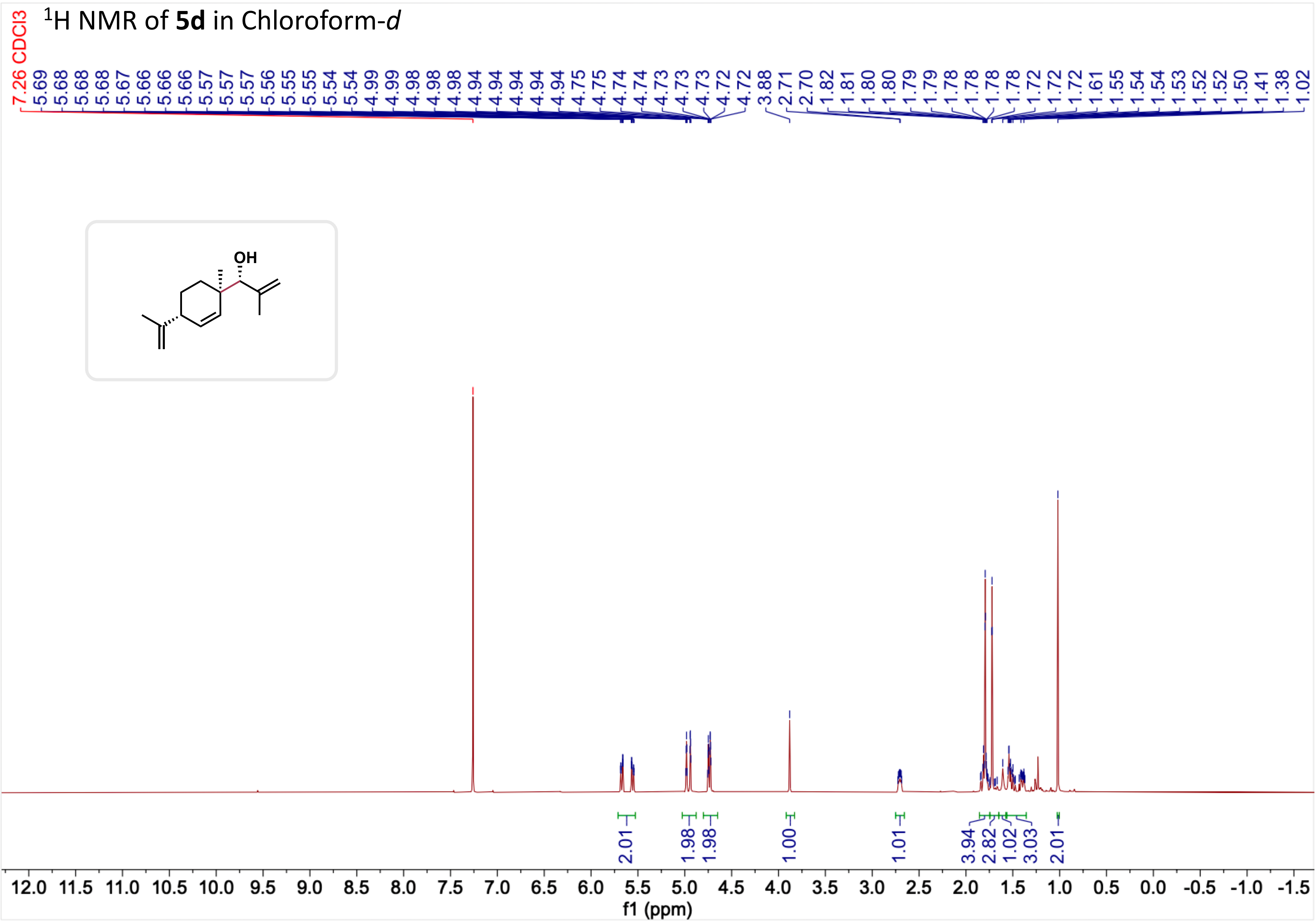

^{1}H NMR of **5d** in Chloroform-*d*
OH
7.26 CDCl3
5.69
5.68
5.68
5.68
5.67
5.66
5.66
5.66
5.57
5.57
5.57
5.56
5.55
5.55
5.54
5.54
4.99
4.99
4.98
4.98
4.98
4.94
4.94
4.94
4.94
4.94
4.75
4.75
4.74
4.74
4.73
4.73
4.73
4.72
4.72
3.88
2.71
2.70
1.82
1.81
1.80
1.80
1.79
1.78
1.78
1.78
1.78
1.78
1.72
1.72
1.72
1.61
1.55
1.54
1.54
1.53
1.52
1.52
1.50
1.41
1.38
1.02
2.01
1.98
1.98
1.00
1.01
3.94
2.82
1.02
3.03
2.01
12.0 11.5 11.0 10.5 10.0 9.5 9.0 8.5 8.0 7.5 7.0 6.5 6.0 5.5 5.0 4.5 4.0 3.5 3.0 2.5 2.0 1.5 1.0 0.5 0.0 -0.5 -1.0 -1.5
f1 (ppm)

^{13}C NMR of **5d** in Chloroform-*d*

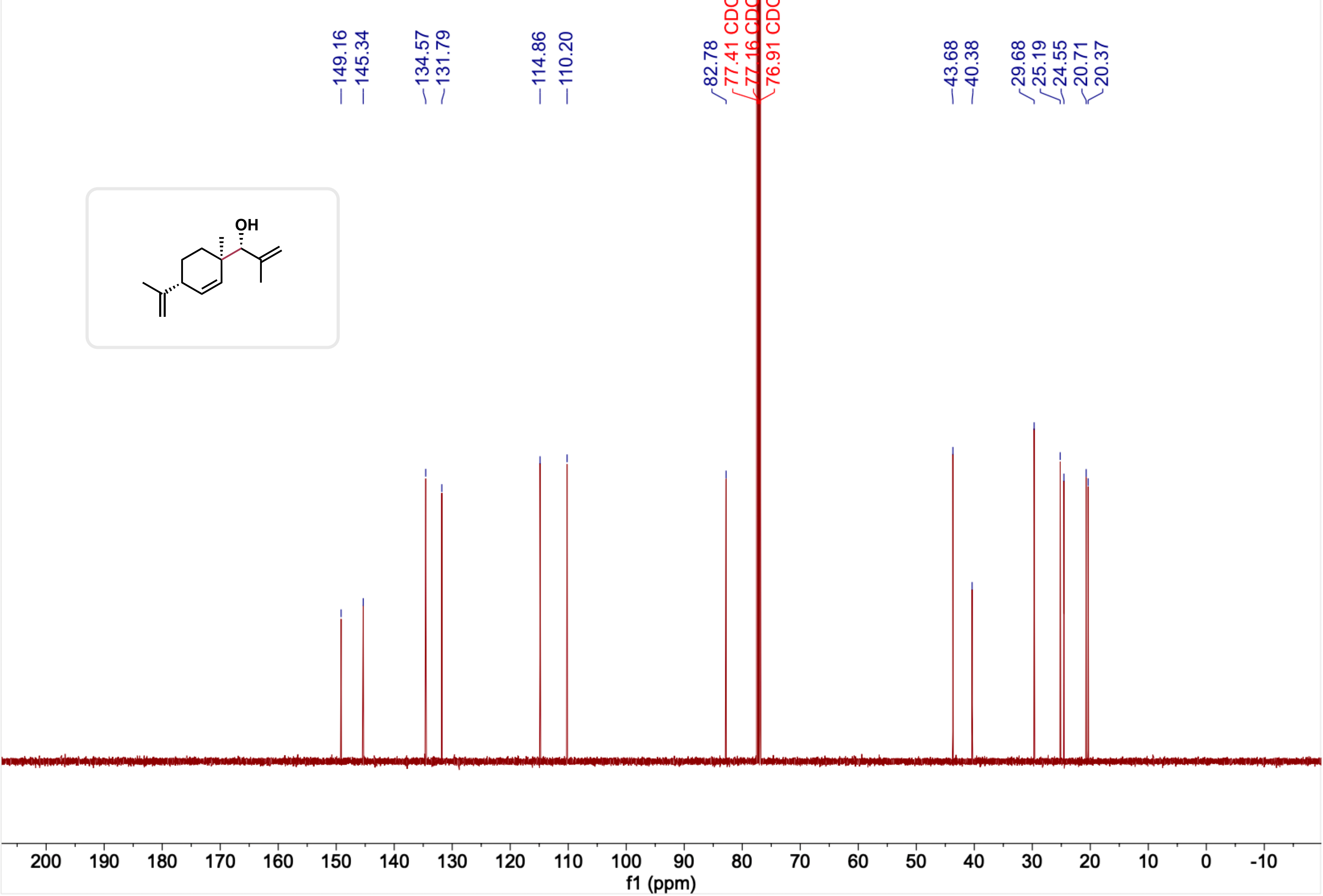

^{1}H NMR of **5e** in Chloroform-*d*

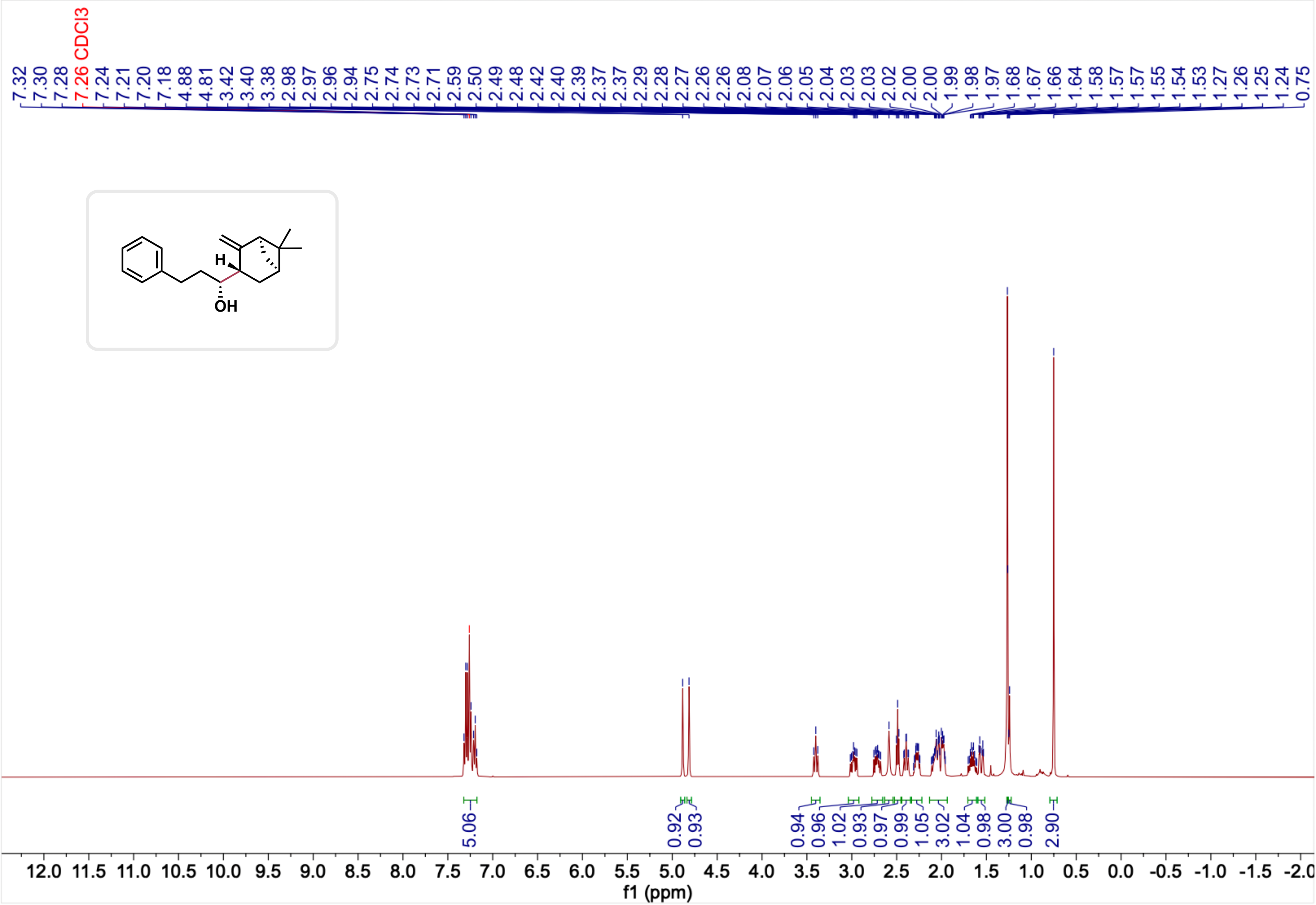

^{13}C NMR of **5e** in Chloroform-*d*

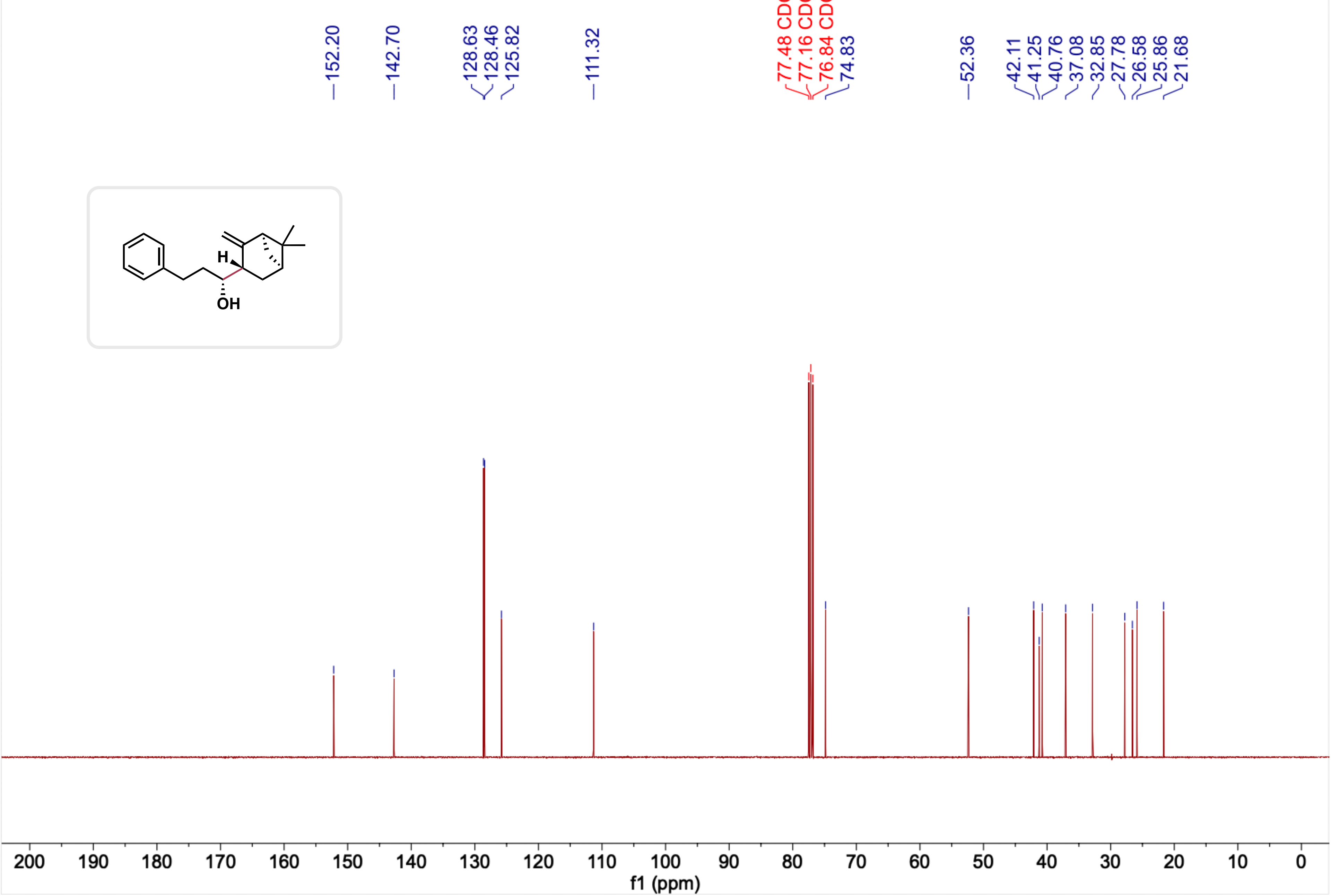

^{1}H-^{1}H NOESY NMR of **5e** in Chloroform-*d*

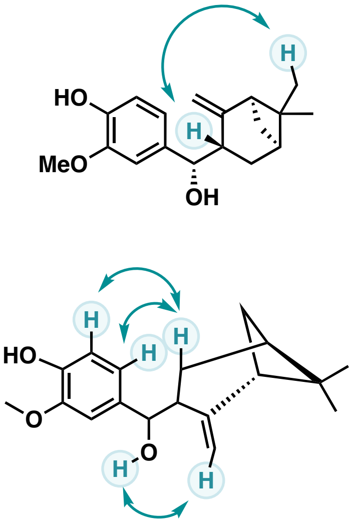

relative configuration determined based on NOESY

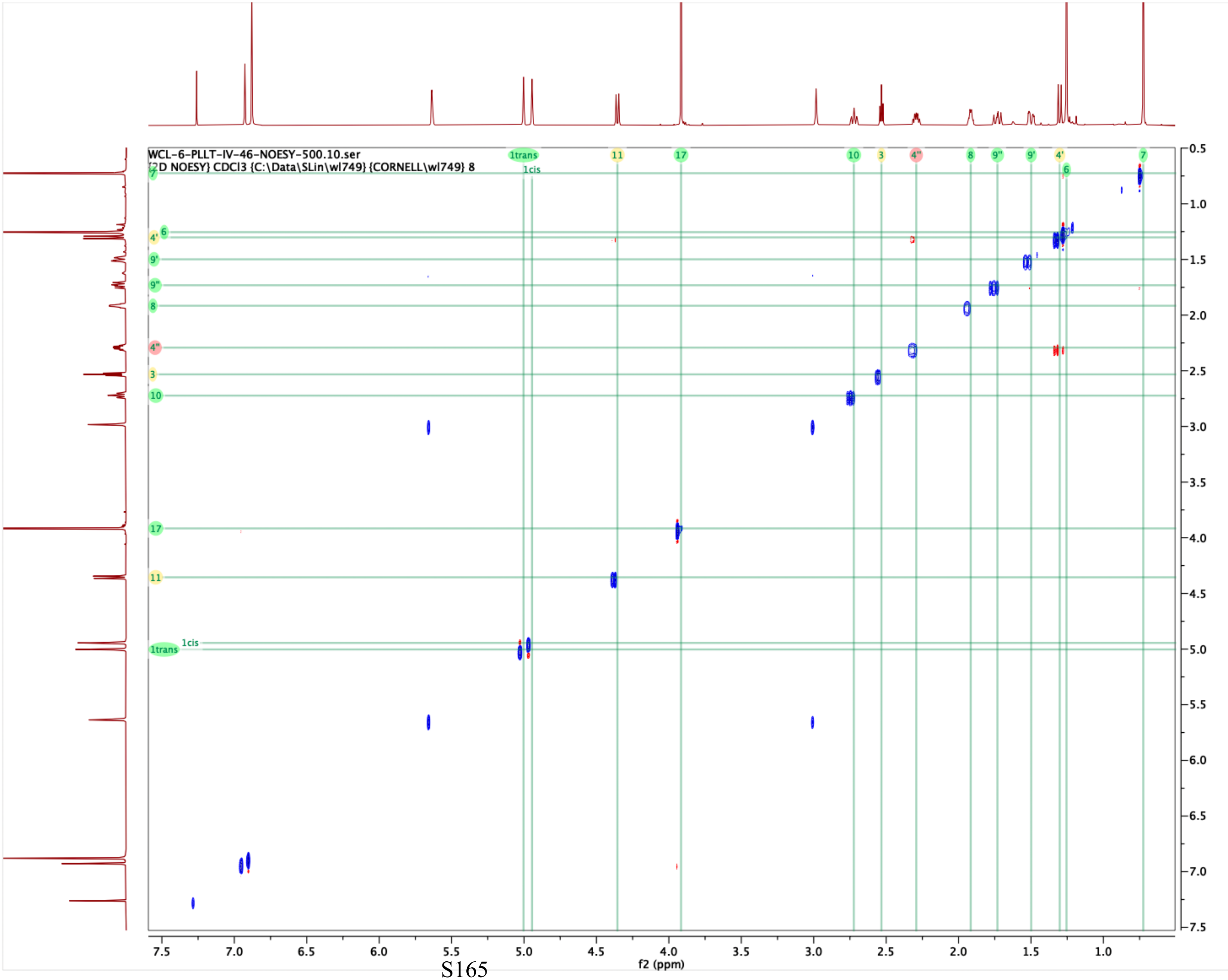

^{1}H NMR of **5f** in Chloroform-*d*

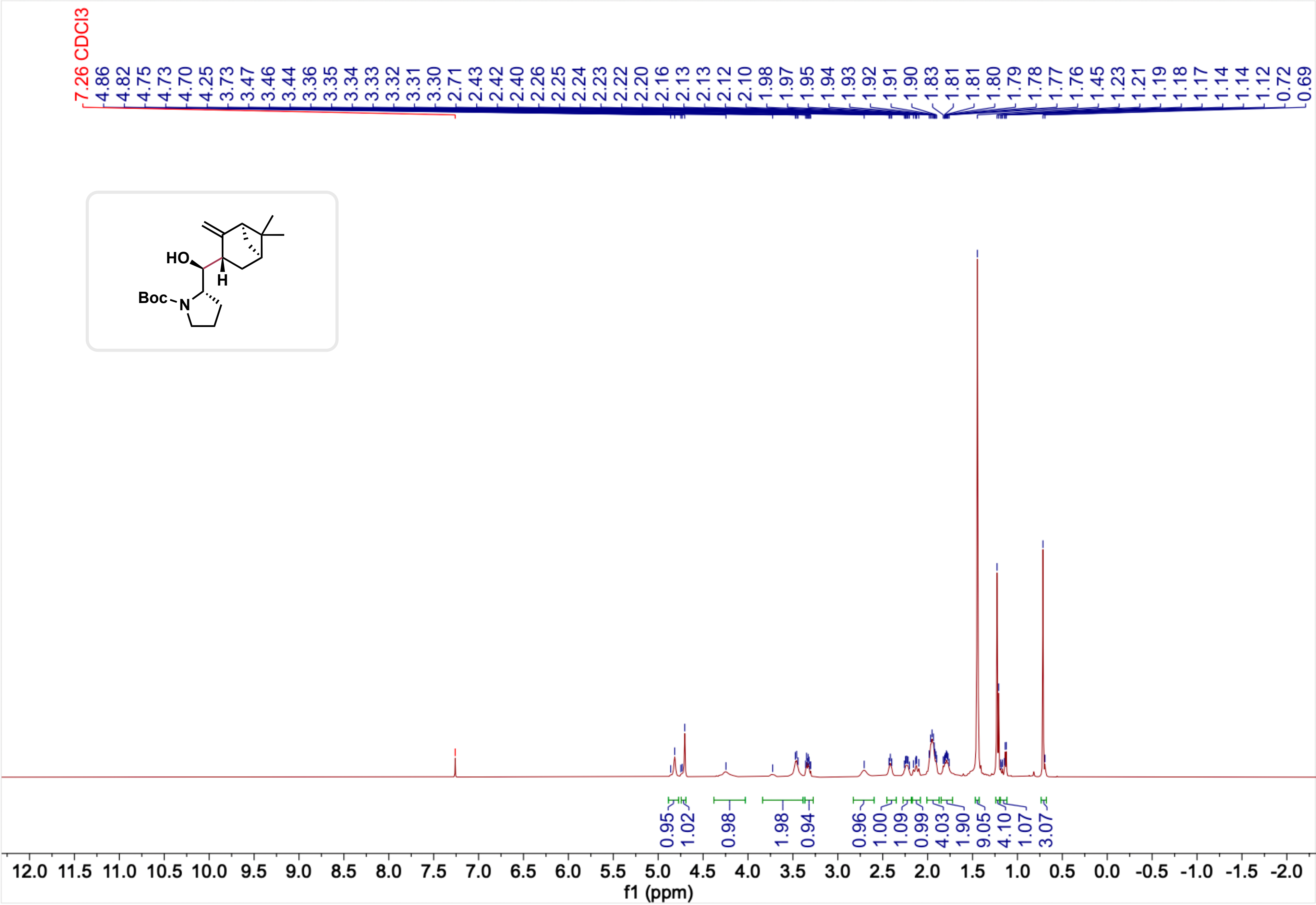

^{13}C NMR of **5f** in Chloroform-*d*

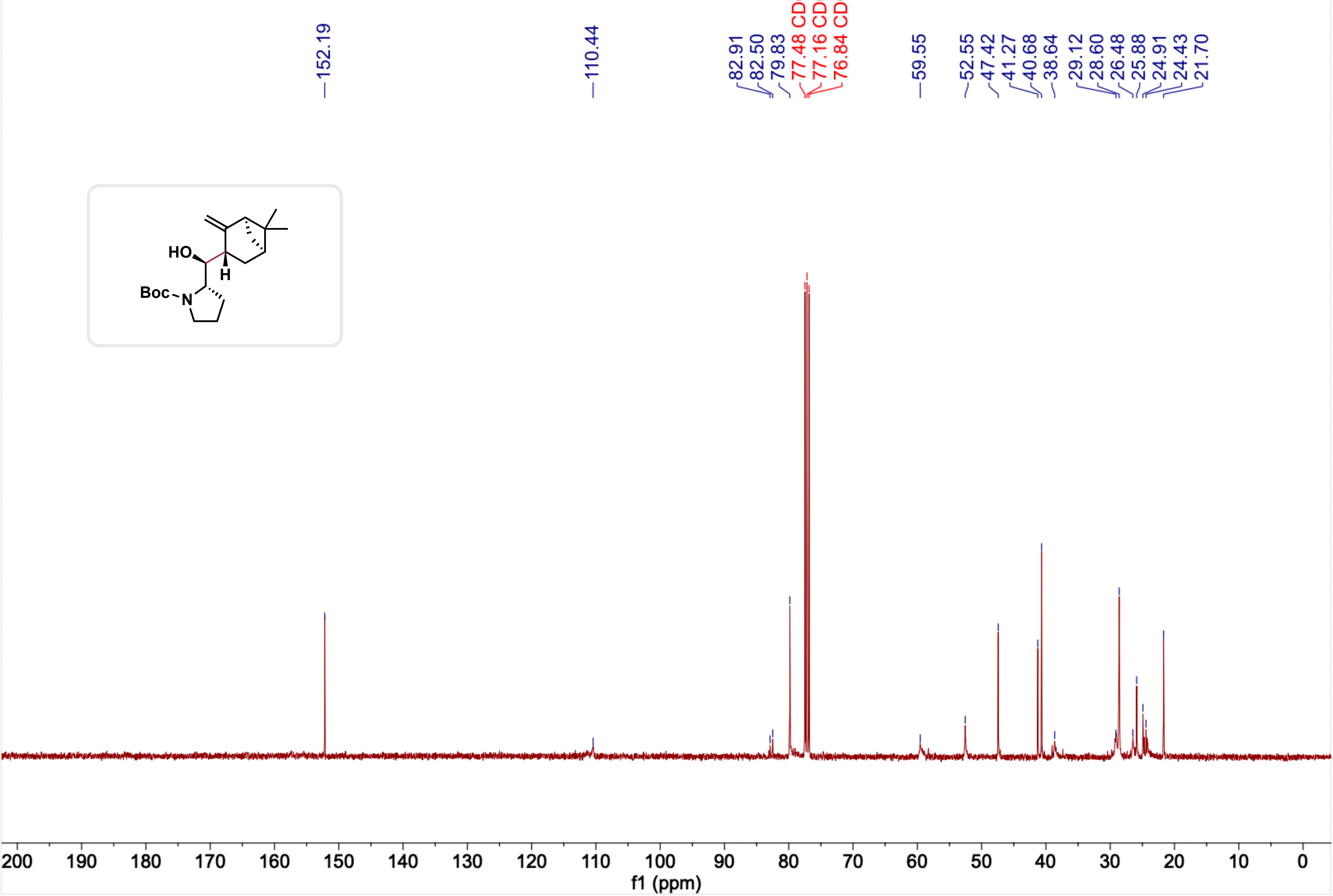

^{1}H NMR of **5g** in Chloroform-*d*

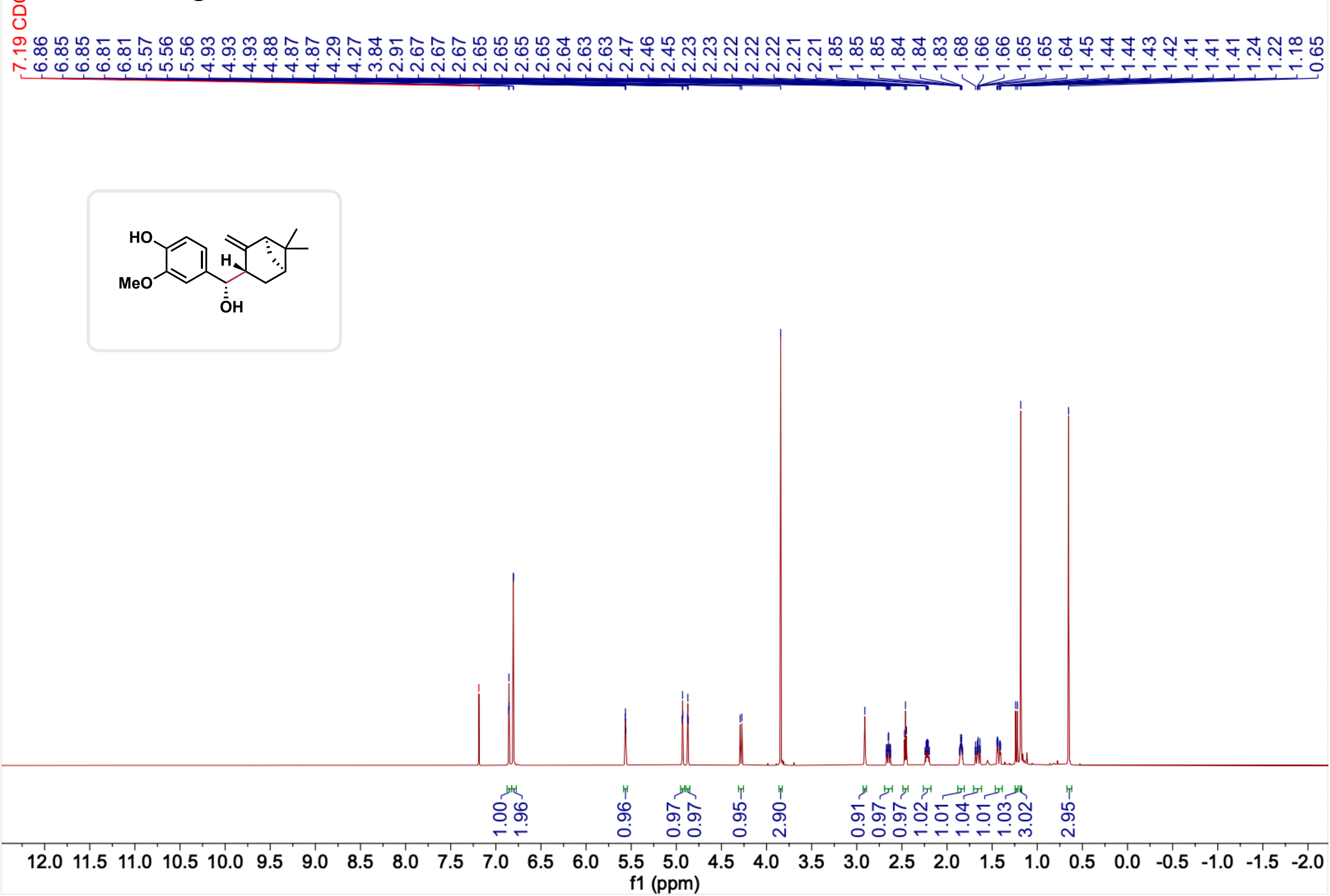

^{13}C NMR of **5g** in Chloroform-*d*

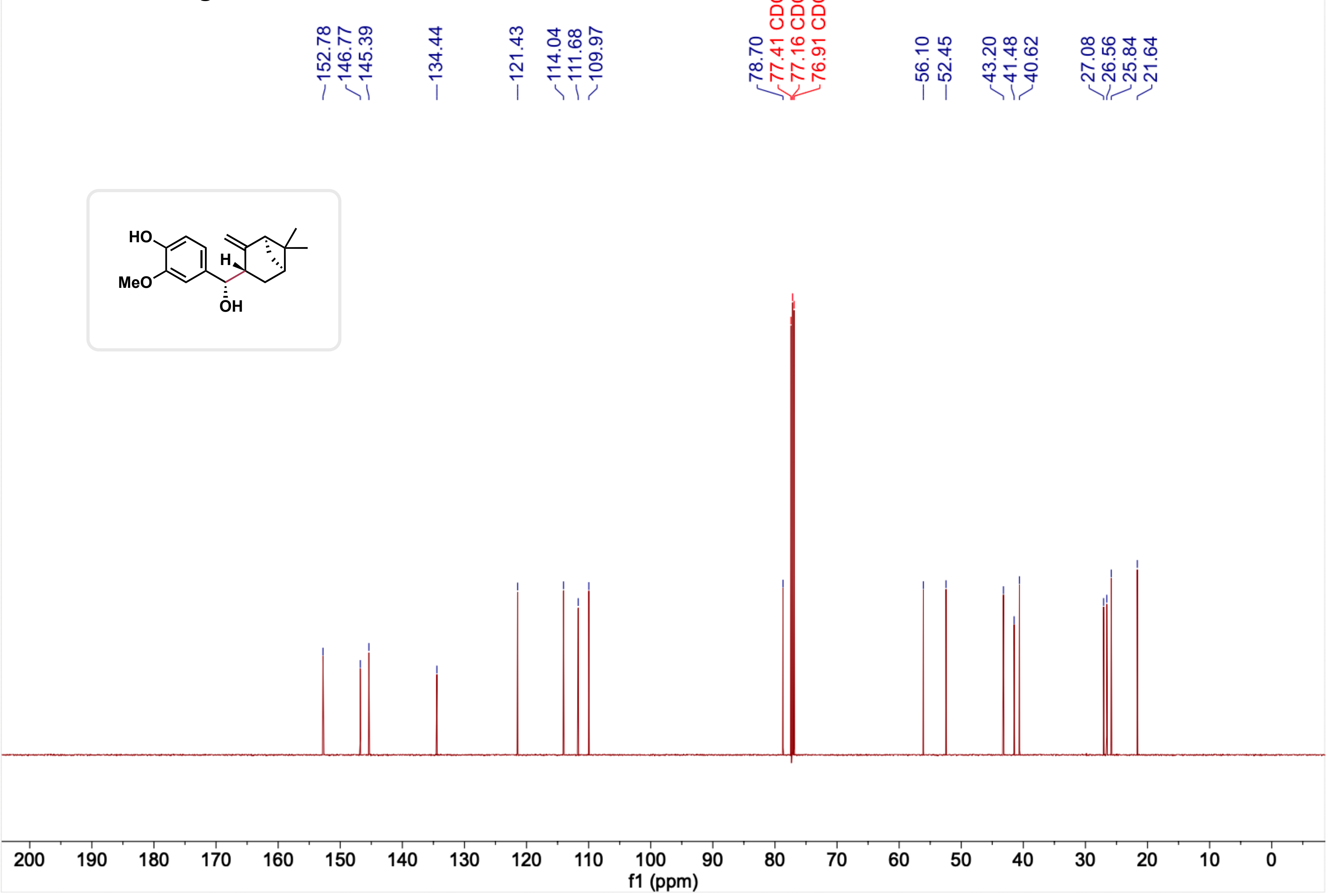

^1H NMR of **5h** in Chloroform-*d*

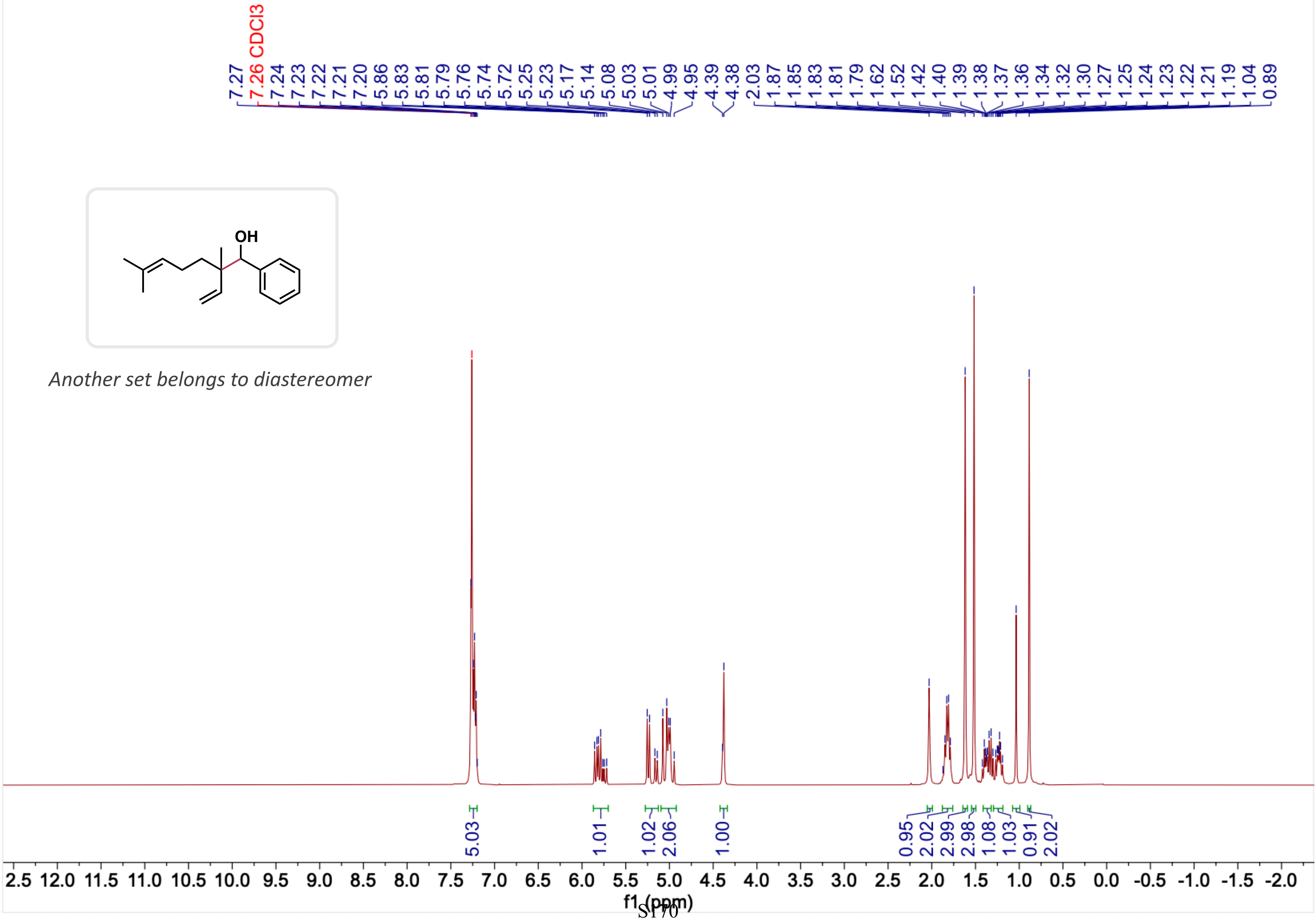

^{13}C NMR of **5h** in Chloroform-*d*

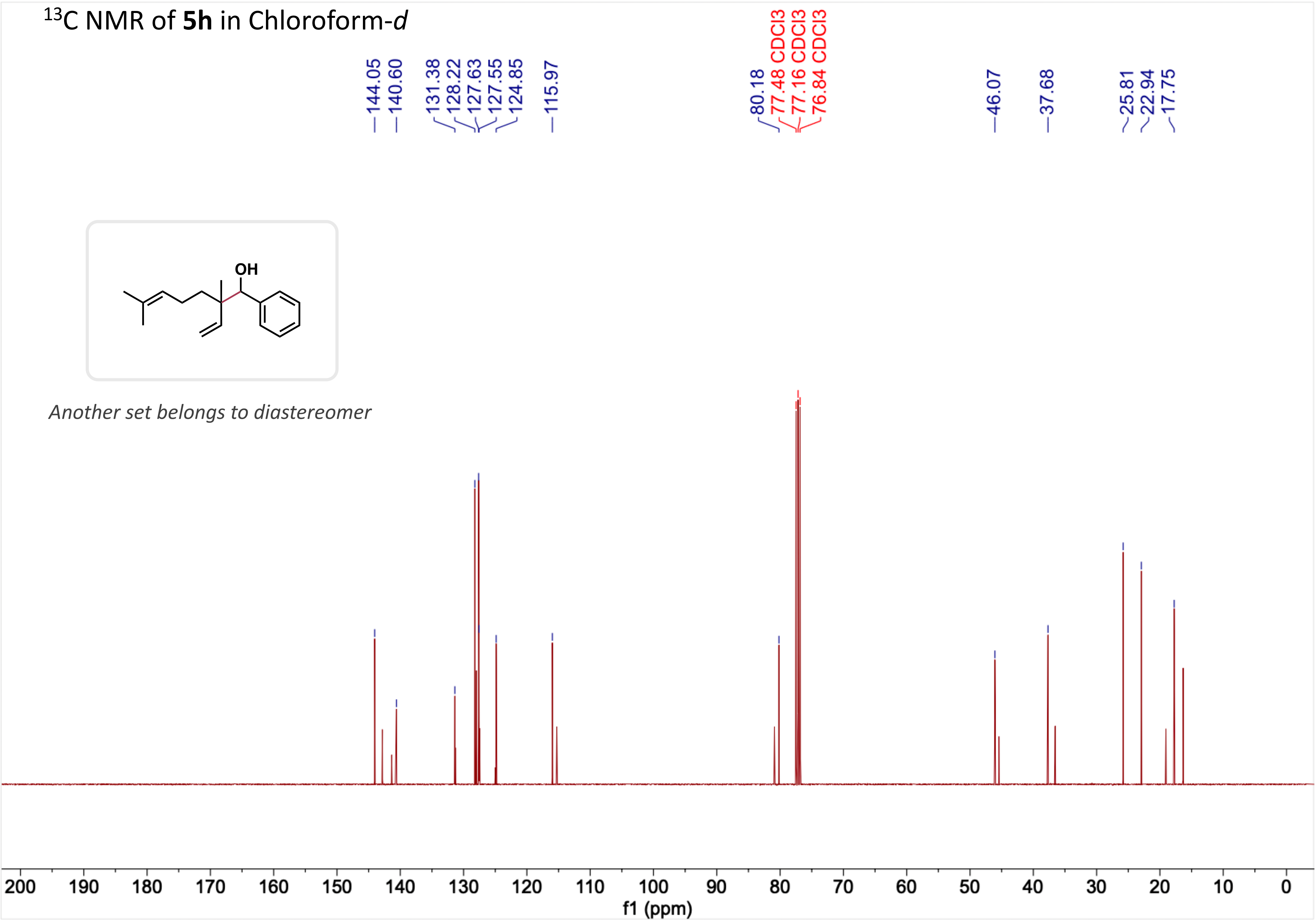

^{1}H NMR of **6a** in Chloroform-*d*

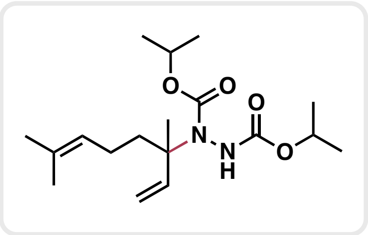

Another set belongs to rotamer

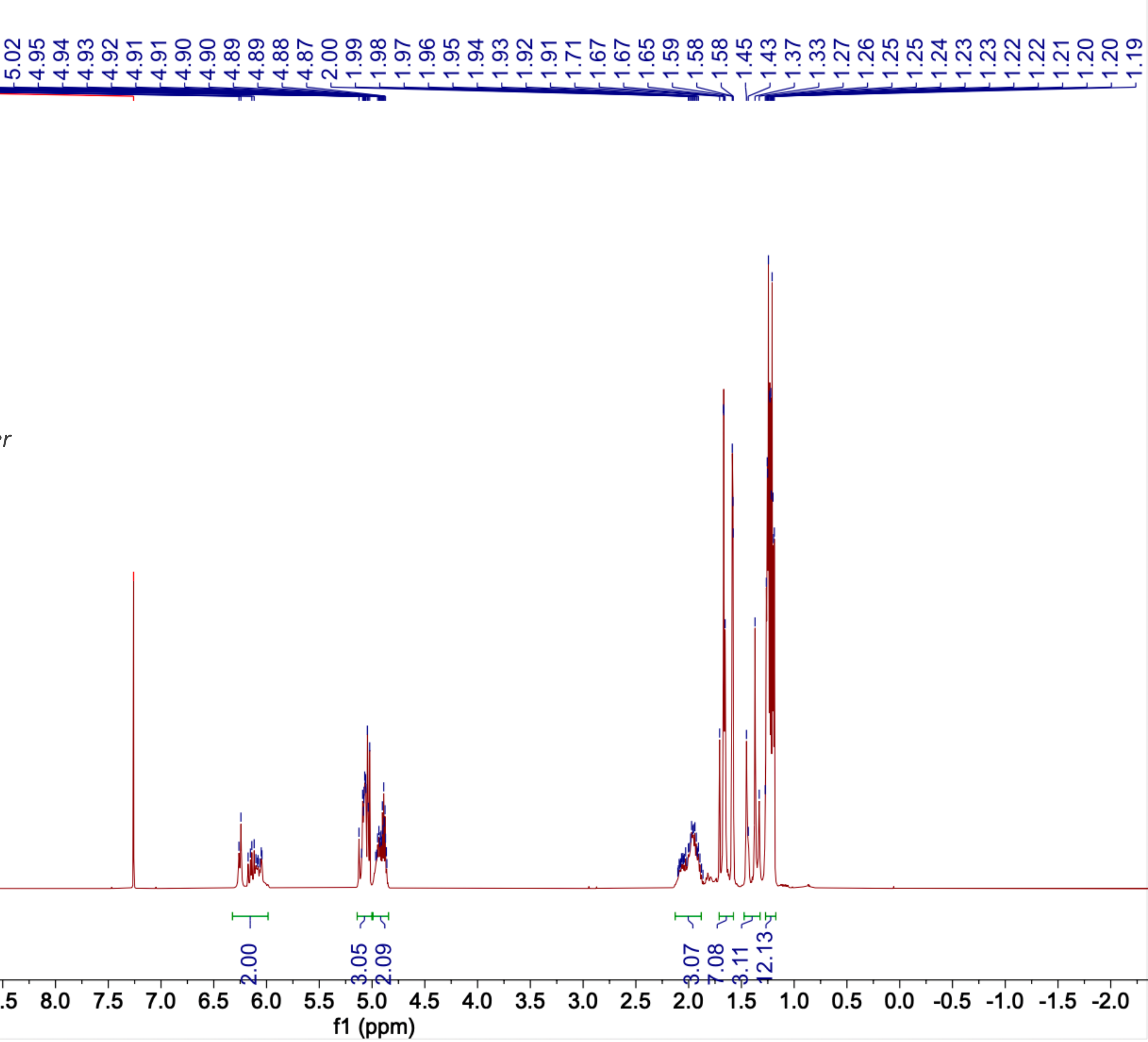

^{13}C NMR of **6a** in Chloroform-*d*

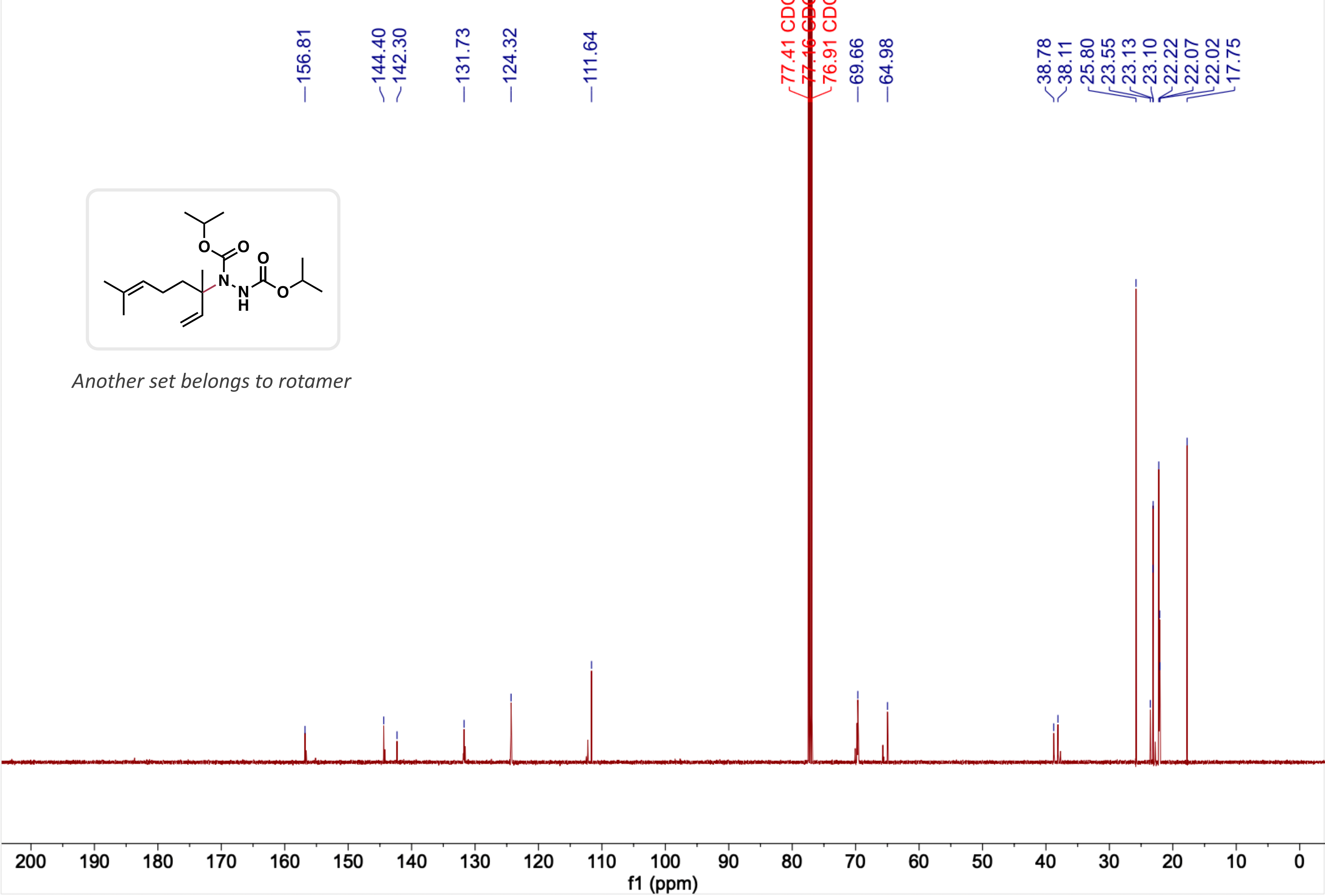

Another set belongs to rotamer
