# Supplementary material for: Regioselective Electrochemical Borylation of Oxygenated Allylic Electrophiles: Method Development and Synthetic Applications
Source: ACS Cent Sci. 2025 Sep 2;11(10):1959–68. doi: 10.1021/acscentsci.5c01074 (PMC12550627; doi:10.1021/acscentsci.5c01074)
Supplement: Supplementary file 2 [file oc5c01074_si_002.pdf]

oc-2025-01074x.R1

Name: Peer Review Information for "Regioselective Electrochemical Borylation of Oxygenated Allylic Electrophiles: Method Development and Synthetic Applications"

First Round of Reviewer Comments

Reviewer: 1

Comments to the Author

This manuscript describes an electrochemical borylation of oxygenated allylic electrophiles with good functional group tolerance and moderate to good yields. The experiments were performed in detail and the catalytic system showed excellent stereoselectivity. Moreover, a thorough exploration on the application approach was carried out further demonstrating the synthetic significance of this method. The reviewer conclude that this paper could be accepted for publication after minor revisions.

1. In Scheme 2B, by tuning the steric profile of the boron electrophile, alkoxy-substituted pinacolboranes led to marked improvements in regioisomeric ratio of the products compared to H–Bpin. Why did the authors explore the effect of cation using H–Bpin in Scheme 2C?

2. Are there any mechanistic studies performed to verify the reaction mechanism, such as cyclic voltammetry experiments?

3. Could X-ray crystallographic data collected for allylic boronates to confirm the geometrical structure?

4. It seems that two standard procedures were used in Scheme 2 (Lines 30-34 left column and Lines 29-32 right column). It should be clarified more clearly in explanatory text or experimental procedure.

Reviewer: 2

Comments to the Author

In their paper ”Regioselective Electrochemical Borylation of Oxygenated Allylic Electrophiles: Method Development and Synthetic Applications“ the Lin group extends their methodology of electrochemical borylation. A brief optimization of their previously published system enables high regioselectivity that is applied to a broad scope, natural products, and examples of sequenced syntheses.

In my view, the overall quality and degree of novelty justifies publication in ACS Central Science but some changes are recommended. There is a lack of clarity regarding the new contributions of this work to the field (Scheme 1), a screening that consists of 25 reactions and overlooks crucial aspects like reactant concentration, as well as scope entries that are identical to the original study by the authors without adequate indication. Additionally, especially when looking at multistep procedures and reaction sequences, a lot of confusion is created by inconsistent figures and insufficient clarification in the text. However, the choice of applications for this methodology is inspiring and comprises unintuitive transformations and valuable additions to/replacements of existing methods. After corrections outlined below, it will make for a great contribution in ACS Central Science.

The following improvements are recommended:

Page 1 right, line 33: One or even several literature references should be cited if referring to the “seminal contributions” to this field.

Scheme 1: While the general structure of A (feedstock), B (utility), and C (this work) is appealing, it does not become clear to the reader, what the actual contribution of this work is. Scheme 1C could well be found the preliminary study by the same authors, as it simply describes the mechanism of electrochemical deoxygenative borylation they discovered. Despite mentioning the challenge of regioselectivity, the extended methodology of this work is not part of Scheme 1. This is also resembled by the text on page 2 left. To improve clarity, reorganization of both Scheme 1 and the related text is necessary.

Page 2 left, lines 52-53: Is the linear product obtained with this methodology generally the more desired outcome of this reaction? Are there approaches to access the branched product?

Scheme 2 A: The number of equivalents of base catalyst and electrolyte should be added (to the text as well!) and the concentration of the reaction solution in DME.

Page 3 right, line 27: The screening presented in Table S1 is not at all detailed. Especially, concentrations of electrolyte and R-Bpin have not been screened albeit being crucial for considerations like atom economy and waste production of the process. Also, different boron compounds like BDAN, BBN, BMIDA could be taken into consideration enabling additional follow-up transformations.

Scheme 3 bottom: Four of the examples for lage-stage deoxygenative borylation are the exact same as in the original study by the same authors. What is more is that the values for yield and isomeric ratios are actually identical. If these were taken from the original publication, this should be indicated.

Page 5 left, lines 50-55: If no column chromatography was done to isolate the products of gram-scale syntheses (which is in accordance with the SI), the materials' purity should be assessed for example using 1H NMR with an internal standard and the spectra should be displayed in the SI.

Scheme 6 A: The depiction of the reaction sequence is different from the general procedure in the SI (page S20). Also, according to the SI, this reaction is not a one-pot procedure as indicated on page 5 left, line 2. In the SI, the product of the electrochemical reaction is displayed in square brackets that commonly indicate reactive intermediates which is irritating.

Scheme 6 B: Once again, the depiction of the sequence deviates from the SI (page S22). In this case, it is even more important, as the reaction mixture undergoes workup between H2O2 oxidation and DMP oxidation. Additionally, it remains unclear what steps (presumably of general procedure 6.1) are included this workup. In this case, the use of square brackets for a partially isolated intermediate is even more confusing.

Scheme 7A and 7B: In these figures, the intermediates displayed in square brackets are actually isolated according to the SI (page S23 and 26). Generally, it does not become clear from the text below Scheme 7, in which cases intermediates are immediately reacted further, worked up, or isolated.

Scheme 7C: Here, the figure correctly indicates that isolated intermediates are used. What are the yields of the single steps of this three-step transformation?

Scheme 8: It is unclear if there are any workups involved in this four-step sequence.

Reviewer: 3

Comments to the Author

Song Lin and co-workers describe herein a further development of their previously reported electroreductive approach for the deoxygenative borylation (J. Am. Chem. Soc. 2023, 145, 16966–16972). Through modified conditions regioselective borylations of allylic alcohols and diverse α,β-unsaturated carbonyl compounds were achieved to access sterically congested allylic boronates. Synthetic application was subsequently demonstrated with the borylation of terpenoid natural products, an efficient scale-up and different synthetic procedures for, amongst other, alcohol and carbonyl transposition, vinylogous homologation and formal cross-coupling reactions.

Comments:

• The introduction is well written and covers the important literature background as well as the limitation that the presented research aims to overcome. To highlight the role of electrochemistry in this field of study, the authors may add related work on borylation (e.g., Ackermann, CCS Chem. 2024, 6, 1430–1438.) to their references.

• The optimization of the reaction` s efficiency and selectivity were guided by steric tuning. Thereby, the authors describe a pronounced effect of the size of the counterion derived from the electrolyte. As a sacrificial magnesium anode is used for all experiments, the substantially more Lewis acidic magnesium ions are also present and should be considered in this rationale. Other sacrificial anodes may be examined to assess the effect of this factor.

o Alternatively, as it was shown in the previous study for the deoxygenative borylation (J. Am. Chem. Soc. 2023, 145, 16966–16972), the reaction also works in a divided cell as well as with DIPEA as a sacrificial reductant. The influence of these experiments on the regioisomeric ratio might also be insightful.

• Does the anion of the electrolyte influence the reaction outcome? As triflate may be chemically involved and could also influence the association of the cation with the proposed allyl anion, other electrolytes might be worth investigating.

• Concerning the scope, the authors show a relatively broad set of differently substituted substrates. Still, mostly non-functionalized aliphatic and aromatic compounds are presented. Does the reaction tolerate more sensitive functional groups?

• With respect to the allylic alcohols, mostly tertiary alcohols were utilized. How does the developed strategy perform with secondary or primary alcohols?

• The authors may comment on the advantages of their established reaction sequences compared to traditional ways of synthesizing the compounds. Especially, the approach for the homologation consists of four steps, whereby three of them are still working with stoichiometric, waste-generating reactants.

• Regarding the SI:

o The authors may indicate the solvent, or the solvent mixture used for purification by flash chromatography for the individual compounds

o The mass data of some compounds shows a relatively large deviation for HRMS. Following compounds might be revisited: 1d, 2k, 2m, 2n, 2p

o The multiplicities provided in the 1H-NMR data might be checked for following compounds as they seem implausible: 2h, 2k

o The authors should include the method for crystallization

Author's Response to Peer Review Comments:

Reviewer 1

This manuscript describes an electrochemical borylation of oxygenated allylic electrophiles with good functional group tolerance and moderate to good yields. The experiments were performed in detail and the catalytic system showed excellent stereoselectivity. Moreover, a thorough exploration on the application approach was carried out further demonstrating the synthetic significance of this method. The reviewer conclude that this paper could be accepted for publication after minor revisions.

We thank the reviewer for the positive evaluation of our manuscript and for recommending its publication in *ACS Central Science* after minor revisions. We also appreciate the reviewer's insightful suggestions, which have helped improve the clarity and overall quality of the manuscript.

1. In Scheme 2B, by tuning the steric profile of the boron electrophile, alkoxy-substituted pinacolboranes led to marked improvements in regioisomeric ratio of the products compared to H–Bpin. Why did the authors explore the effect of cation using H–Bpin in Scheme 2C?

Response: We appreciate the reviewer's insightful question. As shown in Scheme 2D, the cation has minimal impact on regioselectivity when MeOBpin was used as the boron electrophile, as MeOBpin already gives >20:1 r.r. with the smallest Li^+ ion. Nevertheless, the counterion had a significant effect on the yield of the reaction, with KOTf proving to be optimal. The counterion effect was more pronounced when using H–Bpin as the electrophile, as demonstrated in Scheme 2C.

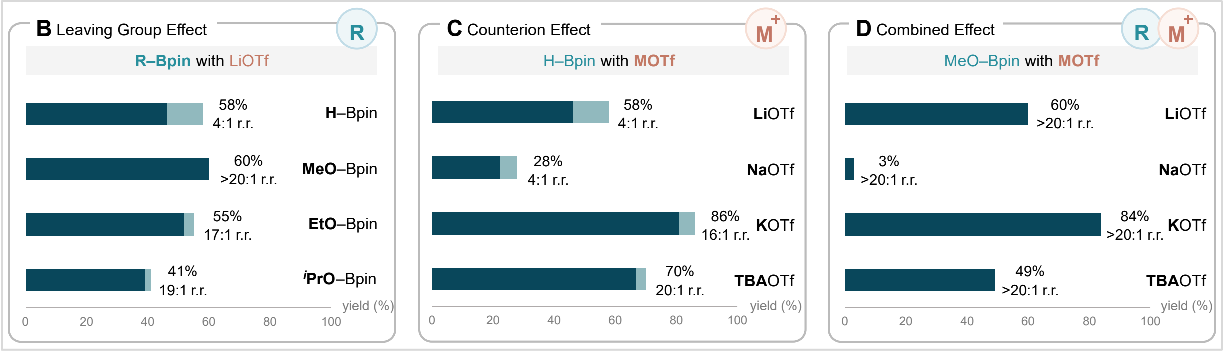

2. Are there any mechanistic studies performed to verify the reaction mechanism, such as cyclic voltammetry experiments?

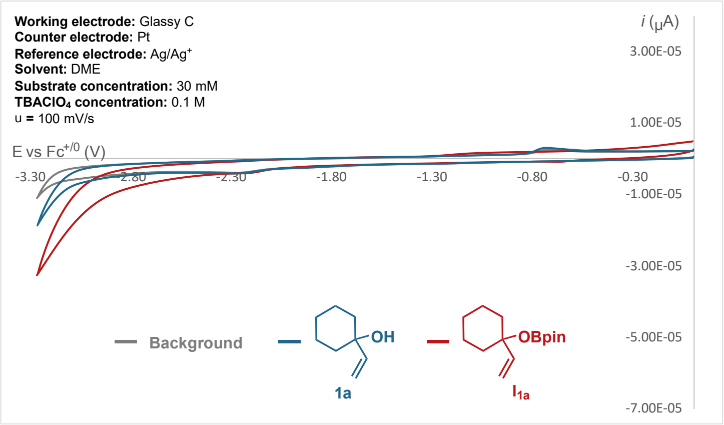

Response: We have indeed performed cyclic voltammetry studies on vinyl alcohol 1a and the corresponding borate complex I_{1a}. However, no distinct reduction peaks were observed in the CV experiments. A sluggish reduction onset was seen at <–2.30 V, which was not observed for allylic alcohol 1a. This has been included in the revised SI.

In our previous study (*J. Am. Chem. Soc.* 2023, *145*, 16966), we demonstrated that Bn–OBpin can be reduced at –2.5 V vs. $Fc^{+/0}$. Based on these data, we propose a similar mechanistic pathway in the current study. Because this work is focused primarily on the synthetic utility of the method, we did not include extensive mechanistic investigations, which will be a focus of our future work.

3. Could X-ray crystallographic data collected for allylic boronates to confirm the geometrical structure?

Response: Although we attempted to grow crystals of the allylic boronate products, the oily physical state of most of the compounds we synthesized hindered the formation of suitable crystals. However, we have confirmed the regiochemistry and structures of the products using 2D NMRs. In addition, we obtained a crystal structure for compound 5f after reaction of allylboron 2t with an aldehyde, which indirectly supports the structural assignment of 2t.

4. It seems that two standard procedures were used in Scheme 2 (Lines 30-34 left column and Lines 29-32 right column). It should be clarified more clearly in explanatory text or experimental procedure.

Response: As suggested, we have included both procedures in the General Procedure of the Supporting Information.

Reviewer 2

In their paper ”Regioselective Electrochemical Borylation of Oxygenated Allylic Electrophiles: Method Development and Synthetic Applications“ the Lin group extends their methodology of electrochemical borylation. A brief optimization of their previously published system enables high regioselectivity that is applied to a broad scope, natural products, and examples of sequenced syntheses. In my view, the overall quality and degree of novelty justifies publication in ACS Central Science but some changes are recommended. There is a lack of clarity regarding the new contributions of this work to the field (Scheme 1), a screening that consists of 25 reactions and overlooks crucial aspects like reactant concentration, as well as scope entries that are identical to the original study by the authors without adequate indication. Additionally, especially when looking at multistep procedures and reaction sequences, a lot of confusion is created by inconsistent figures and insufficient clarification in the text. However, the choice of applications for this methodology is inspiring and comprises unintuitive transformations and valuable additions to/replacements of existing methods. After corrections outlined below, it will make for a great contribution in ACS Central Science.

We are grateful to the reviewer for the positive comments on our work and for recommending the publication of this manuscript in *ACS Central Science* after revisions. We are especially encouraged by the reviewer’s highest rating (Top 1%) for novelty and quality of experimental data, technical rigor. We also appreciate the reviewer’s valuable suggestions, which have helped us further strengthen and clarify the manuscript.

1. Page 1 right, line 33: One or even several literature references should be cited if referring to the “seminal contributions” to this field.

Response: As suggested, we have included the appropriate references in the corresponding sentence of the main text (refs. 41–42, 45, 49).

2. Scheme 1: While the general structure of A (feedstock), B (utility), and C (this work) is appealing, it does not become clear to the reader, what the actual contribution of this work is. Scheme 1C could well be found the preliminary study by the same authors, as it simply describes the mechanism of electrochemical deoxygenative borylation they discovered. Despite mentioning the challenge of regioselectivity, the extended methodology of this work is not part of Scheme 1. This is also resembled by the text on page 2 left. To improve clarity, reorganization of both Scheme 1 and the related text is necessary.

Response: We appreciate the reviewer’s suggestion and have added Scheme 1D summarize our current study and cited this figure in the main text of the introduction where we discuss the advances in this work.

3. Page 2 left, lines 52-53: Is the linear product obtained with this methodology generally the more desired outcome of this reaction? Are there approaches to access the branched product?

Response: Under the optimal conditions, the linear product was predominantly formed. While the branched product could be observed under modified conditions, the regioselectivity remained low, as previously reported in our earlier study (*J. Am. Chem. Soc.* 2023, *145*, 16966).

4. Scheme 2 A: The number of equivalents of base catalyst and electrolyte should be added (to the text as well!) and the concentration of the reaction solution in DME.

Response: As requested, we have revised Scheme 2 accordingly.

5. Page 3 right, line 27: The screening presented in Table S1 is not at all detailed. Especially, concentrations of electrolyte and R-Bpin have not been screened albeit being crucial for considerations like atom economy and waste production of the process. Also, different boron compounds like BDAN, BBN, BMIDA could be taken into consideration enabling additional follow-up transformations.

Response: We have added an optimization detailing the effects of electrolyte concentration and MeO–Bpin loading on the model reaction to Table S1 (also see below).

We appreciate the reviewer's suggestion of testing other boron electrophiles. Indeed, we have considered this during our initial reaction development. However, species such as H–BMIDA, MeO–BMIDA, Cl–BMIDA, H–BDAN, MeO–BDAN, and Cl–BDAN are commercially unavailable. In addition, we evaluated 9-BBN as an alternative electrophile, however, no desired product formation was observed. Because MeOBpin gave satisfactory outcomes for most of the substrates that we tested, we did not further pursue the use of other boron electrophiles. Because the conversion of Bpin to other boron-containing groups are well documented (e.g., to BMIDA: (*J. Am. Chem. Soc.* 2023, *145*, 7548.); to BDAN: (*Angew. Chem. Int. Ed.* 2018, *57*, 6146.); to BF_3K: (*Tetrahedron Let.* 2022, *104*, 154019.)), our products could be transformed to include these groups should it be necessary in follow-up synthetic operations.

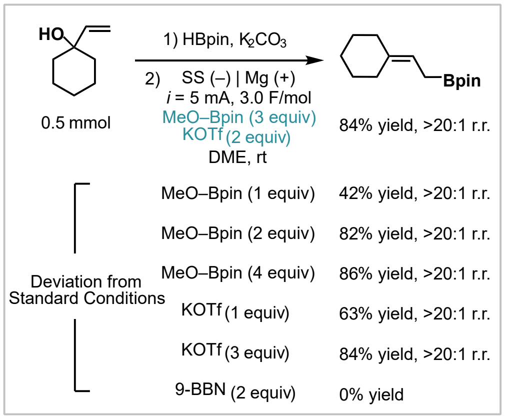

6. Scheme 3 bottom: Four of the examples for lage-stage deoxygenative borylation are the exact same as in the original study by the same authors. What is more is that the values for yield and isomeric ratios are actually identical. If these were taken from the original publication, this should be indicated.

Response: We indeed included results from our previous publication to direct compare the yield and regioselectivity using the previous vs. the newly developed conditions. In all cases, the new conditions provided higher yield and r.r. than the previous conditions. In the original manuscript, we included footnote (d) in Scheme 3 to denote yields using original conditions. In the revision, we added text in footnote (d) to state which data are directly taken from previous paper.

7. Page 5 left, lines 50-55: If no column chromatography was done to isolate the products of gramscale syntheses (which is in accordance with the SI), the materials' purity should be assessed

for example using 1H NMR with an internal standard and the spectra should be displayed in the SI.

Response: As suggested, we have included the ^{1}H NMR spectra of the crude reaction mixture with an internal standard in the Supporting Information. The purity of products was calculated to be ≥95%.

8. Scheme 6 A: The depiction of the reaction sequence is different from the general procedure in the SI (page S20). Also, according to the SI, this reaction is not a one-pot procedure as indicated on page 5 left, line 2. In the SI, the product of the electrochemical reaction is displayed in square brackets that commonly indicate reactive intermediates which is irritating.

Response: We thank the reviewer for the suggestions and have revised both the main text and the procedures in the Supporting Information to ensure consistency and clarity. We have also removed the term "one-pot" from the main text and eliminated the square brackets in the equation in the SI, as suggested.

9. Scheme 6 B: Once again, the depiction of the sequence deviates from the SI (page S22). In this case, it is even more important, as the reaction mixture undergoes workup between H2O2 oxidation and DMP oxidation. Additionally, it remains unclear what steps (presumably of general procedure 6.1) are included this workup. In this case, the use of square brackets for a partially isolated intermediate is even more confusing.

Response: We have revised both the main text and the procedures in the Supporting Information to ensure consistency. We have also removed the square brackets in the equation in the SI.

10. Scheme 7A and 7B: In these figures, the intermediates displayed in square brackets are actually isolated according to the SI (page S23 and 26). Generally, it does not become clear from the text below Scheme 7, in which cases intermediates are immediately reacted further, worked up, or isolated.

Response: We have revised the procedure in the Supporting Information to clarify that the intermediate was not isolated by flash chromatography. Additionally, we have specified in the footnote that an aqueous wash workup was required after the electrolysis step. The square brackets in Scheme 7 have also been removed.

11. Scheme 7C: Here, the figure correctly indicates that isolated intermediates are used. What are the yields of the single steps of this three-step transformation?

Response:

As suggested, we have included the yield information for each intermediate in Scheme 7C.

12. Scheme 8: It is unclear if there are any workups involved in this four-step sequence.

Response: This procedure involved an aqueous wash workup following the allylation step. The crude mixture obtained after electrolysis was used directly in the subsequent oxidation without further workup or purification. After oxidation with H_2O_2, an aqueous wash workup was performed prior to the addition of DMP for the final oxidation step. We have revised the experimental procedures in the Supporting Information to improve clarity.

Reviewer 3

Song Lin and co-workers describe herein a further development of their previously reported electroreductive approach for the deoxygenative borylation (J. Am. Chem. Soc. 2023, 145, 16966–16972). Through modified conditions regioselective borylations of allylic alcohols and diverse α,β-unsaturated carbonyl compounds were achieved to access sterically congested allylic boronates. Synthetic application was subsequently demonstrated with the borylation of terpenoid natural products, an efficient scale-up and different synthetic procedures for, amongst other, alcohol and carbonyl transposition, vinylogous homologation and formal cross-coupling reactions.

We appreciate the reviewer's positive remarks on this manuscript and their recommendation for publication in *ACS Central Science* following minor revisions. We are encouraged by the reviewer's highest rating (Top 1%) in terms of novelty, significance, broad interest, and quality of experimental data, technical rigor. We also thank the reviewer for their constructive suggestions, which have helped enhance the clarity and overall quality of this work.

1. The introduction is well written and covers the important literature background as well as the limitation that the presented research aims to overcome. To highlight the role of electrochemistry in this field of study, the authors may add related work on borylation (e.g., Ackermann, CCS Chem. 2024, 6, 1430–1438.) to their references.

Response: As suggested, we have included the reference as ref. 73 in the main text.

2. The optimization of the reaction's efficiency and selectivity were guided by steric tuning. Thereby, the authors describe a pronounced effect of the size of the counterion derived from the electrolyte. As a sacrificial magnesium anode is used for all experiments, the substantially more Lewis acidic magnesium ions are also present and should be considered in this rationale. Other sacrificial anodes may be examined to assess the effect of this factor.

Response:

We appreciate the reviewer's insightful suggestions. To evaluate the potential counterion effect from metal ions generated by the oxidation of sacrificial anodes, we conducted the borylation reaction using various anodes beyond magnesium. Notably, differences in regioselectivity were observed with alternative anodes such as zinc, iron, and aluminum, suggesting that metal ions released from the anodes can influence regioselective control. These results have been included in Table S2 and are now discussed in the main text.

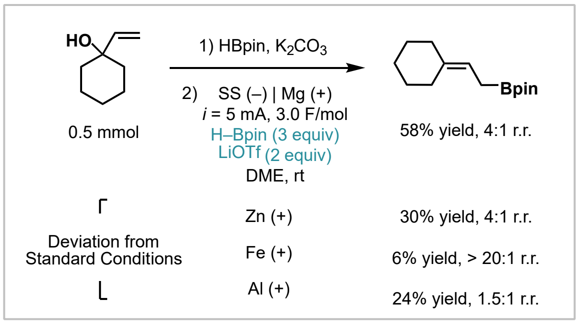

3. Alternatively, as it was shown in the previous study for the deoxygenative borylation (J. Am. Chem. Soc. 2023, 145, 16966—16972), the reaction also works in a divided cell as well as with DIPEA as a sacrificial reductant. The influence of these experiments on the regioisomeric ratio might also be insightful.

Response: We performed the borylation reaction in a divided cell and also evaluated conditions using DIPEA as a sacrificial reductant. We found that the regioselectivity decreased under the DIPEA conditions, whereas it remained unchanged in the divided cell setup. These results have been included in Table S2. The r.r. changes are relatively small, and we do not fully understand these changes at this stage. It is possible that either DIPEA itself or the side products generated from DIPEA oxidation could affect the selectivity.

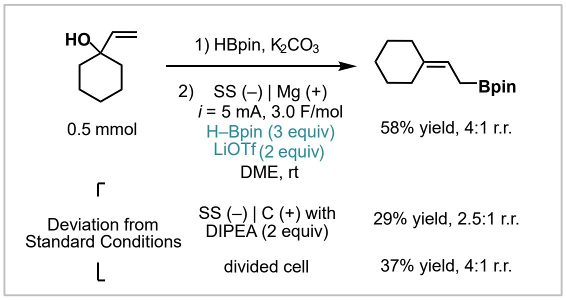

4. Does the anion of the electrolyte influence the reaction outcome? As triflate may be chemically involved and could also influence the association of the cation with the proposed allyl anion, other electrolytes might be worth investigating.

Response: We have conducted a series of borylation reactions using different lithium electrolytes, including $LiPF_6$, $LiClO_4$, LiCl, and LiTFSI. The results indicate that the anion of the electrolyte has a slight influence on regioselective control. These findings have been included in Table S2.

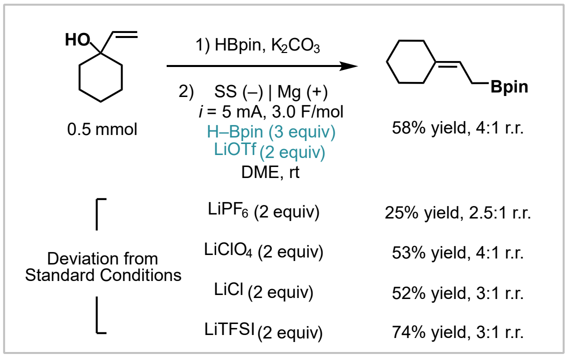

Response:

5. Concerning the scope, the authors show a relatively broad set of differently substituted substrates. Still, mostly non-functionalized aliphatic and aromatic compounds are presented. Does the reaction tolerate more sensitive functional groups?

In our study, we have demonstrated that the borylation reaction is compatible with several functional groups, including ketal, carbamate, silylether, tertiary amine, and tertiary alcohol. We did not test functional group tolerance extensively because we mainly focused on terpene-derived allylic alcohols for the construction of molecules with complex skeletons.

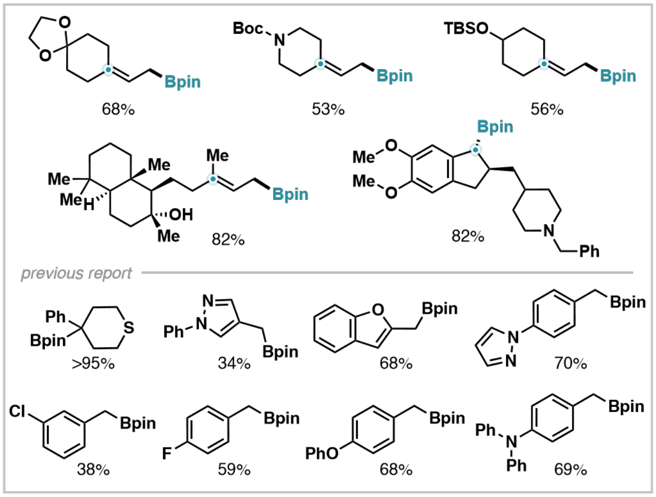

In addition to the examples provided in this work, our previous study (*J. Am. Chem. Soc.* 2023, *145*, 16966) further established that the electrochemical borylation platform is compatible with thioether, aryl fluoride, aryl amine, and various heterocycles such as benzofuran and pyrazole. Aryl chloride, however, undergoes competitive dehalogenation under the reaction conditions. In addition, substrates including a triazole, an indazole, or an *N*,*N*-dialkylamide provided low yield (~30 %).

6. With respect to the allylic alcohols, mostly tertiary alcohols were utilized. How does the developed strategy perform with secondary or primary alcohols?

Response: Under the optimal conditions, the electrochemical deoxygenative borylation effectively activates both secondary and primary alcohols, as demonstrated in our substrate scope studies using Myrtenol and Carveol as representative examples. With Myrenol, the reaction proceeds preferentially at the less sterically hindered site, affording the corresponding product 2t.

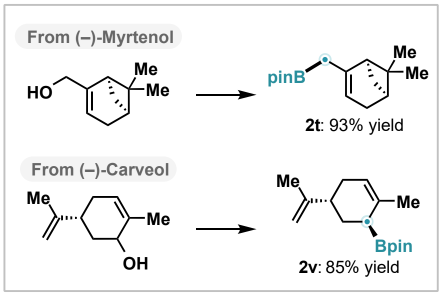

7. The authors may comment on the advantages of their established reaction sequences compared to traditional ways of synthesizing the compounds. Especially, the approach for the homologation consists of four steps, whereby three of them are still working with stoichiometric, waste-generating reactants.

Response: We appreciate the reviewer's sentiment and agree that the current four-step homologation sequence is not better than most efficient known strategies for the same synthesis in terms of step count and use of stoichiometric reagents. Nonetheless, it provides a complementary, metal-free, and highly selective approach that may be applicable in complex target synthesis. In addition to homologation, we also present two additional transformations that would be challenging to achieve

Response:
using alternative synthetic strategies, including the transposition of alcohols and carbonyls as well as the formal coupling of allylic alcohols and aldehydes, both achieved with excellent regioselectivity.

8. The authors may indicate the solvent, or the solvent mixture used for purification by flash chromatography for the individual compounds.

Response: As suggested, we have included the solvent system used for flash chromatography in the Supporting Information.

Response:

9. The mass data of some compounds shows a relatively large deviation for HRMS. Following compounds might be revisited: 1d, 2k, 2m, 2n, 2p.

Response: We thank the reviewer for the careful examination of our data. We have re-checked the characterization data for compounds 1d, 2k, 2m, 2n, and 2p (0.0005 to 0.0009 mass unit deviation), and confirm that the values fall within an acceptable range of experimental deviation. We respectfully leave it to the editor's discretion to determine whether any further experimentation is required.

10. The multiplicities provided in the 1H-NMR data might be checked for following compounds as they seem implausible: 2h, 2k.

Response: Upon re-checking the characterization data, we have revised the reported multiplicities for compounds 2h and 2k accordingly.

11. The authors should include the method for crystallization.

Response: As suggested, we have included the method used for crystallization in the Supporting Information.

Manuscript Formatting Request

1. Author List: Please include the email address(es) of the corresponding author(s) on the first page of the manuscript.

Response: We have added the email address of the corresponding author on the first page.

2. Graphics: If a figure has parts labeled (i.e. a, b, etc.), each part must be mentioned in the figure caption.

Response: As requested, we have revised the corresponding schemes.

3. References: Number references individually, with only one citation per reference. Do not group references.

Response: As requested, we have made the corresponding revision.

4. Synopsis: ACS Central Science requires a brief synopsis. The synopsis should be no more than 200 characters (including spaces) and should reasonably correlate with the Table of Contents (TOC) graphic. The synopsis is intended to explain the importance of the article to a broader readership

Response:

across the sciences. Please place your synopsis in the manuscript file after the TOC graphic and label as "Synopsis."

Response: As requested, we have added a synopsis to the manuscript.

5. TOC Graphic: Please label as "TOC Graphic".

Response: We have added the label "TOC Graphic" to the corresponding image.
